# Selective oxidation and quantification of m5C in ribonucleosides and RNA in an epigenetic context

**DOI:** 10.1039/d6cb00203j

**Published:** 2026-08-21

**Authors:** Kora Holschbach, Tobias Walter, Davide Guglielminotti, Annika Menke, David Schmidl, Doreen Reuter, Ken Kögel, Lola Stroh, Sabine Schneider, Pascal Giehr, Thomas Carell, Lena Daumann

**Affiliations:** a Lehrstuhl für Bioanorganische Chemie, Heinrich-Heine-Universität Düsseldorf Universitätsstraße 1 40225 Düsseldorf Germany lena.daumann@hhu.de; b Center for Nucleic Acid Therapeutics at the Department of Chemistry, Institute for Chemical Epigenetics, Ludwig-Maximilians-Universität München Butenandtstr. 5-13 81377 München Germany thomas.carell@lmu.de; c Department of Chemistry, Ludwig-Maximilians-University München Butenandtstr. 5-13 81377 München Germany

## Abstract

Selective modification of methylated bases in RNA and DNA is central to the field of epigenetics and furthermore fundamental for developing next-generation sequencing and liquid biopsy applications. While in DNA the detection and modification of 5-methylcytidine (5mdC) is fairly advanced, current strategies for detecting this modification (m5C) in RNA face significant technical challenges. Chemical conversion methods, such as bisulfite sequencing, remain widely used but induce substantial degradation of RNA and DNA, leading to incomplete conversion and reduced sequence complexity. Here we demonstrate the first example of a TET-biomimetic, selectively oxidizing the C–H bond of the methyl group in m5C in RNA. We also developed an isotope-labelling mass spectrometry-based strategy for quantification of the hydroxymethyl- (hm5C), formyl- (f5C) and carboxy-derivatives (ca5C), respectively. By changing substrate and iron complex ratios and the reaction time, the product spectrum can be tuned to obtain either more f5C or ca5C as a product. The iron complex’ ligand system can be further modified to fine-tune product yields and distribution.

## Abstract 2

Working with a newly developed UHPLC-MS/MS (QQQ) workflow providing absolute quantification of m5C and its oxidation products, we can show that our TET-biomimetic inorganic complex selectively carries out biomimetic oxidations of m5C-substitued RNA.

## Graphical abstract

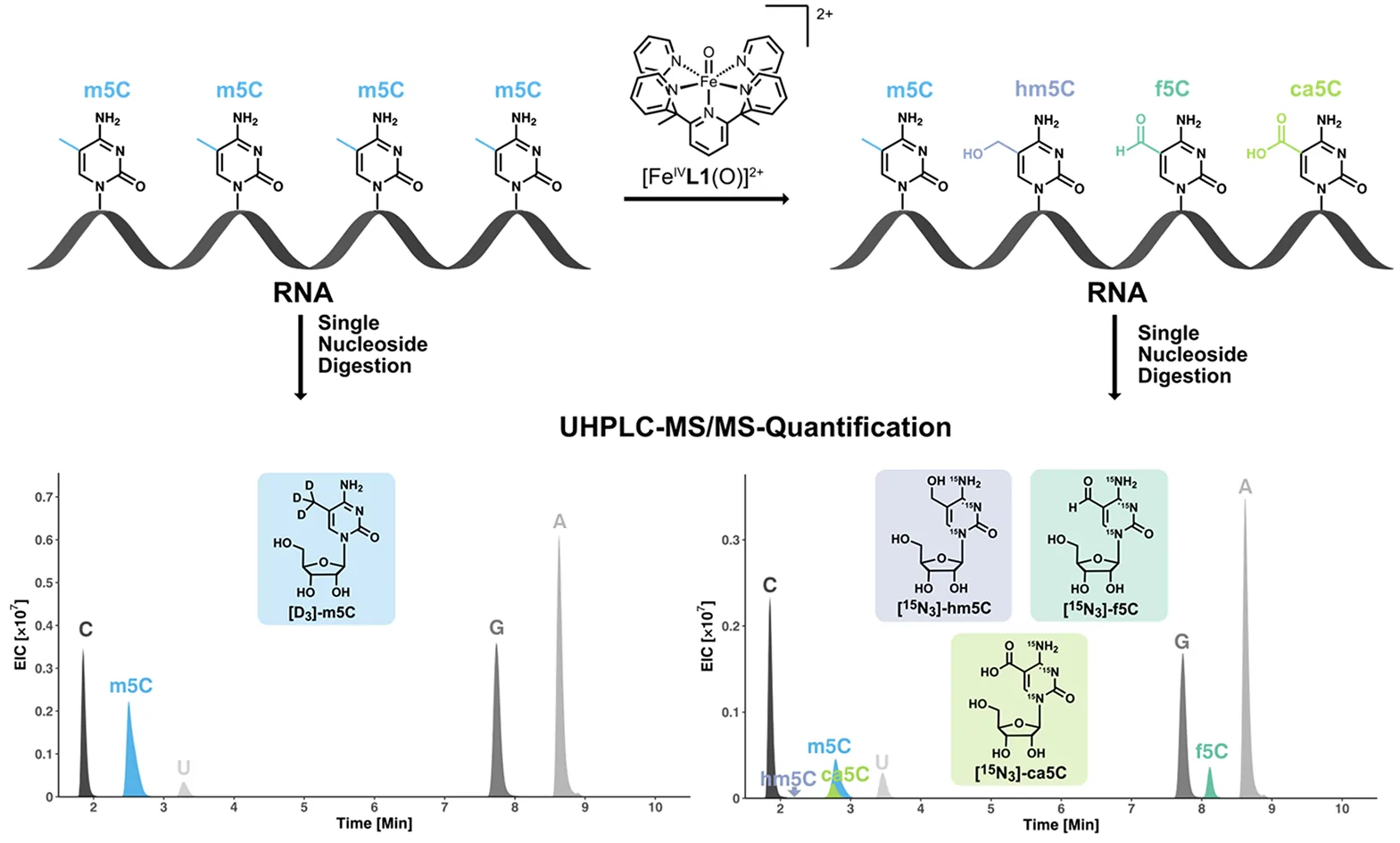

## Introduction

**Fig. 1:**
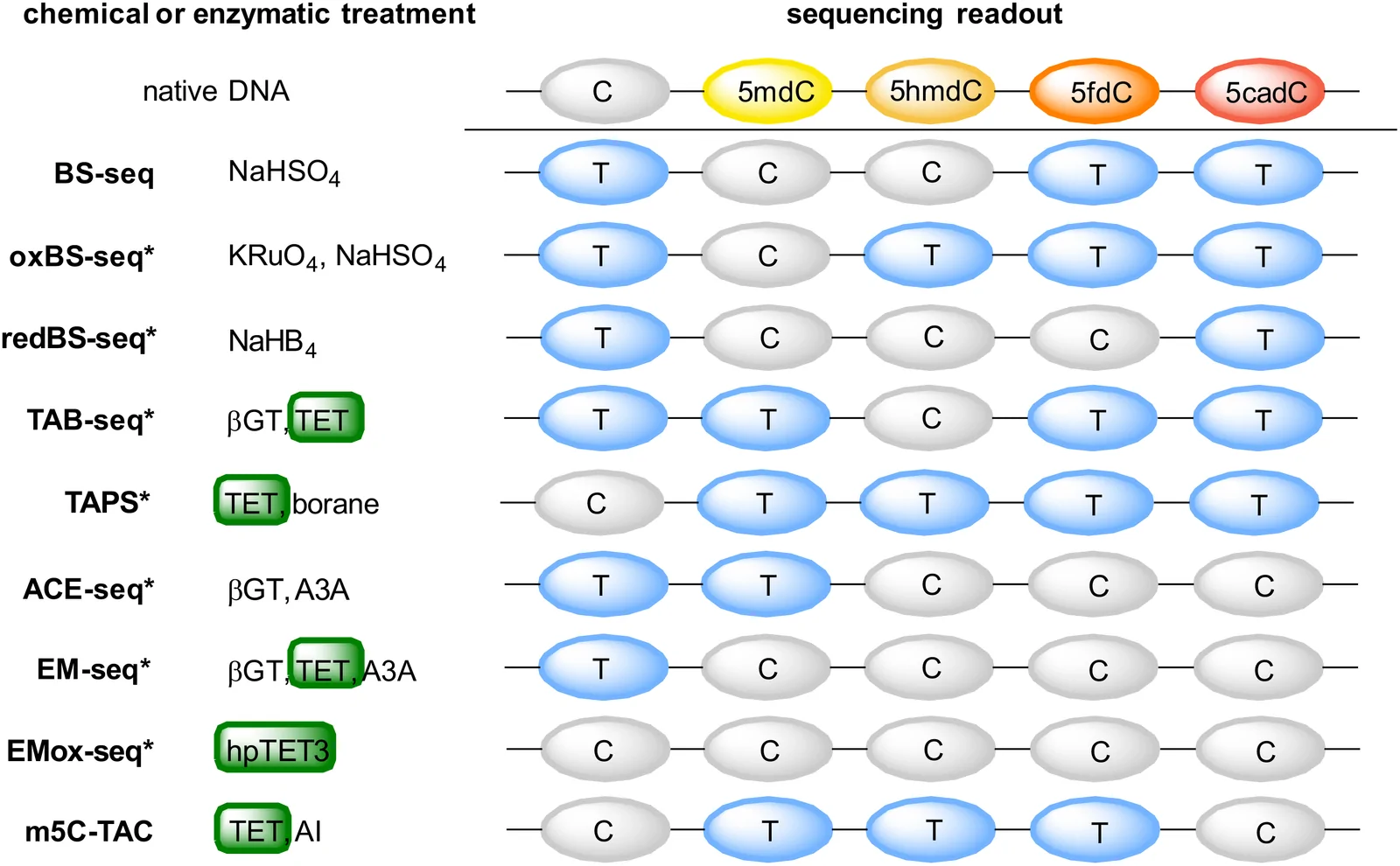
Overview of various currently applied sequencing methods for cytosine modifications with sample treatment and sequence readout. BS-seq = bisulfite sequencing, oxBS-seq = oxidative bisulfite sequencing, redBS-seq = reductive bisulfite sequencing, TAB-seq = TET-assisted bisulfite sequencing, βGT = β-glycosyltransferase, TAPS = TET-assisted pyridine borane sequencing, ACE-seq = APOBEC-coupled epigenetic sequencing, A3A = APOBEC3A, an AID/APOBEC deaminase, EM-seq = enzymatic methylation sequencing, EMox-seq = enzymatic methyl oxidation sequencing, m5C-TAC = m5C-TET-assisted chemical labelling, AI = azido derivative of 1,3-indandione. Involvement of TET enzyme within sequencing procedure is highlighted in green. * indicates lacking applicability to RNA.^3–12,15^

The four-base model for genomic information encrypted in DNA has been surpassed by epigenetic considerations, where modifications to the canonical nucleobases (A, T, G, C) are considered to convey genetic information additional to the sequence. 5-Methylcytosine (5mdC), for example, widely regarded as the “fifth base” in the genetic code, is involved in the silencing of genes and often involved in tumour development.^1,2^ In order to assess the entire information conveyed by the genomic DNA, sequencing methods are required which reliably differentiate between modified bases and thus provide accurate readout. Hence, various second-generation methods have been developed, such as bisulfite sequencing (BS-seq.), which is still widely considered to be the “gold standard” for epigenetic sequencing of 5mdC (Fig. 1). In this process, all non-(hydroxy)-methylated cytidines are converted to uridine while leaving 5mdC unchanged. Based on this reactivity, in combination with enzymatic and chemical methods a variety of bisulfite-based sequencing methods were established (*e.g.* oxBS-Seq, redBS-Seq, TAB-Seq, MAB-Seq).^3^ However, they all employ harsh reaction conditions destroying most of the input DNA and are prone to errors due to the loss of genetic information, leading to significant bioinformatical challenges. To overcome these limitations, other enzyme-based approaches have been introduced. TAPS (ten-eleven-translocation, TET enzyme assisted pyridine borane sequencing) is a bisulfite-free second-generation sequencing method that employs TET-enzymes for selective oxidation of 5mdC*via* 5-hydroxymethyl-deoxycytidine (5hmdC) and 5-formyl-deoxycytidine (5fdC) to 5-carboxy-deoxycytidine (5cadC), followed by a selective reduction step (of 5fdC and 5cadC) with pyridine borane to dihydrouracil (DHU). This in turn changes the coding potential of these modifications allowing for indirect readout of 5mdC. Using this reactivity with different combinations of chemical and enzymatic treatments a whole suite of pyridine borane-based epigenetic sequencing methods was developed (*e.g.* CAPS, CAPS+, TAPSβ).^4,5^ However, loss of aromaticity of the nucleobase, and the consequent loss of planarity, presents a challenge for most commonly employed polymerases, which can lead to systematic underdetection.^6^ Pure enzymatic epigenetic sequencing methods like APOBEC-coupled epigenetic sequencing (ACE-seq, for 5hmdC) and enzymatic methyl sequencing (EM-seq, for 5mdC and 5hmdC) offer milder reaction conditions and improved specificity, but they often suffer from either sequence bias or the requirement for single-stranded substrates (Fig. 1).^4,7–9^ Recently, more stable and reliable TET enzymes (hpTET3) have been reported; however, high sensitivity to reaction conditions and the requirement for elevated iron(ii) concentrations, which promote DNA decomposition, still prevail.^10–12^ So far, only third-generation sequencing methods are able to directly read-out epigenetic nucleobases (*e.g.* EMox-seq)^11^ though modification-induced signal changes. However, the absence of a PCR amplification step necessitates relatively high input material and, combined with elevated error rates and more complex and expensive workflows, makes these largely unsuitable for routine, high-throughput epigenetic sequencing applications.^13,14^

Most of the above-described 5mdC-sequencing methods established for DNA have not been successfully adapted to RNA-Seq workflows yet and hence, current strategies for detecting the m5C – modification in RNA – face significant technical challenges. Chemical conversion methods, such as bisulfite sequencing, remain widely used, but induce, as for DNA, substantial degradation of RNA and reduced sequence complexity. These effects complicate downstream mapping and computational analysis.^16^ Recently, a bisulfite-free approach for m5C detection in RNA has been described.^15^m5C-TAC-seq utilizes TET enzymes to oxidize m5C to 5-formylcytosine (f5C), followed by chemical labelling with an azido-derivative of 1,3-indandione (AI). This enables selective enrichment and induces C-to-T transitions during reverse transcription. However, m5C-TAC-seq remains semi-quantitative due to the limited and inconsistent efficiency of TET-mediated oxidation on RNA and its reliance on chemical enrichment.

Direct detection technologies, such as nanopore sequencing, offer the potential for PCR-free, label-free identification of RNA modifications. Yet, current implementations provide weak m5C signal resolution and still suffer from relatively high background noise, necessitating high sequencing depth and computational correction.^17^ Thus, new reagents to modify m5C are needed. Recently, Jin *et al.* presented an enzyme-free photo-chemical oxidation pathway to convert m5C to f5C (60% conversion, 11 h).^18^ Here, we focus on coordination compounds mimicking the natural functionality of TET or AlkB enzymes to help advance sequencing methods.

**Fig. 2:**
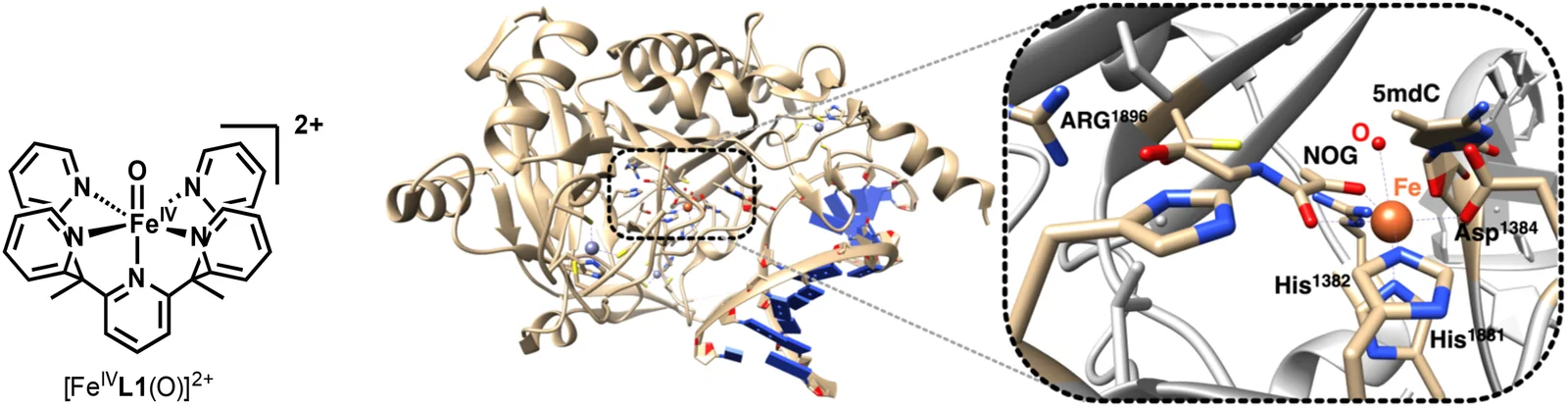
(left) Biomimetic iron(iv)-oxido complex: this complex was first synthesized by Chang *et al.*^19^ (right) Crystal structure of a TET2-DNA complex (PDB code: 4NM6). The active center is depicted, containing an iron(ii) ion coordinated by various amino acid residues (His1382, Asp1384 His1881), NOG (*N*-oxalyl glycine; succinate analog), and oxygen. Moreover, the 5mdC-DNA substrate and the spatial orientation towards the Fe(iv)-oxido species is depicted.^20–23^

The TET enzyme-biomimetic properties of the iron(iv)-oxido complex [Fe^IV^L1(O)]^2+^ with nucleobases or short DNA oligomers were described by Jonasson and Daumann in 2019 and Schmidl *et al.* in 2021 (Fig. 2), respectively.^20,24^ Supported by a pentapyridine ligand backbone, the active iron(iv)-oxido complex (resembling the active species in TET enzymes) is able to oxidize 5mdC*via*5hmdC and 5fdC to the carboxy-derivative (5cadC), both as a monomeric nucleoside and in oligonucleotides. Whilst we have shown that the complex does not differentiate T from 5mdC at the nucleobase level and consequently also not within DNA, this limitation does not apply to RNA due to the presence of the base uracil (U) instead of T.^25^ While 5mdC is the most abundant base modification in DNA, the m5C modification is less common in RNA (0.43% m5C in the human transcriptome).^26,27^m5C is associated with increased mRNA stability and the formation of secondary structure formation elements, such as stem-loop motifs.^28,29^ It was demonstrated by Huang *et al.* that the same oxidation cascade observed for 5mdC (to 5cadC) also applies to m5C, catalyzed by TET enzymes.^30^ Even though RNA is notoriously known for high instability (the additional hydroxy group on the ribose sugar facilitates phosphodiester bond cleavage) the described iron(iv)-oxido complex is perfectly suitable for modifications of methylated RNA bases.^28^ Considering the iron complex [Fe^IV^L1(O)]^2+^ already contains the active iron(iv)-oxido species, no excess of iron is introduced to the reaction mix, which ensures conditions optimized for RNA stability and prevents side reactions such as Fenton chemistry. In this work, we show that the conversion of m5C to the carboxy-derivative (ca5C) is possible with [Fe^IV^L1(O)]^2+^ in RNA, even to levels of 80% conversion of original oligonucleotide, though limitations regarding oligonucleotide length and sequence structure apply. To quantify oxidation products of m5C after treatment with the iron reagent, we developed a mass spectrometry-based strategy using isotopically labelled cytidine standards.

## Results and discussion

### Synthesis of modified cytidine ribonucleosides

**Scheme 1:**
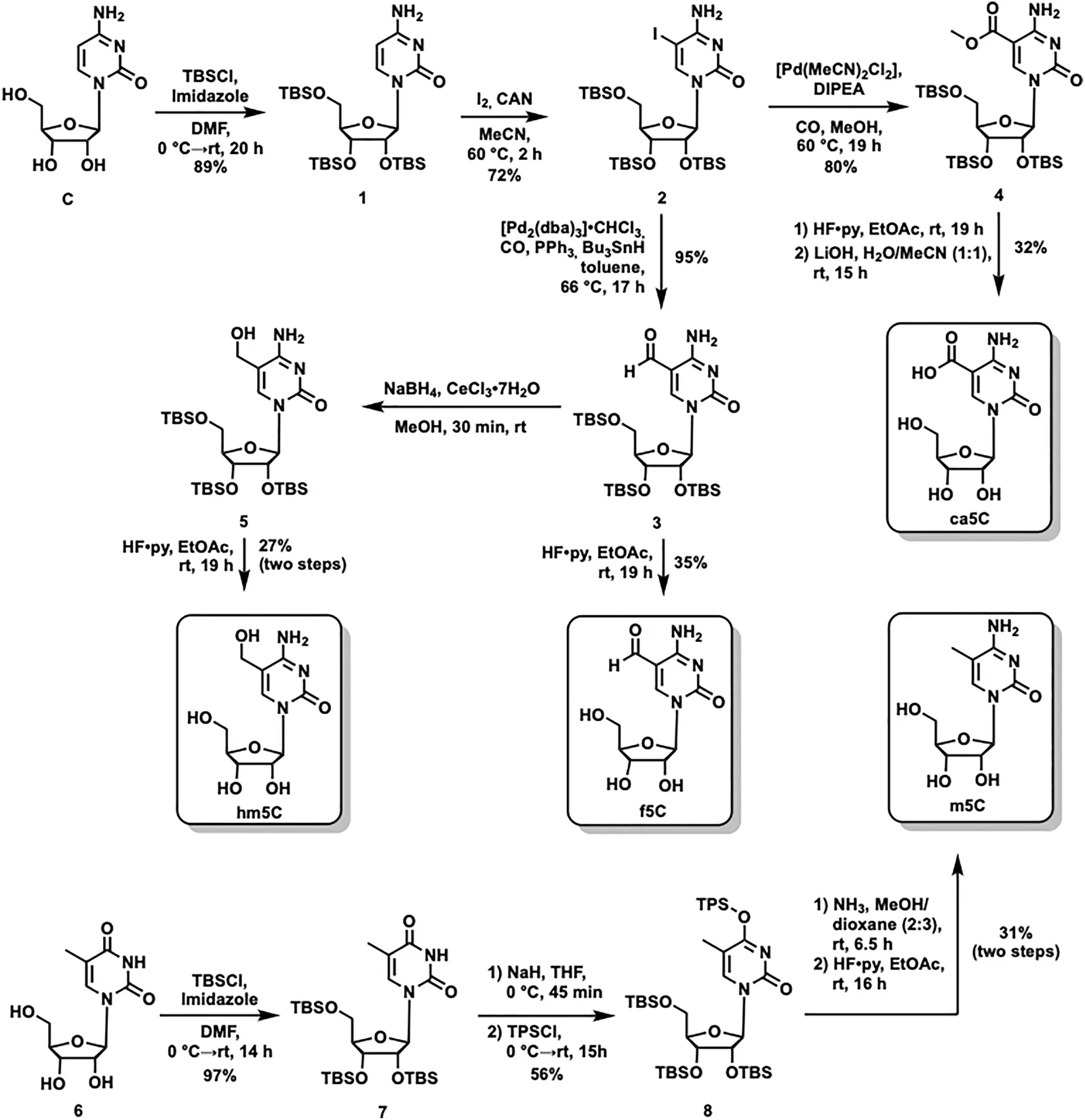
Synthetic route towards the desired reference compounds m5C, hm5C, f5C and ca5C. TBS: *tert*-butyl dimethyl silyl, CAN: cerium(iv) ammonium nitrate, py: pyridine, DIPEA: diisopropylethylamine, dba: dibenzylideneacetone, TPS: 2,4,6-triisopropylbenzenesulfonyl.^31–35^

All required ribonucleosides were synthesized similarly as reported previously for their deoxyribonucleoside counterparts (Scheme 1).^31–35^ In brief, silylated cytidine (1) was transformed into the corresponding 5-iodocytidine precursor (2) by oxidative iodination with iodine and cerium(iv) ammonium nitrate (CAN). Palladium-catalysed Stille-type cross coupling of this compound in the presence of tributyltin hydride and carbon monoxide followed by *tert*-butyl dimethyl silyl (TBS) deprotection afforded f5C. Similarly, the corresponding methoxycarbonylated species (4) was obtained under basic conditions in methanol. Ester saponification with lithium hydroxide and deprotection afforded compound ca5C. Through a Luche-type reduction of TBS-protected f5C (5) and subsequent cleavage of the silyl ethers, hm5C was obtained. m5C was synthesized from TBS-protected 5-methyluridine which was then converted to the respective cytidine species *via* a 2,4,6-triisopropylbenzenesulfonyl intermediate, amination using ammonia and final TBS cleavage.

### Conversion of ribonucleosides

**Scheme 2:**
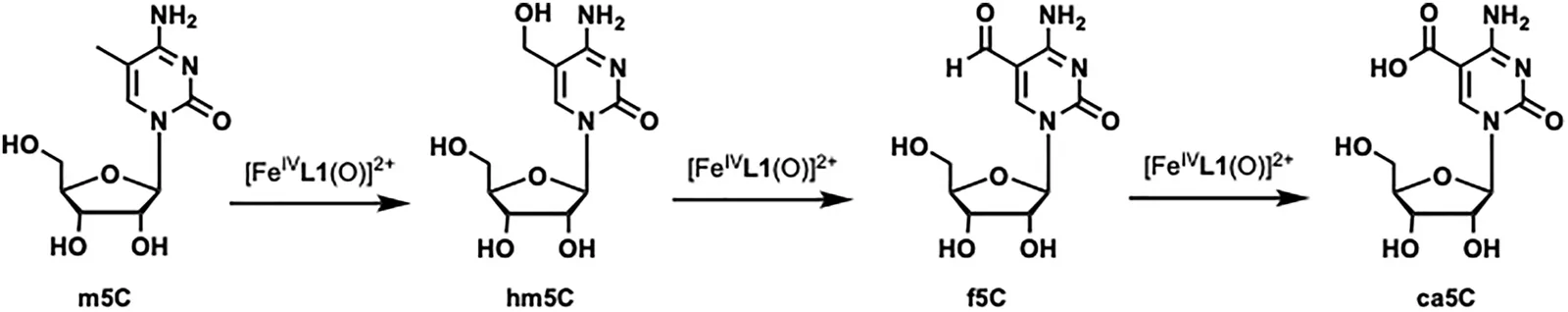
Successive oxidation of the ribonucleoside m5C by the complex [Fe^IV^L1(O)]^2+^, yielding hm5C, f5C and ca5C.

[Fe^IV^L1(O)]^2+^ was previously successfully established as a biomimetic complex mimicking TET enzyme activity towards methylated DNA bases (Fig. 2), but these experiments also showed side reactions with dT-containing oligomers.^20,24,25^ Given (unmethylated) rU is incorporated in RNA instead of dT, it was hypothesized that RNA would display less unwanted side reactions upon treatment with the complex. Thus, the ribonucleoside m5C was first reacted with [Fe^IV^L1(O)]^2+^ to assess the oxidation reaction on the nucleoside level (Scheme 2).

**Fig. 3:**
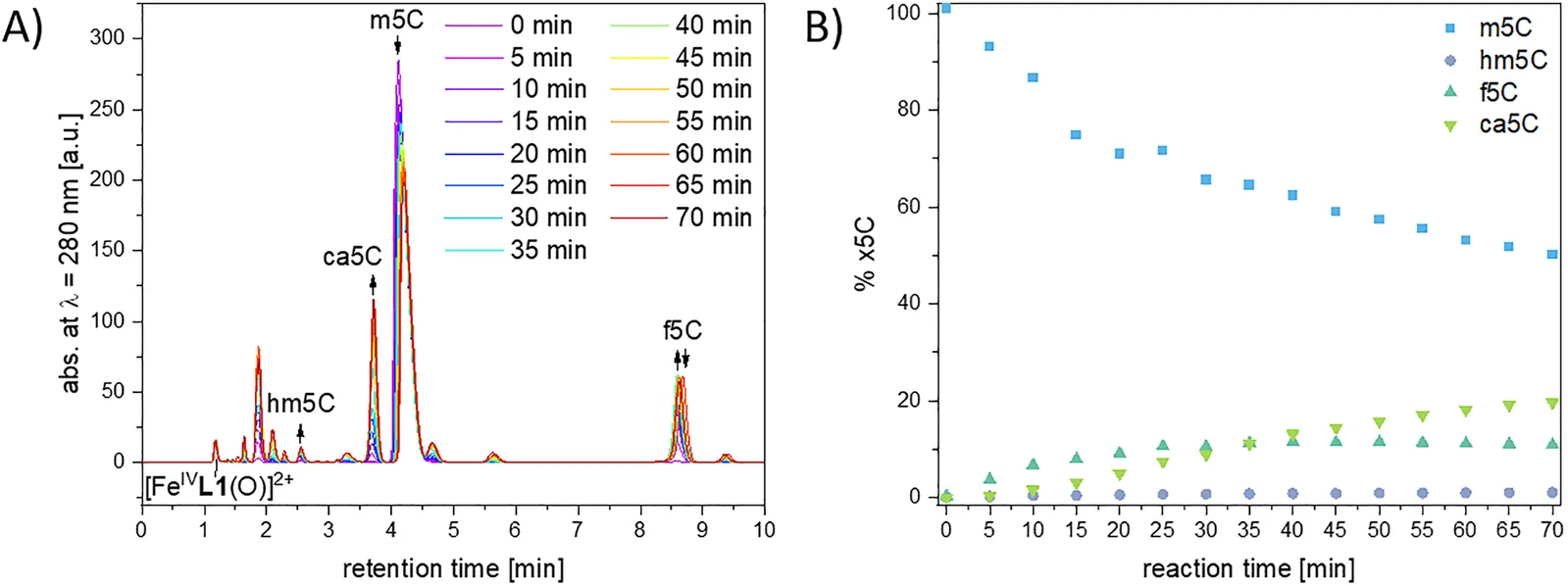
(A) HPLC chromatograms of the obtained mixtures from a reaction of m5C with [Fe^IV^L1(O)]^2+^ at different reaction times. The concentration of m5C is decreasing over time, whilst the concentrations of hm5C, f5C and ca5C are increasing. (B) Species distribution of cytidine derivatives over time. Conditions (Table S1): [m5C] = 1 mM, [Fe^IV^L1(O)]^2+^ = 5 mM, H_2_O, 25 °C.

Following incubation of the biomimetic complex with m5C, the formed products were identified and quantified by analytical high performance liquid chromatography (HPLC) using the expected oxidation products hm5C, f5C and ca5C as reference samples (Fig. 3). For this purpose, an HPLC method was developed where all potential cytidine and cytosine derivatives present in the reaction mixture showed baseline-separated retention times (see Table S1). Calibration curves were obtained *via* a dilution series of a mixture of all compounds in concentrations ranging from 0.0012 to 0.099 mg mL^−1^ (see Fig. S2 and Fig. 3A).

The reaction of [Fe^IV^L1(O)]^2+^ with m5C was carried out in water under ambient conditions over a range of 70 min. Every 5 min, a sample was taken from the reaction mixture, the iron species separated out by filtration through silica to stop the reaction at defined time points, and the filtrate lyophilized. The resuspended residue was subjected to analytical HPLC and the resulting absorption peaks quantified against the calibration set (Fig. 3B).^20^ All four ribocytidine derivatives m5C, hm5C, f5C and ca5C were detected in the reaction mixture.

As expected, the amount of m5C starting material decreased over time and a mixture of oxidation products formed. The amount of hm5C stayed very low throughout the reaction, suggesting a fast transformation into the subsequent oxidation product f5C. The amount of f5C in contrast accumulated slightly in the beginning and then decreased minimally, evening out around a constant 10%. Bond dissociation energy (BDE) calculations performed for C–H bond at the C5 methyl group of the deoxynucleobases by Liu *et al.* support this observation: compared to the 5mdC BDE (387.3 kJ mol^−1^) and the 5fdC BDE (401.0 kJ mol^−1^), the 5hmdC BDE is lowest with 367.4 kJ mol^−1^.^36^ The main species present at the end of the monitored reaction time was ca5C, which is in line with observations made by Schmidl *et al.* for the reaction of [Fe^IV^L1(O)]^2+^ with the corresponding deoxyribonucleoside 5mdC.^20^ The low level of total starting material consumption can be attributed to limiting amounts of iron complex present in the reaction mixture. Five equivalents of [Fe^IV^L1(O)]^2+^ were employed to each one equivalent of ribonucleoside, but the amount available for reaction with m5C should be considered halved due to a competing comproportionation side reaction.^37^ Nevertheless, based on these encouraging results, we moved on to longer oligomeric RNA structures.

### Synthesis of isotopically labelled ribonucleosides as internal standards for UHPLC-MS/MS (QQQ)-based quantification

**Scheme 3:**
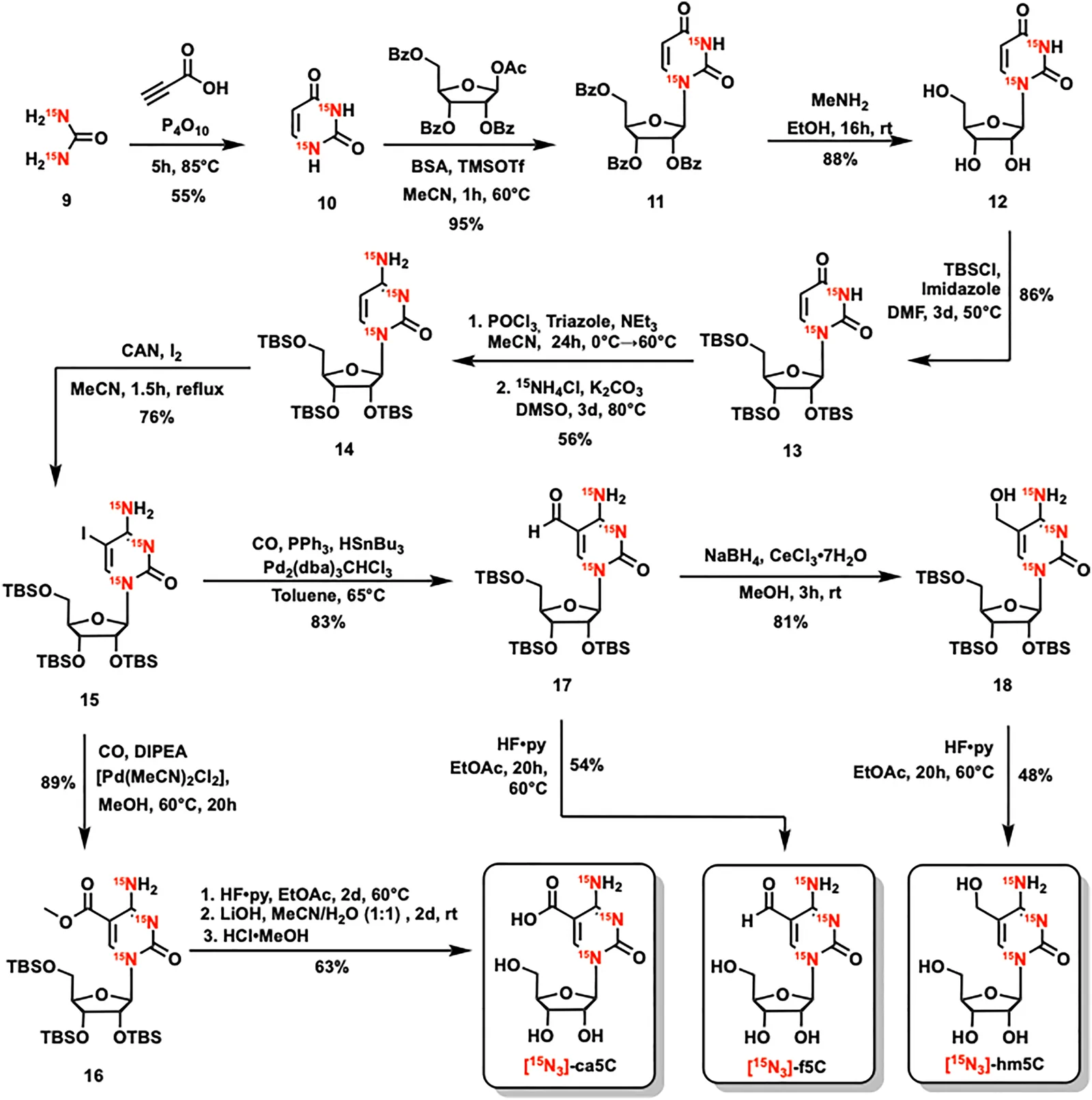
Synthetic route towards the isotopically labelled ribonucleosides [^15^N_3_]-m5C, [^15^N_3_]-hm5C, [^15^N_3_]-f5C and [^15^N_3_]-ca5C. TBS: *tert*-butyl dimethyl silyl, CAN: cerium(iv) ammonium nitrate, py: pyridine, DIPEA: diisopropyl ethyl amine, dba: dibenzylideneacetone, BSA: bis(trimethylsilyl) acetamide, TMSOTf: trimethylsilyl trifluoromethanesulfonate.^31–33,35,38–40^

For accurate quantification of the oxidation products (hm5C, f5C and ca5C) of m5C-containing RNA using a tandem mass spectrometry-based approach, a set of isotopically labelled ribonucleosides was synthesized to serve as an internal standards. To obtain the [^15^N_3_]-cytidine precursor (14), [^15^N_2_]-uracil (10) was first synthesized from [^15^N_2_]-urea (9) using phosphorus pentoxide and propiolic acid. Glycosylation with benzoyl-protected ribose and subsequent deprotection yielded [^15^N_2_]-uridine (12), which was TBS-protected and then converted to the [^15^N_3_]-cytidine species (14) using triazole and POCl_3_ and subsequent treatment with ^15^NH_4_Cl. The remainder of the synthetic route for the isotopically labelled cytidine standards was carried out as described above for the non-labelled ribonucleosides (Scheme 3).^31–33,35,38–40^

### Conversion of m5C in oligonucleotides

With the full set of required standards and internal standards available, we developed a high-performance liquid chromatography-tandem mass spectrometry (HPLC-MS/MS, QQQ)-based method to quantify the oxidation products generated by reaction of the iron(iv)-oxido complex [Fe^IV^L1(O)]^2+^ with RNA. In brief, each digested sample was spiked with a mix of the internal standards (see Table S3). The HPLC method was optimized to achieve sufficient chromatographic separation for reliable LC-MS/MS quantification of the oxidation products, utilizing a C18 column with a gradient elution profile tailored to the polarity of the analytes (Table S3). Data acquisition was performed in multiple reaction monitoring (MRM) mode allowing for highly sensitive and selective detection of the analytes. The analytes were analyzed using calibration curves generated from known concentrations of the respective ribonucleosides (1 : 2 dilution series; 10.0 pmol–4.9 fmol for modifications and 100 pmol–49 fmol for canonical nucleosides) and normalized to their respective internal standards, enabling accurate quantification of the oxidation products in the samples.^41^

**Fig. 4:**
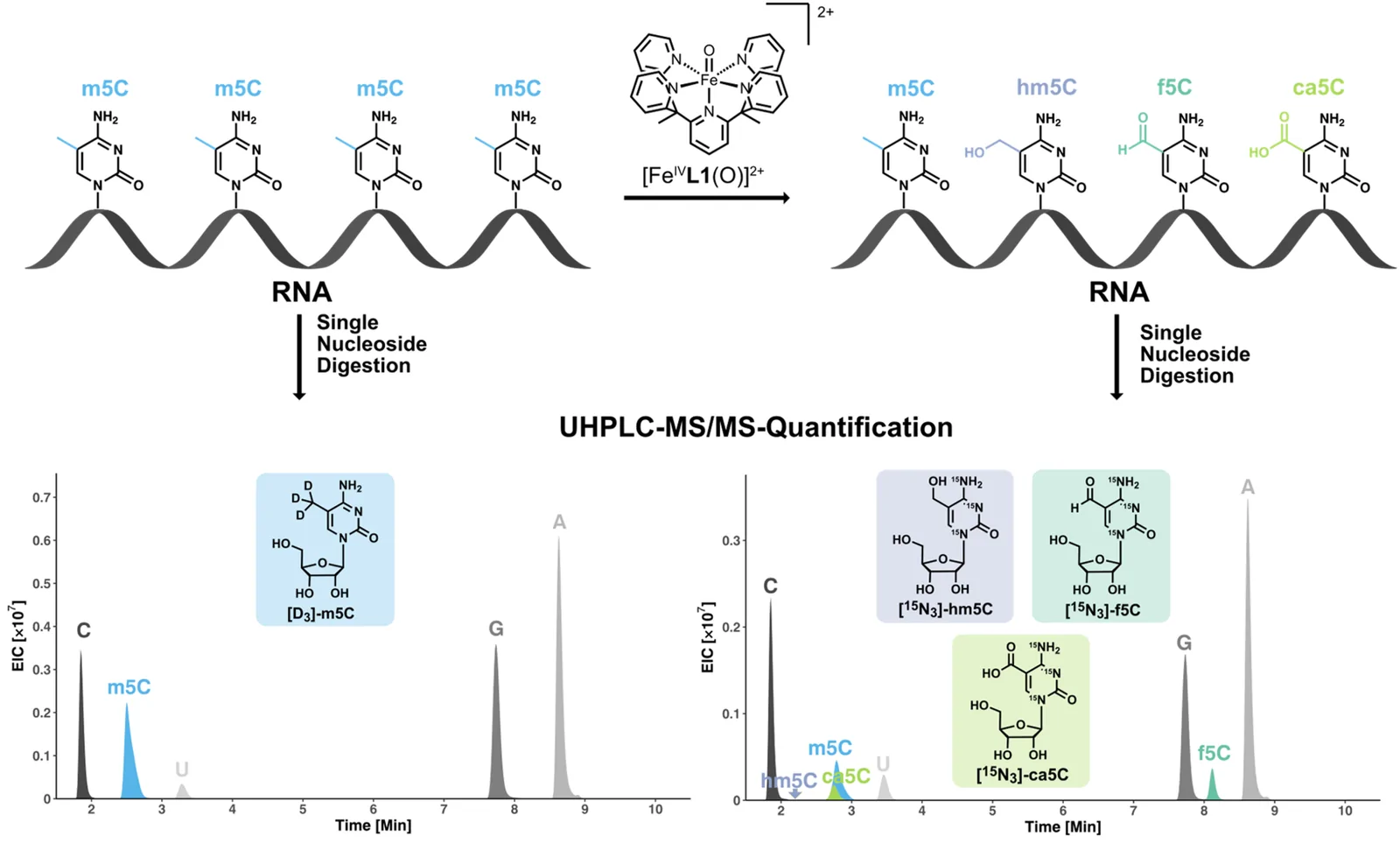
Principle of the LC-MS/MS-based quantification of m5C, hm5C, f5C, and ca5C in [Fe^IV^L1(O)]^2+^-treated RNA samples, including structures of the respective isotopologs that were used as internal standards. The position of hm5C is indicated by an arrow, since the low abundance in [Fe^IV^L1(O)]^2+^-treated samples (see Fig. 5) together with its lower ionization efficiency compared to the other analytes, results in a peak that is obscured in the current depiction of the chromatogram due to the display scale.

**Fig. 5:**
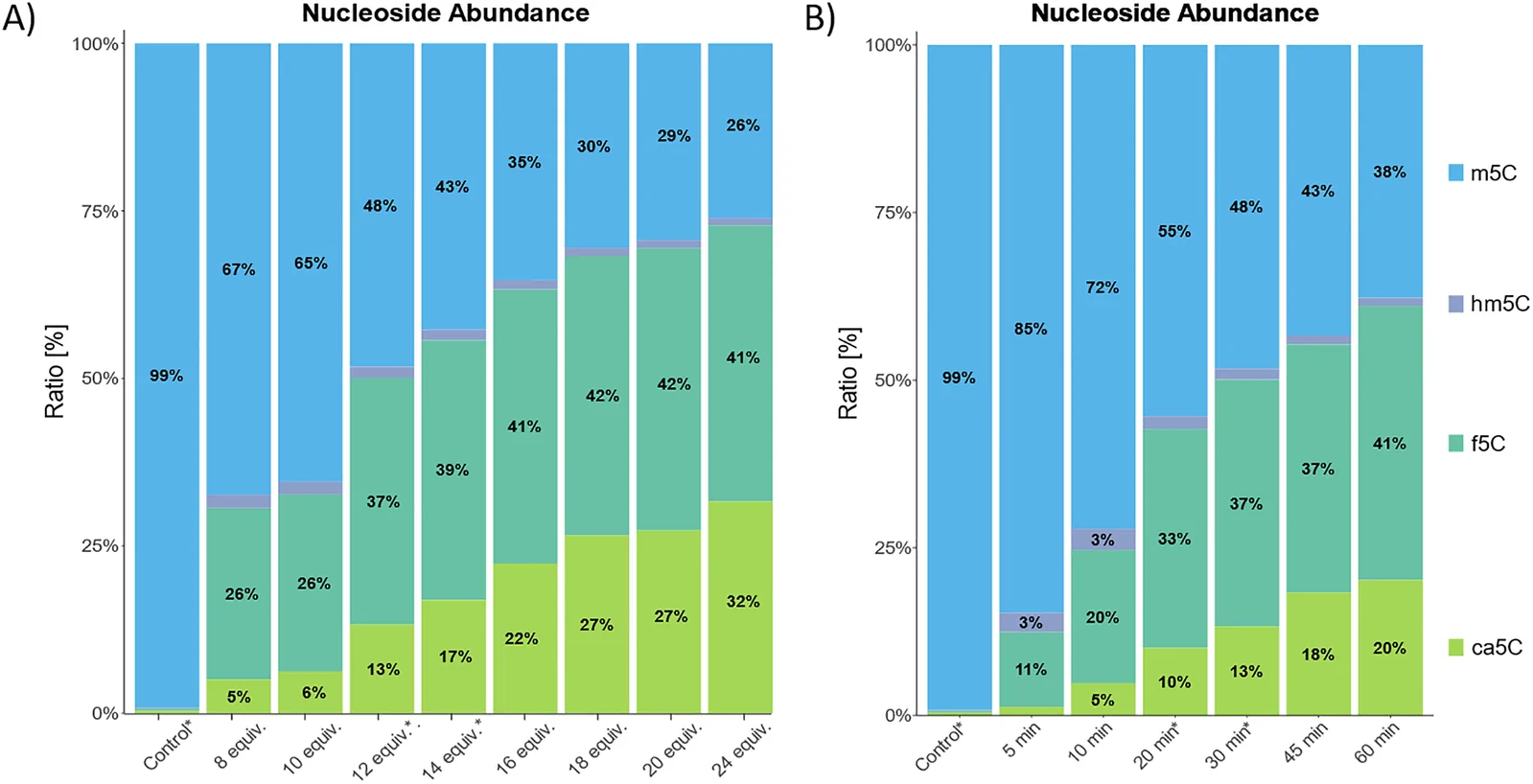
To find optimal reaction conditions, oxidation product ratios across a range of [Fe^IV^L1(O)]^2+^ equivalents and reaction time points were compared. (A) Variation of [Fe^IV^L1(O)]^2+^ equivalents: RNA 1 was reacted with 8 to 24 equivalents [Fe^IV^L1(O)]^2+^ for 30 min. Strand intactness was controlled for by MALDI-TOF-MS before digestion of RNA 1 to single nucleotide level and analysis *via* UHPLC-MS/MS (QQQ, Table S4). Two sets of independent replicates were collected (8 to 12 *vs.* 12 to 24 equivalents), and * indicates the combined average of both data sets. (B) Variation of reaction times: RNA 1 was reacted with 12 equivalents [Fe^IV^L1(O)]^2+^ for 5 to 60 min. Strand intactness was controlled for by MALDI-TOF-MS before digestion of RNA 1 to single nucleotide level and analysis *via* UHPLC-MS/MS (QQQ, Table S5). Two sets of independent replicates were collected for timepoints 20 and 30 min, and * indicates the combined average of both data sets. Conditions: [RNA 1] = 10 µM, H_2_O, 20 °C.

It was subsequently investigated how the [Fe^IV^L1(O)]^2+^ complex would enable the oxidation of m5C located in a 10-mer single-stranded RNA (5-AUUG(m5C)CACUG-3′, RNA 1, 10 µM). RNA 1 was treated with [Fe^IV^L1(O)]^2+^, added from a freshly prepared 1 mM stock solution, and the iron species removed from the sample by centrifugal size-exclusion filtration with a 3 kDa cut-off. Analysis was performed by RNA digestion followed by single nucleoside quantification *via* UHPLC-MS/MS (triple quadrupole LC-MS, QQQ, Fig. 4), as well as by verifying strand intactness using MALDI-TOF-MS. In order to optimize reaction conditions and maximize oxidation of m5C, reaction time and [Fe^IV^L1(O)]^2+^ equivalents were investigated as tuneable variables (Fig. 5). The reaction conditions used for the oxidation of m5C ribonucleoside were applied to oligomeric ssRNA 1, but minimum 8 equivalents of [Fe^IV^L1(O)]^2+^ were employed to compensate for comproportionation reaction effects.^37^ Upon reaction of the [Fe^IV^L1(O)]^2+^ complex with RNA 1, a turnover of substrate m5C to f5C and ca5C was observed.

#### Varying equivalents of iron reagent

Analysis of the oxidation product ratios compared to starting material RNA 1 showed an increase in overall oxidation products formed, depending on the [Fe^IV^L1(O)]^2+^ equivalents (Fig. 5A). From 8 to 12 equivalents of [Fe^IV^L1(O)]^2+^, a steep increase from 32.6% (8 equivalents) to 51.8% (12 equivalents) of total formed oxidation products was observed, corresponding to a nearly 59% increase in formed oxidation products for a fifty percent increase in [Fe^IV^L1(O)]^2+^ equivalents. Increasing from 12 to 24 equivalents (doubling [Fe^IV^L1(O)]^2+^ amounts), another 45% increase was detected, thus any increase in [Fe^IV^L1(O)]^2+^ beyond 12 equivalents had a reduced effect on conversion rates. It was decided to mainly investigate 12 or 24 equivalents in subsequent experiments, as these amounts represent a good balance between required [Fe^IV^L1(O)]^2+^ input and maximum m5C oxidation (Fig. 5A). Higher [Fe^IV^L1(O)]^2+^ stoichiometries (48 equivalents) led to increased product decomposition in a preliminary test and were not further investigated. In good agreement with the data for m5C nucleoside (Fig. 3) and for 5hmdC-containing DNA oligomers,^20^ the hydroxy-form was short-lived and only formed in low amounts (1–2%) in the reaction mixture (Fig. 5A).^15^ Interestingly, in the presence of 16 equivalents and more, the f5C-containing RNA product made up for constant 40–42% of the total reaction products, whilst the relative ca5C content kept increasing with rising [Fe^IV^L1(O)]^2+^ equivalents, while the m5C content kept decreasing with overall increased levels of ssRNA 1 oxidation. This suggests a relatively stable intermediate state for f5C which could potentially be exploited by further functionalisation at the aldehyde position.^42^ Trapping of the f5C aldehyde intermediate is of heightened interest for sequencing application purposes, as various biocompatible derivatisation approaches are known. Yan *et al.* showed proof-of-concept for the functionalisation of 5fdC in combination with decatungstate photocatalytic oxidation reaction, and Schmidl *et al.* showed that malononitrile derivatisation of 5fdC can be used for direct sequencing readout of the formyl-modification, reducing the number of processing steps required.^42,43^

#### Optimizing product distribution over time

An investigation into the time dependence of the oxidation reaction revealed that longer reaction times of RNA 1 with 12 equivalents [Fe^IV^L1(O)]^2+^ led to increased oxidation product formation (Fig. 5B). Less than 40% of the original m5C substrate remained after 60 min, whilst MALDI-TOF-MS indicated high stability of the oligomer towards the reaction conditions. For 24 equivalents [Fe^IV^L1(O)]^2+^, extension of the reaction time beyond 45 min did not lead to increased oxidation performance of the [Fe^IV^L1(O)]^2+^ (Fig. S5), potentially due to comproportionation reaction effects. Based on these data and considering practical implications, all subsequent experiments were conducted for 60 min.

#### Centrifugal filtration product loss and validation of strand intactness

A centrifugal filtration step with a 3 kDa size cut-off was implemented for removal of small side-reaction products or oligomer decomposition products from the reaction mixture before analysis. The highest number of decomposition products would be expected with high amounts of [Fe^IV^L1(O)]^2+^ and extended reaction times. Accordingly, the flow-through of a reaction with 24 equivalents [Fe^IV^L1(O)]^2+^ for 60 min was collected, combined, lyophilized and analysed by MALDI-TOF-MS to control for RNA 1 decomposition. MALDI-TOF-MS analysis of the flow-through showed only background noise (Fig. S3) and no decomposition product peaks were observed in the flow-through. Additionally, to identify strand intactness for the oxidation products of RNA 1, the reaction was scaled up 10×, whilst all other reaction conditions were kept constant. After analytical HPLC separation, product identities were verified by MALDI-TOF-MS (Fig. S4). This indicated that the major reaction products formed were indeed those of [Fe^IV^L1(O)]^2+^-mediated m5C oxidation in RNA 1.

#### Impact of RNA secondary structure on m5C conversion

**Fig. 6:**
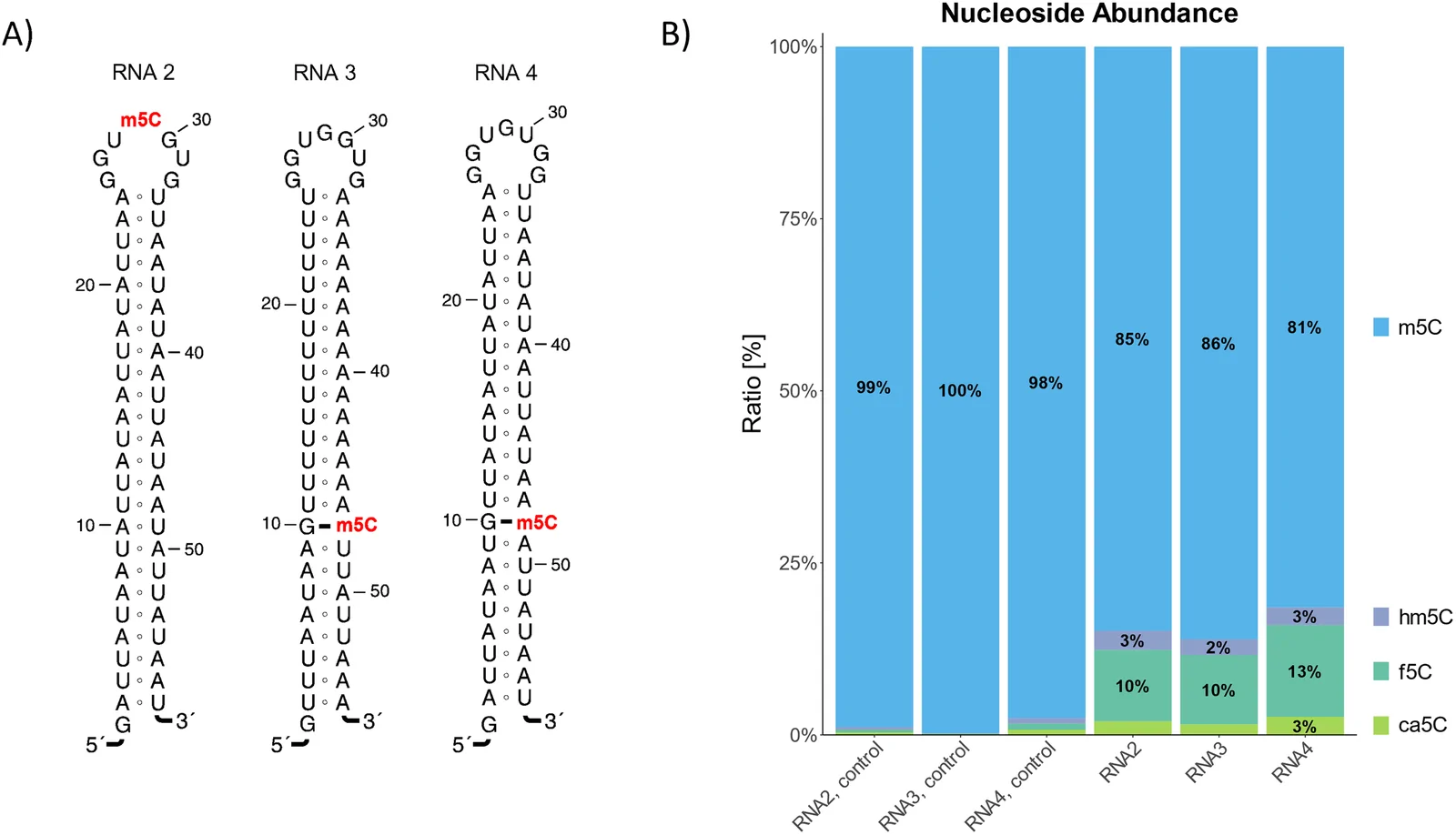
(A) RNA 2–4 were generated by *in vitro* transcription forming a hairpin structure due to complementary sequences at the 5′ and 3′ ends (Tables S9 and S10). m5C was either placed in the open loop (RNA 2) or within the duplex region (G-m5C base pairing, RNA 3, 4). (B) An investigation into oxidation product ratios for RNA 2–4 with 24 equivalents [Fe^IV^L1(O)]^2+^ was performed in triplicate. Strand intactness was controlled by MALDI-TOF-MS before digestion of RNA to single nucleotide level, and analysis *via* UHPLC-MS/MS (QQQ, Table S6). Conditions: [RNA] = 10 µM, H_2_O, 20 °C.

5mdC present in dsDNA was previously reported to show a lower conversion rate compared to ssDNA when reacted with [Fe^IV^L1(O)]^2+^, presumably because the nucleobases are less accessible for oxidation in the double strand.^44^ In order to investigate the efficiency of m5C oxidation in RNA forming secondary structures, 55-mer RNA strands with complementary sequences at the 5′ and 3′ ends that enable hairpin formation were prepared by *in vitro* transcription (Fig. 6 and Fig. S7, Tables S9, S10). We hypothesized that the complex would be able to oxidize the openly accessible m5C base located in the loop region (RNA 2), but would be less efficient with the m5C positioned in the double strand (RNA 3 and RNA 4).

As expected, the complex showed very low oxidation for m5C within the duplex region (RNA 3, 4, Fig. 6). However, when the m5C was positioned in the loop structure (RNA 2), a small decrease in oxidation products could be observed compared to RNA 4, suggesting that the complex is partially sensitive to secondary structure, although not in the hypothesized manner. This highlights that the complex is active for longer RNA substrates, and optimisation of the reaction conditions might be possible to achieve higher turnover numbers. As previously observed with DNA,^20^ the iron complex performs better with single-stranded substrates. To break the secondary structure of RNA, formamide can be used at elevated temperature.^45^ To investigate whether our complex could withstand these conditions and whether it would be able to oxidize m5C in denatured RNA, RNA 2–4 were preincubated at 85 °C over 8 min with an excess of formamide before dilution and addition of [Fe^IV^L1(O)]^2+^. The results of the formamide-treated strands revealed that no oxidation occurred, which can be attributed to the complex decomposition under high formamide concentration conditions. Kinetic analysis of the complex stability (tested concentration ratios [[Fe^IV^L1(O)]^2+^] : [formamide] = 1 : 3–1 : 50) showed rapid decomposition of the complex within seconds, which is orders of magnitudes faster than the intended m5C oxidation reaction. Thus, whilst formamide is a useful tool for the denaturation of RNA secondary structure when working with TET2 enzymes, it cannot be used in combination with TET-mimetic iron complex [Fe^IV^L1(O)]^2+^.

#### Optimisation of the complex reactivity profile

**Fig. 7:**
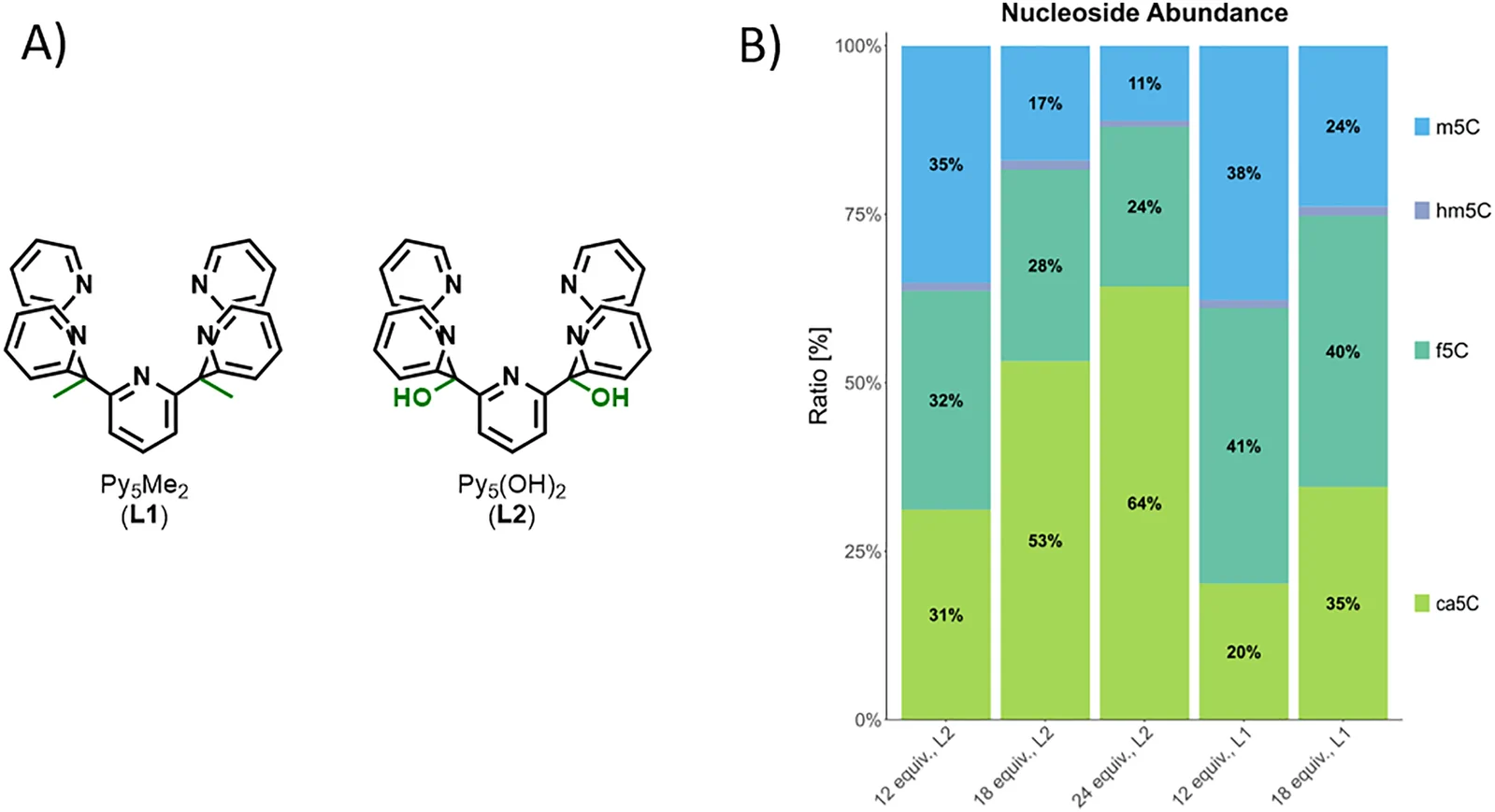
(A) The two ligand systems L1 und L2 used to generate an iron(iv)-oxido species of the type [Fe^IV^L_*x*_(O)]^2+^. (B) RNA 1 was reacted with [Fe^IV^L2(O)]^2+^ at given amounts of complex for 60 min in triplicates. For condition “RNA, 18 equivalents, L2” only duplicates were taken into consideration. Strand intactness was controlled by MALDI-TOF-MS before digestion of RNA 1 to single nucleotide level, and analysis *via* UHPLC-MS/MS (QQQ, Table S7). For comparison, data collected for [Fe^IV^L1(O)]^2+^ reaction with RNA 1 for 60 min is shown alongside. Conditions: [RNA 1] = 10 µM, H_2_O, 20 °C.

Considering that the [Fe^IV^L1(O)]^2+^ has a tuneable organic ligand system, the catalyst can be further optimized to yield increased m5C oxidation rates. By changing the pentapyridine-derived ligand system to include a hydroxy group ((Py_5_(OH)_2_), L2, Fig. 7) instead of the methyl substituent (L1), the complex activity can be altered. Because [Fe^IV^L2(O)]^2+^ is less stable than [Fe^IV^L1(O)]^2+^ at room temperature, UV-Vis spectra of the complex species were recorded at the starting time point of the reaction with RNA 1 to compare the effective concentration of catalytically active Fe^IV^ species in the sample.^37^ It was noted that, presumably due to storage and handling conditions, the concentration of catalytically active Fe^IV^ species varies for both ligand systems. To circumvent this issue, only data collected on the same day was directly compared for [Fe^IV^L2(O)]^2+^.

When treating RNA 1 ([RNA 1] = 10 µM) for 60 min, the [Fe^IV^L2(O)]^2+^ complex outperformed [Fe^IV^L1(O)]^2+^ (Fig. 7). Whilst the total conversion of m5C by [Fe^IV^L2(O)]^2+^ was only slightly higher at 12 equivalents, a notable difference in oxidation product distribution could already be observed. [Fe^IV^L2(O)]^2+^ generated about one third more ca5C oxidation product than [Fe^IV^L1(O)]^2+^ but less f5C intermediate. It is apparent, that further tuning of the ligand scaffold enables the generation of high f5C and ca5C ratios, which in turn could help establish a novel m5C-Seq method for RNA, analogously to the TAPS sequencing technique for DNA.

## Summary

DNA and RNA modifications are increasingly recognized as valuable molecular markers in biomedical research and clinical diagnostics. These applications critically depend on reliable and efficient conversion chemistries, as input material is often limited. Consequently, bisulfite-free alternatives have gained considerable attention due to their milder reaction conditions and their potential to preserve the integrity of nucleic acid samples.

However, current enzymatic approaches, particularly TET-based oxidation methods, continue to exhibit inherent limitations. TET enzymes display a pronounced sequence bias, acting predominantly on CpG dinucleotides, with markedly reduced activity outside the CpG context and only limited turnover on RNA substrates. In addition, as large Fe(ii)/2-oxoglutarate-dependent dioxygenases, TETs are highly sensitive to storage conditions, freeze–thaw cycles, buffer composition, and the redox state of the reaction environment. These factors collectively complicate their reproducible use in routine laboratory workflows and diagnostic settings.

These limitations highlight the need for robust, enzyme-independent alternatives. The chemically driven oxidation enabled by our iron complex provides a stable, sequence-independent approach that has the potential to overcome these limitations and thus represents a promising strategy toward standardized, broadly applicable protocols for research and diagnostic applications.

We have successfully shown how an inorganic complex can selectively carry out biomimetic oxidations of m5C-substituted RNA. By changing substrate and complex ratios as well as the reaction time, product formation can be tuned to obtain either more f5C or ca5C as a product. The pentapyridine-supported iron complex offers additional scope for modification toward this purpose with a hydroxy group variant showing increased turnover and ca5C formation. We confirmed that the iron complexes only minimally affected strand integrity, with small amounts of decomposition products detected by HPLC, LC-MS and MALDI-TOF-MS measurements. Overall, the bioinorganic reagent was shown to be best suited for single-stranded RNA motifs, whereas m5C incorporated in small loops or stem positions of short RNAs exhibited only minimal reactivity towards the complex. The chemically driven oxidation enabled by our iron complex provides a stable, sequence-independent approach and thus represents a promising strategy toward standardized, broadly applicable protocols for research and diagnostic applications. These findings are complemented by the development of a quantitative UHPLC-MS/MS (QQQ) workflow using isotopically labelled cytidine internal standards, providing absolute quantification of m5C and its three oxidation products.

## Conflicts of interest

There are no conflicts to declare.

## Supplementary Material

CB-OLF-D6CB00203J-s001

## Data Availability

Data supporting the conclusions of this manuscript are included in the supplementary information (SI). Raw data has been deposited on RADAR4chem, see DOI: https://radar4chem.radar-service.eu/radar/en/dataset/z7ufn2cy2714vurn. Supplementary information is available. See DOI: https://doi.org/10.1039/d6cb00203j.
