# Supplementary material for: Selective oxidation and quantification of m5C in ribonucleosides and RNA in an epigenetic context
Source: RSC Chem Biol. 2026 Aug 21;7(10):2080–90. doi: 10.1039/d6cb00203j (PMC13494956; doi:10.1039/d6cb00203j)
Supplement: Supplementary file 1 [file CB-007-D6CB00203J-s001.pdf]

Supplementary Information (SI) for RSC Chemical Biology.
This journal is © The Royal Society of Chemistry 2026

Supporting Information for

Selective Oxidation and Quantification of m5C in Ribonucleosides and RNA in an Epigenetic Context

Kora Holschbach*[,1], Tobias Walter*[,2], Davide Guglielminotti[2], Annika Menke[3], David Schmidl[3], Doreen Reuter[1], Ken Kögel[2], Lola Stroh[2], Sabine Schneider[2], Pascal Giehr[2], Thomas Carell[2,c], Lena Daumann[1,c]

[1]Lehrstuhl für Bioanorganische Chemie, Heinrich-Heine-Universität Düsseldorf, Universitätsstraße 1, 40225 Düsseldorf (Germany)

[2]Center for Nucleic Acid Therapeutics at the Department of Chemistry, Institute for Chemical Epigenetics, Ludwig-Maximilians-Universität München, Butenandtstr. 5-13, 81377 München, Germany

[3]Department of Chemistry, Ludwig-Maximilians-University München, Butenandtstr. 5–13, 81377 München Germany

*Both authors contributed equally to this work.

[C]Corresponding authors lena.daumann@hhu.de, thomas.carell@lmu.de

Table of Contents

Synthesis of ribonucleosides including isotopically labelled internal standards 2

Synthesis of m5C, hm5C, f5C and ca5C Ribonucleosides 2

Synthesis of isotopically labelled ribonucleosides 16

Analytical Methods 29

HPLC 29

MALDI-TOF-MS for Qualitative Oligonucleotide Analysis 32

UHPLC-MS/MS (QQQ) for Nucleoside-Quantification 35

Materials and Methods 39

General Considerations 39

Preparation of $[Fe^{IV}\mathbf{L_x}(O)]^{2+}$ (1 mM) with L1 or L2 ligand system 39

RNA Oligomers 40

Enzymatic RNA Synthesis 40

In vitro transcription of RNA substrates 41

Sample preparation and reaction for analysis by mass spectrometry 42

Sample digestion and preparation for UHPLC-MS/MS (QQQ) analysis 42

Complex decomposition with formamide 43

NMR Spectroscopy 44

HR-ESI-MS 68

Supporting References 90

Synthesis of ribonucleosides including isotopically labelled internal standards

Synthesis of m5C, hm5C, f5C and ca5C Ribonucleosides

*O*2',3',5'-Tris(*tert*-butyldimethylsilyl)-cytidine (1)

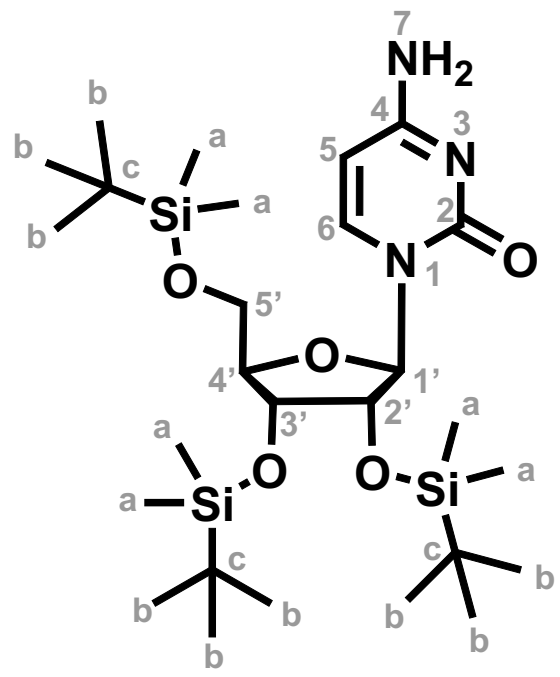

1

According to a modified literature procedure.[1]

Cytidine rC (300 mg, 1.23 mmol, 1.0 equiv.) and imidazole (670 mg, 9.84 mol, 8.0 equiv.) were dissolved in anhydrous *N*,*N*-dimethylformamide (2.5 mL) and the solution was cooled to 0 °C. *tert*-Butyldimethylsilyl chloride (837 mg, 5.55 mmol, 4.5 equiv.) was added in two portions and the reaction mixture was allowed to slowly warm to 21 °C. After 20 h, the reaction mixture was diluted with ethyl acetate (20 mL) and saturated aqueous sodium bicarbonate solution (20 mL) was added slowly. The layers were separated and the aqueous layer was extracted with ethyl acetate (3×20 mL). The combined organic layers were washed with saturated aqueous sodium chloride solution (25 mL) and dried over sodium sulfate. The dried solution was filtered and the filtrate was concentrated *in vacuo*. The resulting colorless oil was purified by flash column chromatography (5% methanol in dichloromethane) to afford silyl-protected cytidine **1** (640 mg, 1.09 mmol, 89%) as a foaming colorless solid.

The obtained analytical data were in full agreement with those reported in the literature.[2]

HR MS (ESI+): *m/z* calcd for $(C_{27}H_{56}N_3O_5Si_3)^+$ [M+H]+: 586.3522, found: 586.3536.

^{1}**H-NMR** (400 MHz, $CDCl_3$, 295 K): δ (ppm) = 8.18 (d, J = 7.4 Hz, 1H-6), 5.78 (d, J = 1.8 Hz, 1H, H-1’), 5.66 (s, 2H, H-7), 5.58 (d, J = 7.4 Hz, 1H, H-5), 4.12 – 4.00 (m, 4H, H-2’, H-3’, H-4’, H-5’a), 3.78 (dd, J = 11.6, 1.4 Hz, 1H, H-5’b), 0.95 (s, 9H, H-b), 0.90 (s, 9H, H-b), 0.88 (s, 9H, H-b), 0.22 (s, 3H, H-a), 0.13 (s, 3H, H-a), 0.11 (s, 3H, H-a), 0.11 (s, 3H, H-a), 0.05 (s, 3H, H-a), 0.04 (s, 3H, H-a).

^{13}**C-{^{1}H}-NMR** (101 MHz, $CDCl_3$, 295 K): δ (ppm) = 165.6 (C-4), 155.9 (C-2), 142.2 (C-6), 93.1 (C-5), 90.5 (C-1’), 83.0 (C-4’), 76.4 (C-2’), 69.4 (C-3’), 61.1 (C-5’), 26.3 (C-b), 26.0 (C-b), 26.0 (C-b), 18.7 (C-c), 18.2 (C-c), 18.2 (C-c), -3.9 (C-a), -4.0 (C-a), -4.9 (C-a), -5.0 (C-a), -5.1 (C-a), -5.4 (C-a).

***O*2',3',5'-Tris(*tert*-butyldimethylsilyl)-5-iodo-cytidine (2)**

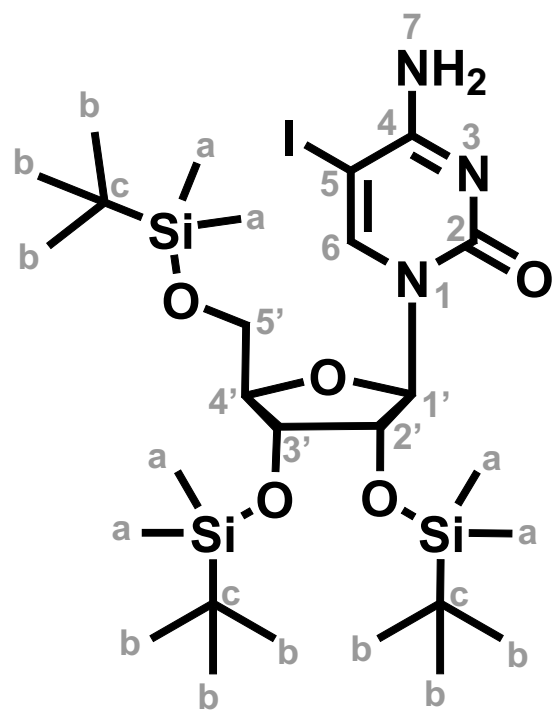

2

According to a modified literature procedure.[3]

Silyl-protected cytidine **1** (1.36 g, 2.32 mmol, 1.0 equiv.) was dissolved in anhydrous acetonitrile (110 mL) followed by the addition of iodine (1.30 g, 5.11 mmol, 2.2 equiv.) and ceric ammonium nitrate (2.80 g, 5.11 mmol, 2.2 equiv.). The resulting dark red reaction mixture was heated to 60 °C under exclusion of light for 2 h. The reaction was allowed to cool to 21 °C and the brown suspension was slowly poured into a mixture of saturated aqueous sodium bicarbonate solution (150 mL) and aqueous sodium thiosulfate solution (1.0 M, 100 mL). Acetonitrile was evaporated from the resulting slightly yellow suspension and ethyl acetate (150 mL) was added. After the separation of the layers, the aqueous layer was extracted with ethyl acetate (3×80 mL). All organic layers were washed with saturated aqueous sodium chloride solution (80 mL) and then dried over magnesium sulfate. The filtrate obtained after filtration was concentrated *in*

vacuo. Purification of the obtained yellow solid followed by flash column chromatography (2% to 4% to 5% methanol in dichloromethane) to afford silyl-protected 5-iodocytidine **2** (1.20 g, 1.69 mmol, 72%) as a colorless solid.

HR MS (ESI+): *m/z* calcd for $(C_{27}H_{55}IN_3O_5Si_3)^+$ $[M+H]^+$: 712.2489, found: 712.2498.

^{1}H-NMR (400 MHz, $CDCl_3$, 295 K): δ (ppm) = 8.06 (s, 1H, H-6), 6.81 (s, 1H, H-7a), 5.97 (d, *J* = 4.5Hz, 1H, H-1'), 5.50 (s, 1H, H-7b), 4.16 – 4.05 (m, 2H, H-2', H-4'), 4.03 – 3.94 (m, 2H, H-3', H-5'a), 3.76 (dd, *J* = 11.6, 2.3 Hz, 1H, H-5'b), 0.99 (s, 9H, H-b), 0.90 (s, 9H, H-b), 0.88 (s, 9H, H-b), 0.19 (s, 3H, H-a), 0.19 (s, 3H, H-a), 0.07 (s, 3H, H-a), 0.06 (s, 3H, H-a), 0.06 (s, 3H, H-a), 0.05 (s, 3H, H-a).

^{13}C-{^{1}H}-NMR (101 MHz, $CDCl_3$, 295 K): δ (ppm) = 163.3 (C-4), 154.8 (C-2), 147.2 (C-6), 89.3 (C-1'), 84.9 (C-4'), 77.3 (C-5), 76.4 (C-2'), 71.4 (C-3'), 62.6 (C-5'), 26.6 (C-b), 26.0 (C-b), 26.0 (C-b), 18.9 (C-c), 18.2 (C-c), 18.1 (C-c), −4.1 (C-a), −4.3 (C-a), −4.5 (C-a), −4.6 (C-a), −4.6 (C-a), −4.9 (C-a).

Synthesis of f5C

***O*2',3',5'-Tris(*tert*-butyldimethylsilyl)-5-formyl-cytidine (3)**

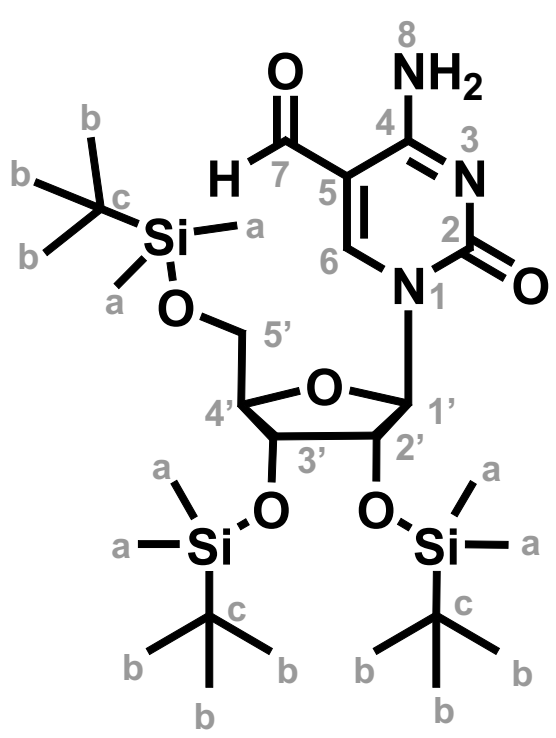

3

According to a modified literature procedure.[3]

In a high-pressure autoclave, 5-iodocytidine **2** (500 mg, 0.702 mmol, 1.0 equiv.), $[Pd_2(dba)_3]\cdot CHCl_3$ (72.7 mg, 0.070 mmol, 0.1 equiv.) as well as triphenylphosphine (110 mg, 0.421 mmol, 0.6 equiv.) were dissolved in anhydrous toluene (21 mL) under argon atmosphere. After flushing the autoclave with carbon monoxide gas (3.5 bar)

while stirring the reaction solution, pressure was released when 10 min had passed. Achieving carbon monoxide saturation of the reaction solution was done by flushing the autoclave two more times with carbon monoxide. The autoclave was then saturated with carbon monoxide gas (3.5 bar) again and the reaction was started by heating the mixture to 60 °C. Over a period of 16 h, a dropwise addition of a solution of tributyltin hydride (0.3 mL, 0.842 mmol, 1.2 equiv.) in anhydrous toluene (0.3 mL) *via* syringe (0.060 mL per hour) occurred. After 17 h from the start of the tributyltin hydride addition, the reaction was allowed to cool to 21 °C and the pressure was released. The obtained greenish black suspension was diluted with ethyl acetate (10 mL) and filtered through a plug of silica. The filter cake was extensively washed with ethyl acetate (400 mL) and the filtrate was concentrated *in vacuo*. An orange solid was obtained and purified by flash column chromatography (25% to 50% ethyl acetate in hexanes) to afford silyl-protected 5-formylcytidine **3** (410 mg, 0.668 mmol, 95%) as a colorless solid.

HR MS (ESI+): *m/z* calcd for $(C_{28}H_{56}N_3O_6Si_3)^+$ $[M+H]^+$: 614.3471, found: 614.3479.

^{1}H-NMR (400 MHz, $CDCl_3$, 295 K): δ (ppm) = 9.52 (s, 1H, H-7), 8.62 (s, 1H, H-6), 8.16 (s, 1H, H-8a), 5.94 (s, 1H, H-8b), 5.87 (d, J = 2.5 Hz, 1H, H-1’), 4.20 (dd, J = 4.1, 2.5 Hz, 1H, H-2’), 4.17 (dt, J = 6.8, 2.1 Hz, 1H, H-4’), 4.15 – 4.08 (m, 1H, H-5’a), 3.98 (dd, J = 6.7, 4.1 Hz, 1H, H-3’), 3.82 (dd, J = 11.9, 2.0 Hz, 1H, H-5’b), 0.97 (s, 9H, H-b), 0.91 (s, 9H, H-b), 0.90 (s, 9H, H-b), 0.17 (s, 3H, H-a), 0.17 (s, 3H, H-a), 0.13 (s, 3H, H-a), 0.11 (s, 3H, H-a), 0.07 (s, 3H, H-a), 0.06 (s, 3H, H-a).

^{13}C-{^{1}H}-NMR (101 MHz, $CDCl_3$, 295 K): δ (ppm) = 187.6 (C-7), 162.9 (C-4), 153.9 (C-2), 153.7 (C-6), 105.8 (C-5), 91.1 (C-1’), 84.3 (C-4’), 76.9 (C-2’), 70.2 (C-3’), 62.0 (C-5’), 26.8 (C-b), 26.3 (C-b), 26.3 (C-b), 19.3 (C-c), 18.5 (C-c), 18.5 (C-c), −3.5 (C-a), −3.7 (C-a), −4.1 (C-a), −4.5 (C-a), −4.6 (C-a), −4.9 (C-a).

5-Formyl-cytidine (f5C)

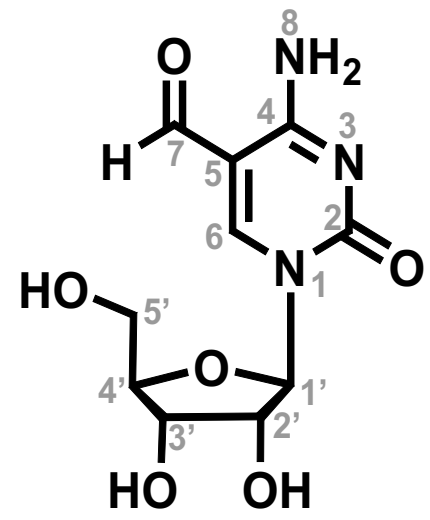

f5C

According to a modified literature procedure.[5]

Protected 5-formylcytidine **3** (470 mg, 0.765 mmol, 1.0 equiv.) was dissolved in anhydrous ethyl acetate (23 mL) in a plastic tube open to air and hydrogen fluoride pyridine complex (70wt% HF, 0.30 mL, 12 mmol, 15 equiv.) was added slowly via syringe. The yellow solution was then stirred at 21 °C for 19 h. The resulting suspension was cooled to 0 °C, methoxytrimethylsilane (3.4 mL, 25 mmol, 32 equiv.) was added via syringe and the reaction was allowed to slowly warm to 21 °C. After 5 h, the reaction mixture was diluted with methanol (30 mL) and all volatiles were removed *in vacuo* to yield 400 mg of an orange solid. The crude product was purified by HPLC (Column: Agilent Prep-C18 column 50×10.0 mm; 5 µm; 2% MeCN in H_2O for 7 min) to afford 5-formylcytidine (73.0 mg, 0.269 mmol, 35%) as a colorless solid.

HR MS (ESI+): *m/z* calcd for $(C_{10}H_{14}N_3O_6)^+$ $[M+H]^+$: 272.0877, found: 272.0874.

^{1}H-NMR (400 MHz, D_2O, 295 K): δ (ppm) = 9.50 (s, 1H, H-7), 8.92 (s, 1H, H-6), 5.89 (d, *J* = 2.0Hz, 1H, H-1’), 4.34 (dd, *J* = 4.4, 2.1Hz, 1H, H-2’), 4.26 – 4.17 (m, 2H, H-3’/H-4’), 4.07 (dd, *J* = 13.1, 2.3 Hz, 1H, H-5’a), 3.88 (dd, *J* = 13.1, 3.1 Hz, 1H, H-5’b).

^{13}C-{^{1}H}-NMR (101 MHz, D_2O, 295 K): δ (ppm) = 190.2 (C-7), 162.6 (C-4), 155.2 (C-2), 154.7 (C-6), 105.8 (C-5), 91.4 (C-1’), 83.4 (C-4’), 74.5 (C-2’), 68.0 (C-3’), 59.6 (C-5’).

Synthesis of ca5C

*O*2',3',5'-Tris(*tert*-butyldimethylsilyl)-5-(methoxycarbonyl)-cytidine (4)

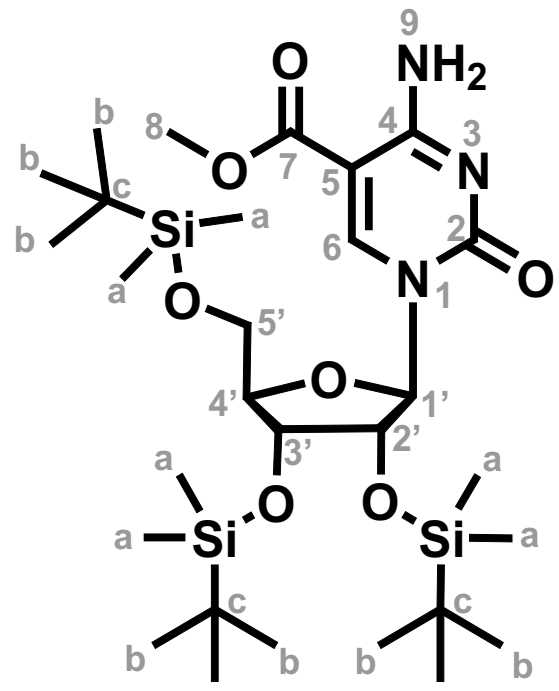

4

According to a modified literature procedure.[4]

The following reaction was carried out in an high-pressure autoclave under argon atmosphere. Silyl-protected iodocytidine **2** (500 mg, 0.702 mmol, 1.0 equiv.) and bis(acetonitrile)palladium dichloride (9.11 mg, 35.12 µmol, 0.05 equiv.) were dissolved in anhydrous methanol (8.33 mL) under argon atmosphere in the autoclave. Flushing of the autoclave with carbon monoxide gas (3.5 bar) occurred while stirring the reaction solution. After 10 min the pressure was released. After achieving carbon monoxide saturation of the reaction solution by repeating the flushing procedure two more times, the autoclave was filled with carbon monoxide gas (3.5 bar) and the reaction mixture was heated to 60 °C. The addition of a solution of *N*,*N*-di-*iso*-propylethylamine (244.68 µL, 1.40 mmol, 2.0 equiv.) in anhydrous methanol (282 L) followed *via* syringe in one portion. The reaction was allowed to cool to 21 °C after 19 h and the pressure was released. The reaction was transferred to a 50 mL flask and the autoclave was washed with ethyl acetate (10 mL). The orange-black suspension was diluted with ethyl acetate and filtered through a plug of silica. The filter cake was washed with ethyl acetate (ca. 1000 mL) repeatedly and the filtrate was concentrated *in vacuo*. The resulting orange solid was purified by flash column chromatography (0% to 2% to 5% methanol in dichloromethane) to afford silyl-protected 5-methoxycarbonylcytidine **4** (363.5 mg, 0.564 mmol, 80%) as a light yellow powder.

HR MS (ESI+): *m/z* calcd for $(C_{29}H_{58}N_3O_7Si_3)^+$ $[M+H]^+$: 644.3577, found: 644.3581.

^{1}H-NMR (400 MHz, $CDCl_3$, 295 K): δ (ppm) = 8.50 (s, 1H, H-6), 7.94 (d, J = 4.4 Hz, 1H, H-9a), 5.96 (d, J = 4.7 Hz, 1H, H-1'), 5.89 (s, 1H, H-9b), 4.23 (t, J = 4.5 Hz, 1H, H-2'), 4.12 (m, 1H, H-4'), 3.98 – 3.90 (m, 2H, H-3', H-5'a), 3.84 (s, 3H, H-8), 3.76 (dd, J = 11.5, 4.0 Hz, 1H, H-5'b), 0.95 (s, 9H, H-b), 0.90 (s, 9H, H-b), 0.88 (s, 9H, H-b), 0.14 (s, 3H, H-a), 0.13 (s, 3H, H-a), 0.07 (s, 3H, H-a), 0.06 (s, 3H, H-a), 0.05 (s, 3H, H-a), 0.04 (s, 3H, H-a).

^{13}C-{^{1}H}-NMR (101 MHz, $CDCl_3$, 295 K): δ (ppm) = 165.4 (C-7), 163.1 (C-4), 153.9 (C-2), 148.2 (C-6), 95.7 (C-5), 90.2 (C-1'), 85.3 (C-4'), 76.1 (C-2'), 72.0 (C-3'), 63.4 (C-5'), 52.2 (C-8), 26.2 (C-b), 26.0 (C-b), 26.0 (C-b), 18.2 (C-c), 18.1 (C-c), 18.1 (C-c), −4.1 (C-a), −4.3 (C-a), −4.6 (C-a), −4.6 (C-a), −5.1 (C-a), −5.3(C-a).

5-Carboxyl-cytidine (ca5C)

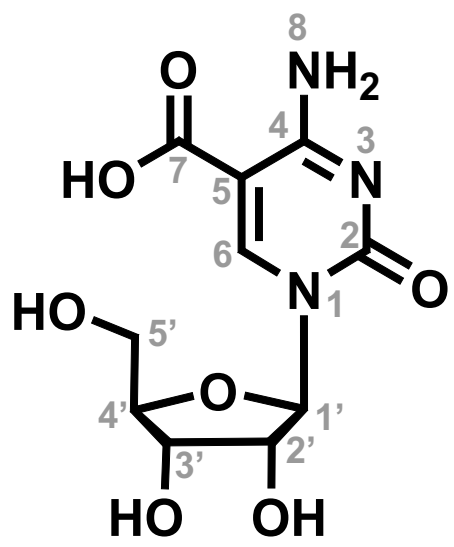

ca5C

According to a modified literature procedure.[5]

In a plastic tube open to air silyl-protected methyl ester **4** (152 mg, 0.236 mmol, 1.0 equiv.) was suspended in anhydrous ethyl acetate (7.1 mL) and hydrogen fluoride pyridine complex (70 wt% HF, 64 µL, 2.5 mmol, 11 equiv.) was added slowly *via* syringe resulting in a clear solution. After 17 h, the reaction was cooled to 0 °C and careful addition of methoxytrimethylsilane (1.1 mL, 7.6 mmol, 32 equiv.) followed *via* syringe. The reaction was allowed to slowly warm to 21 °C. After 5 h, the suspension was diluted with methanol (15 mL) and all volatiles were removed *in vacuo*. Complete conversion was expected to continue the reaction. The resulting yellowish solid was dissolved in a mixture of water and acetonitrile (1:1, 100 mL) open to air, following the addition of lithium hydroxide (67.8 mg, 2.83 mmol, 12 equiv.). After 16 h, the solution was brought to pH 3 by dropwise addition of 5.0 M hydrochloric acid and all volatiles were removed *in vacuo*. The resulting tan solid was purified by HPLC (Column: Agilent

Prep-C18 column 50×10.0 mm; 5 µm; 0% MeCN in H_2O (both containing 0.1% trifluoroacetic acid) for 2 min, then 2% for 3.5 min) to afford 5-carboxycytidine ca5C (21.4 mg, 74.5 µmol, 32% over two steps) as a colorless solid.

HR MS (ESI+): *m/z* calcd for $(C_{10}H_{14}N_3O_7)^+$ $[M+H]^+$: 288.0826, found: 288.0829.

^{1}H-NMR (400 MHz, DMSO-d$_6$, 295 K): δ (ppm) = 8.95 (s, 1H, H-6), 5.76 (d, *J* = 3.2 Hz, 1H, H-1’), 4.00 (dd, *J* = 4.6, 3.3 Hz, 1H, H-2’), 3.97 – 3.86 (m, 2H, H-3’, H-4’), 3.71 (dd, *J* = 12.0, 2.6 Hz, 1H, H-5’a), 3.56 (dd, *J* = 12.1, 2.8 Hz, 1H, H-5’b).

^{13}C-{^{1}H}-NMR (101 MHz, DMSO-d$_6$, 295 K): δ (ppm) = 166.4 (C-7), 163.0 (C-4), 152.9 (C-2), 149.1 (C-6), 95.8 (C-5), 90.1 (C-1’), 84.2 (C-4’), 74.5 (C-2’), 68.8 (C-3’), 60.2 (C-5’).

Synthesis of hm5C

***O*2',3',5'-Tris(*tert*-butyldimethylsilyl)-5-hydroxymethyl-cytidine (5)**

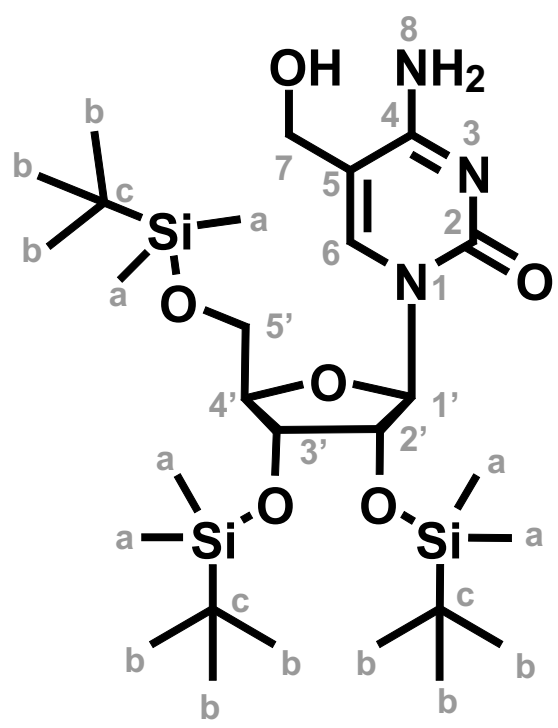

5

According to a modified literature procedure.[1]

Silyl-protected 5-formyl-cytidine **3** (185 mg, 0.301 mmol, 1.0 equiv.) and cerium trichloride heptahydrate (336 mg, 0.903 mmol, 3.0 equiv.) were suspended in HPLC grade methanol (15 mL) followed by the addition of sodium borohydride (11.4 mg, 0.301 mmol, 1.0 equiv.). The dark brown suspension was stirred at 21 °C for 30 min and saturated aqueous ammonium chloride solution (20 mL) was added slowly. After further addition of water (20 mL), the emulsion was stirred for 10 min. Methanol was evaporated, the resulting suspension was filtered through a plug of celite and the filter

cake was washed with ethyl acetate (50 mL). The layers were separated and the aqueous layer was extracted with ethyl acetate (3×30 mL). The combined organic layers were washed with saturated aqueous sodium chloride solution (50 mL) and dried over sodium sulfate. The dried solution was filtered and the filtrate was concentrated *in vacuo*. The resulting slightly brown solid was purified by flash column chromatography (2% to 5% to 9% methanol plus 1% triethylamine in dichloromethane) to afford 149 mg of a colorless powder.

^{1}H-NMR (400 MHz, $CDCl_3$, 295 K): δ (ppm) = 7.66 (s, 1H; H-6), 7.00 (s, 2H; H-8), 5.82 (d, J = 2.9 Hz, 1H; H-1'), 4.49 – 4.37 (m, 2H; H-5'), 4.13 – 3.92 (m, 3H), 3.97 – 3.63 (m, 1H), 3.06 (q, J = 7.3 Hz, 2H), 0.95 (s, 9H, H-b), 0.89 (s, 9H, H-b), 0.87 (s, 9H, H-b), 0.13 (s, 3H, H-a), 0.12 (s, 3H, H-a), 0.06 (s, 3H, H-a), 0.06 (s, 3H, H-a), 0.05 (s, 3H, H-a), 0.04 (s, 3H, H-a).

^{13}C-{^{1}H}-NMR (101 MHz, $CDCl_3$, 295 K): δ 164.2 (C-4), 156.1 (C-2), 141.6 (C-6), 106.1 (C-5), 89.8 (C-1') 84.4 (C-4'), 76.3 (C-2'), 71.0 (C-3'), 58.6 (C-5'), 46.0 (C-7), 26.4 (C-b), 26.0 (C-b), 26.0 (C-b), 18.8 (C-c), 18.2 (C-c), 18.2 (C-c), -4.0 (C-a), -4.2 (C-a), -4.7 (C-a), -4.8 (C-a), -4.9 (C-a), -5.2 (C-a).

5-Hydroxymethyl-cytidine (hm5C)

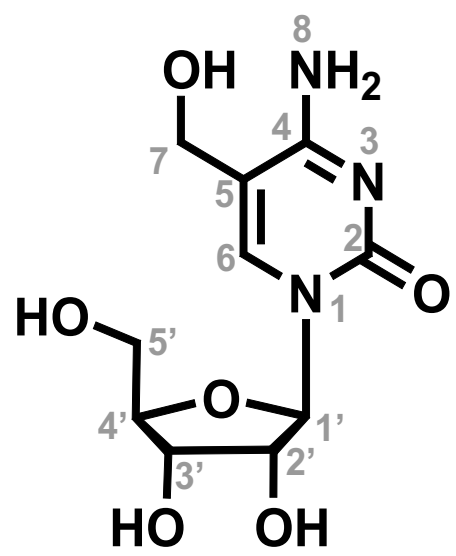

hm5C

According to a modified literature procedure.[1]

Silyl-protected 5-hydroxymethyl-cytidine **5** (1.0 equiv.) was dissolved in anhydrous ethyl acetate (6.7 mL) in a plastic tube open to air and hydrogen fluoride pyridine complex (70wt% HF, 0.17 mL, 6.7 mmol, 30 equiv.) was added *via* syringe. After stirring the reaction solution at 21 °C for 16 h, the mixture was cooled to 0 °C and methoxytrimethylsilane (2.0 mL, 14 mmol, 64 equiv.) was added slowly *via* syringe. The reaction

was allowed to slowly warm to 21 °C and after 20 h, the suspension was diluted with methanol (15 mL). All volatiles were removed *in vacuo* to yield 83.9 mg of a colorless solid. The crude product was purified by HPLC (Column: Agilent Prep-C18 column 50×10.0 mm; 5 µm; 0% MeCN in H_2O for 5 min) to afford 5-hydroxymethylcytidine hm5C (21.8 mg, 79.8 µmol, 27% over two steps) as a colorless solid.

HR MS (ESI+): *m/z* calcd for $(C_{10}H_{16}N_3O_6)^+$ $[M+H]^+$: 274.1034, found: 274.1032.

^{1}H-NMR (400 MHz, D_2O, 295 K): δ (ppm) = 7.98 (s, 1H, H-6), 5.90 (d, *J* = 3.7 Hz, 1H, H-1'), 4.45 (s, 2H, H-7), 4.31 (dd, *J* = 5.2, 3.7 Hz, 1H, H-2'), 4.22 (dd, *J* = 6.3, 5.2 Hz, 1H, H-3'), 4.14 (ddd, *J* = 6.5, 4.0, 2.8 Hz, 1H, H-4'), 3.96 (dd, *J* = 12.9, 2.8 Hz, 1H, H-5'a), 3.83 (dd, *J* = 12.9, 4.0 Hz, 1H, H-5'b).

^{13}C-{^{1}H}-NMR (101 MHz, D_2O, 295 K): δ (ppm) = 164.4 (C-4), 156.5 (C-2), 140.7 (C-6), 106.6 (C-5), 90.3 (C-1'), 83.7 (C-4'), 74.0 (C-2'), 68.9 (C-3'), 60.4 (C-5'), 57.7 (C-7).

Synthesis of m5C

***O*2',3',5'-Tris(*tert*-butyldimethylsilyl)-5-methyl-uridine (7)**

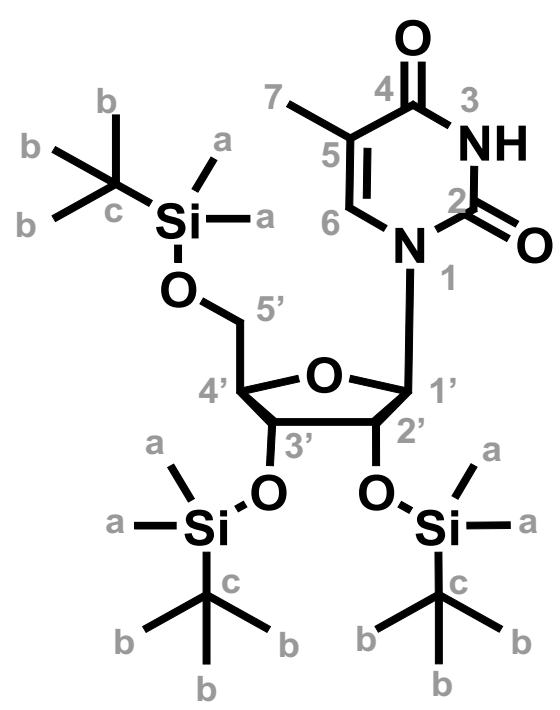

7

According to a modified literature procedure.[6]

Both 5-methyluridine **6** (500 mg, 1.94 mmol, 1.0 equiv.) and imidazole (15.5 mmol, 1.06 g, 8.0 equiv.) were dissolved in anhydrous *N*,*N*-dimethylformamide (4.0 mL) and the solution was cooled to 0 °C. *tert*-Butyldimethylsilyl chloride (1.31 g, 8.71 mmol, 4.5 equiv.) was added in two portions and the reaction mixture was allowed to slowly

warm to 21 °C. After stirring for 14 h, the reaction mixture was diluted with ethyl acetate (20 mL), saturated aqueous sodium bicarbonate solution (20 mL) and water (10 mL) were added. The layers were separated and the aqueous layer was extracted with ethyl acetate (3×20 mL). The combined organic layers were washed with saturated aqueous sodium chloride solution (20 mL) and dried over sodium sulfate. The dried solution was filtered and the filtrate was concentrated *in vacuo*. The resulting slightly yellow viscous oil was purified by flash column chromatography (20% ethyl acetate in hexanes) to afford a mixture of silyl-protected 5-methyluridine **6** (92wt%, 1.13 g, 1.88 mmol, 97%) with *tert*-butyldimethylsilanol as a sticky colorless solid.

HR MS (ESI+): *m/z* calcd for $(C_{28}H_{57}N_2O_6Si_3)^+$ $[M+H]^+$: 601.3519, found: 601.3512.

^{1}H-NMR (400 MHz, $CDCl_3$, 295 K): δ (ppm) = 7.96 (s, 1H, H-3), 7.47 (q, J = 1.2 Hz, 1H, H-6), 6.03 (d, J = 6.6 Hz, 1H, H-1'), 4.16 – 4.09 (m, 1H, H-2'), 4.06 (dd, J = 4.7, 2.2 Hz, 1H, H-3'), 4.02 (q, J = 2.2 Hz, 1H, H-4'), 3.89 (dd, J = 11.6, 2.3 Hz, 1H, H-5'a), 3.74 (dd, J = 11.6, 2.1 Hz, 1H, H-5'b), 1.93 (d, J = 1.2 Hz, 3H, H-7), 0.97 (s, 9H, H-b), 0.91 (s, 9H, H-b), 0.86 (s, 9H, H-b), 0.15 (s, 3H, H-a), 0.14 (s, 3H, H-a), 0.09 (s, 3H, H-a), 0.07 (s, 3H, H-a), 0.02 (s, 3H, H-a), −0.06 (s, 3H, H-a).

^{13}C-{^{1}H}-NMR (101 MHz, $CDCl_3$, 295 K): δ (ppm) = 163.5 (C-4), 150.4 (C-2), 136.0 (C-6), 111.0 (C-5), 87.4 (C-1'), 86.2 (C-4'), 75.4 (C-2'), 72.6 (C-3'), 63.3 (C-5'), 26.2 (C-b), 26.0 (C-b), 25.8 (C-b), 18.7 (C-c), 18.2 (C-c), 18.1 (C-c), 12.5 (C-7), −4.3 (C-a), −4.3 (C-a), −4.5 (C-a), −4.7 (C-a), −5.2 (C-a), −5.2 (C-a).

O2',3',5'-Tris(*tert*-butyldimethylsilyl)-O4-(2,4,6-Tri(iso-propyl)phenylsulfonyl)-5-methyl-cytidine (8)

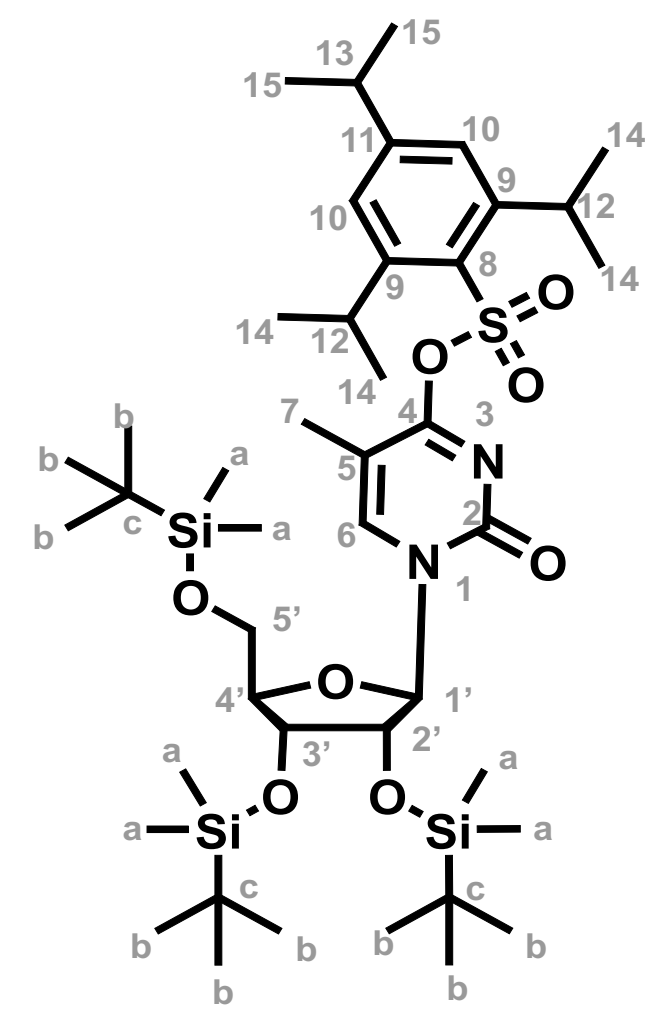

8

According to a modified literature procedure.[6]

Silyl-protected 5-methyluridine **7** (97wt%, 870 mg, 1.33 mmol, 1.0 equiv.) was dissolved in anhydrous tetrahydrofuran (5.3 mL) and the solution was cooled to 0 °C. A suspension of sodium hydride in mineral oil (60wt%, 266 mg, 6.66 mmol, 5.0 equiv.) was added upon which gas evolution was observed. After stirring at 0 °C for 45 min, 2,4,6-tri-(*iso*-propyl)phenylsulfonyl chloride (806 mg, 2.66 mmol, 2.0 equiv.) was added and the reaction was allowed to slowly warm to 21 °C. After stirring for 15 h, the reaction mixture had become solid. It was diluted with ethyl acetate (30 mL) and saturated aqueous ammonium chloride solution (50 mL) was added slowly. The layers were separated and the aqueous layer was extracted with ethyl acetate (4×20 mL). The combined organic layers were washed with saturated aqueous sodium chloride solution (20 mL) and dried over sodium sulfate. The dried solution was filtered and the filtrate was concentrated *in vacuo*. The resulting colorless solid was purified by flash column chromatography (0% to 10% ethyl acetate in hexanes) to afford sulfonic acid ester **8** (646 mg, 0.745 mmol, 56%) as a colorless powder.

HR MS (ESI+): *m/z* calcd for $(C_{43}H_{79}N_2O_8SSi_3)^+$ $[M+H]^+$: 867.4859, found: 867.4866.

^{1}H-NMR (400 MHz, $CDCl_3$, 295 K): δ (ppm) = 7.96 (q, J = 1.1 Hz, 1H, H-6), 7.18 (s, 2H, H-10), 5.88 (d, J = 4.1 Hz, 1H, H-1'), 4.30 (h, J = 6.7 Hz, 2H, H-12), 4.09 – 4.06 (m, 1H, H-4'), 4.05 (dd, J = 4.3, 4.3 Hz, 1H, H-2'), 3.98 (dd, J = 11.8, 2.3 Hz, 1H, H-

5'a), 3.96 (t, J = 4.8 Hz, 1H, H-3'), 3.75 (dd, J = 11.7, 2.0 Hz, 1H, H-5'b), 2.88 (h, J = 6.9 Hz, 1H, H-13), 2.03 (d, J = 1.0 Hz, 3H, H-7), 1.32 – 1.21 (m, 18H; H-14/H-15), 0.95 (s, 9H, H-b), 0.88 (s, 9H, H-b), 0.80 (s, 9H, H-b), 0.45 (s, 3H, H-a), 0.12 (s, 3H, H-a), 0.06 (s, 3H, H-a), 0.04 (s, 3H, H-a), −0.03 (s, 3H, H-a), −0.09 (s, 3H, H-a).

^{13}C-{^{1}H}-NMR (101 MHz, $CDCl_3$, 295 K): δ (ppm) = 166.2 (C-4), 154.3 (C-1), 153.8 (C-2), 151.3 (C-9), 143.3 (C-6), 131.0 (C-8), 124.1 (C-10), 103.6 (C-5), 89.8 (C-1'), 85.0 (C-4'), 76.6 (C-2'), 71.1 (C-3'), 62.3 (C-5'), 34.3 (C-13), 29.7 (C-12), 26.3 (C-b), 26.0 (C-b), 25.9 (C-b), 24.6 (C-14a), 24.5 (C-14b), 23.6 (C-15), 18.8 (C-c), 18.2 (C-c), 18.1 (C-c), 12.4(C-7), −4.1 (C-a), −4.6 (C-a), −4.8 (C-a), −4.8 (C-a), −4.9 (C-a), −5.3 (C-a).

5-methylcytidine (m5C)

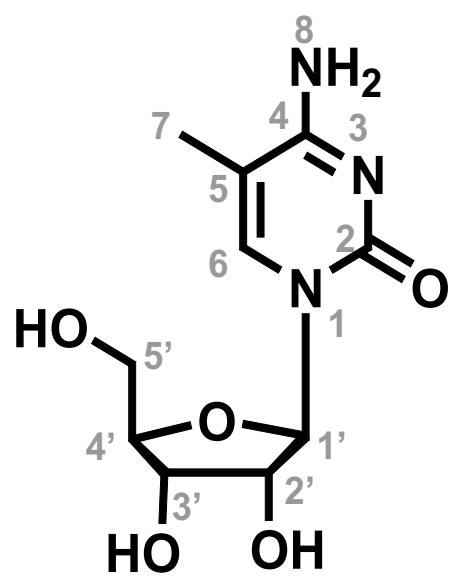

m5C

According to a modified literature procedure.[6]

Amination: Silyl-protected sulfonic acid ester **8** (461 mg, 0.531 mmol, 1.0 equiv.) was dissolved in anhydrous 1,4-dioxane (11 mL) and a solution of ammonia in anhydrous methanol (7.0 M, 7.6 mL, 10 equiv.) was added dropwise *via* syringe at 21 °C. After 6.5 h, ice and saturated aqueous ammonium chloride solution (50 mL) were added and the reaction mixture was diluted with ethyl acetate (20 mL). The layers were separated and the aqueous layer was extracted with ethyl acetate (3×50 mL). The combined organic layers were washed with saturated aqueous sodium chloride solution (50 mL) and dried over sodium sulfate. The dried solution was filtered and the filtrate was concentrated *in vacuo*. The resulting colorless solid was purified by flash column chromatography (2% to 5% methanol in dichloromethane) to give a colorless solid (275 mg).

Deprotection: The colorless solid (1.0 equiv.) was dissolved in anhydrous ethyl acetate (14 mL) in a plastic tube open to air and hydrogen fluoride pyridine complex

(70wt% HF, 0.36 mL, 14 mmol, 30 equiv.) was added *via* syringe. The reaction mixture was stirred at 21 °C for 16 h. The resulting suspension was cooled to 0 °C and methoxytrimethylsilane (4.0 mL, 29 mmol, 64 equiv.) was added slowly *via* syringe. After 30 min, the mixture was allowed to warm to 21 °C and was stirred for another 1 h. The reaction suspension was diluted with methanol (15 mL) and all volatiles were removed *in vacuo*. The resulting colorless solid was purified by HPLC (Column: Agilent Prep-C18 column 50×10.0mm; 5 µm; 0% MeCN in H_2O for 8 min) to afford 5-methylcytidine m5C (42.1 mg, 0.164 mmol, 31% over two steps) as a colorless solid.

HR MS (ESI+): *m/z* calcd for $(C_{10}H_{16}N_3O_5)^+$ $[M+H]^+$: 258.1084, found: 258.1082.

^{1}H-NMR (400 MHz, D_2O, 295 K): δ (ppm) = 7.69 (q, *J* = 1.1 Hz, 1H, H-6), 5.91 (d, *J* = 4.2 Hz, 1H, H-1’), 4.30 (dd, *J* = 5.4, 4.2 Hz, 1H, H-2’), 4.22 (dd, *J* = 5.7, 5.2 Hz, 1H, H-3’), 4.12 (ddd, *J* = 5.9, 4.2, 2.9 Hz, 1H, H-4’), 3.93 (dd, *J* = 12.8, 2.9 Hz, 1H, H-5’a), 3.82 (dd, *J* = 12.8, 4.3 Hz, 1H, H-5’b), 1.97 (d, *J* = 1.1 Hz, 3H, H-7).

^{13}C-{^{1}H}-NMR (101 MHz, D_2O, 295 K): δ (ppm) = 166.0 (C-4), 157.5 (C-2), 138.7 (C-6), 104.6 (C-5), 90.0 (C-1’), 83.7 (C-4’), 73.8 (C-2’), 69.2 (C-3’), 60.6 (C-5’), 12.3 (C-7).

Synthesis of isotopically labelled ribonucleosides

Uracil-[$^{15}N_2$] (10)

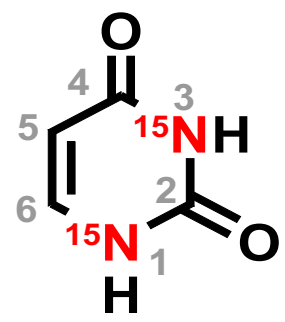

10

According to a modified literature procedure.[7]

A round-bottom flask was charged with [$^{15}N_2$]-Urea **9** (0.50 g, 8.06 mmol, 1.0 equiv.), propiolic acid (0.57 g, 8.06 mmol, 1.0 equiv.), and phosphorus(V) oxide (11.62 g, 41.0 mmol, 5.1 equiv.). The viscous mixture was gently stirred for 5 h at 85 °C and was then cooled to 0 °C using an ice bath. Subsequently, water (24 mL) was added in portions until a clear solution was formed. The mixture was allowed to crystallize at 4 °C overnight. The resulting solid was collected by vacuum filtration and washed with a small amount of cold water to yield **10** as a light-brown solid (0.51 g, 4.43 mmol, 55%).

HR MS (ESI+): *m/z* calcd for ($C_4H_5{}^{15}N_2O_2$): 115.0286 $[M+H]^+$; found: 115.0290.

^{1}H-NMR (500 MHz, DMSO-d$_6$, 295 K): δ (ppm) = 11.00 (d, ^1J = 90 Hz, ^{15}N-H, 1H, H-3), 10.79 (d, ^1J = 90 Hz, 1H, ^{15}N-H, H^1), 7.38 (ddd, J = 7.6, 5.8, 3.5 Hz, 1H, H-6), 5.44 (ddtd, J = 7.8, 4.5, 2.3, 1.3 Hz, 1H, H-5).

^{13}C-{^{1}H}-NMR (126 MHz, DMSO-d$_6$, 295 K): δ (ppm) = 164.3 (d, J = 9.2 Hz, C-4), 151.5 (dd, J = 18.6, 16.3 Hz, C-2), 142.2 (d, J = 11.0 Hz, C-6), 100.2 (d, J = 6.4 Hz, C-5).

***O*2',3',5'-Tris(benzoyl)-(*N*1,*N*3-15N)-uridine (11)**

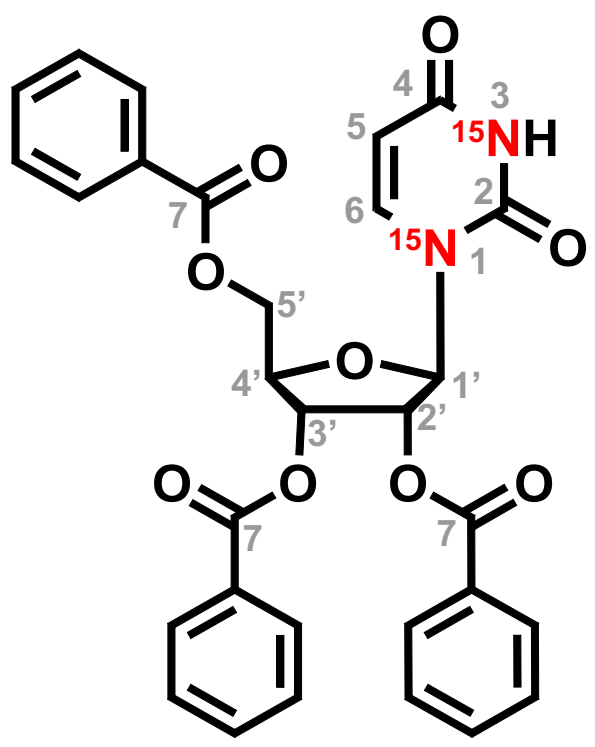

11

According to a modified literature procedure.[8]

[$^{15}N_2$]-Uracil **10** (0.50 g, 4.38 mmol, 1.0 equiv.) was suspended in acetonitrile (6.00 mL), 1'-*O*-acetyl-2',3',5'-tri-O-benzoyl-β-D-ribofuranose (2.21 g, 4.38 mmol, 1.0 equiv.) and *N,O*-bis(trimethylsilyl)acetamide (3.30 mL, 13.15 mmol, 3.0 equiv.) were added, and the mixture was stirred at 60 °C under argon atmosphere. After 30 min, trimethylsilyl trifluoromethanesulfonate (2.80 mL, 15.34 mmol, 3.5 equiv.) was adeed, and the reaction mixture was stirred for another 30 min. The solvent was removed *in vacuo*, and the oily residue was resuspended in dichloromethane (50 mL). The solution was washed with sat. aq. $NaHCO_3$ solution (50 mL), the organic layer was dried over Na_2SO_4, and the solvent was removed *in vacuo*. The crude product was purified by flash chromatography (DCM/MeOH 97:3) to afford **11** (2.33 g, 4.16 mmol, 95%) as a white foam.

HR MS (ESI+): *m/z* calcd for ($C_{30}H_{24}{}^{15}N_2O_9Na$): 581.1315 [M+Na]$^+$; found: 581.1334.

^{1}H-NMR (500 MHz, CD_2Cl_2, 295 K): δ (ppm) = 8.71 (d, ^1J = 90 Hz, 1H, ^{15}N-H, H-3), 8.10 – 7.38 (m, 15H, H^{Ar}), 7.65 – 7.56 (m, 1H, H-6), 6.28 (d, J = 4.3 Hz, 1H, H-1'), 5.89 (dd, J = 6.1, 4.8 Hz, 1H, H-3'), 5.81 – 5.77 (m, 1H, H-2'), 5.61 (ddt, J = 8.1, 5.0, 2.5 Hz, 1H, H-5), 4.67 – 4.40 (m, 2H, H-5'), 4.75 (m, 1H, H-4').

^{13}C-{^{1}H}-NMR (126 MHz, CD_2Cl_2, 295 K): δ (ppm) = 166.4 (C-7), 165.7 (C-8), 165.6 (C-9), 162.8 (d, J = 9.2 Hz, C-4), 150.4 (t, J = 18.6 Hz, C-2), 140.3 (d, J = 12.4 Hz, C-6), 134.2 –129.0 (18 × C-Ar), 103.5 (d, J = 6.9 Hz, C-5), 88.9 (d, J = 13.8 Hz, C-1'), 80.8 (C-4'), 74.2 (C-2'), 71.5 (C-3'), 64.1 (C-5').

(N^1,N^3-^{15}N)-uridine (12)

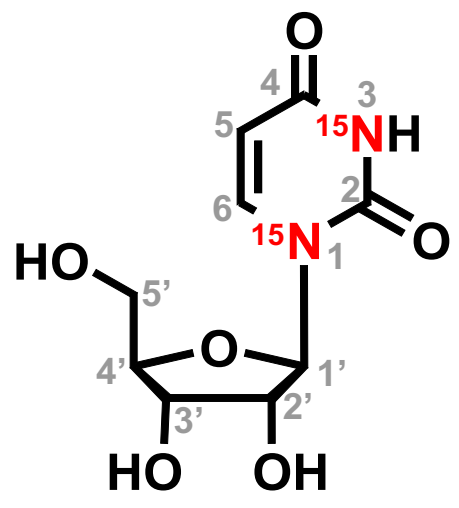

12

2',3',5'-Tri-*O*-benzoyl-[$^{15}N_2$]-uridine **11** (2.32 g, 4.15 mmol, 1.0 equiv.) was dissolved in methylamine (5.00 mL, 111.96 mmol, 27 equiv.) and absolute ethanol (10 mL). The solution was stirred at room temperature under argon atmosphere for 16 h and the solvent was then removed *in vacuo*. The oily residue was diluted with water (50 mL) and washed with DCM (30 mL). The aqueous layer was evaporated *in vacuo* and the residual off-white foam was recrystallized from ethanol to obtain **12** as a white powder (0.90 g, 3.66 mmol, 88%).

HR MS (ESI+): *m/z* calcd for ($C_9H_{12}{}^{15}N_2O_6Na$) 269.0528 $[M+Na]^+$; found: 269.0531.

^{1}H-NMR (500 MHz, DMSO-d$_6$, 295 K): δ (ppm) = 11.30 (d, ^1J = 83.5 Hz, 1H, ^{15}N-H, H-3), 7.88 (dd, J = 8.1, 2.1 Hz, 1H, H-6), 5.78 (d, J = 4.3 Hz, 1H, H-1'), 5.64 (ddd, J = 8.1, 4.5, 2.5 Hz, 1H, H-5), 5.36 (d, J = 5.8 Hz, 1H, C2'-OH), 5.11 – 5.05 (m, 1H, C3'-OH), 4.02 (qd, J = 5.4, 1.6 Hz, 1H, H-2'), 3.99 – 3.92 (m, 1H, H-3'), 3.83 (q, J = 3.3 Hz, 1H, H-4'), 3.62 (ddd, J = 12.0, 5.2, 3.3 Hz, 1H, H-5'a), 3.54 (ddd, J = 12.0, 5.1, 3.3 Hz, 1H, H-5'b).

^{13}C-{^{1}H}-NMR (126 MHz, DMSO-d$_6$, 295 K): δ (ppm) = 163.1 (d, J = 9.1 Hz, C-4), 150.7 (t, J = 17.9 Hz, C-2), 140.7 (d, J = 11.9 Hz, C-6), 101.7 (d, J = 6.2 Hz, C-5), 87.7 (d, J = 11.9 Hz, C-1'), 84.8 (C-4'), 73.5 (C-2'), 69.9 (C-3'), 60.8 (C-5').

***O*2',3',5'- Tris(*tert*-butyldimethylsilyl)-(*N*^{1},*N*^{3}-^{15}N)-uridine (13)**

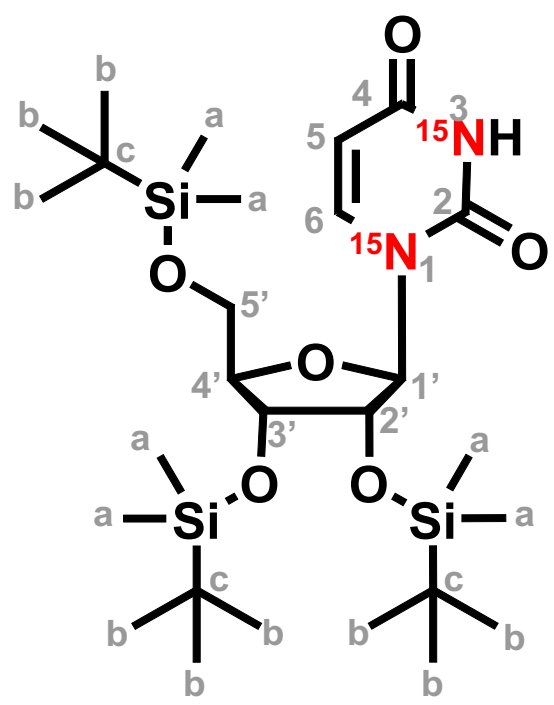

13

According to a modified literature procedure.[1]

[$^{15}N_2$]-Uridine **12** (0.90 g, 3.66 mmol, 1.0 equiv.) and imidazole (2.00 g, 29.31 mmol, 8.0 equiv.) were dissolved in dry DMF (20.00 mL) under argon atmosphere before TBDMSCl (2.76 g, 18.32 mmol, 5.0 equiv.) was added. The solution was stirred at 50 °C for 3 days. The solvent was removed *in vacuo* and the residue was taken up in EtOAc (200 mL) and sat. $NaHCO_3$ solution (200 mL). After separation, the aqueous layer was extracted with EtOAc (3 × 150 mL). The combined organic layers were washed with brine (100 mL), dried over Na_2SO_4, filtered, and evaporated *in vacuo*. The crude was purified by flash chromatography (DCM/EtOAc 95:5) to obtain **13** (1.85 g, 3.10 mmol, 86%) as a white foam.

HR MS (ESI+): *m/z* calcd for ($C_{27}H_{54}{}^{15}N_2O_6Si_3Na$): 611.3123 [M+Na]$^+$; found: 611.3114.

^{1}H-NMR (500 MHz, CD_2Cl_2, 295 K): δ (ppm) = 8.77 (d, ^1J = 90.6 Hz, 1H, ^{15}N-H, H-3), 7.96 (dd, J = 8.2, 1.9 Hz, 1H, H-6), 5.87 (dd, J = 4.3, 1.4 Hz, 1H, H-1'), 5.66 (ddd, J = 10.8, 4.8, 2.5 Hz, 1H, H-5), 4.14 – 4.04 (m, 3H, H-2',3',4'), 3.97 (dd, J = 11.6, 2.3 Hz, 1H, H-5'a), 3.77 (dd, J = 11.7, 1.7 Hz, 1H, H-5'b), 0.96 (s, 9H, H-b), 0.92 (s, 9H, H-b), 0.89 (s, 9H, H-b), 0.14 (s, 6H, H-a), 0.10 (s, 6H, H-a), 0.07 (s, 6H, H-a).

^{13}C-{^{1}H}-NMR (126 MHz, CD_2Cl_2, 295 K): δ (ppm) = 163.4 (t, J = 8.7 Hz, C-4), 150.7 (td, J = 18.6, 2.5 Hz, C-2), 140.7 (d, J = 12.4 Hz, C-6), 102.3 (d, J = 6.9 Hz, C-5), 88.9 (d, J = 11.5 Hz, C-1'), 85.5 (d, C-4'), 76.6 (C-2'), 71.8 (C-3'), 62.6 (C-5'), 26.2 (C-b), 26.0 (C-b), 26.0 (C-b), 18.8 (C-c), 18.4 (C-c), 18.3 (C-c), −4.1 (C-a), −4.5 (C-a), −4.6 (C-a), −4.7 (C-a), −5.3 (C-a), −5.4 (C-a).

***O*2',3',5'- Tris(*tert*-butyldimethylsilyl)-(N^1,N^3,N^4-^{15}N)-cytidine (14)**

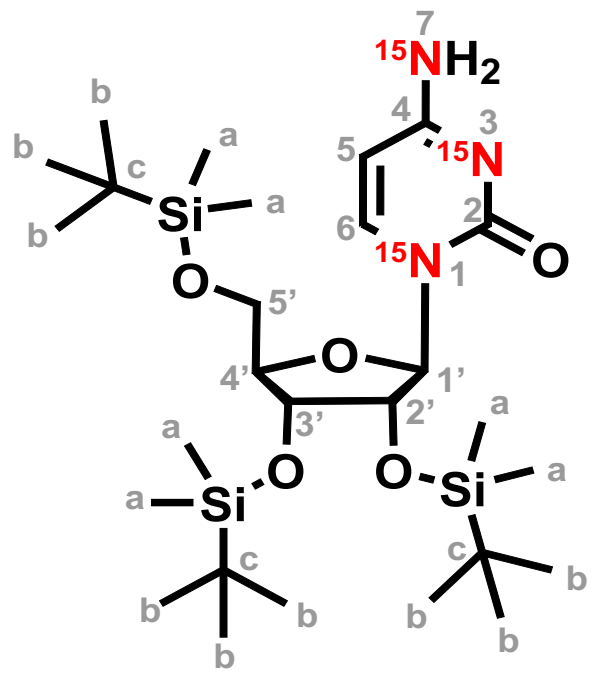

14

According to a modified literature procedure.[9]

In a round bottom flask, 1,2,4-Triazole (1.06 g, 15.28 mmol, 9.0 equiv.) was dried under vacuum for 30 min before MeCN (30 mL) was added under an argon atmosphere. The solution was cooled to 0 °C, and $POCl_3$ (0.31 mL, 3.40 mmol, 2.0 equiv.) was added dropwise. After 10 min, NEt_3 (2.00 mL, 14.39 mmol, 8.6 equiv.) was added, and the mixture was stirred for an additional 20 min. Subsequently, 2′,3′,5′-Tri-O-*tert*- butyldimethylsilyl-[$^{15}N_2$]-uridine **13** (1.00 g, 1.70 mmol, 1.0 equiv.) was added, and the reaction mixture was heated to 60 °C and stirred overnight. The mixture was washed with brine (50 mL) and extracted with DCM (3 × 100 mL). The combined organic layers were dried over Na_2SO_4, filtered, and concentrated *in vacuo* to yield an orange foam, which was transferred to a pressure tube. $^{15}NH_4Cl$ (462.60 mg, 8.50 mmol, 5.0 equiv.), K_2CO_3 (1.20 g, 8.50 mmol, 5.0 equiv.), and dry DMSO (8 mL) were added. The mixture was stirred at 80 °C for 3 days. After cooling, the reaction mixture was diluted with DCM (50 mL) and washed sequentially with sat. $NaHCO_3$ (100 mL) solution, 5 % aq. citric acid (100 mL), sat. $NaHCO_3$ solution (100 mL), and brine (100 mL). The organic layer was dried over Na_2SO_4, filtered, and the solvent was removed *in vacuo*. The crude product was purified by flash chromatography (stepwise gradient MeOH/DCM 99:1 to 93:7) to afford the **14** as a white solid (0.56 mg, 0.95 mmol, 56%).

HR MS (ESI+): *m/z* calcd for ($C_{27}H_{55}{}^{15}N_3O_5Si_3Na$): 611.3253 [M+Na]$^+$; found: 611.3238.

^{1}H-NMR (500 MHz, CD_2Cl_2, 295 K): δ (ppm) = 8.12 (d, J = 7.5 Hz, 1H, H-6), 5.78 (d, J = 1.5 Hz, 1H, H-1'), 5.65 (dd, J = 7.4, 3.4 Hz, 1H, H-5), 4.11 – 4.02 (m, 4H, H-2',3',4',5'a), 3.79 (dd, J = 11.7, 1.4 Hz, 1H, H-5'b), 0.96 (s, 9H, H-b), 0.90 (s, 18H, H-

b), 0.18 (s, 3H, H-a), 0.14 (s, 3H, H-a), 0.13 (s, 3H, H-a), 0.11 (s, 3H, H-a), 0.07 (s, 6H, H-a).

^{13}C-{^{1}H}-NMR (126 MHz, CD_2Cl_2, 295 K): δ (ppm) = 165.9 (dd, J = 20.2, 6.0 Hz, C-4), 156.1 – 155.6 (m, C-2), 142.4 (d, J = 12.9 Hz, C-6), 93.5 (d, J = 3.6 Hz, C-5), 90.4 (d, J = 10.1 Hz, C-1'), 83.7 (C-4'), 76.8 (C-2'), 70.2 (C-3'), 61.6 (C-5'), 26.3 (C-b), 26.1 (C-b), 18.8 (C-c), 18.4 (C-c), 18.4 (C-c), −3.9 (C-a), −4.1 (C-a), −4.8 (C-a), −4.9 (C-a), −5.2 (C-a), −5.4 (C-a).

*O*2',3',5'-Tris(*tert*-butyldimethylsilyl)-5-iodo-(N^1,N^3,N^4-^{15}N)-cytidine (15)

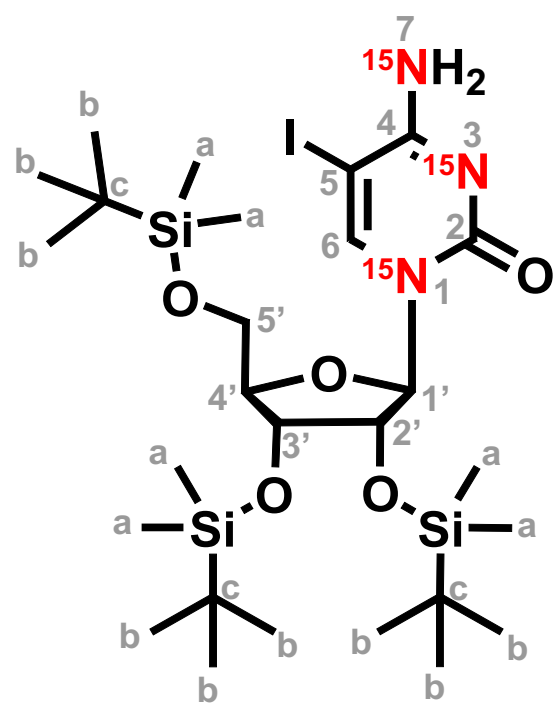

15

According to a modified literature procedure.[3]

2',3',5'-Tri-*O*-*tert*-butyldimethylsilyl-[$^{15}N_2$]-cytidine **14** (519 mg, 0.88 mmol, 1.0 equiv.) was dissolved in MeCN (28 mL) under an argon atmosphere. $(NH_4)_2[Ce(NO_3)_6]$ (966 mg, 1.76 mmol, 2.0 equiv.) and iodine (447 mg, 1.76 mmol, 2.0 equiv.) were added, and the resulting red mixture was heated to reflux for 90 min at 60 °C. After complete conversion, the reaction was quenched with sat. $NaHCO_3$ solution (25 mL) and with sat. $Na_2S_2O_3$ solution (25 mL). The compound was extracted with DCM (8 × 25 mL) and the combined organic layers were washed with brine (2 × 50 mL) and dried over Na_2SO_4. After removing the solvent *in vacuo*, the crude product was purified by flash chromatography (DCM/MeOH 98:2) to obtain **15** as a yellow solid (479 mg, 0.67 mmol, 76%).

HR MS (ESI+): *m/z* calcd for ($C_{27}H_{54}I^{15}N_3O_5Si_3Na$): 737.2219 $[M+Na]^+$; found: 737.2210.

^{1}H-NMR (500 MHz, CD_2Cl_2, 295 K): δ (ppm) = 8.03 (s, 1H, H-6), 5.93 (dd, J = 5.1, 1.3 Hz, 1H, H-1'), 4.10 (ddd, J = 5.3, 4.5, 1.4 Hz, 1H, H-2'), 4.07 (dt, J = 4.4, 2.3 Hz, 1H, H-4'), 4.03 (t, J = 4.2 Hz, 1H, H-3'), 3.96 (dd, J = 11.6, 2.3 Hz, 1H, H-5'a), 3.77 (dd, J = 11.6, 2.4 Hz, 1H, H-5'b), 0.99 (s, 9H, H-b), 0.92 (s, 9H, H-b), 0.87 (s, 9H, H-b), 0.20 (s, 3H, H-a), 0.20 (s, 3H, H-a), 0.10 (s, 3H, H-a), 0.09 (s, 3H, H-a), 0.05 (s, 3H, H-a), 0.03 (s, 3H, H-a).

^{13}C-{^{1}H}-NMR (126 MHz, CD_2Cl_2, 295 K): δ (ppm) = 163.8 (dd, J = 22.5, 5.5 Hz, C-4), 154.9 (td, J = 11.5, 3.7 Hz, C-2), 147.4 (d, J = 11.9 Hz, C-6), 89.1 (d, J = 10.1 Hz, C-1'), 85.6 (C-4'), 76.9 (C-2'), 72.2 (C-3'), 63.1 (C-5'), 26.6 (C-b), 26.1 (C-b), 26.0 (C-b), 19.1 (C-c), 18.4 (C-c), 18.3 (C-c), −4.1 (C-a), −4.5 (C-a), −4.6 (C-a), −4.7 (C-a), −4.9 (C-a).

*O*2',3',5'-Tris(*tert*-butyldimethylsilyl)-5-(methoxycarbonyl)-(*N*^{1},*N*^{3},*N*^{4}-^{15}N)-cytidine (16)

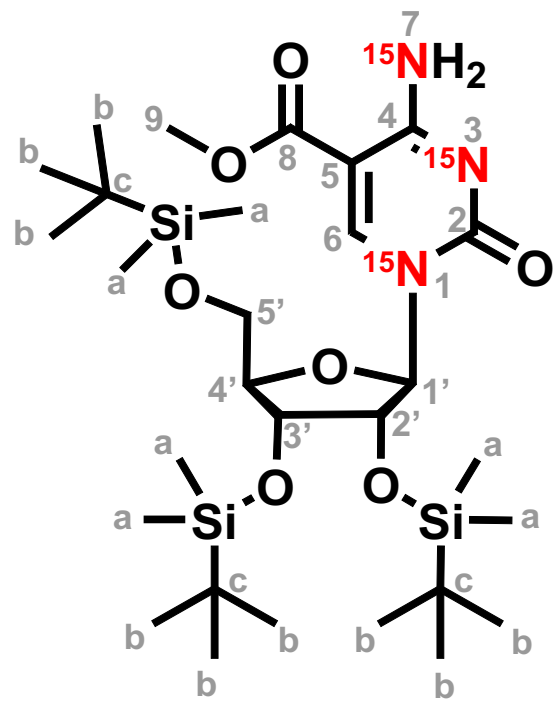

16

According to a modified literature procedure.[4]

In a dry high-pressure autoclave glass vessel, 5-iodo- 4-amino-2′,3′,5′-tri-*O*-*tert*-butyldimethylsilyl-[$^{15}N_3$]-cytidine **15** (70.0 mg, 97.92 µmol, 1.0 equiv.) and $PdCl_2(MeCN_3)_2$ (1.40 mg, 5.40 µmol, 0.1 equiv.) were dissolved in dry MeOH (10 mL) under an argon flow. The vessel was sealed and flushed with CO gas (3.50 bar) twice before DIPEA (34.1 µL, 195.84 µmol, 2.0 equiv.) was added through the syringe inlet. The vessel was then pressurized with CO gas (3.50 bar) again and the mixture was stirred overnight at 60 °C. The mixture was then filtered through Celite and the solvent evaporated *in vacuo*. The crude was purified by flash chromatography (DCM/MeOH 99:1) to obtain **16** as a white solid (97.0 mg, 0.15 mmol, 89%).

HR MS (ESI+): *m/z* calcd for ($C_{29}H_{57}{}^{15}N_3O_7Si_3Na$): 669.3308 $[M+Na]^+$; found: 669.3294.

^{1}H-NMR (500 MHz, CD_2Cl_2, 295 K): δ (ppm) = 8.48 (s, 1H, H-6), 8.03 – 7.84 (m, 1H, ^{15}N-H, H-3), 6.34 (d, ^1J = 87.8 Hz, ^{15}N-H, H-7), 5.94 (d, *J* = 6.3 Hz, 1H, H-1'), 4.18 (td, *J* = 4.7, 1.3 Hz, 1H, H-2'), 4.12 (td, *J* = 4.2, 2.5 Hz, 1H, H-4'), 3.96 (t, *J* = 4.3 Hz, 1H, H-3'), 3.93 (dd, *J* = 11.5, 2.5 Hz, 1H, H-5'a), 3.83 (s, 3H, H^9), 3.78 (dd, *J* = 11.5, 4.1 Hz, 1H, H-5'b), 0.95 (s, 9H, H-b), 0.92 (s, 9H, H-b), 0.88 (s, 9H, H-b), 0.15 (s, 3H, H-a), 0.13 (s, 3H, H-a), 0.10 (s, 3H, H-a), 0.08 (s, 3H, H-a), 0.06 (s, 3H, H-a), 0.03 (s, 3H, H-a).

^{13}C-{^{1}H}-NMR (126 MHz, CD_2Cl_2, 295 K): δ (ppm) = 165.7 (C-8), 163.7 (dd, *J* = 21.4, 6.2 Hz, C-4), 154.4 – 154.0 (m, C-2), 148.4 (d, *J* = 13.8 Hz, C-6), 96.2 (C-5), 89.9 (d, *J* = 10.1 Hz, C-1'), 85.8 (C-4'), 76.6 (C-2'), 72.5 (C-3'), 63.8 (C-5'), 52.4 (C-9), 26.3 (C-b), 26.0 (C-b), 26.0 (C-b), 18.8 (C-c), 18.4 (C-c), 18.3 (C-c), −4.1 (C-a), −4.5 (C-a), −4.6 (C-a), −4.6 (C-a), −5.2 (C-a), −5.4 (C-a).

5-Carboxyl-(*N*^{1},*N*^{3},*N*^{4}-^{15}N)-cytidine ([$^{15}N_3$]-ca5C)

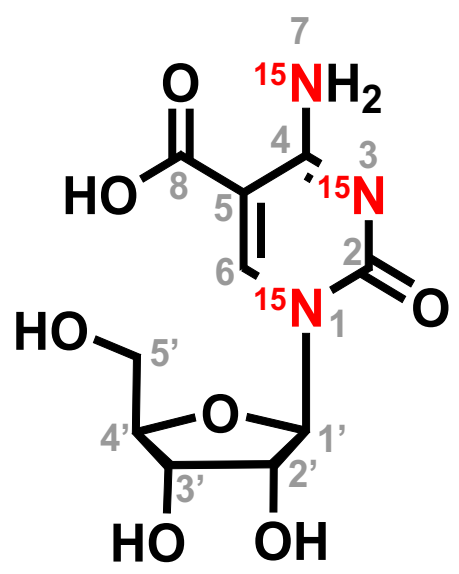

[$^{15}N_3$]-ca5C

According to a modified literature procedure.[5]

Methyl 2′,3′,5′-tri-*O*-*tert*-butyldimethylsilyl-[$^{15}N_3$]-cytidine-5-carboxylate **16** (20.0 mg, 30.9 µmol, 1.0 equiv.) was dissolved in a mixture of EtOAc (2 mL) and pyridine (40 µL) under an argon atmosphere. HF·Py 70% (15.0 µL, 0.37 mmol, 12.0 equiv.) was added using a plastic pipette. The solution was stirred for 2 days at 60 °C. The mixture was then quenched with methoxytrimethylsilane (1.0 mL) and the solvent evaporated *in vacuo*. The crude product was dissolved in a mixture of MeCN (2.0 mL) and water (0.5 mL). LiOH (8.90 mg, 371 µmol, 12 equiv.) was added and the solution was stirred at room temperature for 2 d. It was then quenched with HCl·MeOH (0.25 mL, 3 N). The

product was purified by HPLC (H_2O, 0–10% MeCN over 45 min) to obtain **[$^{15}N_3$]-ca5C** (5.70 mg, 19.65 µmol, 63%) as a white solid.

HR MS (ESI–): *m/z* calcd for ($C_{10}H_{13}{}^{15}N_3O_7$): 289.0592 [M–H]$^-$; found: 289.0591.

^{1}H-NMR (500 MHz, DMSO-d$_6$, 295 K): δ (ppm) = 8.70 (d, 1H, ^1J = 91.5 Hz, ^{15}N-H, H-7), 8.56 (s, 1H, H-6), 7.65 (dd, 1H, J = 90.9, 5.3 Hz, ^{15}N-H, H-7), 5.79 (dd, J = 4.4, 1.0 Hz, 1H, H-1'), 3.97 (td, J = 4.8, 1.4 Hz, J = 4.8, 1.4 Hz, 1H, H-2'), 3.91 (t, J = 5.1 Hz, 1H, H-3'), 3.85 (dt, J = 5.3, 3.4 Hz, 1H, H-4'), 3.64 (dd, J = 11.9, 3.1 Hz, 2H, H-5'a), 3.54 (dd, J = 11.9, 3.8 Hz, 2H, H-5'b).

^{13}C-{^{1}H}-NMR (126 MHz, DMSO-d$_6$, 295 K): δ (ppm) = 166.9 (C-8), 164.3 (dd, J = 19.9, 4.7 Hz, C-4), 154.6 – 154.0 (m, C-2), 147.0 (d, J = 12.4 Hz, C-6), 99.6 – 99.0 (m, C-5), 89.5 (d, J = 10.6 Hz, C-1'), 84.3 (C-4'), 74.1 (C-2'), 69.6 (C-3'), 60.8 (C-5').

*O*2',3',5'-Tris(*tert*-butyldimethylsilyl)-5-formyl-(*N*^{1},*N*^{3},*N*^{4}-^{15}N)-cytidine (17)

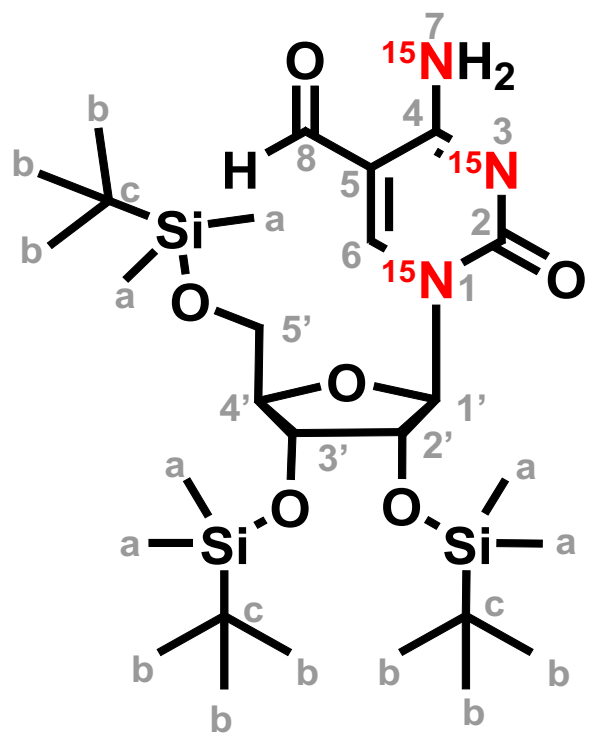

17

According to a modified literature procedure.[5]

In a dry high-pressure autoclave glass vessel, 5-Iodo-4-amino-2′,3′,5′-tri-*O*-*tert*-butyl-dimethylsilyl-[$^{15}N_3$]-cytidine **15** (200 mg, 279 µmol, 1.0 equiv.), [$Pd_2(dba)_3$]·$CHCl_3$ (29.1 mg, 28.00 µmol, 0.1 equiv.) and PPh_3 (44.0 mg, 167.8 µmol, 0.6 equiv.) were dissolved in dry Toluene (20 mL) under an argon counterflow. The vessel was sealed and flushed with CO gas (3.50 bar) twice. The vessel was pressurized again with CO gas (3.50 bar) before $HSnBu_3$ (125 mg, 418 µmol, 1.5 equiv.) was added through the syringe inlet over 10 hours while the mixture was stirred at 65 °C overnight. The mixture was then filtered through Celite and the solvent evaporated *in vacuo*, the crude product was purified by flash chromatography (DCM/MeOH 98:2) to obtain **17** (142 mg, 0.23 mmol, 83%) as a white solid.

HR MS (ESI+): *m/z* calcd for ($C_{28}H_{55}{}^{15}N_3O_6Si_3Na$): 639.3202 $[M+Na]^+$, found: 639.3188.

^{1}H-NMR (500 MHz, CD_2Cl_2, 295 K): δ (ppm) = 9.51 (s, 1H, H-8), 8.57 (s, 1H, H-6), 8.14 (d, ^1J = 92.9 Hz, 1H, ^{15}N-H, H-7), 6.26 (d, ^1J = 90.9 Hz, 1H, ^{15}N-H, H-7), 5.86 (d, J = 2.0 Hz, 1H, H-1'), 4.19 (t, J = 3.6 Hz, 1H, H-2'), 4.16 (dt, J = 6.3, 2.3 Hz, 1H, H-4'), 4.10 (dd, J = 11.8, 2.3 Hz, 1H, H-5'a), 4.01 (dd, J = 6.3, 4.1 Hz, 1H, H-3'), 3.83 (dd, J = 11.9, 2.3 Hz, 1H, H-5'b), 0.98 – 0.89 (m, 27H, H-b), 0.18 – 0.07 (m, 18H, H-a).

^{13}C-{^{1}H}-NMR (126 MHz, CD_2Cl_2, 295 K): δ (ppm) = 187.6 (d, J = 2.3 Hz, C-8), 163.0 (dd, J = 21.1, 5.6 Hz, C-4), 153.8 (d, J = 13.8 Hz, C-6), 153.6 – 153.4 (m, C-2), 105.8 (C-5), 90.7 (d, J = 8.7 Hz, C-1'), 84.6 (C-4'), 77.0 (C-2'), 70.7 (C-3'), 62.3 (C-5'), 26.5 (C-b), 26.1 (C-b), 19.1 (C-c), 18.3 (C-c), −3.9 (C-a), −4.2 (C-a), −4.6 (C-a), −4.8 (C-a), −4.8 (C-a), −5.2 (C-a).

5-Formyl-(*N*^{1},*N*^{3},*N*^{4}-^{15}N)-cytidine ([$^{15}N_3$]-f5C)

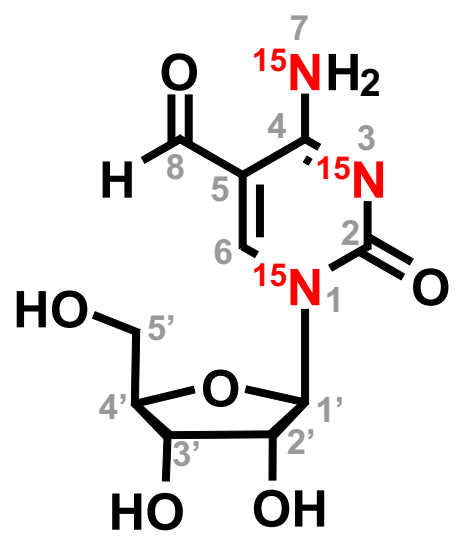

[$^{15}N_3$]-f5C

According to a modified literature procedure.[5]

In a plastic falcon tube, 5-Formyl-2′,3′,5′-tri-*O*-*tert*-butyldimethylsilyl-[$^{15}N_3$]-cytidine **10** (60.0 mg, 97.3 µmol, 1.0 equiv.) was dissolved in a mixture of EtOAc (2.0 mL) and pyridine (80.00 µL) under an argon atmosphere. HF·Py 70% (35.0 µL, 1.19 mmol, 12.0 equiv.) was added using a plastic pipette. The solution was stirred for 2 days at 60 °C. The mixture was then quenched with methoxytrimethylsilane (1.0 mL) and the solvent evaporated *in vacuo*. The crude product was purified by (H_2O, 0–10% MeCN over 45 min) to obtain **[$^{15}N_3$]-f5C** (14.4 mg, 53.0 µmol, 54%) as a white solid.

HR MS (ESI−): *m/z* calcd for ($C_{10}H_{13}{}^{15}N_3O_6Cl$) 309.0409 $[M+Cl]^-$; found: 309.0413.

^{1}H-NMR (500 MHz, DMSO-d_6, 295 K): δ (ppm) = 9.42 (s, 1H, H-8), 9.02 (s, 1H, H-6), 8.12 (d, ^1J = 91.5, 1H, ^{15}N-H, H-7), 7.85 (ddd, J = 90.9, 6.7, 3.6 Hz, 1H, ^{15}N-H, H-7),

5.76 (dd, *J* = 2.1, 1.0 Hz, 1H, H-1'), 5.54 (d, *J* = 4.9 Hz, 1H, C-2'-OH), 5.27 (t, *J* = 5.0 Hz, 1H, C-5'-OH), 5.03 (d, *J* = 6.1Hz, 1H, C-3'-OH), 4.05 – 3.98 (m, 2H, H-2',3'), 3.93 (dt, *J* = 6.5, 2.9 Hz, 1H, H-4'), 3.83 (ddd, *J* = 12.3, 5.2, 2.8 Hz, 1H, H-5'a), 3.64 (ddd, *J* = 12.3, 4.9, 2.8 Hz, 1H, H-5'b).

^{13}C-{^{1}H}-NMR (126 MHz, DMSO-d$_6$, 295 K): δ (ppm) = 188.3 (d, *J* = 2.8 Hz, C-8), 162.1 (dd, *J* = 20.0, 5.3 Hz, C-4), 155.0 (d, *J* = 13.3 Hz, C-6), 153.0 – 152.4 (m, C-2), 104.8 (C-5), 90.4 (d, *J* = 9.2 Hz, C-1'), 83.9 (C-4'), 74.4 (C-2'), 68.0 (C-3'), 59.5 (C-5').

*O*2',3',5'-Tris(*tert*-butyldimethylsilyl)-5-hydroxymethyl-(*N*^{1},*N*^{3},*N*^{4}-^{15}N)-cytidine (18)

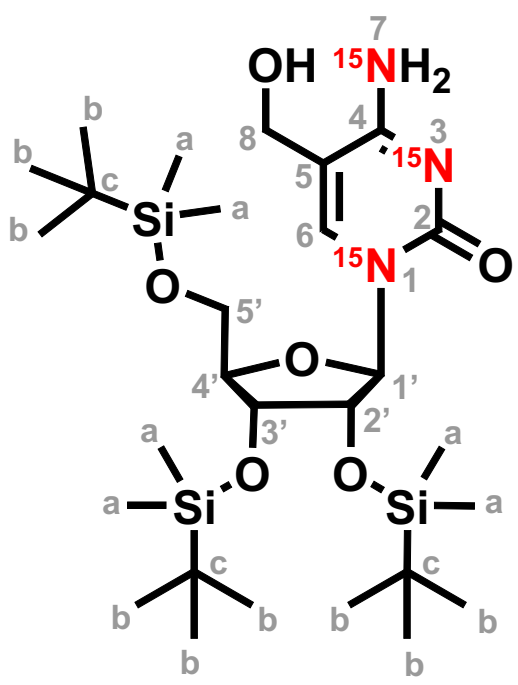

18

According to a modified literature procedure.[5]

5-Formyl-2′,3′,5′-tri-*O*-*tert*-butyldimethylsilyl-[$^{15}N_3$]-cytidine **17** (90.0 mg, 146 µmol, 1.0 equiv.) and $CeCl_3{\cdot}7H_2O$ (163 mg, 437 µmol, 3.0 equiv.) were dissolved in MeOH (7.30 mL) under an argon atmosphere. $NaBH_4$ (5.52 mg, 146 µmol, 1.0 equiv.) was added, and the reaction mixture was stirred for 3 hours at room temperature and then quenched with a saturated ammonium chloride solution (60 mL). The aqueous layer was extracted with EtOAc (2×60 mL), the resulting organic layer was dried over Na_2SO_4 and the solvent was then removed *in vacuo*. The crude residue was purified by flash chromatography (DCM/MeOH 95:5) to afford a colourless solid, which was recrystallized from hexane to yield **18** (73.0 mg, 118 µmol, 81%) as colourless crystals.

HR MS (ESI+): *m/z* calcd for ($C_{28}H_{57}{}^{15}N_3O_6Si_3Na$): 641.3358 $[M+Na]^+$; found: 641.3352.

^{1}H-NMR (500 MHz, CD_2Cl_2, 295 K): δ (ppm) = 7.57 (d, *J* = 5.0 Hz, 1H, H-6), 5.77 (d, *J* = 4.4 Hz, 1H, H-1'), 4.42 (d, 1H, H-8), 4.46 – 4.32 (m, 1H, H-8), 4.06 (dq, *J* = 5.5,

2.8 Hz, 2H, H-2',4'), 3.99 (dd, J = 11.6, 2.4 Hz, 1H, H-5'a), 3.97 – 3.93 (m, 1H, H-3'), 3.76 (dd, J = 11.6, 2.5 Hz, 1H, H-5'b), 0.96 (s, 9H, H-b), 0.91 (s, 9H, H-b), 0.88 (s, 9H, H-b), 0.14 (d, 6H, H-a), 0.07 (d, 6H, H-a), 0.05 (d, 6H, H-a).

^{13}C-{^{1}H}-NMR (126 MHz, CD_2Cl_2, 295 K): δ (ppm) = 165.5 (dd, J = 20.4, 7.0 Hz, C-4), 157.3 – 155.8 (m, C-2), 138.6 (d, J = 13.9 Hz, C-6), 106.5 (C-5), 90.4 – 88.2 (m, C-1'), 84.9 (C-4'), 76.9 (C-2'), 71.5 (C-3'), 62.8 (C-5'), 60.2 (C-8), 27.3 (C-b), 26.4 (C-b), 26.1 (d, C-b), 18.9 (C-c), 18.4 (C-c), 18.3 (C-c), −4.0 (C-a), −4.2 (C-a), −4.7 (C-a), −4.8 (C-a), −5.0 (C-a), −5.1 (C-a).

5-Hydroxymethyl-(N^1,N^3,N^4-^{15}N)-cytidine ([^{15}N$_3$]-hm5C)

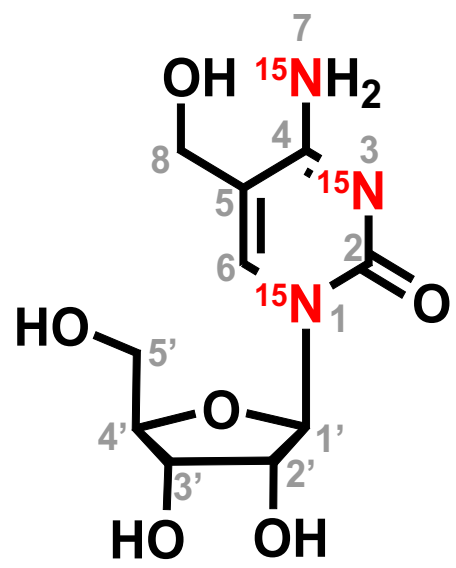

[^{15}N$_3$]-hm5C

According to a modified literature procedure.[5]

In a plastic falcon tube, 5-Formyl-2′,3′,5′-tri-*O*-*tert*-butyldimethylsilyl-[^{15}N$_3$]cytidine **18** (70.0 mg, 113 µmol, 1.0 equiv.) was dissolved in a mixture of EtOAc (2.0 mL) and pyridine (80.0 µL) under an argon atmosphere. HF·Py 70% (40.0 µL, 1.67 mmol, 12.0 equiv.) was added using a plastic pipette. The solution was stirred overnight at 60 °C to precipitate the product. The solution was stirred for 2 days at 60 °C. The mixture was then quenched with methoxytrimethylsilane (1.0 mL) and the solvent evaporated *in vacuo*. The crude product was purified by HPLC (H_2O, 0–10% MeCN over 45 min) to obtain **[^{15}N$_3$]-hm5C** (15.1 mg, 54.7 µmol, 48%) as a white solid.

HR MS (ESI−): *m/z* calcd for ($C_{10}H_{15}{}^{15}N_3O_6Cl$): 311.0566 [M+Cl]$^-$; found: 311.0569.

^{1}H-NMR (500 MHz, DMSO-d$_6$, 295 K): δ (ppm) = 7.81 (s, 1H, H-6), 7.33 (d, ^1J = 89.9 Hz, 1H, ^{15}N-H, H-7), 6.58 (d, ^1J = 90.2 Hz, 1H, ^{15}N-H, H-7), 5.80 – 5.76 (m, 1H, H-1'), 5.29 – 5.24 (m, 1H, C-2'-OH), 5.05 (t, J = 5.2 Hz, 1H, C-5'-OH), 5.02 – 4.95 (m, 2H, C3',8-OH), 4.18 (d, J = 5.3 Hz, 2H, H-8), 3.98 – 3.91 (m, 2H, H-2',3'), 3.86 – 3.79

(m, 1H, H-4’), 3.66 (ddd, J = 12.0, 5.1, 3.2 Hz, 1H, H-5’a), 3.55 (ddd, J = 11.9, 5.3, 3.6 Hz, 1H, H-5’b).

^{13}C-{^{1}H}-NMR (126 MHz, DMSO-d_6, 295 K): δ (ppm) = 164.4 (dd, J = 19.8, 5.5 Hz, C-4), 155.5 – 154.6 (m, C-2), 139.6 (d, J = 12.9 Hz, C-6), 105.8 (C-5), 89.1 (d, J = 11.5 Hz, C-1’), 84.1 (C-4’), 73.9 (C-2’), 69.5 (C-3’), 60.7 (C-5’), 57.5 (C-8).

Analytical Methods

HPLC

HPLC for nucleoside/nucleobase studies

Table S1: Detailed information of the applied analytical HPLC method for the separation of the cytidine and cytosine derivatives.

column	ACE® C18-PFP(150×4.6 mm; 5 μm)
column temperature [°C]	30.0
eluent (A/B)	H_2O + 0.1% FA/MeOH + 0.1% FA
eluent flow [mL min^{-1}]	1.500
eluent gradient	from 100/0 to 99.7/0.3 in 5 min, to 90/10 in 5 min, to 0/100 in 1 min, hold for 6 min, to 100/0 in 1 min, hold for 7 min
Total run time [min]	24.0
monitored λ_{abs} [nm]	280
sample concentration [mg mL^{-1}]	0.3
injection volume [μL]	5

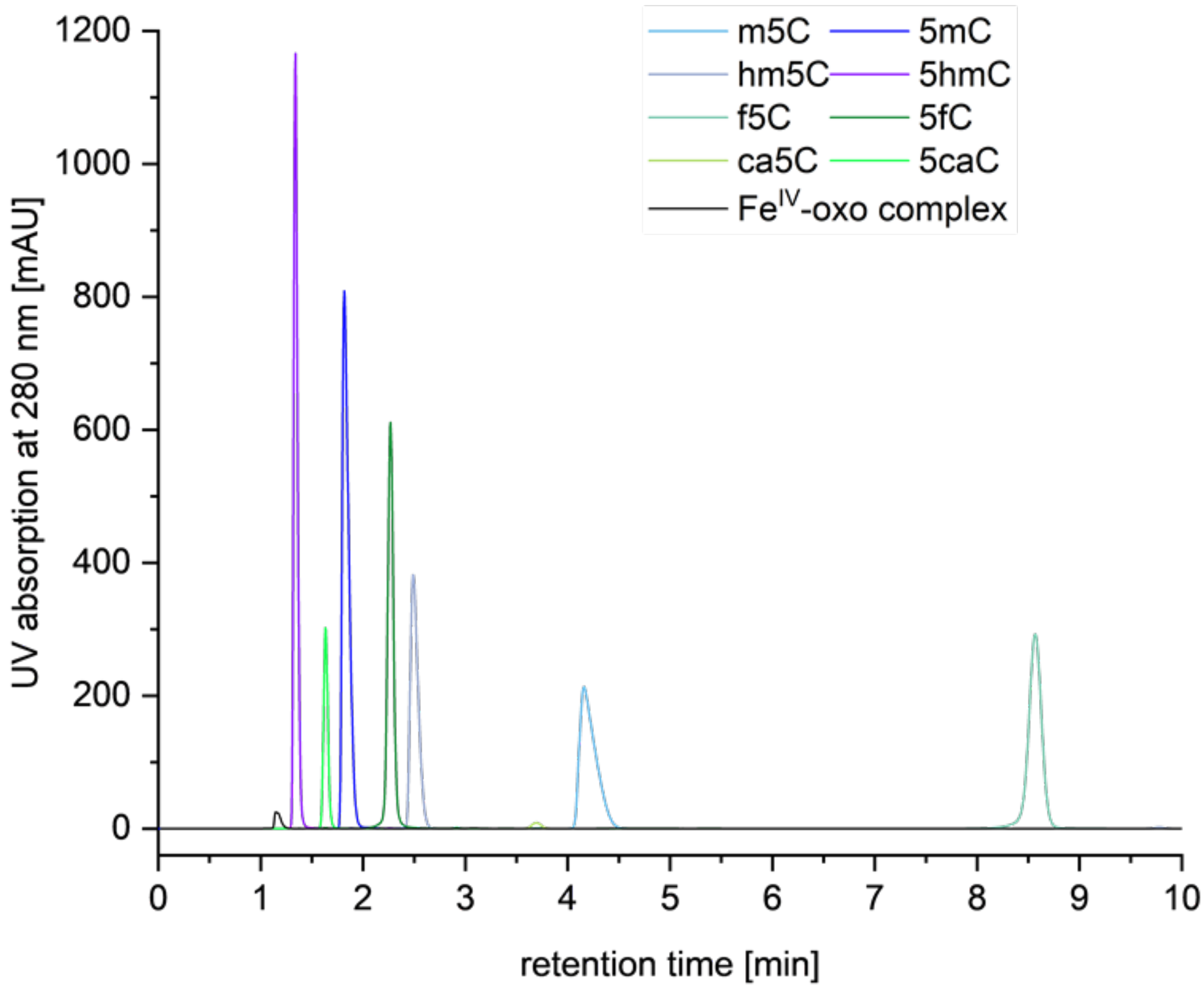

Figure S1: HPLC-chromatogram from the measurement of the reference samples. Conditions: c = 0.3 mg mL^{-1}, 30 °C, H_2O.

HPLC for Oligonucleotide studies

$[Fe^{IV}\mathbf{L1}(O)]^{2+}$ (1 mM, 12 or 24 equivalents) was added to RNA-oligo (50 µM, 100 µL) and water to give a final volume of 500 µL at timepoint t = 0. Samples were incubated (300 rpm) for 60 min. Reactions were quenched by placement on ice and removal of $[Fe^{IV}\mathbf{L1}(O)]^{2+}$ from the sample mixture by transfer of the reaction volume to centrifugal spin columns (Amicon® Ultra Centrifugal Filter, 3 kDa MWCO, Millipore, Darmstadt, Germany) and addition of 4 mL of water before centrifugation (4,000 g, 20 min, 4 °C). Three centrifugation steps with addition of 4 mL water between centrifugation steps were performed to achieve $[Fe^{IV}\mathbf{L1}(O)]^{2+}$ removal. After the last centrifugation step, sample was lyophilised overnight and resuspended in 20 µL of water before subjection to HPLC (injection volume 15 – 20 µL). Sample identity was confirmed by MALDI. All reactions were performed in duplicate.

Analytical RP-HPLC experiments were carried out using an Agilent Technologies 1260 Infinity II System (Agilent, Waldbronn, Germany) equipped with a G7165A detector and an autosampler module. Separation was performed on an EC 250/4 Nucleodur 100-3 C18ec column from Macherey Nagel (Düren, Germany). A flow rate of 0.8 mL/min was used at 60 °C, following the gradient described in the respective experimental procedure. For RNA analysis, a buffer system was employed consisting of buffer A (100 mM triethylammonium acetate in water, pH 7) and buffer B (100 mM triethylammonium acetate in 80% acetonitrile in water, pH 7). The chromatographic run was monitored at a wavelength of 260 nm.

Table S2: Detailed information of the applied analytical HPLC method for the separation of the RNA 1 oxidation products.

column	EC 250/4 Nucleodur 100-3 C18ec column from Macherey Nagel (3 µm, 250x4 mm)
column temperature [°C]	60.0
eluent (A/B)	H_2O + 100 mM TEAA, pH 7/ 80% ACN in H_2O + 100 mM TEAA, pH 7
eluent flow [mL min^{-1}]	0.8
eluent gradient	8-16%
Total run time [min]	35.0
monitored λ_{abs} [nm]	260
injection volume [µL]	15-20

MALDI-TOF-MS for Qualitative Oligonucleotide Analysis

Non-digested sample (4 μL) was desalted (0.025 μm MCE membrane, 1 h, room temperature, Merck Millipore, Darmstadt, Germany) using double-distilled water (ddH_2O), and subsequently co-crystallised in an HPA matrix (0.7 M 3-hydroxypicolinic acid in ddH_2O with 0.07 M diammonium citrate) in a 1:1 or 2:1 ratio.

MALDI-MS spectrometry was performed using a Bruker Autoflex II equipped with a time-of-flight (TOF) detector. Before analysis, the probes were desalted on a 0.025 µm VSWP filter using double-distilled water (ddH_2O), and subsequently co-crystallized in an HPA matrix (0.7 M 3-hydroxypicolinic acid in ddH_2O with 0.07 M diammonium citrate). Samples were analyzed in linear positive mode with a mass range of m/z 3000–10000. Each sample was measured with 2000 laser shots at a frequency of 2000 Hz. The detector gain was set to 2829 V (4.5×). The Smartbeam parameter was set to “ultra”, with ion source 1 at 19.50 kV, ion source 2 at 18.25 kV, and the lens voltage at 7.80 kV.

Flow-through Control

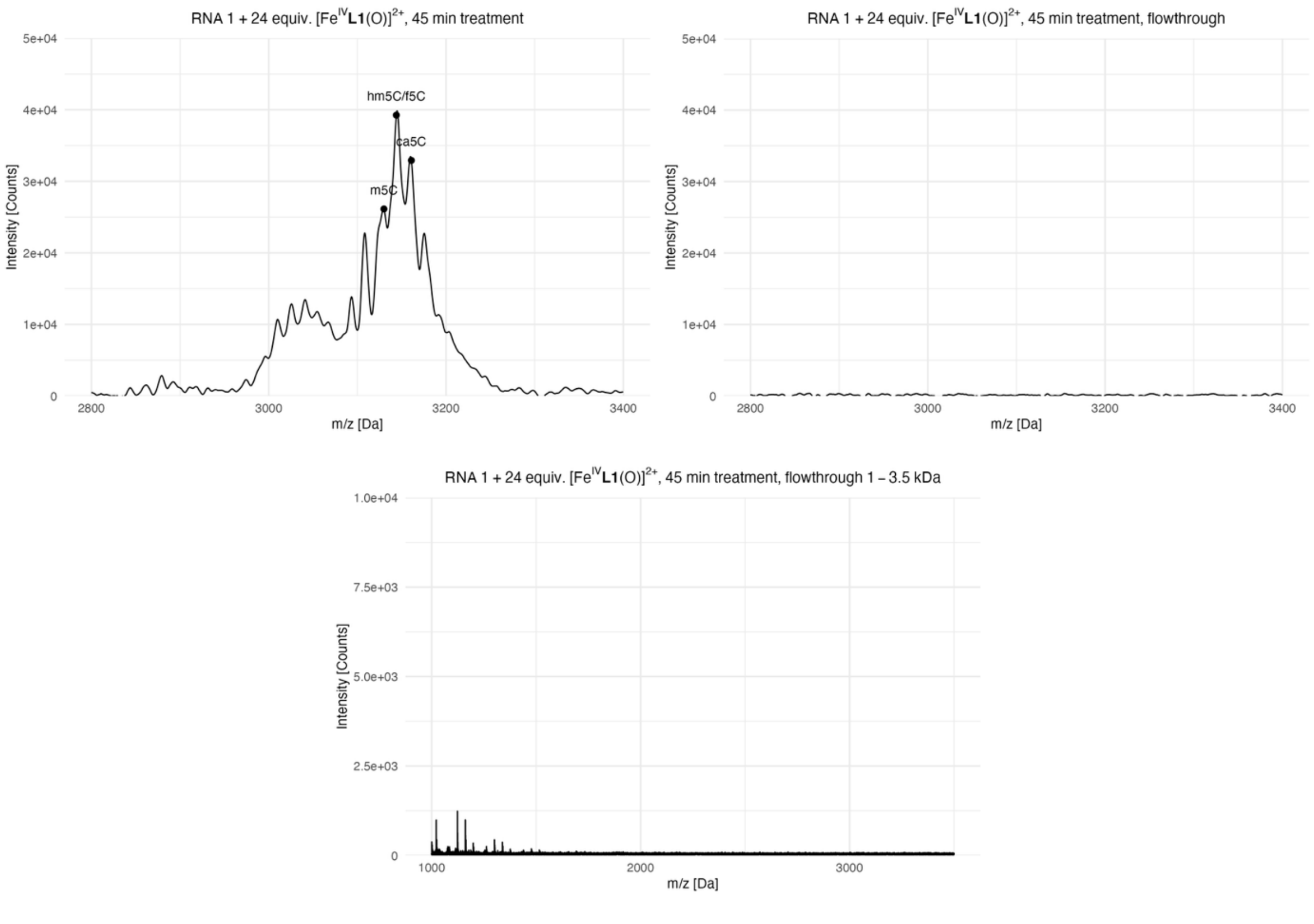

Figure S2: Centrifugal filtration is performed to remove the iron species from the reaction mixture to stop the oxidation process. For the condition 24 equivalents $[Fe^{IV}\mathbf{L1}(O)]^{2+}$ and 60 min reaction time with RNA **1**, the flowthrough was collected, lyophilised and analysed by MALDI-MS. As a comparison, the MALDI-MS spectrum of the sample remaining within the filter was measured at working concentration. MALDI-MS was measured both in positive and negative mode, above data only shows the results for negative mode. **Top**: The sample measured at working conditions shows the expected RNA **1** and oxidised product peaks at relatively high intensities up to 40 000, as well as decomposition products at lower intensities. **Middle**: the flowthrough of the same sample was analysed after lyophilisation and rehydration, it shows only background noise where peaks would be expected if RNA **1** was present, measurement mode optimised for 2 - 10 kDa. **Bottom**: The flowthrough was measured and only background noise was observed, measurement mode optimised for 0.6 - 3.5 kDa. Measurements were performed in duplicates, and one representative sample spectrum is shown for duplicate samples.

HPLC MALDI Peak Spectra

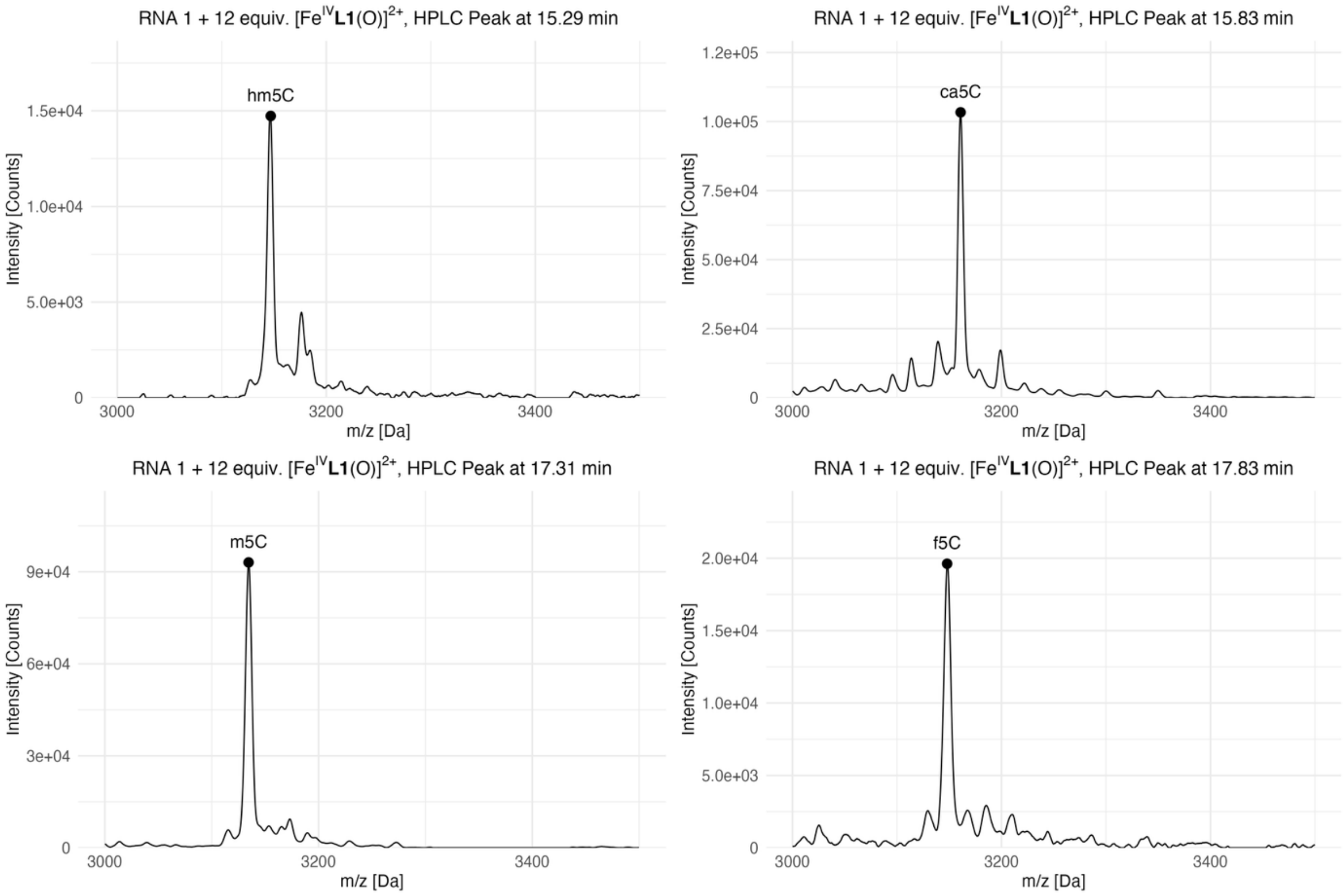

Figure S3: The reactions were scaled up 10x and run in a 500 µL volume with 12 equivalents of $[Fe^{IV}L1(O)]^{2+}$. After removal of the iron species by filtration, the samples were 25x concentrated by lyophilisation and redissolved in MQ water. The samples

were then subjected to analytical HPLC, the major peaks collected and their identity verified by MALDI-MS in negative measurement mode. This acted as an additional control for strand intactness of oxidised RNA 1

products. All three m5C oxidation products could be found incorporated into the intact RNA 1 strand.

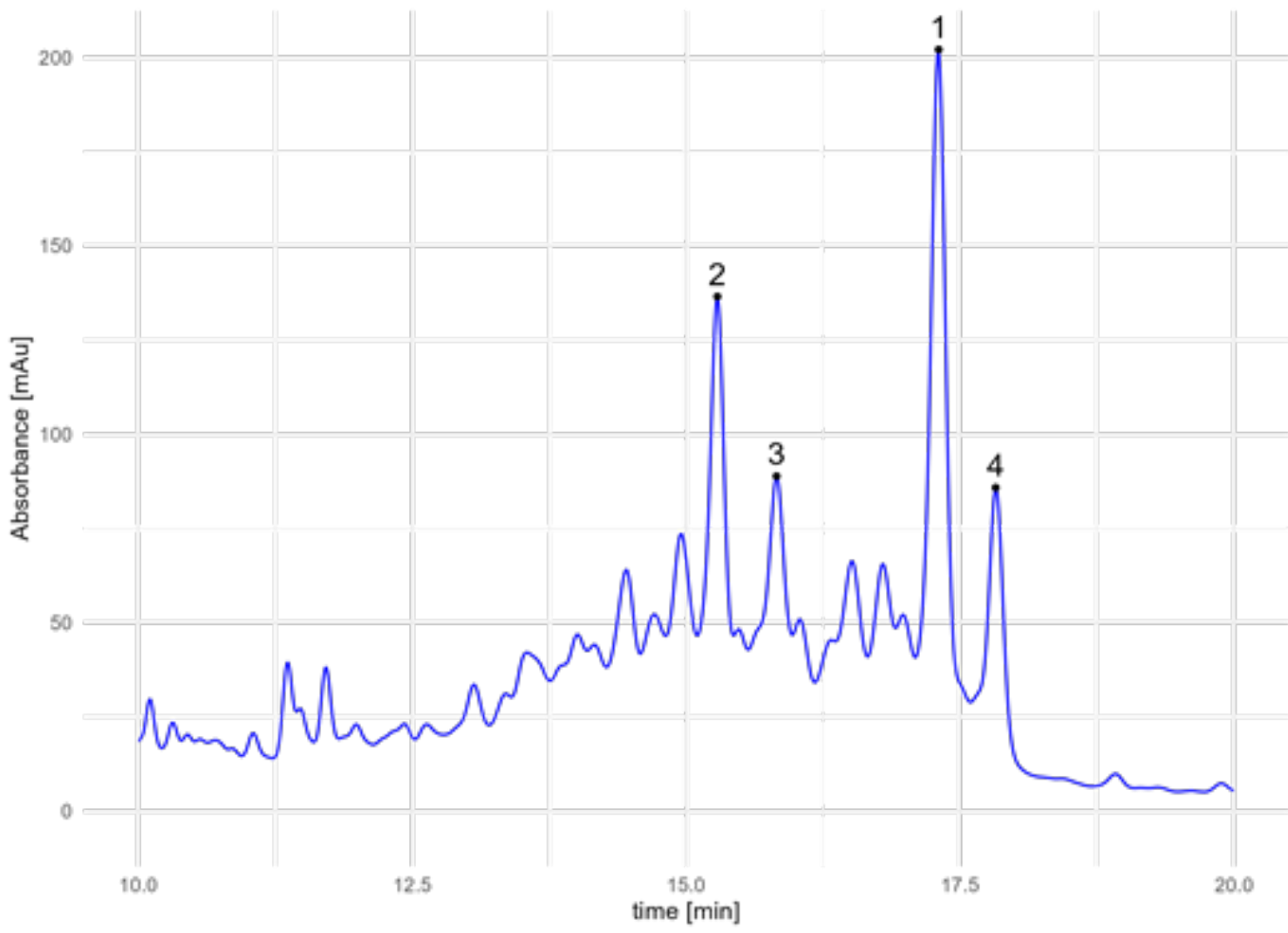

Peak	Runtime [min]	RNA **1** with
1	17.31	m5C
2	15.29	f5C/hm5C
3	15.83	c5C
4	17.83	f5C/hm5C

Figure S4: The reactions were scaled up 10x and run in a 500 µL volume with 12 equivalents of $[Fe^{IV}L1(O)]^{2+}$. After removal of the iron species by filtration, the samples were 25x concentrated by lyophilisation and redissolved in MQ water. The samples were then subjected to analytical HPLC and the major peaks collected. The obtained HPLC spectrum is depicted and the highest intensity peaks 1-4 could be assigned to the respective oxidation products of RNA **1** with high confidence *via* MALDI-MS. A zoom in on 10-20 min HPLC trace of $[Fe^{IV}L1(O)]^{2+}$ reacted RNA **1** (12 equivalents, 60 min) is shown. Run settings: gradient from 8-16% B over 35 min, 60 °C. The HPLC peak heights cannot be interpreted quantitatively.

UHPLC-MS/MS (QQQ) for Nucleoside-Quantification

Oxidation efficiency was determined by quantitative UHPLC-MS/MS (Triple Quadrupole LC/MS, QQQ) analysis of digested DNA/ODN samples using an Agilent 1290 Infinity II UHPLC system equipped with a variable wavelength detector and an Agilent Technologies 6490 triple quadrupole mass spectrometer. Chromatography was performed with an InfinityLab Poroshell 120 SB-C18 column (Agilent, 2.7 µm, 2.1 mm × 150 mm) at 35 °C using a gradient of water and MeCN, each containing 0.0075% (v/v) formic acid, at a flow rate of 0.35 mL/min: 0–2.5 min: 0% MeCN; 2.5–5.0 min: 0%→1.5% (v/v) MeCN; 5.5–7.0 min: 1.5%→5.0% MeCN; 7.0–7.5 min: 5.0%→80% MeCN; 7.5–9.5 min: 80% MeCN; 9.5–10.0 min: 80→0% MeCN; 10.0–13.0 min: 0% MeCN. The autosampler was cooled to 4 °C and the injection volume was set to 10 µL for all samples. The nucleosides of interest were quantified using an adjusted version of the previously reported “stable isotope dilution technique”.[1] The source-dependent parameters were set as follows: gas temperature 120 °C, gas flow 11 L/min (N_2), nebulizer 60 psi, sheath gas temperature 280 °C, sheath gas flow 11 L/min (N_2), capillary voltage 3000 V in positive ion mode, nozzle voltage 500 V, high-pressure RF at 150 V and low-pressure RF at 60 V. The compound-dependent parameters are summarized Supporting Table S3. For all compounds, the fragmentor voltage was set to 380 V and the Electron multiplier voltage was set to 500 V (positive mode). Each sample was spiked with 1 µL of a stable isotope-labeled internal standard (ISTD) mix containing the isotope standards stated in Supporting Information, Table 3. The sample data were analyzed by Quantitative MassHunter Software (Agilent, Version B07.01) using the built-in calibration function. The results of Quantification are summarized in Supporting Information, Table 3.

Table S3: Compound-dependent LC-MS/MS-parameters. CE: collision energy; CAV: collision cell accelerator voltage, t_R: retention time.

Compound	Precursor Ion [m/z]	MS1 Resolution	Product Ion [m/z]	MS2 Resolution	t_R [min]	Δt_R [min]	Fragmentor	CE [V]	CAV [V]	Polarity
C	244.0	Wide	112.1	Unit	1.82	3	380	8	5	Positive
[$^{13}C_9$]-C ISTD	253.0	Wide	116.1	Unit	1.82	3	380	8	5	Positive
hm5C	274.1	Wide	142.0	Unit	2.07	3	380	8	5	Positive
[$^{15}N_3$]-hm5C ISTD	277.1	Wide	145.0	Unit	2.07	3	380	8	5	Positive
m5C	258.1	Wide	126.1	Unit	2.81	3	380	10	5	Positive
[D_3]-m5C ISTD	261.1	Wide	129.1	Unit	2.81	3	380	10	5	Positive
ca5C	288.0	Wide	156.0	Unit	3.09	3	380	10	5	Positive
[$^{15}N_3$]-ca5C ISTD	291.0	Wide	159.0	Unit	3.09	3	380	10	5	Positive
U	245.1	Wide	113.1	Unit	3.8	3	380	5	5	Positive

[$^{13}C_9$]-U ISTD	254.1	Wide	117.1	Unit	3.8	3	380	5	5	Positive
G	284.1	Wide	152.1	Unit	7.87	4	380	10	5	Positive
[$^{13}C_{10}$]-G ISTD	294.1	Wide	157.1	Unit	7.87	4	380	10	5	Positive
f5C	272.0	Wide	140.0	Unit	8.2	3	380	10	5	Positive
[$^{15}N_3$]-f5C ISTD	275.0	Wide	143.0	Unit	8.2	3	380	10	5	Positive
A	268.1	Wide	136.1	Unit	8.62	5	380	16	5	Positive
[$^{13}C_{10}$]-A ISTD	278.1	Wide	141.1	Unit	8.62	5	380	16	5	Positive

Sample preparation and reaction in presence of formamide, for UHPLC-MS/MS

Formamide (16 μL) was added to RNA-oligo (5 μM, 4 μL) in water on ice. Samples were then heated to 85 °C for 8 min, and immediately transferred on ice again. [$Fe^{IV}L1(O)]^{2+}$ (1 mM, equivalents 12- 24 eq) and water were added to give a final volume of 50 μL. Samples were incubated (300 rpm) for 60 min. Reactions were quenched by placement on ice and by removal of [FeIVL1(O)]$^{2+}$ from the sample mixture by transfer of the reaction volume to centrifugal spin columns (Amicon® Ultra Centrifugal Filter, 3 kDa MWCO, Cat. UFC5003, Millipore) and addition of 400 μL of water before centrifugation (14,000 g, 20 min, 4 °C). Three centrifugation steps with addition of 400 μL water between centrifugation steps were performed to achieve [FeIVL1(O)]$^{2+}$ removal. After the last centrifugation step, sample identity was confirmed by MALDI and the remaining sample volume was transferred to an Eppendorf tube and samples were frozen at –20 °C until further processing. All reactions were performed in duplicate.

Supporting Data QQQ

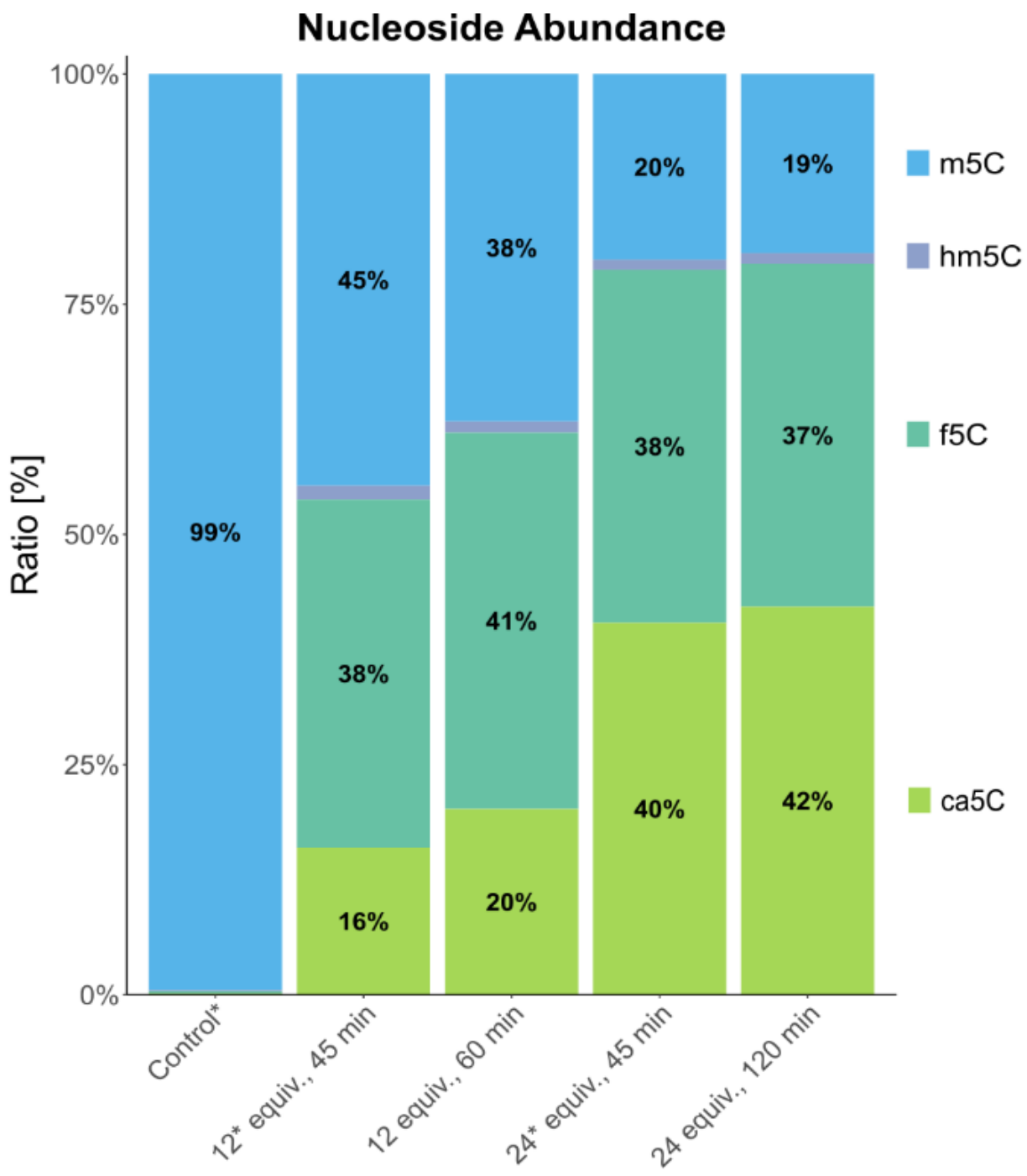

Figure S5: To find optimal reaction conditions, an investigation into oxidation product ratios along increasing reaction times was performed. RNA **1** was reacted with 12 equivalents $[Fe^{IV}\mathbf{L1}(O)]^{2+}$ for up to 120 min. Strand intactness was controlled for by MALDI-TOF-MS before digestion of RNA **1** to single nucleotide level, and analysis via UHPLC-MS/MS (QQQ, Table S8). Data collected on two different days is indicated by *.

Raw data used for plotting of UHPLC-MS/MS results

Table S4: Raw data for Figure 5A

**Sample**	**m5C/rN [×10²]**	**hm5C/rN [×10²]**	**f5C/rN [×10²]**	**ca5C/rN [×10²]**		**m5C [%]**	**hm5C [%]**	**f5C [%]**	**ca5C [%]**
Control*	9.34 ± 0.96	0.01 ± 0.01	0.03 ± 0.01	0.03 ± 0.01		99.16 ± 0.16	0.12 ± 0.10	0.35 ± 0.04	0.37 ± 0.10
8 equiv.	6.00 ± 0.43	0.18 ± 0.01	2.28 ± 0.16	0.45 ± 0.00		67.37 ± 0.31	2.00 ± 0.01	25.57 ± 0.05	5.06 ± 0.35
10 equiv.	6.21 ± 0.34	0.18 ± 0.01	2.51 ± 0.39	0.59 ± 0.00		65.46 ± 3.98	1.91 ± 0.04	26.42 ± 4.01	6.21 ± 0.07
12* equiv.	4.99 ± 0.31	0.17 ± 0.01	3.80 ± 0.56	1.37 ± 0.42		48.57 ± 5.51	1.67 ± 0.22	36.65 ± 2.68	13.11 ± 3.06
14* equiv.	4.60 ± 0.39	0.17 ± 0.02	4.17 ± 0.68	1.82 ± 0.56		43.13 ± 6.95	1.61 ± 0.26	38.56 ± 3.34	16.70 ± 3.90
16 equiv.	3.99 ± 0.13	0.15 ± 0.00	4.62 ± 0.09	2.52 ± 0.32		35.39 ± 2.02	1.37 ± 0.04	40.92 ± 0.24	22.32 ± 2.29
18 equiv.	3.49 ± 0.16	0.14 ± 0.01	4.77 ± 0.04	3.04 ± 0.05		30.49 ± 1.00	1.24 ± 0.07	41.71 ± 0.14	26.56 ± 0.78
20 equiv.	3.56 ± 0.02	0.15 ± 0.01	5.11 ± 0.16	3.31 ± 0.14		29.39 ± 0.84	1.21 ± 0.09	42.12 ± 0.42	27.28 ± 0.52
24 equiv.	3.22 ± 0.15	0.14 ± 0.00	5.09 ± 0.07	3.91 ± 0.08		26.08 ± 1.19	1.10 ± 0.00	41.19 ± 0.54	31.63 ± 0.65

Table S5: Raw data for Figure 5B

Sample	m5C/rN [×10^2]	hm5C/rN [×10^2]	f5C/rN [×10^2]	ca5C/rN [×10^2]		m5C [%]	hm5C [%]	f5C [%]	ca5C [%]
Control*	9.34 ± 0.96	0.01 ± 0.01	0.03 ± 0.01	0.03 ± 0.01		99.16 ± 0.16	0.12 ± 0.10	0.35 ± 0.04	0.37 ± 0.10
5 min	7.46 ± 0.16	0.25 ± 0.01	0.98 ± 0.22	0.11 ± 0.03		84.72 ± 2.75	2.88 ± 0.12	11.15 ± 2.34	1.25 ± 0.30
10 min	6.70 ± 0.32	0.30 ± 0.09	1.84 ± 0.23	0.44 ± 0.23		72.31 ± 3.36	3.15 ± 0.65	19.84 ± 0.66	4.69 ± 2.05
20 min*	5.76 ± 1.10	0.19 ± 0.02	3.39 ± 1.29	1.05 ± 0.56		56.18 ± 14.67	1.89 ± 0.34	32.12 ± 10.41	9.80 ± 4.70
30 min*	4.99 ± 0.31	0.17 ± 0.01	3.80 ± 0.56	1.37 ± 0.42		48.57 ± 5.51	1.67 ± 0.22	36.65 ± 2.68	13.11 ± 3.06
45 min	4.85 ± 0.16	0.15 ± 0.01	4.13 ± 0.18	2.05 ± 0.04		43.34 ± 1.28	1.34 ± 0.07	36.98 ± 1.68	18.33 ± 0.32
60 min	4.31 ± 0.05	0.14 ± 0.00	4.67 ± 0.24	2.30 ± 0.09		37.76 ± 0.80	1.19 ± 0.04	40.87 ± 0.76	20.18 ± 0.08

Table S6: Raw data for Figure 6

Sample	m5C/rN [×10^2]	hm5C/rN [×10^2]	f5C/rN [×10^2]	ca5C/rN [×10^2]		m5C [%]	hm5C [%]	f5C [%]	ca5C [%]
RNA2, con-trol	9.79 ± NA	0.04 ± NA	0.04 ± NA	0.03 ± NA		98.87 ± NA	0.38 ± NA	0.41 ± NA	0.34 ± NA
RNA3, con-trol	15.44 ± NA	0.01 ± NA	0.01 ± NA	0.01 ± NA		99.75 ± NA	0.08 ± NA	0.09 ± NA	0.07 ± NA
RNA4, con-trol	11.08 ± NA	0.09 ± NA	0.10 ± NA	0.09 ± NA		97.55 ± NA	0.81 ± NA	0.89 ± NA	0.75 ± NA
RNA2	8.92 ± 0.28	0.29 ± 0.01	1.09 ± 0.04	0.21 ± 0.01		84.85 ± 0.22	2.81 ± 0.08	10.38 ± 0.06	1.97 ± 0.16
RNA3	14.93 ± 0.46	0.40 ± 0.02	1.75 ± 0.08	0.26 ± 0.01		86.11 ± 0.13	2.32 ± 0.02	10.06 ± 0.12	1.51 ± 0.01
RNA4	10.22 ± 0.55	0.33 ± 0.02	1.67 ± 0.16	0.33 ± 0.02		81.46 ± 1.15	2.60 ± 0.21	13.33 ± 0.90	2.61 ± 0.19

Table S7: Raw data for Figure 7

Sample	m5C/rN [×10^2]	hm5C/rN [×10^2]	f5C/rN [×10^2]	ca5C/rN [×10^2]		m5C [%]	hm5C [%]	f5C [%]	ca5C [%]
12 equiv., L2	3.73 ± 0.25	0.13 ± 0.01	3.44 ± 0.38	3.31 ± 0.45		35.33 ± 4.26	1.22 ± 0.04	32.37 ± 1.88	31.08 ± 2.51
18 equiv., L2	1.99 ± 0.03	0.16 ± 0.04	3.32 ± 0.02	6.22 ± 0.11		17.04 ± 0.05	1.39 ± 0.31	28.39 ± 0.10	53.19 ± 0.36
24 equiv., L2	1.53 ± 0.05	0.13 ± 0.02	3.25 ± 0.14	8.82 ± 0.25		11.13 ± 0.31	0.93 ± 0.14	23.65 ± 0.58	64.29 ± 0.97
12 equiv., L1	4.31 ± 0.05	0.14 ± 0.00	4.67 ± 0.24	2.30 ± 0.09		37.76 ± 0.80	1.19 ± 0.04	40.87 ± 0.76	20.18 ± 0.08
18 equiv., L1	2.71 ± 0.78	0.16 ± 0.04	4.56 ± 0.30	3.92 ± 0.80		23.65 ± 3.02	1.44 ± 0.63	40.48 ± 3.88	34.43 ± 1.49

Table S8: Raw data for Figure S5

Sample	m5C/rN [×10^2]	hm5C/rN [×10^2]	f5C/rN [×10^2]	ca5C/rN [×10^2]		m5C [%]	hm5C [%]	f5C [%]	ca5C [%]
Control*	9.18 ± NA	0.02 ± NA	0.02 ± NA	0.01 ± NA		99.47 ± NA	0.17 ± NA	0.21 ± NA	0.15 ± NA
12* equiv., 45 min	4.72 ± 0.14	0.16 ± 0.01	3.99 ± 0.16	1.69 ± 0.33		44.78 ± 1.49	1.51 ± 0.16	37.83 ± 1.18	15.88 ± 2.27
12 equiv., 60 min	4.31 ± 0.05	0.14 ± 0.00	4.67 ± 0.24	2.30 ± 0.09		37.76 ± 0.80	1.19 ± 0.04	40.87 ± 0.76	20.18 ± 0.08
24* equiv., 45 min	2.44 ± 0.05	0.13 ± 0.02	4.63 ± 0.23	4.88 ± 0.43		20.21 ± 1.04	1.11 ± 0.21	38.33 ± 0.32	40.35 ± 1.44

Materials and Methods

General Considerations

$[Fe^{IV}\mathbf{L_x}(O)]^{2+}$ refers to either $[Fe^{IV}(O)\mathbf{L1}]^{2+}$ or $[Fe^{IV}(O)\mathbf{L2}]^{2+}$, as described by Jonasson.[10]

Water refers to ultrapure water (type 1, pH 5.6, 18.2 MΩ * cm at 25 °C) obtained from either a Stakpure (Niederahr, Germany) or MilliQ (Merck, Darmstadt, Germany) device.

All experiments involving RNA **1**-**4** were performed using centrifugal tubes (1.5 mL or 0.5 mL. Eppendorf, Hamburg, Germany) whilst working under sterile conditions next to a bunsenburner and using autoclaved pipette tips (Brand, Wertheim, Germany) and tubes only. Water used in RNA experiments was always left running for 30 s before collection in a 50 mL falcon tube (Greiner Bio-One, Kremsmünster, Austria).

If not otherwise specified, reactions are performed at room temperature (20-25 °C).

Preparation of $[Fe^{IV}\mathbf{L_x}(O)]^{2+}$ (1 mM) with L1 or L2 ligand system

A 10 mM stock solution of $[Fe^{IV}\mathbf{L_x}(O)]^{2+}$ was prepared according to a modified literature procedure and stored on ice.[10]

For an activity test prior to use, $[Fe^{IV}\mathbf{L1}(O)]^{2+}$ (10 mM, 20 μL) was diluted with water (60 μL) before addition of benzylalcohol (20 μL). Upon addition of benzylalcohol, the solution turned yellow within the next seconds to minute to indicate complex activity. If no yellow colouring was visually observed, the solution was discarded. Following the activity test, the 10 mM stock solution was diluted with water to obtain the 1 mM stock solution and stored on ice.

If specified, an aliquot of $[Fe^{IV}\mathbf{L_x}(O)]^{2+}$ (1 mM, 1 mL) was taken and a UV-Vis spectrum (190 nm - 1100 nm, 1 nm scanning interval) was recorded at the time of the reaction start or shortly after on a Genesys 10S UV-visible Spectrophometer (Thermo Scientific, Darmstadt, Germany) using a quartz cuvette, blanking against water. Spectra were automatically baseline subtracted, exported as .csv and absorption values were graphically determined at 720 nm, respectively 730 nm (Figure S7).

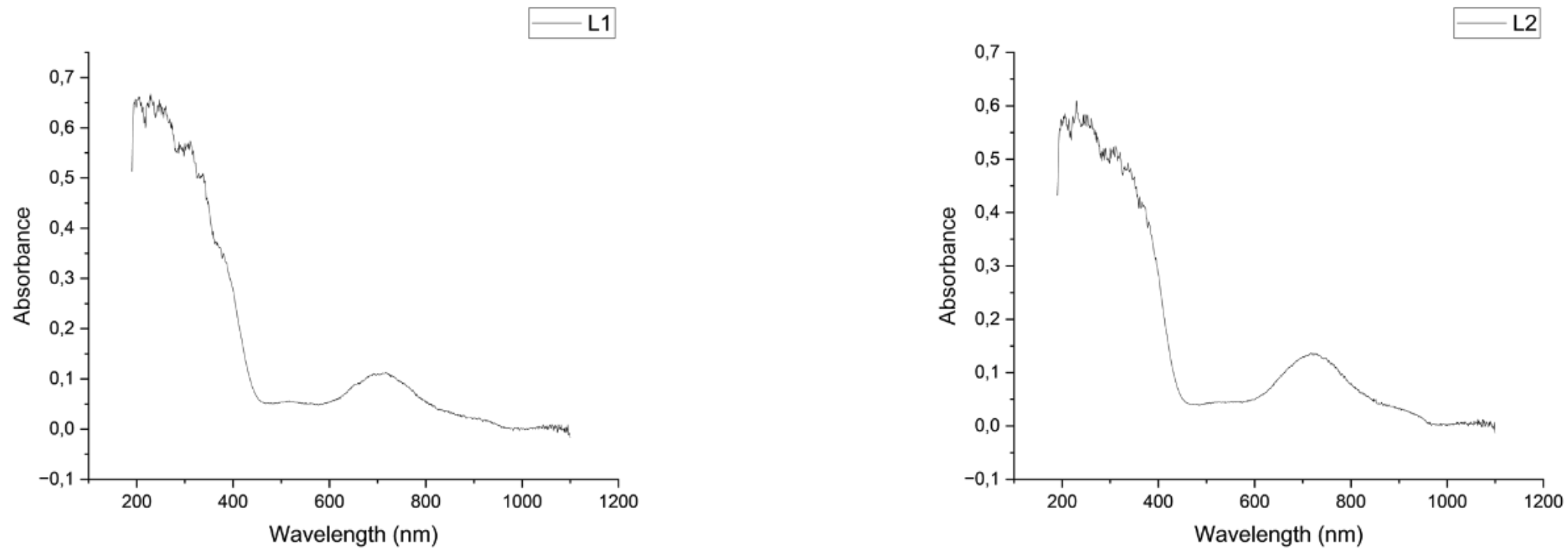

Figure S6: UV-vis spectra of $[Fe^{IV}\mathbf{L_x}(O)]^{2+}$ (1 mM, 1 mL) were recorded at reaction start and complex integrity evaluated via the 720 nm ($[Fe^{IV}\mathbf{L_1}(O)]^{2+}$, left), respectively 730 nm ($[Fe^{IV}\mathbf{L_2}(O)]^{2+}$, right) and compared to the $[Fe^{II}\mathbf{L_x}(OH_2)]^{2+}$ absorption of the Fe(II) species.[11]

RNA Oligomers

Lyophilized RNA-Oligo RNA **1** (100 - 200 nmol) was custom ordered from Ella Biotech (Fürstenfeldbruck, Germany) with incorporation of the 5-Me-C-TOM-CE Phosphoramidite (Glen Research, Sterling, USA, 100 μmol). Lyophilized RNA-Oligo RNA **1** (5'-AUUG(**m5C**)CACUG-3', Ella Biotech, Fürstenfeldbruck, Germany, 0.5 mM) was dissolved in water according to manufacturer's instructions and stored at -20 °C.

Enzymatic RNA Synthesis

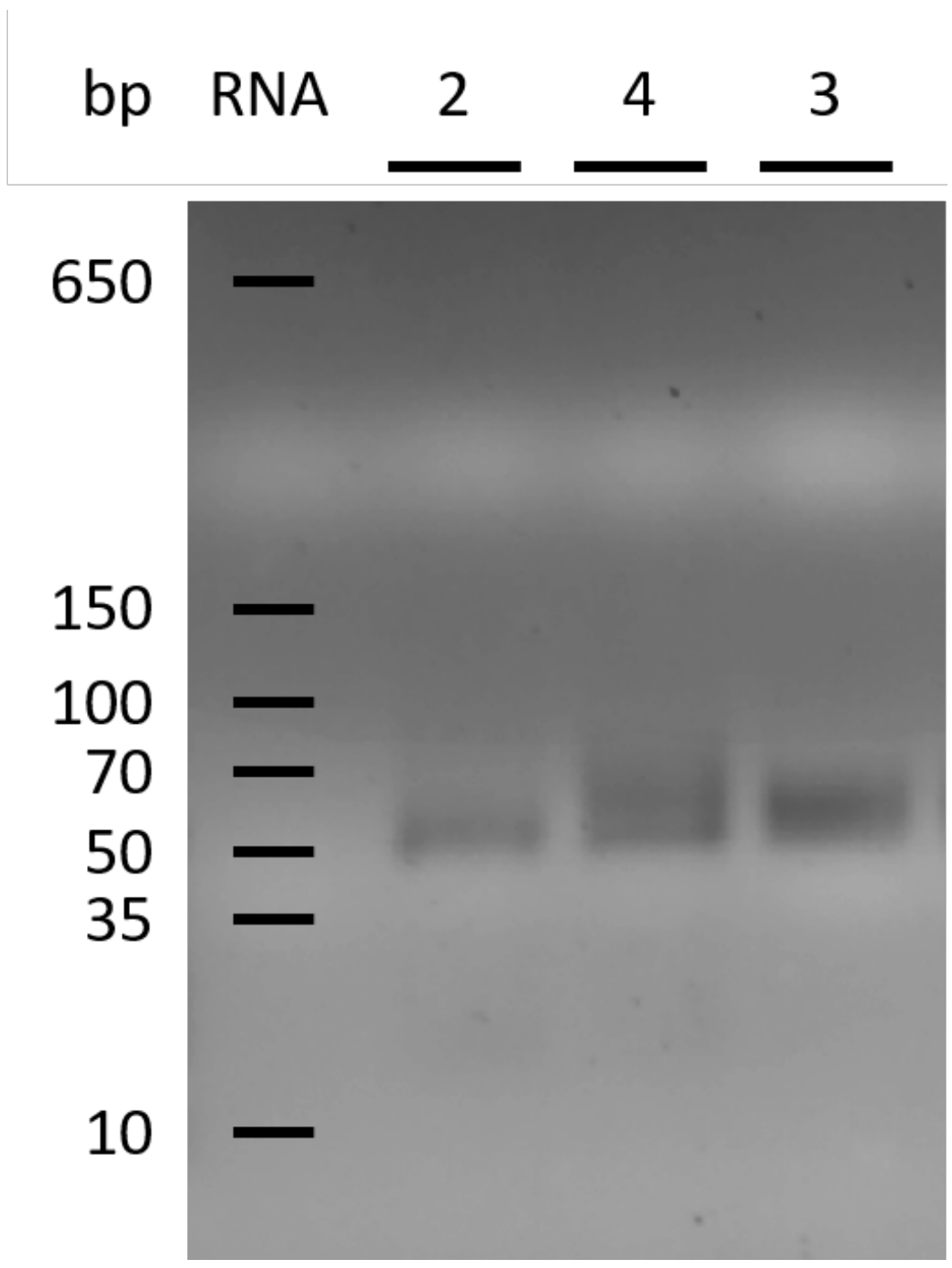

Figure S7: RNA **2** (185.56 μmol/L), RNA **4** (262.49 μmol/L), and RNA **3** (236.24 μmol/L) were prepared by in vitro transcription.

Table S9: Sequences of RNA **1-4**.

RNA Oligomer	Sequence
RNA **1**	5-AUUG(**m5C**)CACUG-3
RNA **2**	5‘-GAUUAUAAUAUUAUAAUUAUAUUAAGGU[**m5C**]UGGUUAAUAUAAU-UAUAAUAUUAUAAU-3‘
RNA **3**	5‘-GUUUAAUAAGUUUUUUUUUUUUUUUGGUGGUAAAAAAAAAAAAAA-A[**m5C**]UUAUUAAA-3‘
RNA **4**	5‘-GAUUAUAAUGUUAUAAUUAUAUUAUAUUAAGGUGUGGUUAAUAUA-AU-UAUAA[**m5C**]AUUAUAAU-3‘

In vitro transcription of RNA substrates

Template DNA encoding for the sequences of RNA **2-4** and a 5’ T7 RNA polymerase promoter sequence, were produced via polymerase chain reaction (Q5® High-Fidelity DNA Polymerase, New England Biolabs,New England Biolabs, Ipswitch, USA). For the *in vitro* transcription the reaction was set up in a 100 µL reaction mixture containing template DNA (75 pmol), HEPES (40 mM, pH 8.0), DTT (5 mM), Triton X-100 (0.05%), $MgCl_2$ (32 mM), ATP (3.75 mM), GTP (3.75 mM), UTP (3.75 mM), 5-methyl-CTP (2.5 mM), RNase inhibitor NEB (800 U), self-produced T7 polymerase (8 µL) were added and incubated for 16 hours at 37 °C. To remove template DNA, DNase I-XT (2 µL) was added and incubated at 37°C for 2 h. The RNA products were then purified (Monarch RNA Cleanup Kit, New England Biolabs, Ipswitch, USA) and stored at −80 °C. To ensure proper folding, the tRNA was heated to 85 °C and subsequently cooled down to 20 °C over 30 min.

Table S10: Protocol for *in vitro* transcription of RNA substrates

	Temp. [°C]	time	
Initial Melting	98	2 min	
Melting	98	30 s	32x
Annealing	59	20 s	
Extension	72	30 s	
Final Extension	98	2 min	
Incubation	12		

Primer fw (RNA 2-4)	5‘-GATCTAATACGACTCACTATAG-3‘

Primer Rev (RNA 2)	5‘-ATTATAATATTATAATTATATTAACC-3‘
Primer Rev (RNA 4)	5‘-ATTATAATGTTATAATTATATTAACC-3‘
Primer Rev (RNA 3)	5‘-TTTAATAGTTTTTTTTTTTTTTTTTCAC-3‘

Sample preparation and reaction for analysis by mass spectrometry

$[Fe^{IV}\mathbf{L1}(O)]^{2+}$ (1 mM, 8 – 40 equivalents) was added to RNA (50 μM, 10 μL) and water to give a final volume of 50 μL at timepoint t = 0. Samples were incubated (300 rpm) for 5, 10, 20, 30, 45 or 60 min respectively. Reactions were quenched by placement on ice and by removal of $[Fe^{IV}\mathbf{L1}(O)]^{2+}$ from the sample mixture by transfer of the reaction volume to centrifugal spin columns (Amicon® Ultra Centrifugal Filter, 3 kDa MWCO, Millipore, Darmstadt, Germany) and addition of 400 μL of water before centrifugation (14,000 g, 20 min, 4 °C). Three centrifugation steps with addition of 400 μL water between centrifugation steps were performed to achieve $[Fe^{IV}\mathbf{L1}(O)]^{2+}$ removal. After the last centrifugation step, sample identity was confirmed by MALDI-TOF-MS and the remaining sample volume was transferred to an Eppendorf tube and samples were frozen at – 20 °C until further processing. All reactions were performed in duplicate or triplicate.

Sample preparation for reaction with $[Fe^{IV}\mathbf{L2}(O)]^{2+}$ followed the same protocol.

Sample digestion and preparation for UHPLC-MS/MS (QQQ) analysis

Frozen samples were defrosted and 20 μL taken off and transferred to either a centrifugal tube (0.2 mL) or a 96-well plate (VWR PCR plate, Avantor, Darmstadt, Germany). Nucleoside Digestion Mix (New England Biolabs, Frankfurt am Main, Germany) was used for one-step generation of single nucleosides from RNA. To each sample (20 μL), first Nucleoside Digestion Mix Reaction Buffer (10x, 2 μL) was added, then Nucleoside Digestion Mix (1 μL) and the centrifugal tubes or 96 well-plate were incubated (37 °C, 300 rpm, 1 h) on a Thermomixer or Thermostat C (Eppendorf, Hamburg, Germany). A filter plate (Acroprep advance, 0.2 μm, Pall Corporation, Port Washington, USA) was covered with an aluminium adhesive film (Adhesive PCR Sealing Foil Sheets, Thermo, Darmstadt, Germany) and placed on top of a 96-well plate. The filter plate and the 96-wellplate were taped together on one side. After incubation, 20 μL of water was added to the digested samples and the total volume was transferred onto the filter plate by

piercing through the aluminium foil. After centrifugation (2500 g, 40 min, 4°C), the samples were collected from the 96-well plates and transferred to an MS measurement plate.

Complex decomposition with formamide

UV-Vis Spectroscopy_measurements were either conducted with a BioTek Synergy HTX Multimode Reader or an Agilent Cary 3500 Multizone UV-Vis spectrophotometer. Measurements performed on the BioTek Synergy HTX Multimode Reader were carried out in 96-well cell culture plates (Greiner Bio-One, Kremsmünster, Austria) made of sterile, cell culture-treated polystyrene. Each well contained a total volume of 200 µL, and the plate lid was kept on during all measurements. Absorbance at 720 nm was recorded every 4 seconds over a total period of 5 minutes, unless stated otherwise. All experiments were performed in triplicate. Manual pathlength correction was applied after data acquisition. Pathlengths for individual wells were determined by an additional single absorbance measurement at 720 nm following the kinetic run.
Measurements conducted on the Agilent Cary 3500 Multizone UV–Vis spectrophotometer were performed in 1.5 mL semi-micro disposable PMMA cuvettes (Brand GmbH, Wertheim, Germany) with a final sample volume of 1.3 mL. All measurements were referenced during acquisition and manually baseline-corrected afterward. Absorbance at 720 nm was recorded in intervals specified in the corresponding figure legends. All experiments were performed in duplicate.
A 12 M stock solution of formamide was prepared by dissolving 0.956 mL formamide (24.0 mmol) in 1.04 mL MilliQ water.
For kinetic measurements, the concentration of $[Fe^{IV}(O)\mathbf{L1}]^{2+}$ in each well or each cuvette was varied from 120 µM, 500 µM, 1 mM and 2 mM, while the concentration of formamide was maintained at 6 M. In a separate set of experiments, the $[Fe^{IV}(O)\mathbf{L1}]^{2+}$ concentration was held at 500 µM and the formamide concentration increased to 20 M. All concentrations mentioned refer to final concentration in each well (200 µL) or cuvette (1.3 mL), respectively.

NMR Spectroscopy

^1H and ^{13}C NMR spectra were recorded at room temperature on a Bruker Avance III (500 MHz), Bruker Avance III (400 MHz), Varian NMR-System 600 (600 MHz) or Bruker Avance III HD 800 (800 MHz) spectrometer. Raw signals are reported in parts per million (ppm) based on their chemical shift (δ) relative to the internal standard tetramethylsilane (TMS), which is set to 0.00 ppm. For exact calibration of the obtained ^1H-spectra, literature-known shifts of the residual protonated form attributed to the deuterated solvent were used: $CDCl_3$ (δH = 7.26 ppm), CD_2Cl_2 (δH = 5.32 ppm), D_2O (δH = 4.79 ppm), DMSO-d_6 (δH = 2.50 ppm) and Acetone-d_6 (δH = 2.05 ppm). ^{13}C chemical shifts are calibrated with their literature known solvent signals: $CDCl_3$ (δC = 77.16 ppm), CD_2Cl_2 (δC = 53.84 ppm) DMSO-d_6 (δC = 39.52 ppm), Acetone-d_6 (δC = 29.84 ppm).[12]

Coupling constants *J* are reported in Hertz (Hz). In order to assign the ^1H and ^{13}C signals, they are labeled according to the numbers given in the corresponding molecule structure.

All NMR spectra were generated from raw data using MestreNova (version 15.0.0-34764 from Mestrelab Research S.L. Two-dimensional correlation spectroscopy (COSY), heteronuclear single quantum coherence spectroscopy (HSQC), and heteronuclear multiple bond correlation (HMBC) experiments were used to help assign the signals in the spectra.

*O*2',3',5'-Tris(*tert*-butyldimethylsilyl)-cytidine (1)

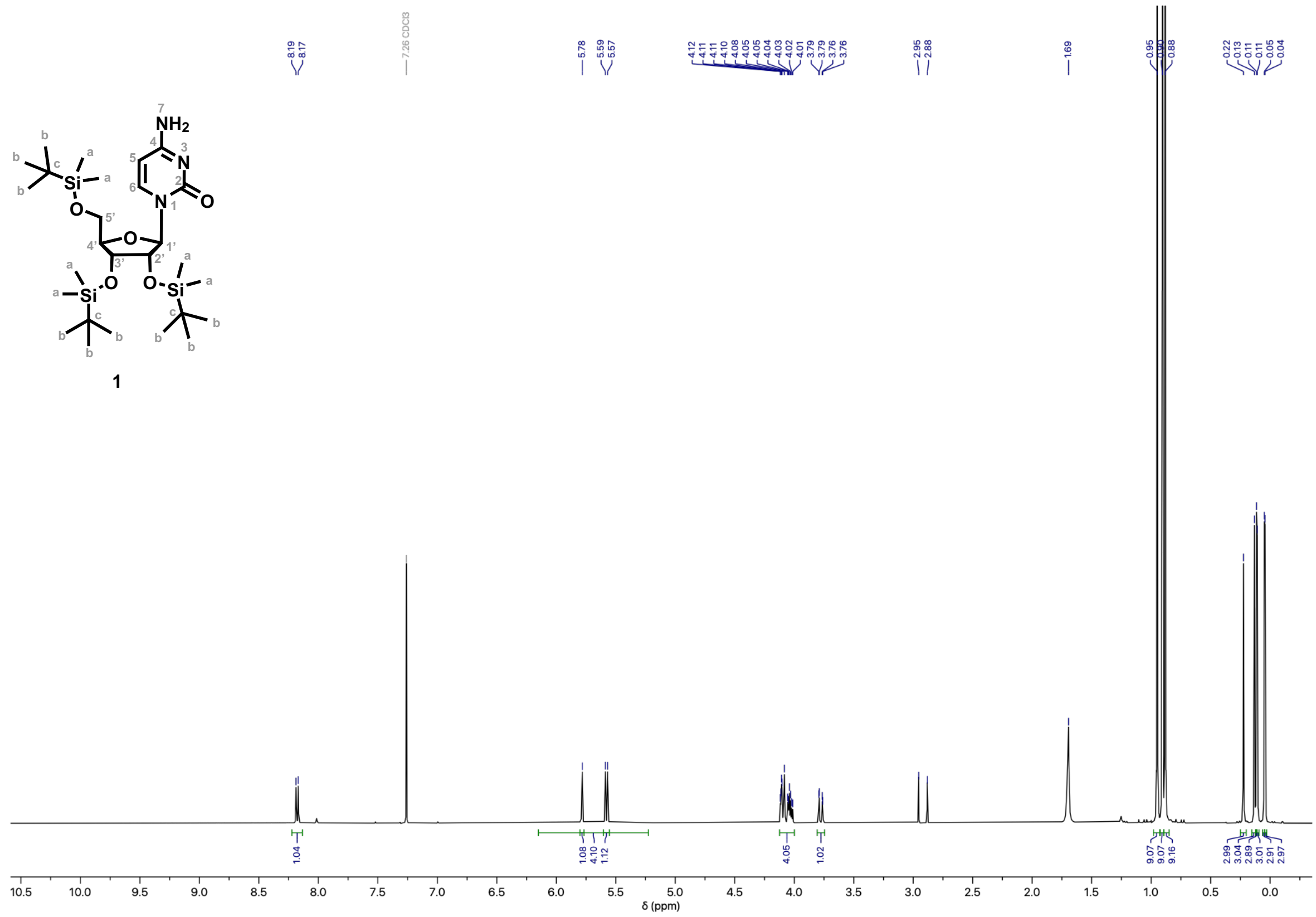

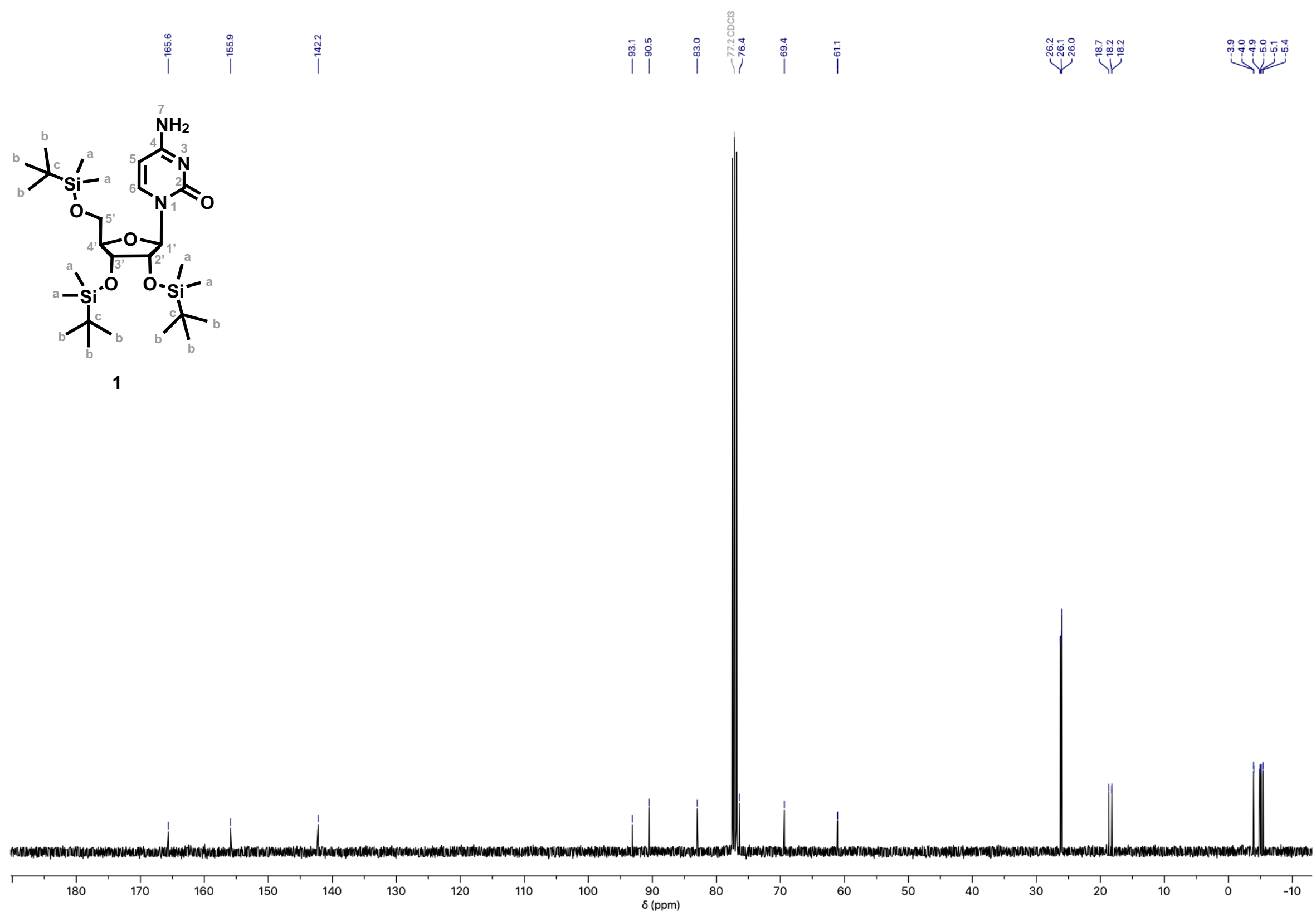

*O*2',3',5'-Tris(*tert*-butyldimethylsilyl)-5-iodo-cytidine (2)

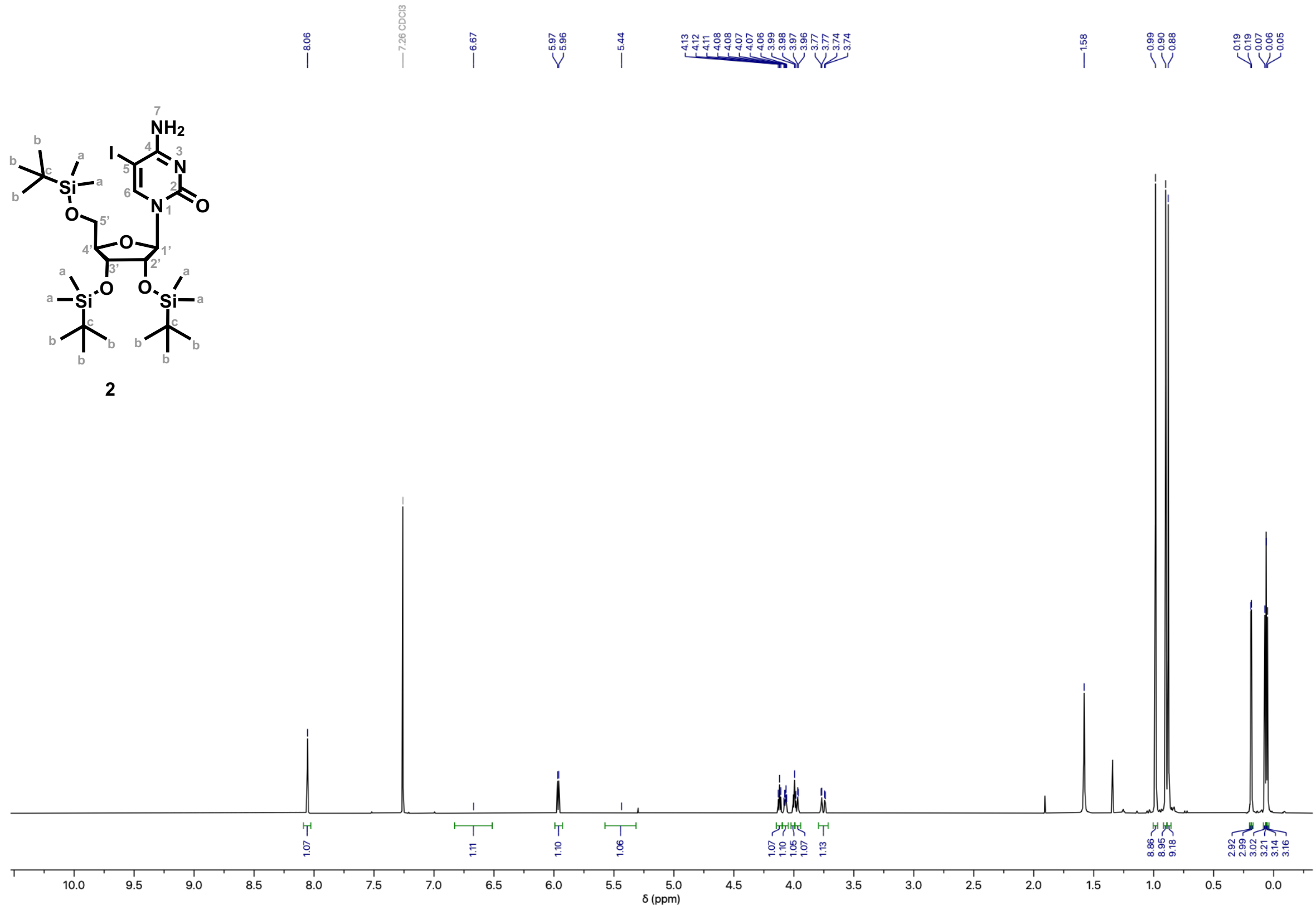

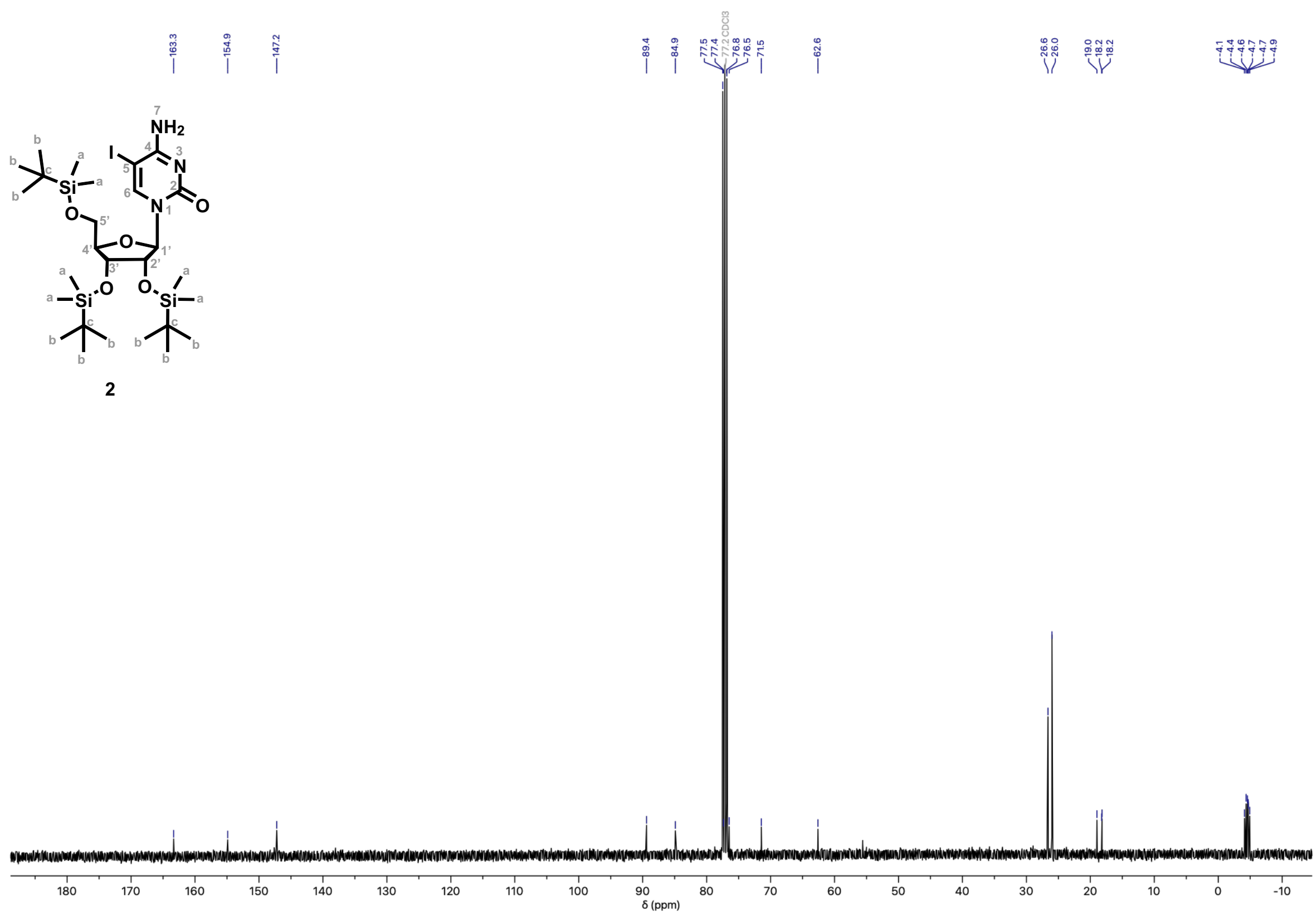

*O*2',3',5'-Tris(*tert*-butyldimethylsilyl)-5-formyl-cytidine (3)

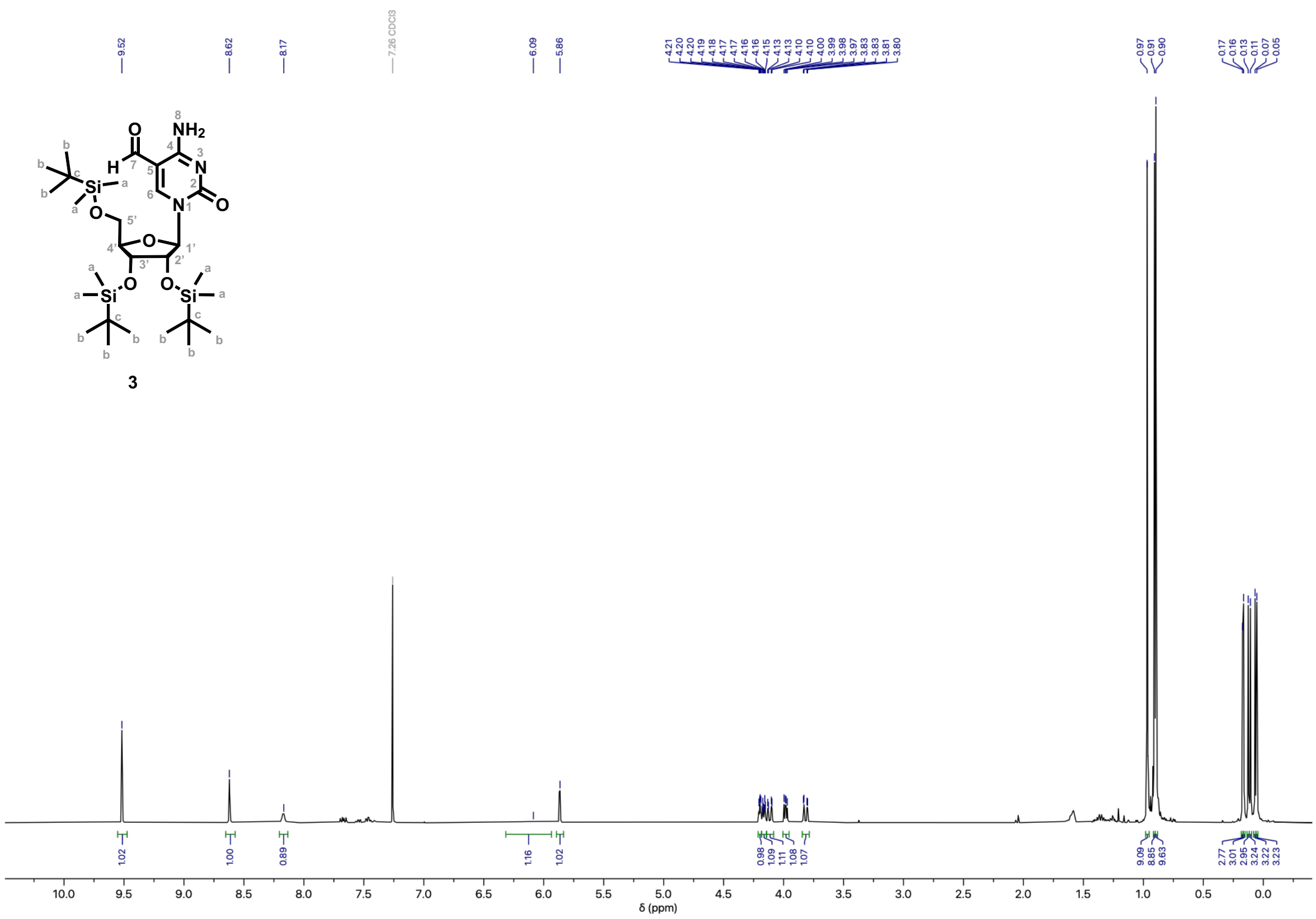

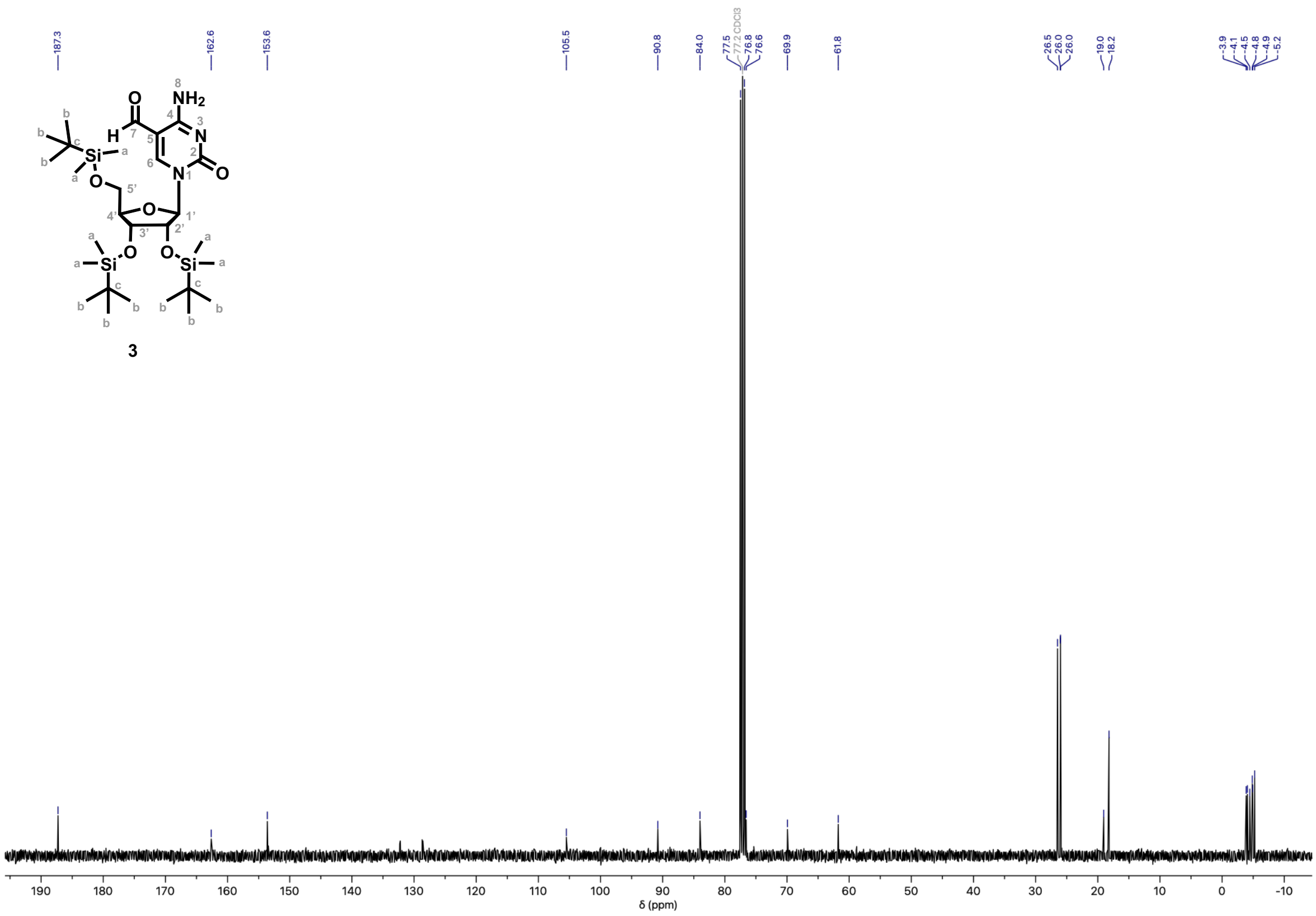

*O*2',3',5'-Tris(*tert*-butyldimethylsilyl)-5-(methoxycarbonyl)-cytidine (4)

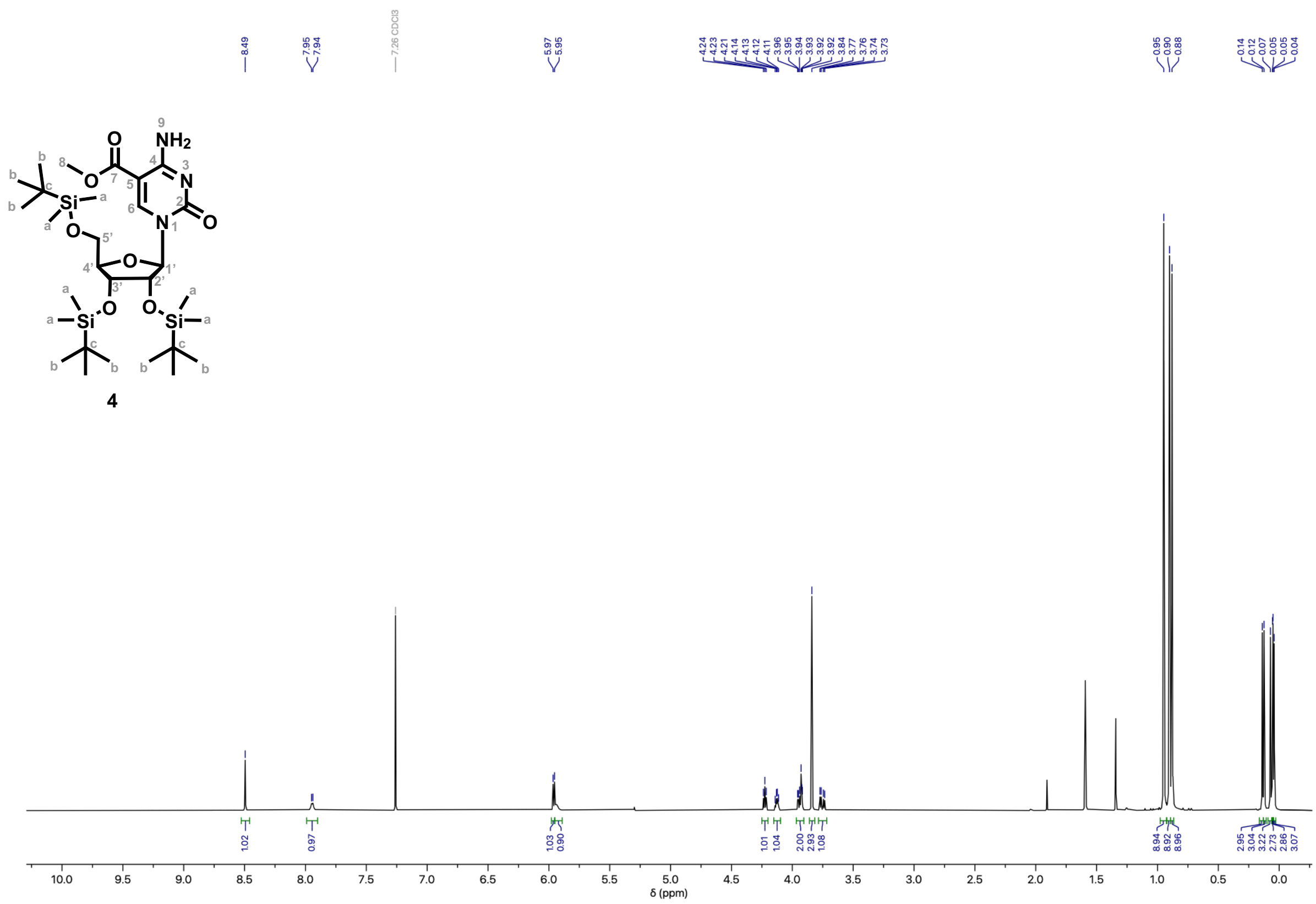

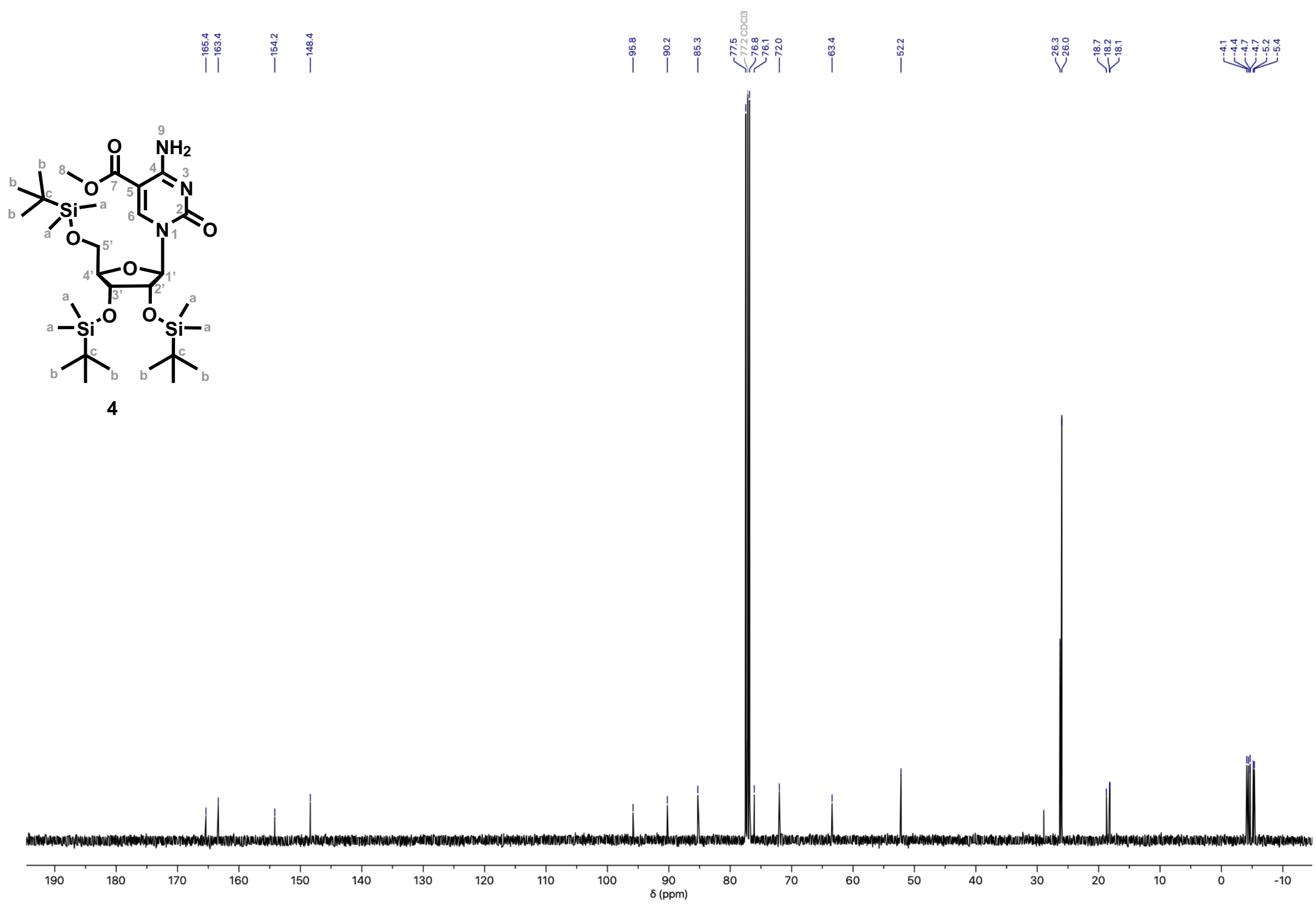

*O*2',3',5'-Tris(*tert*-butyldimethylsilyl)-5-hydroxymethyl-cytidine (5)

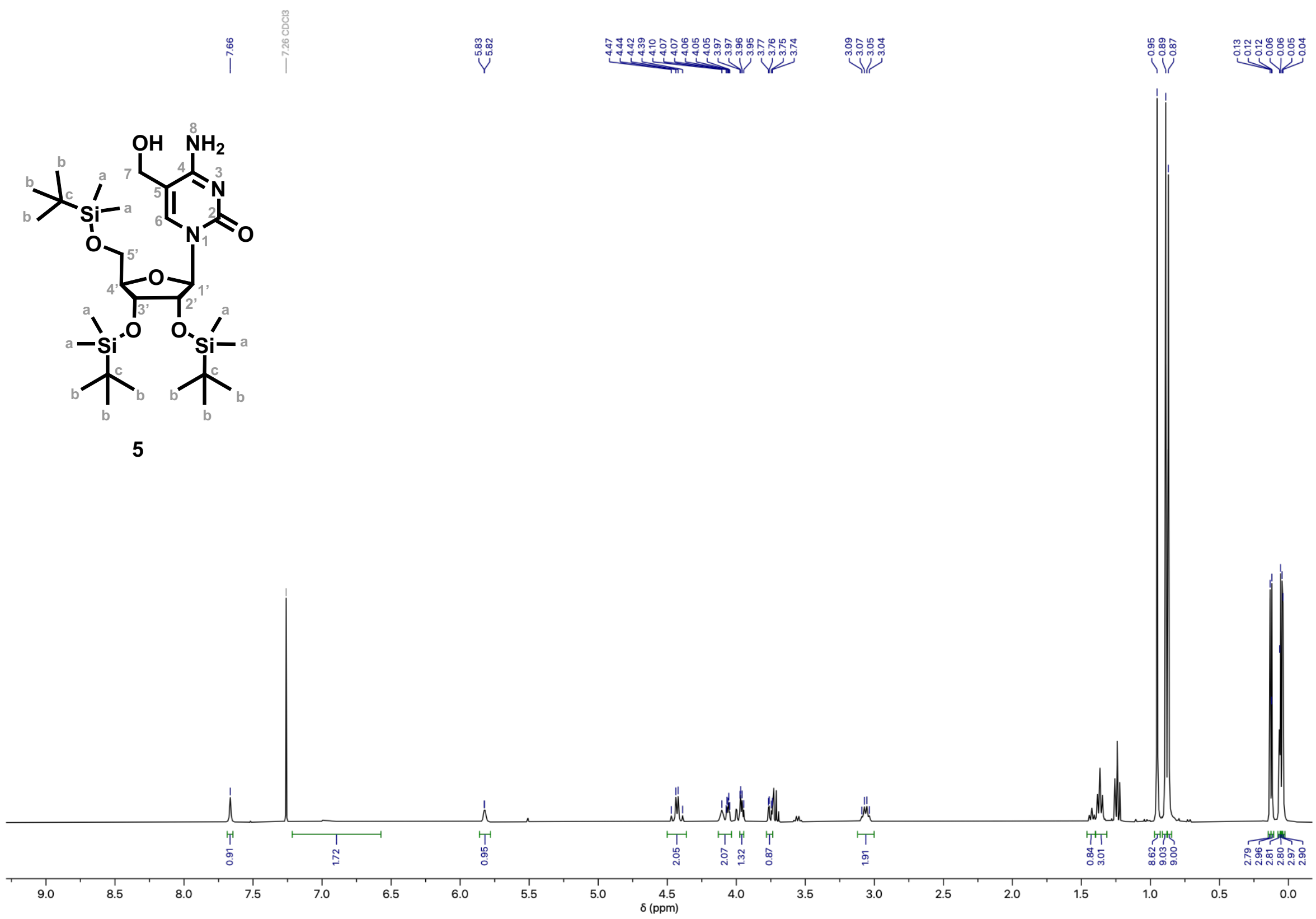

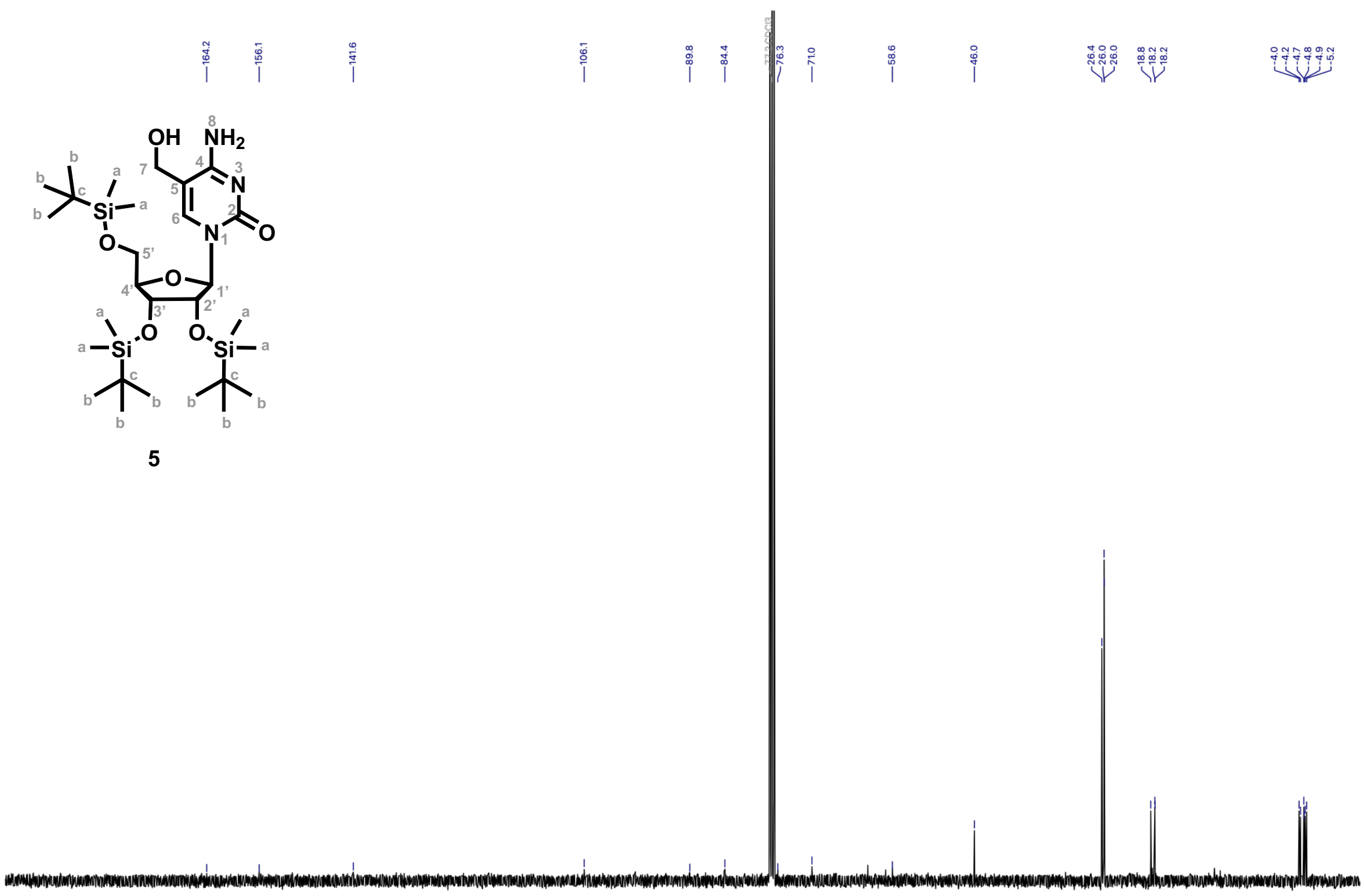

*O*2',3',5'-Tris(*tert*-butyldimethylsilyl)-5-methyl-cytidine (7)

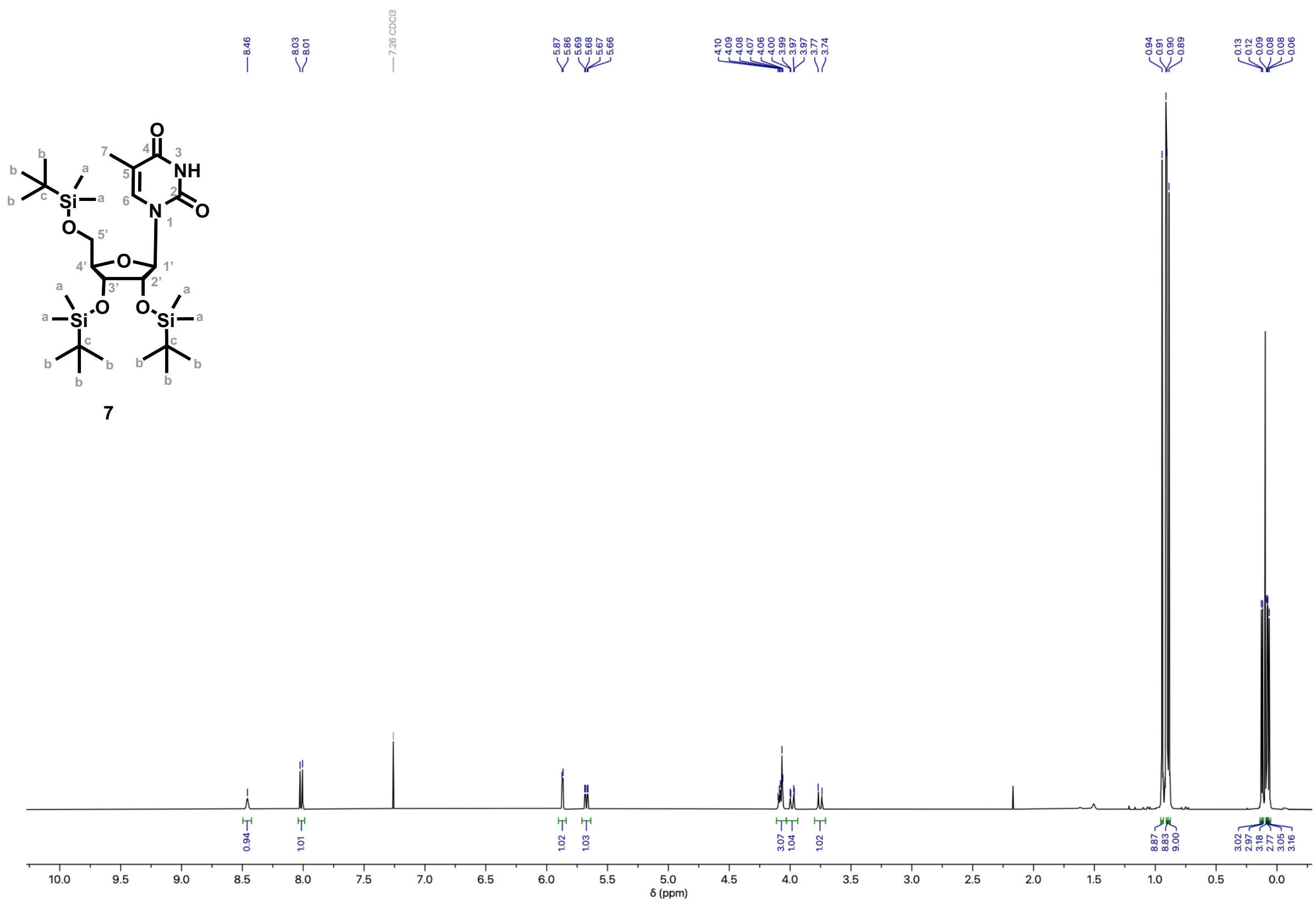

7

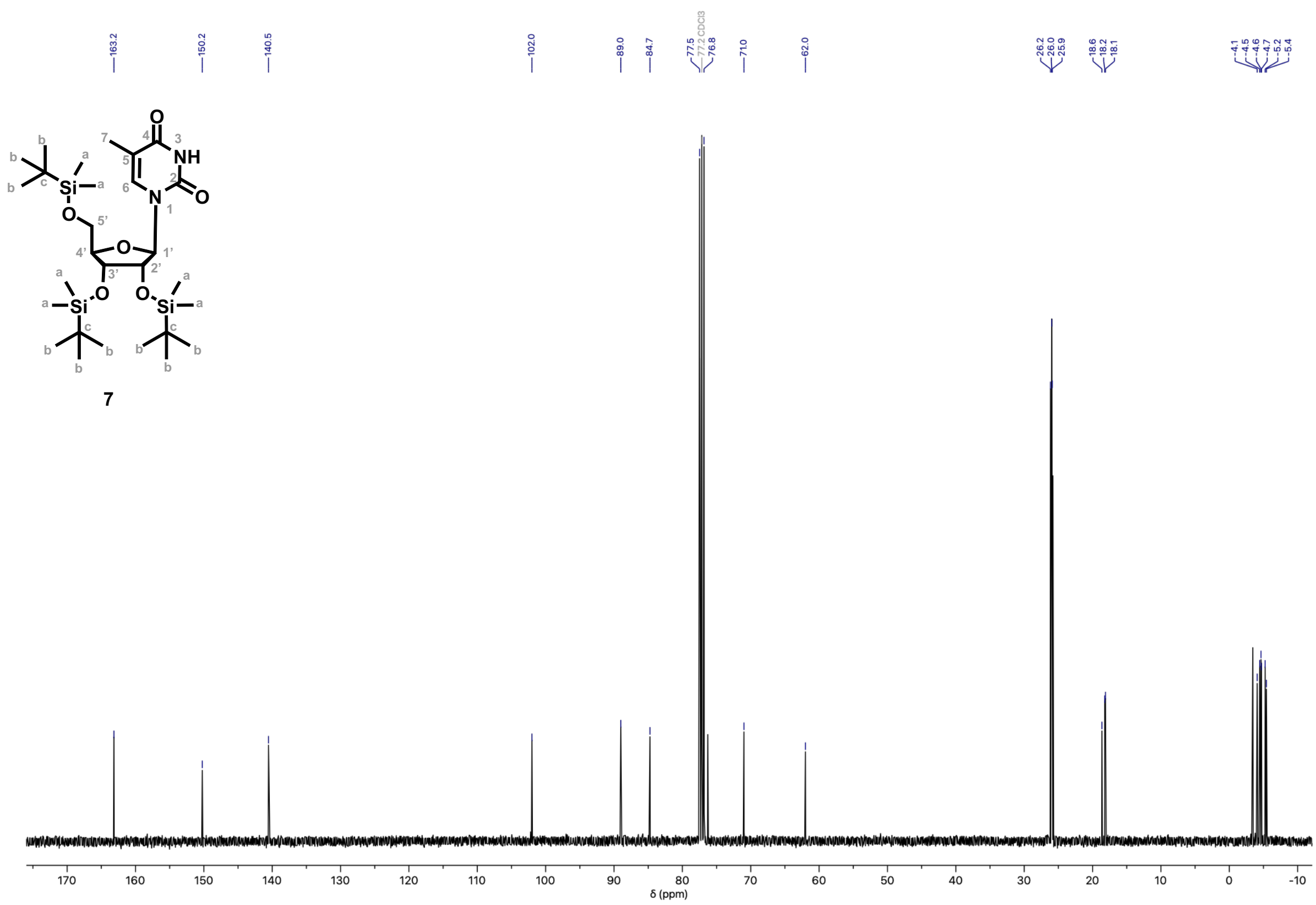

7

_O2_',3',5'-Tris(_tert_-butyldimethylsilyl)-_O4_-(2,4,6-Tri(iso-propyl)phenylsulfonyl)-5-methyl-cytidine (8)

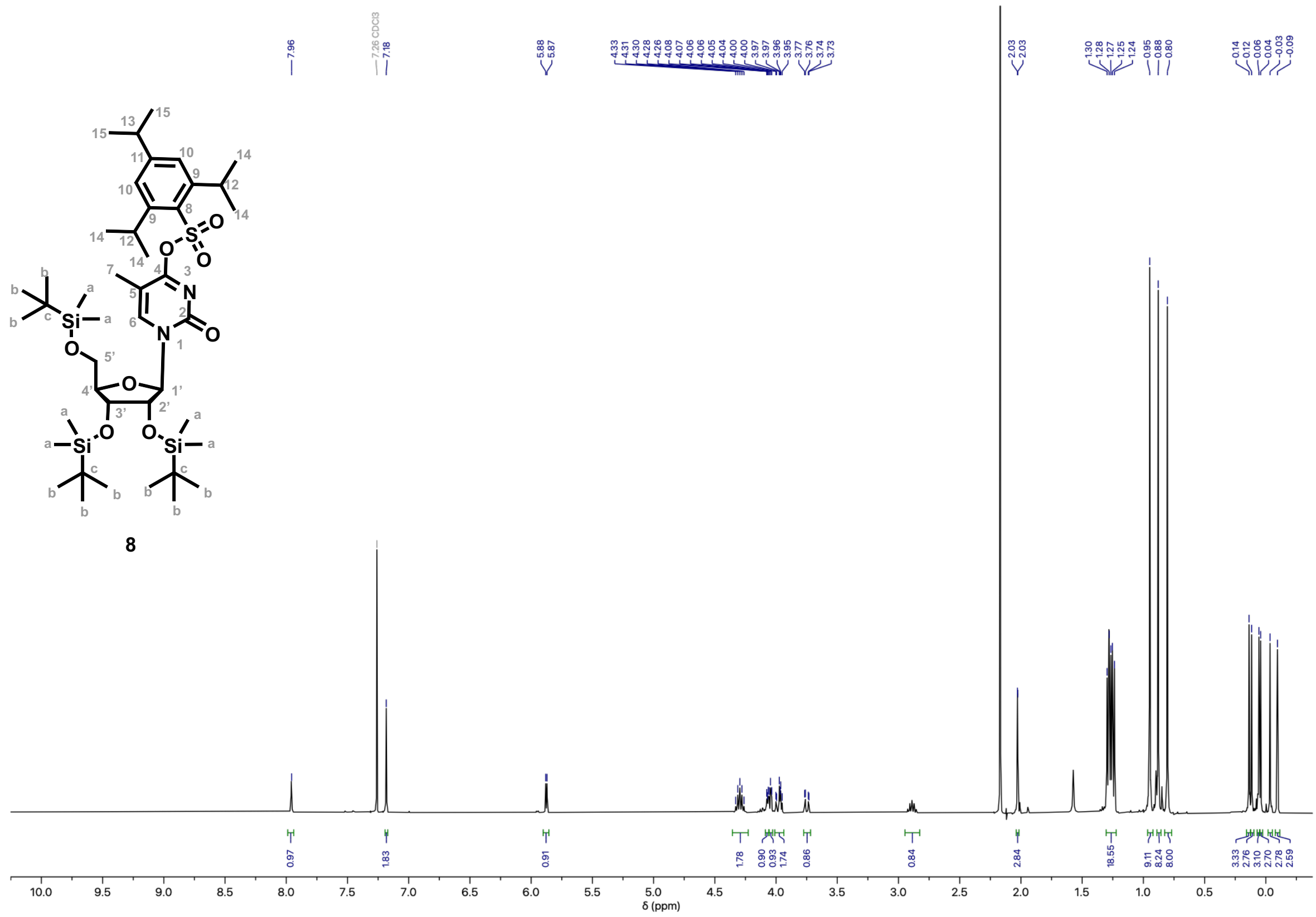

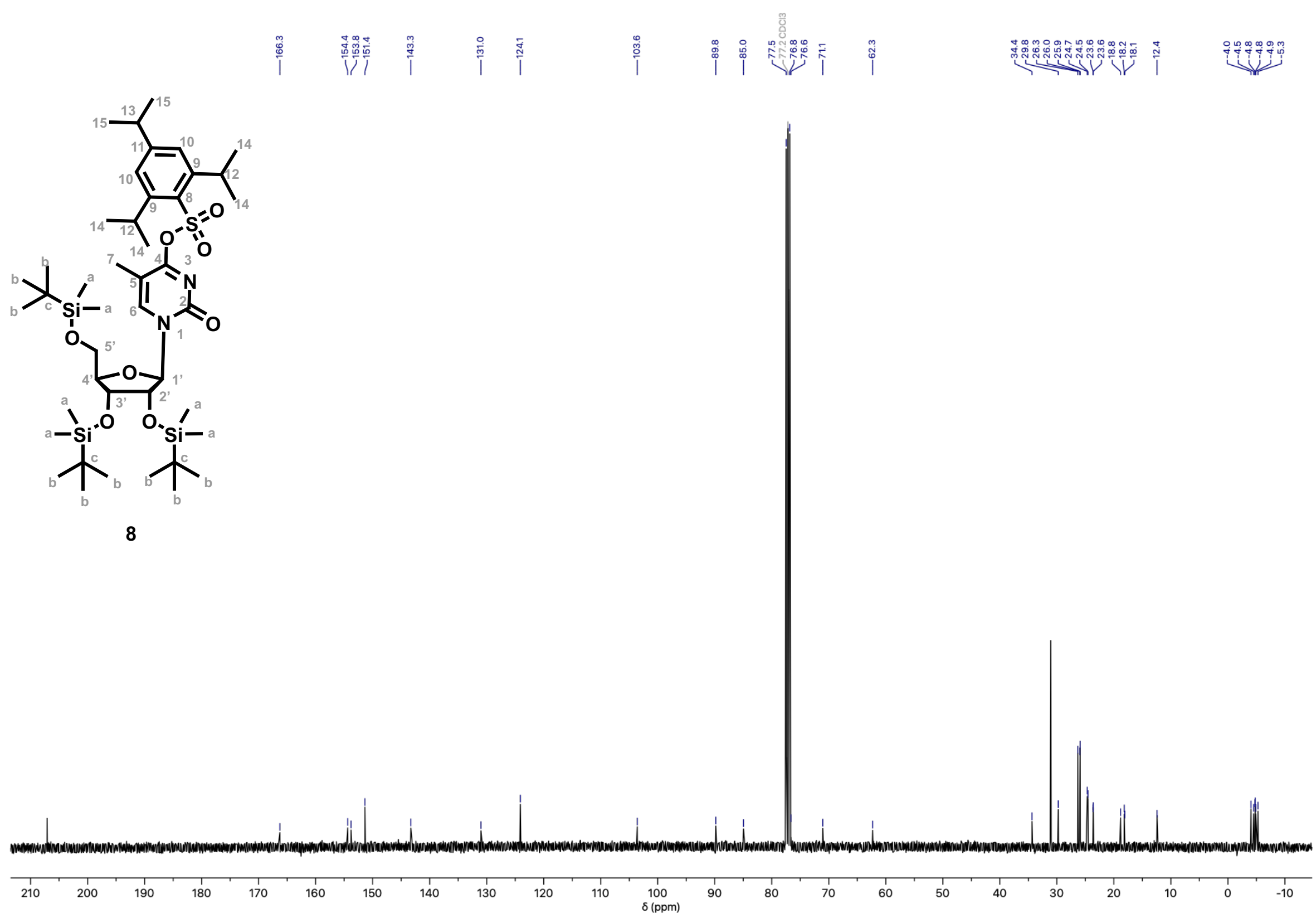

5-methyl-cytidine (m5C)

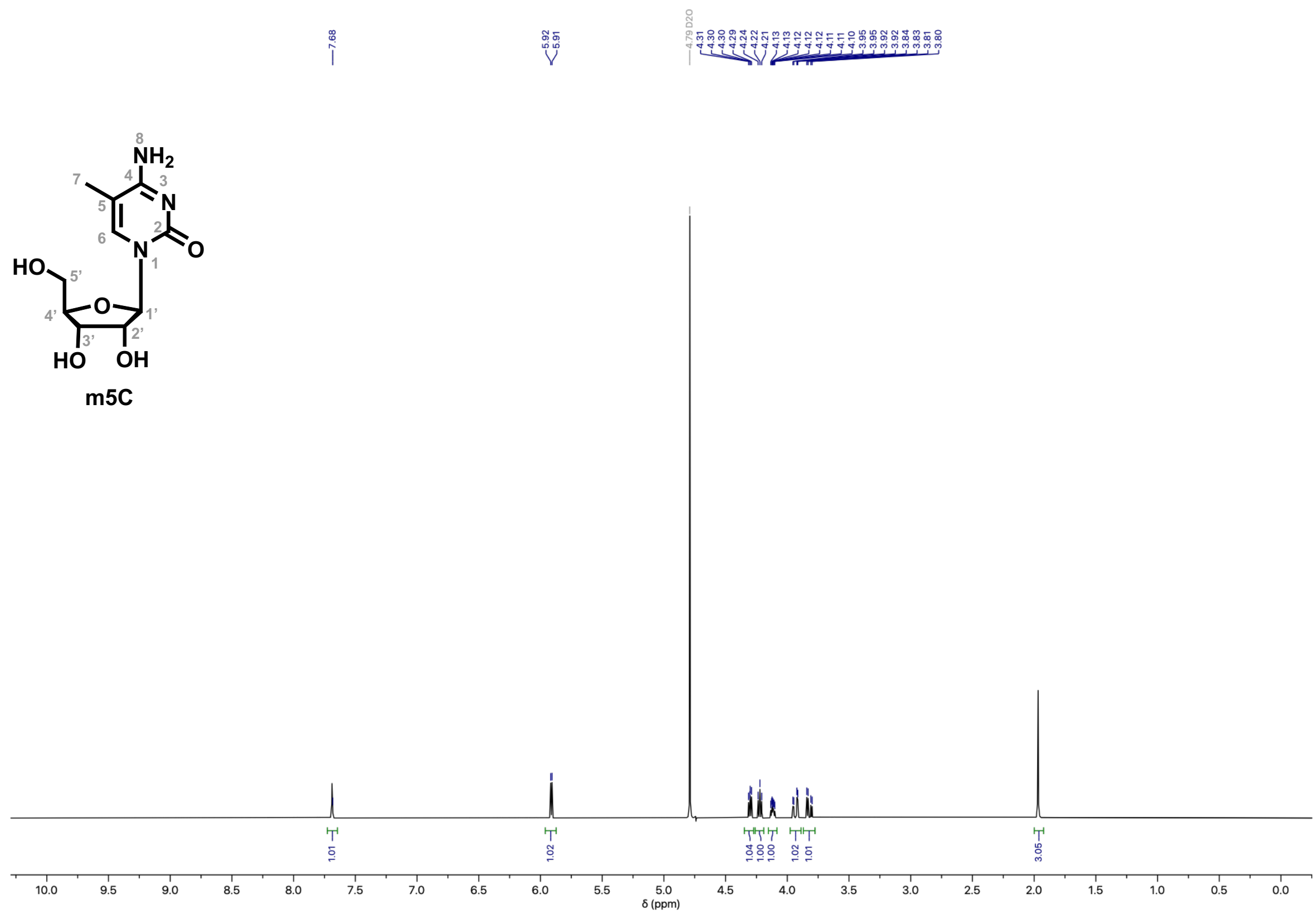

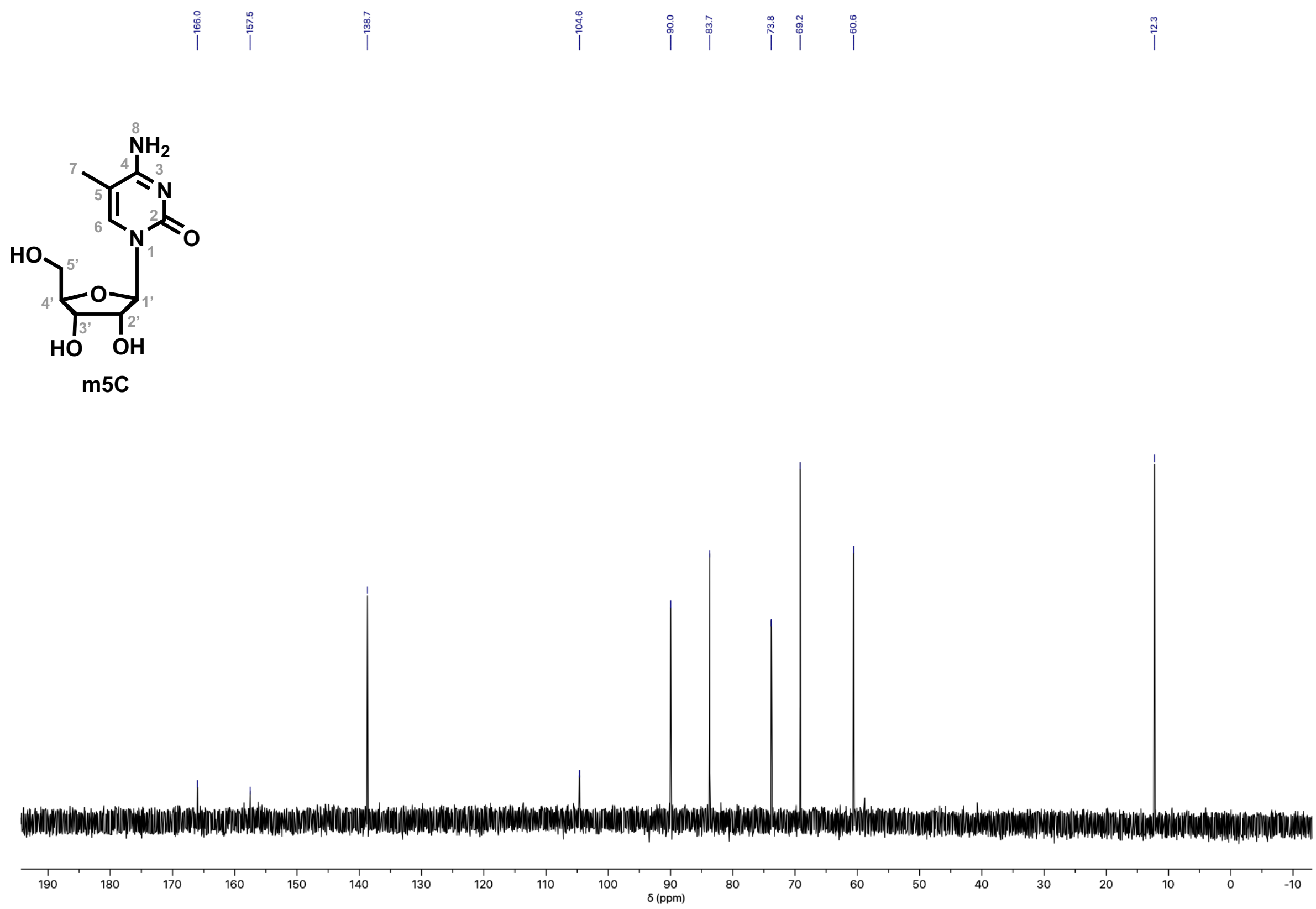

5-Hydroxymethyl-cytidine (hm5C)

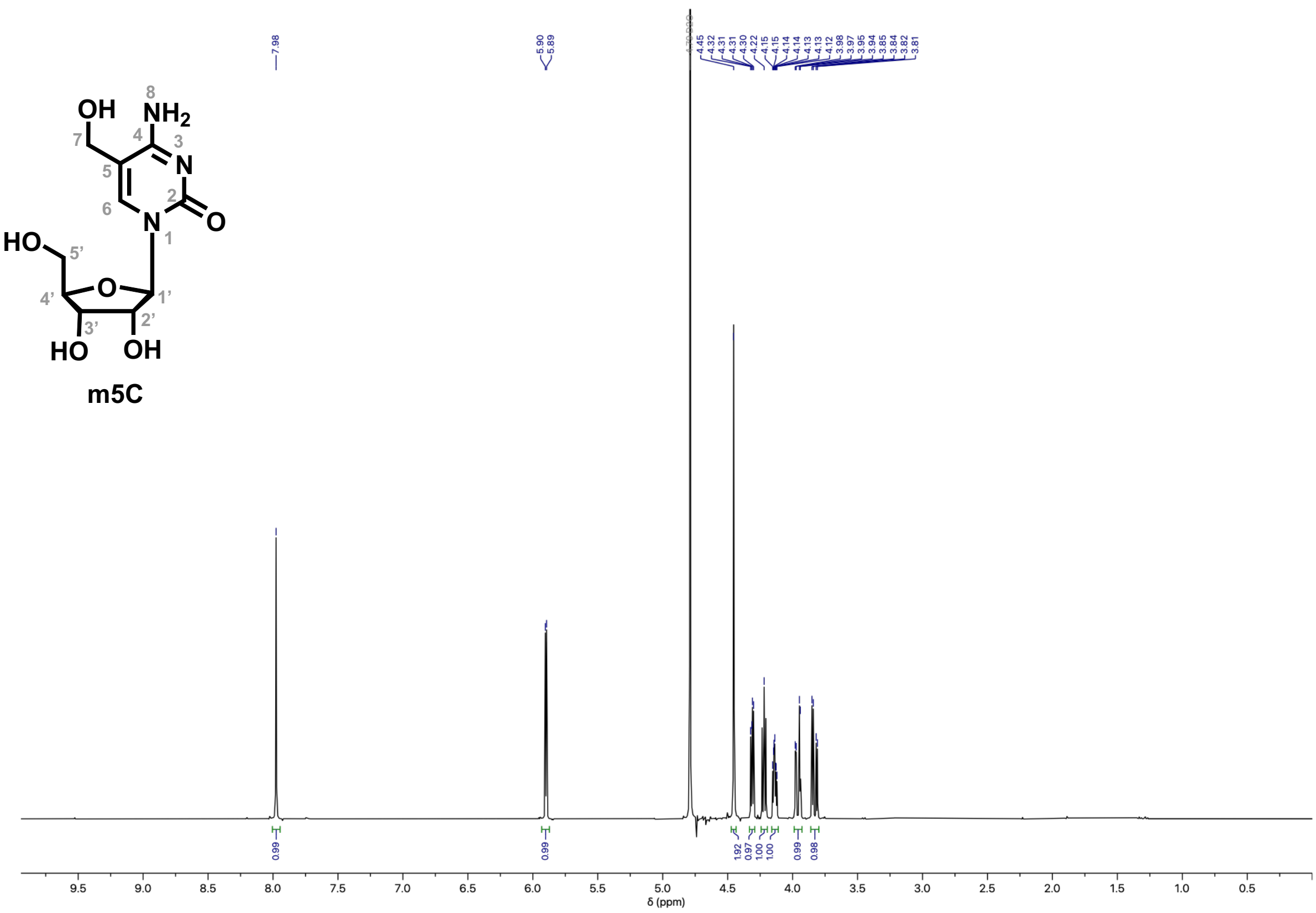

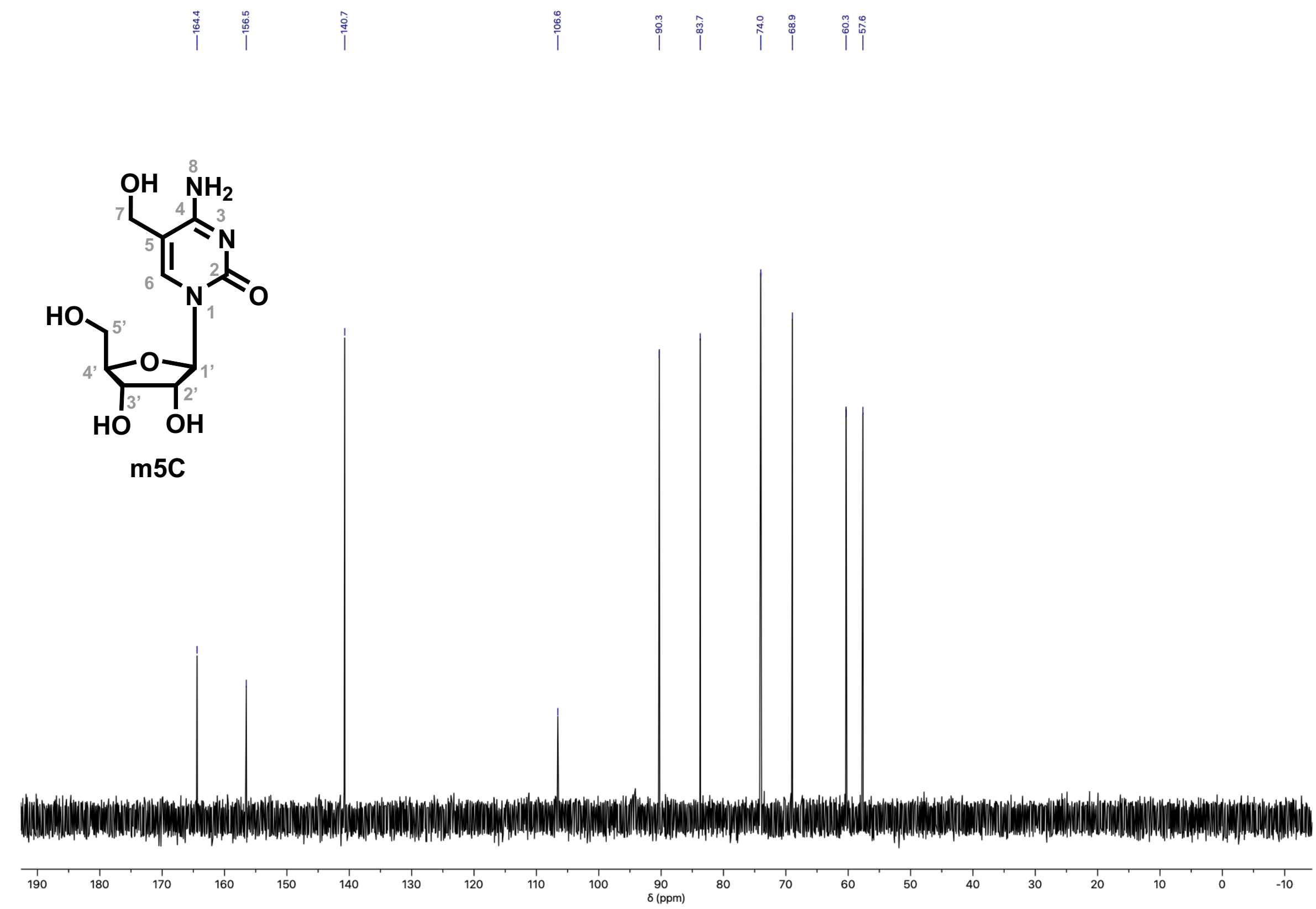

5-Formyl-cytidine (f5C)

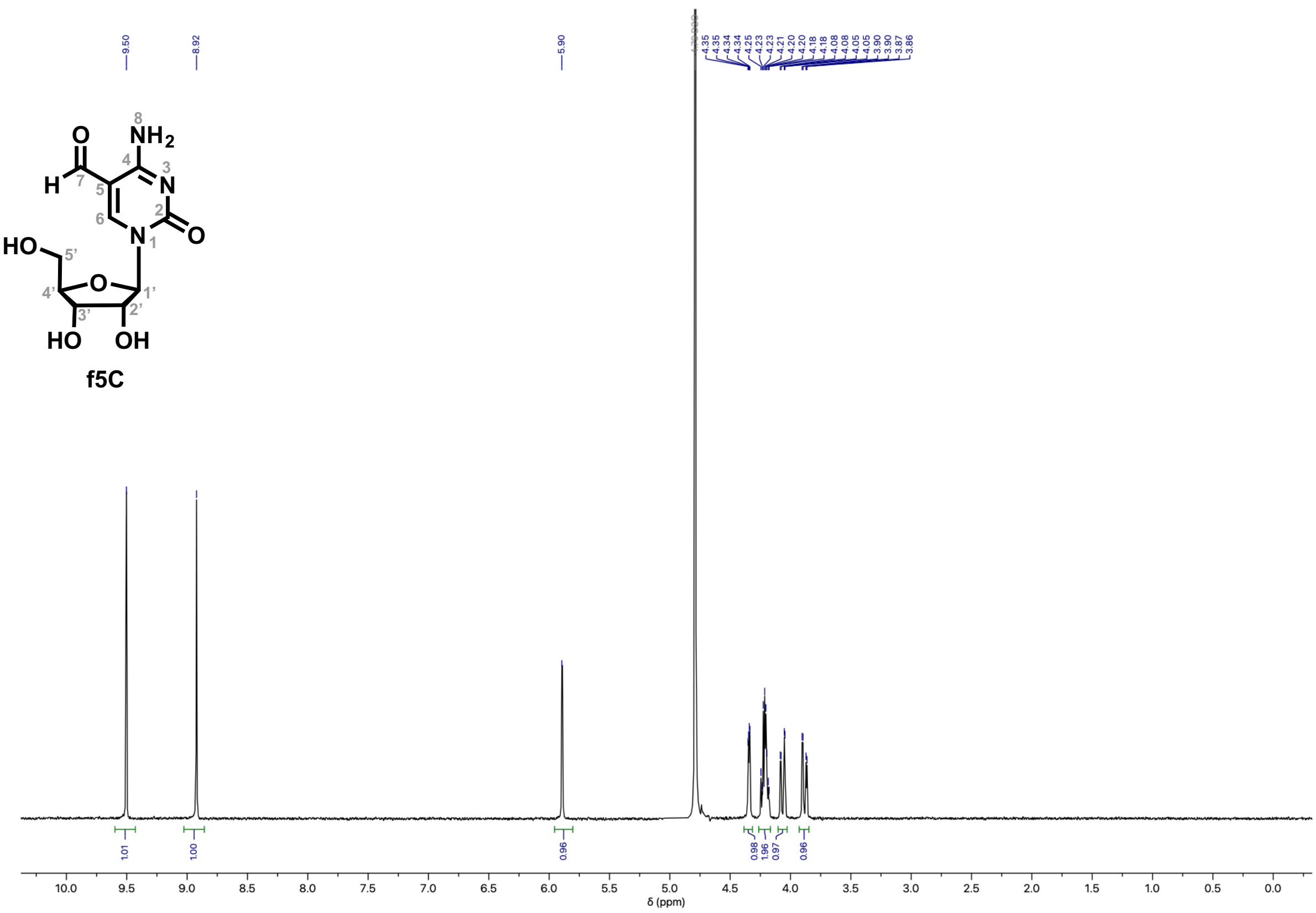

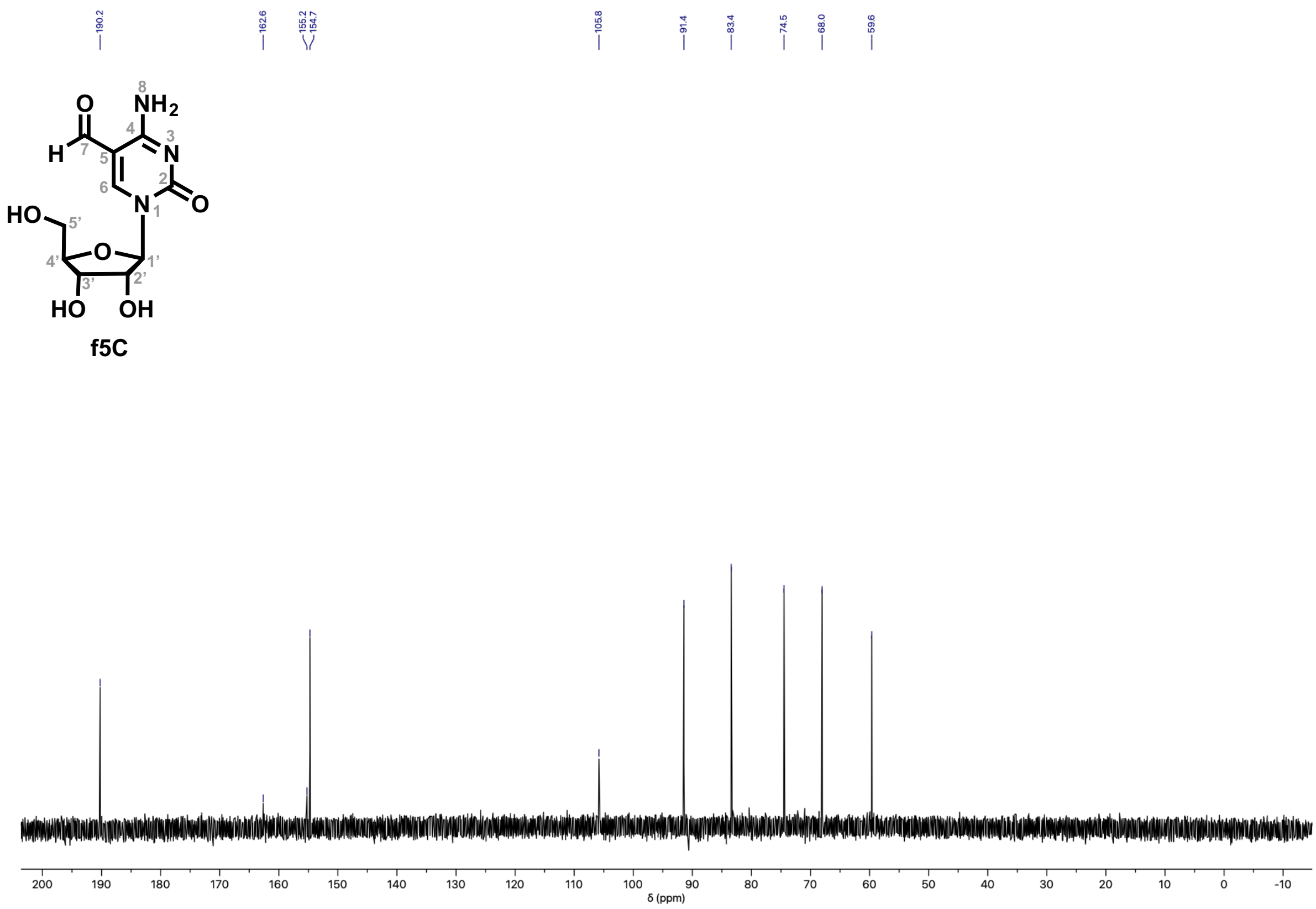

5-Carboxyl-cytidine (ca5C)

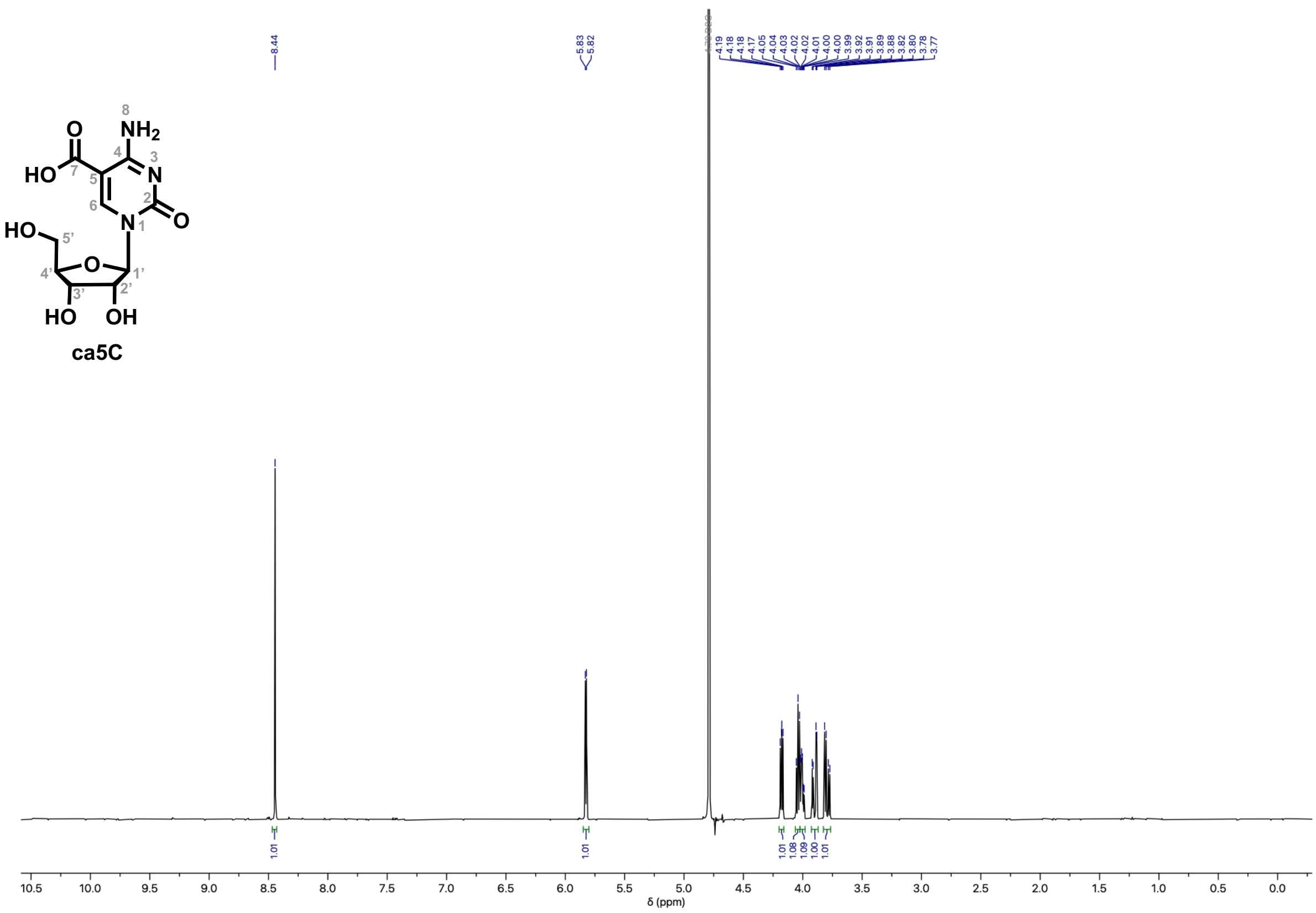

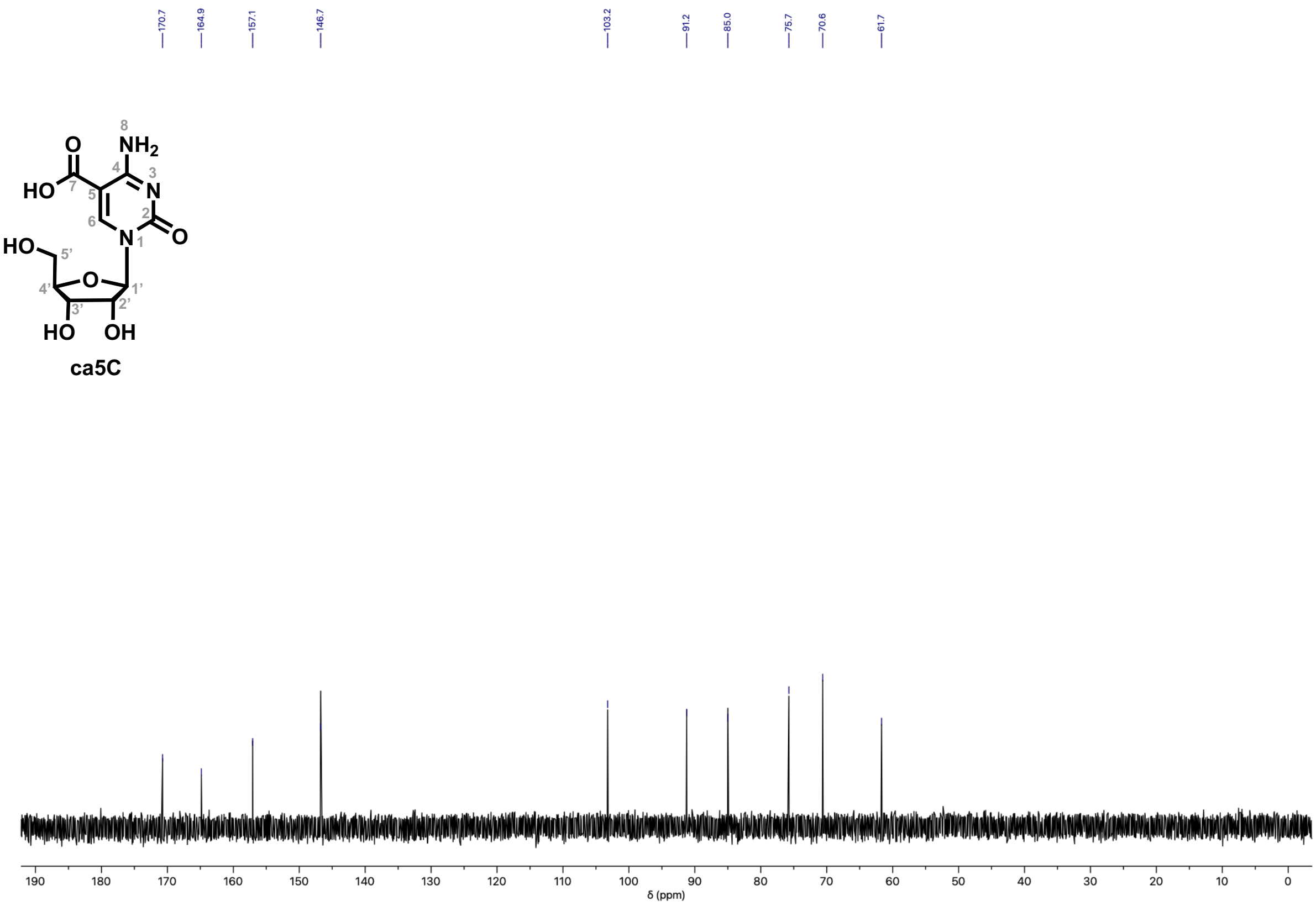

Uracil-[$^{15}N_2$] (10)

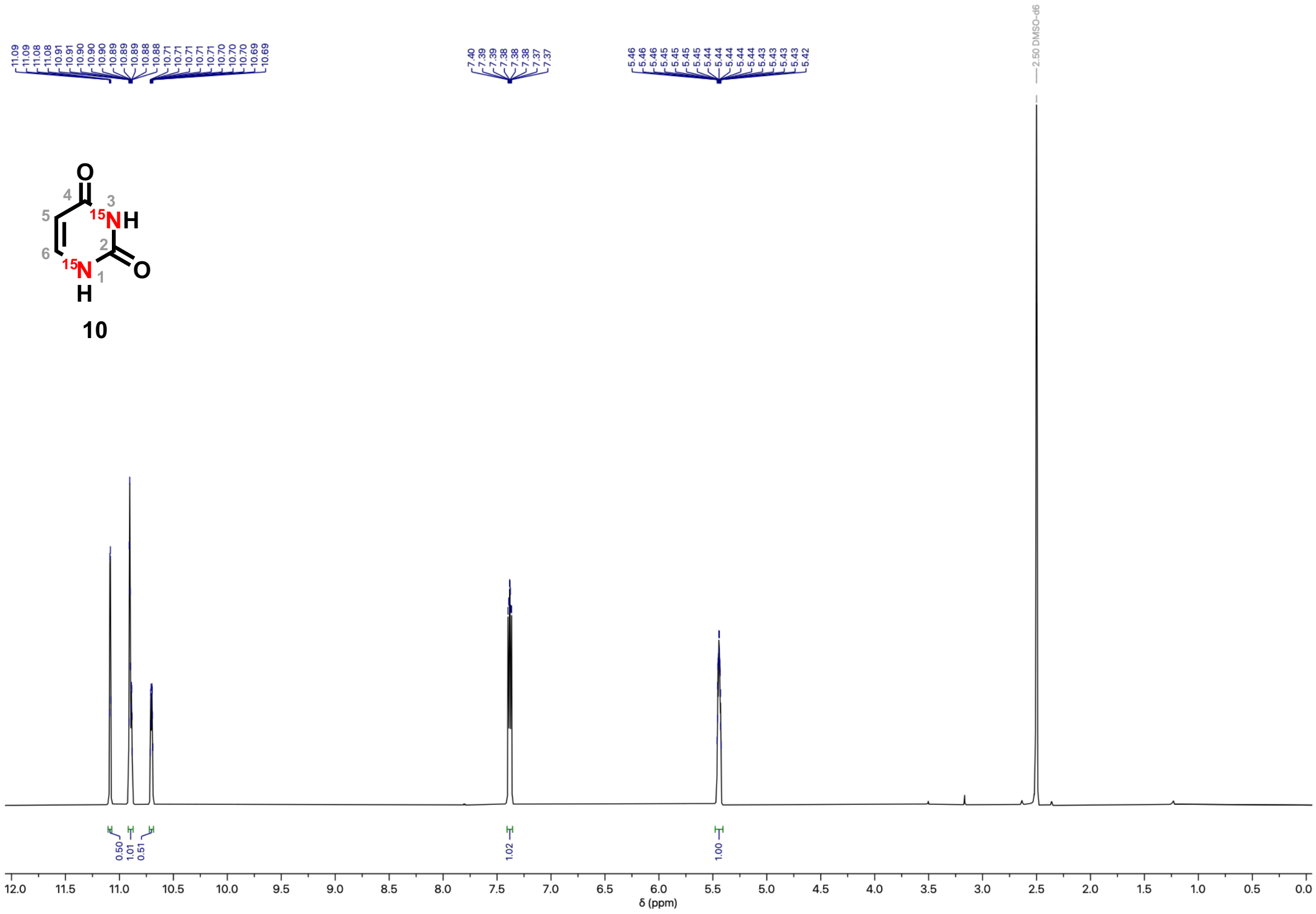

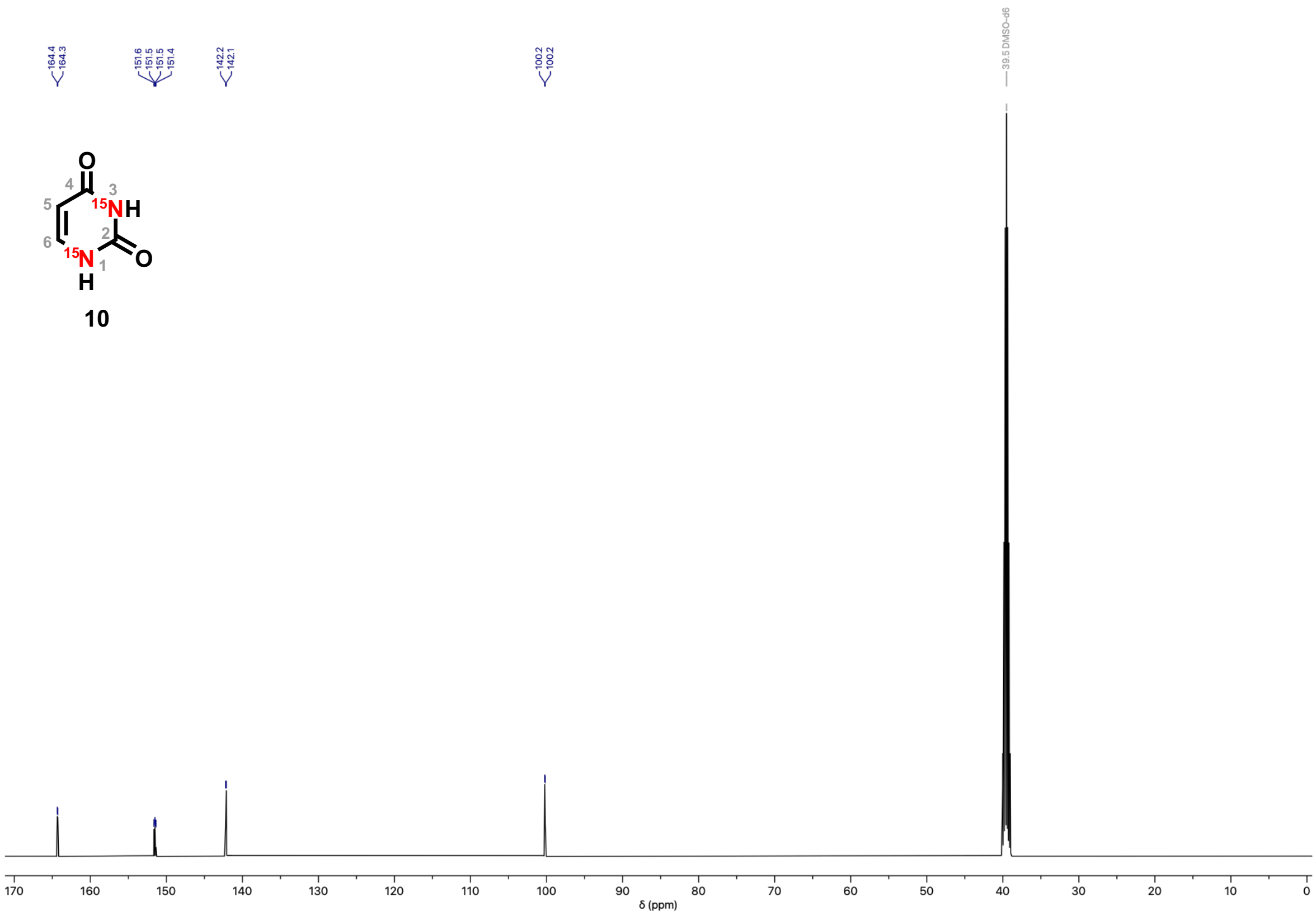

*O*2',3',5'-Tris(benzoyl)-(N^1,N^3-^{15}N)-uridine (11)

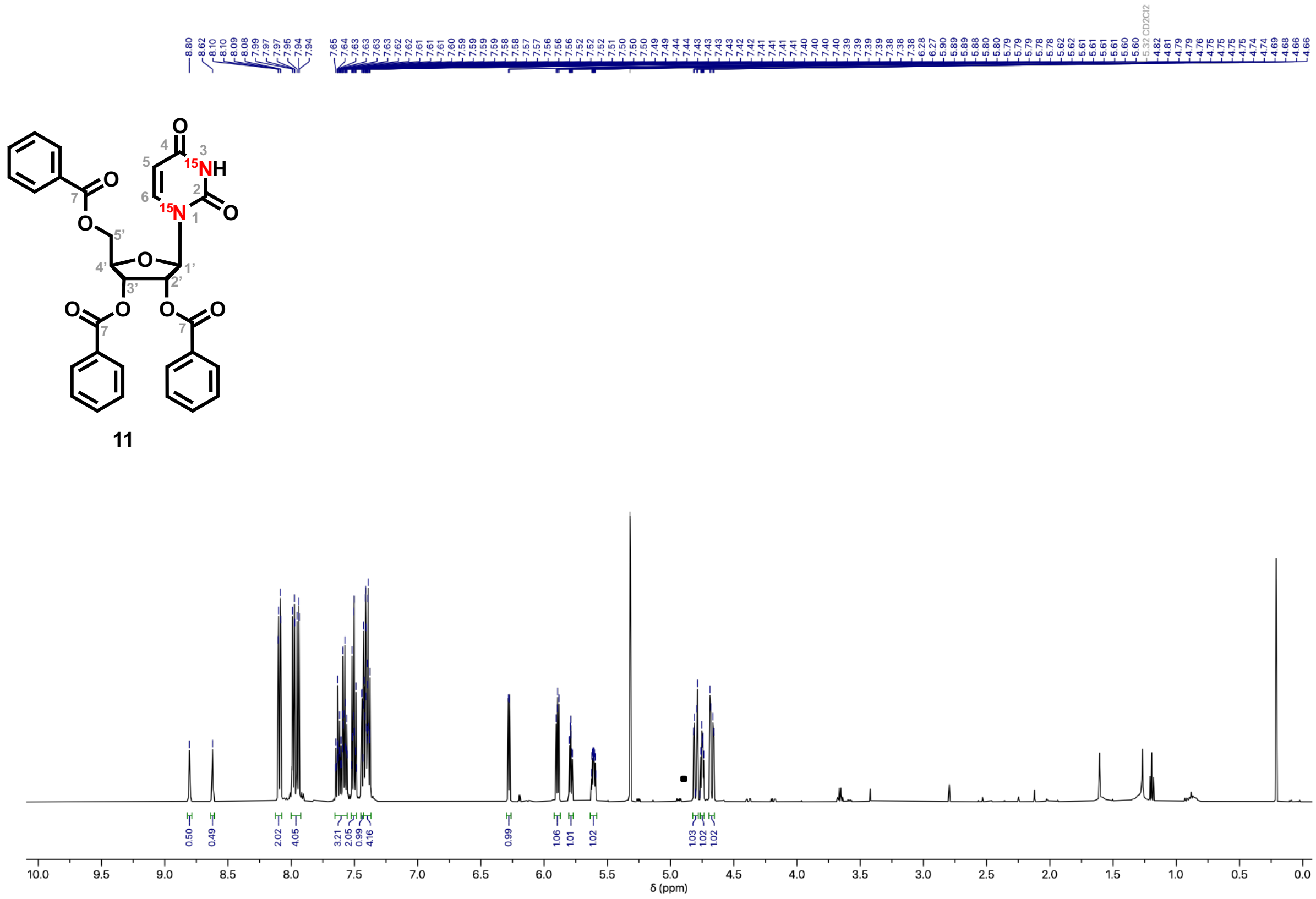

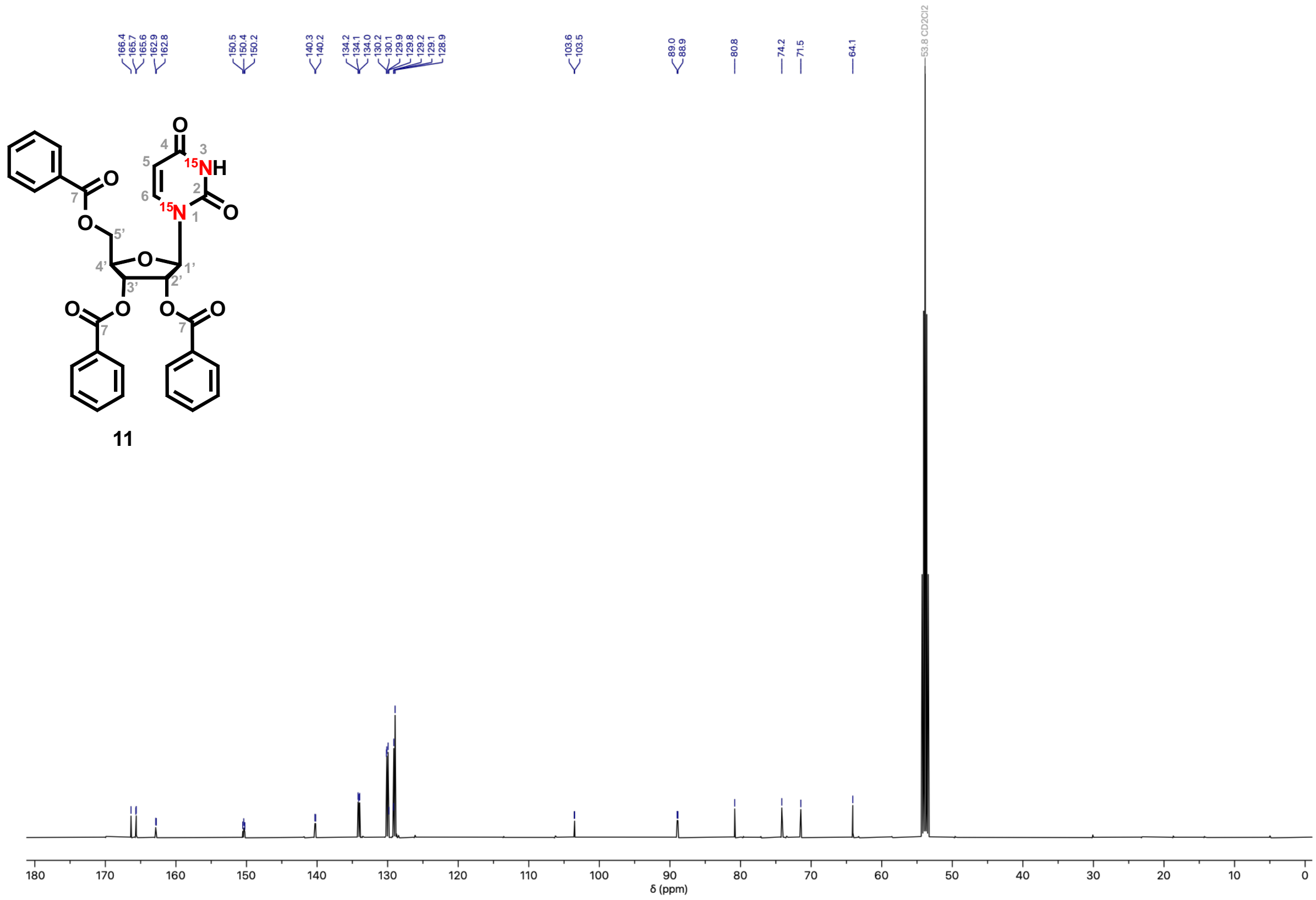

(N^1,N^3-^{15}N)-uridine (12)

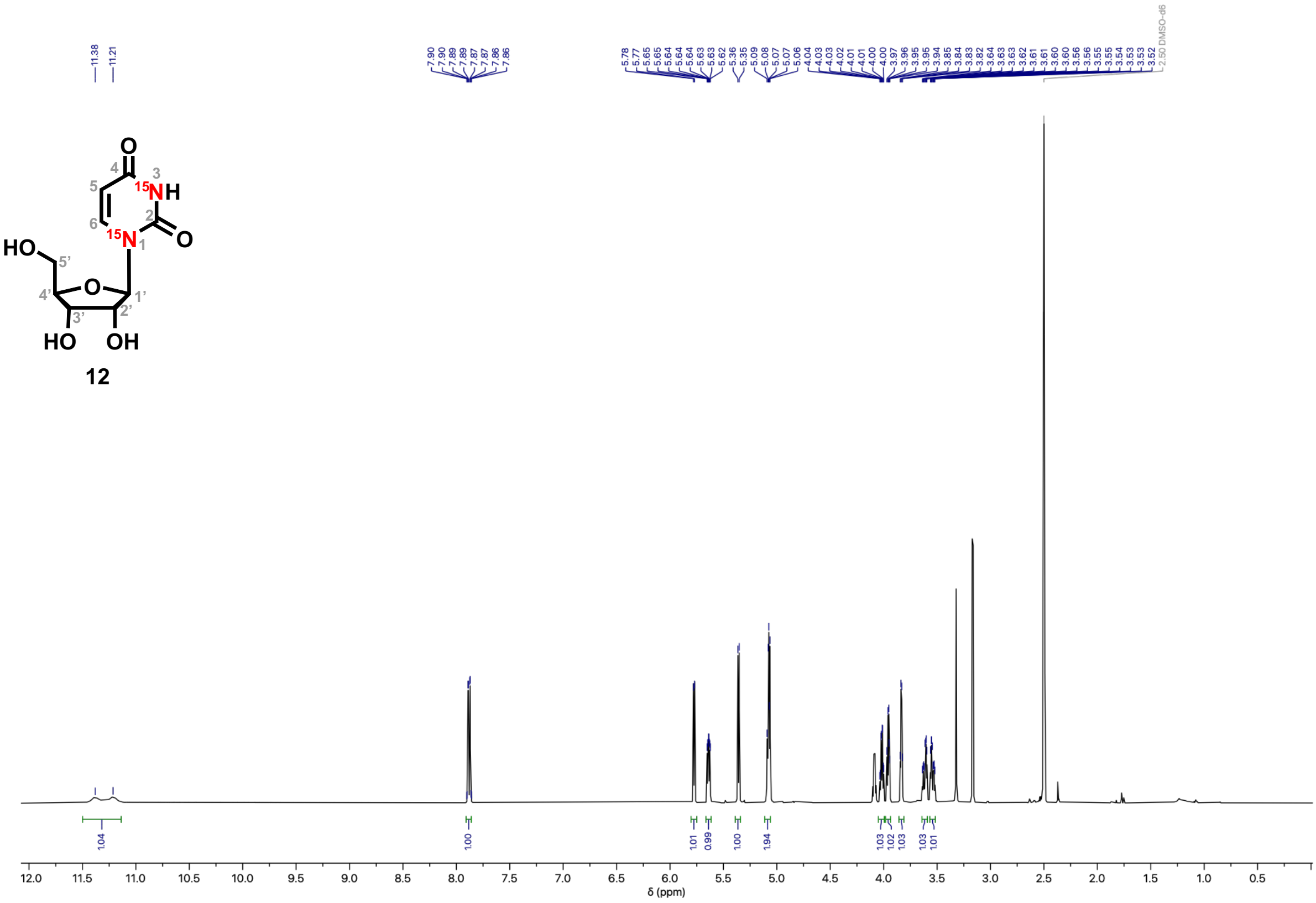

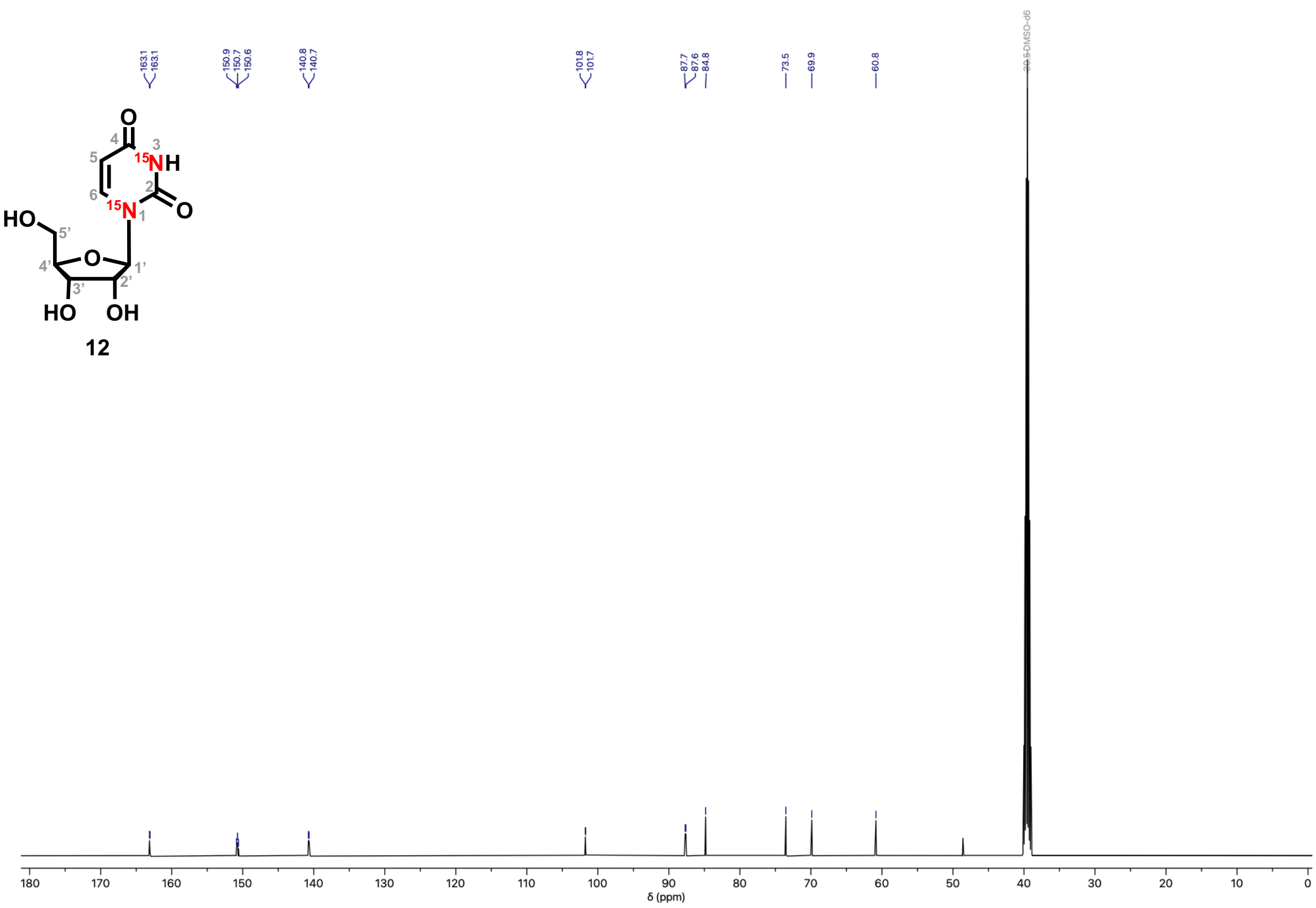

*O*2',3',5'- Tris(*tert*-butyldimethylsilyl)-(*N*[1],*N*[3]-^{15}N)-uridine (13)

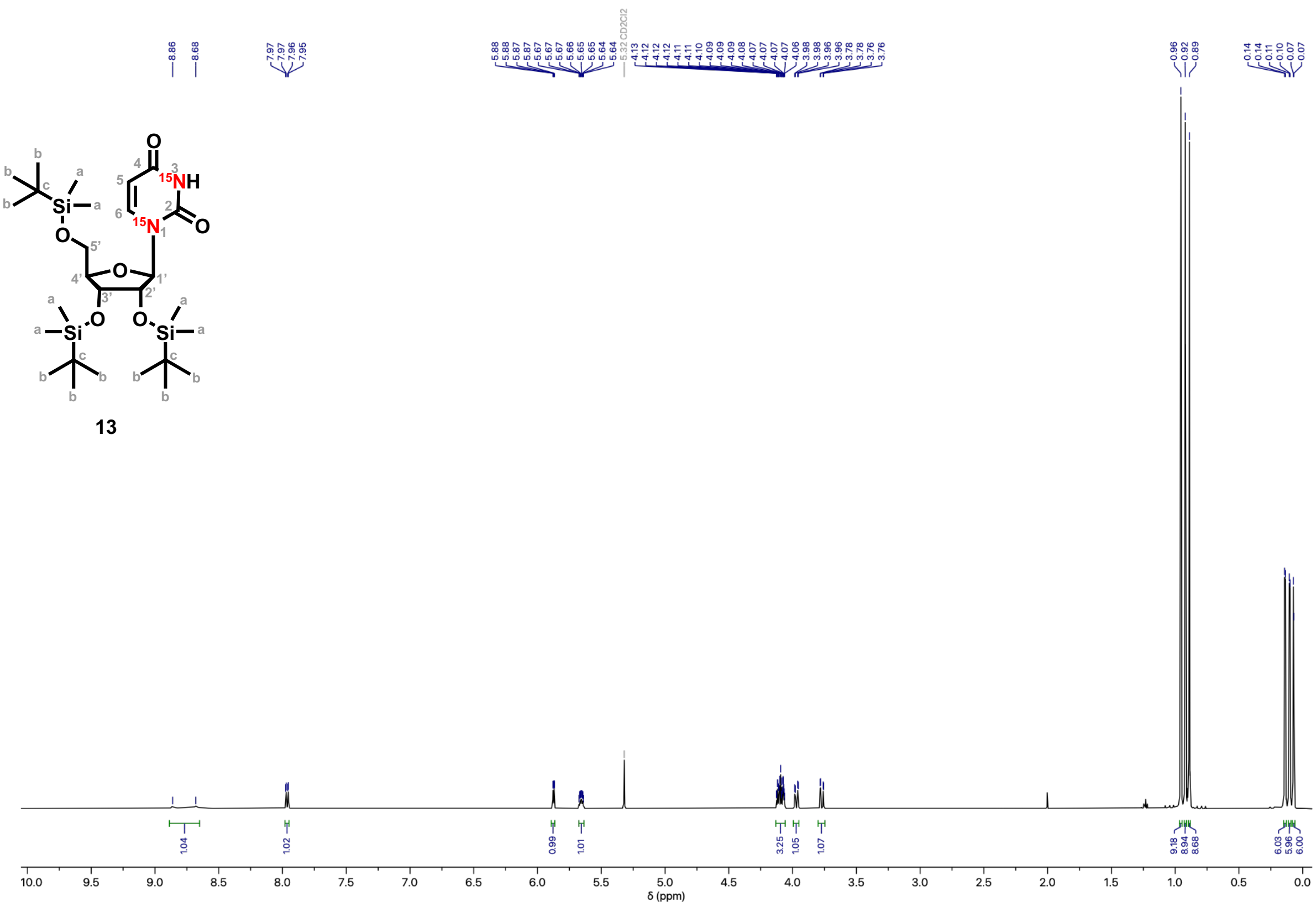

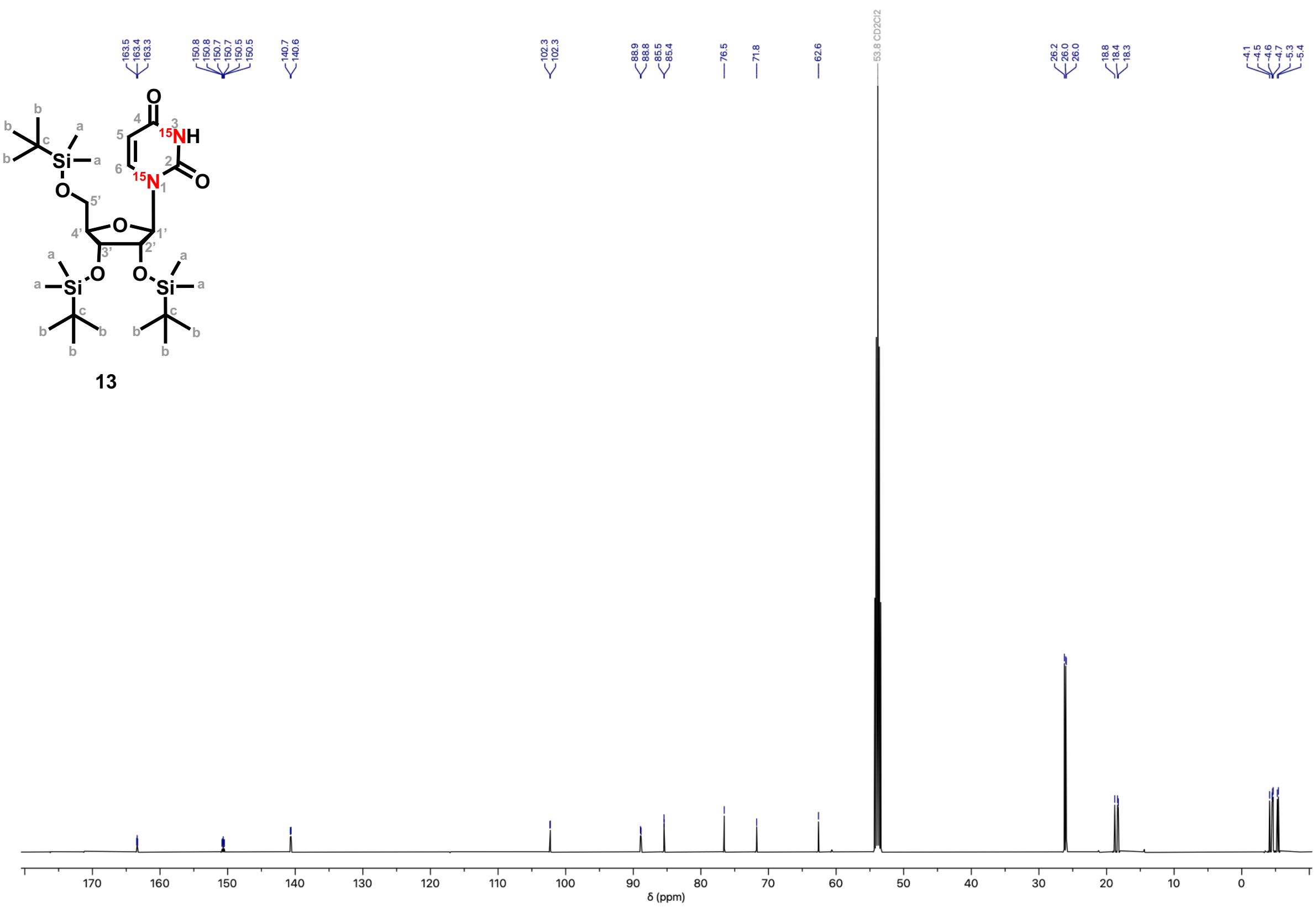

*O*2',3',5'- Tris(*tert*-butyldimethylsilyl)-(N^1,N^3,N^4-^{15}N)-cytidine (14)

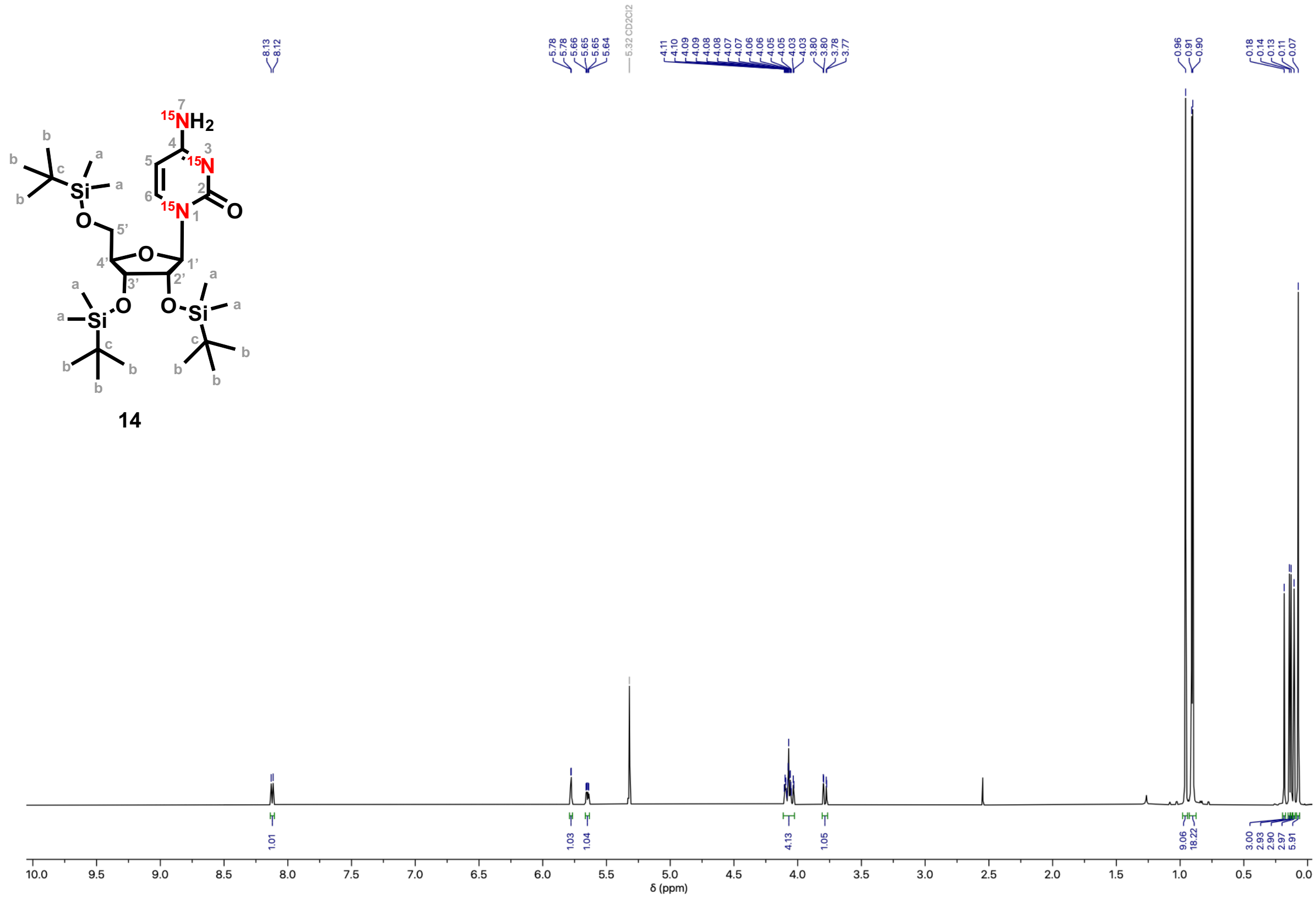

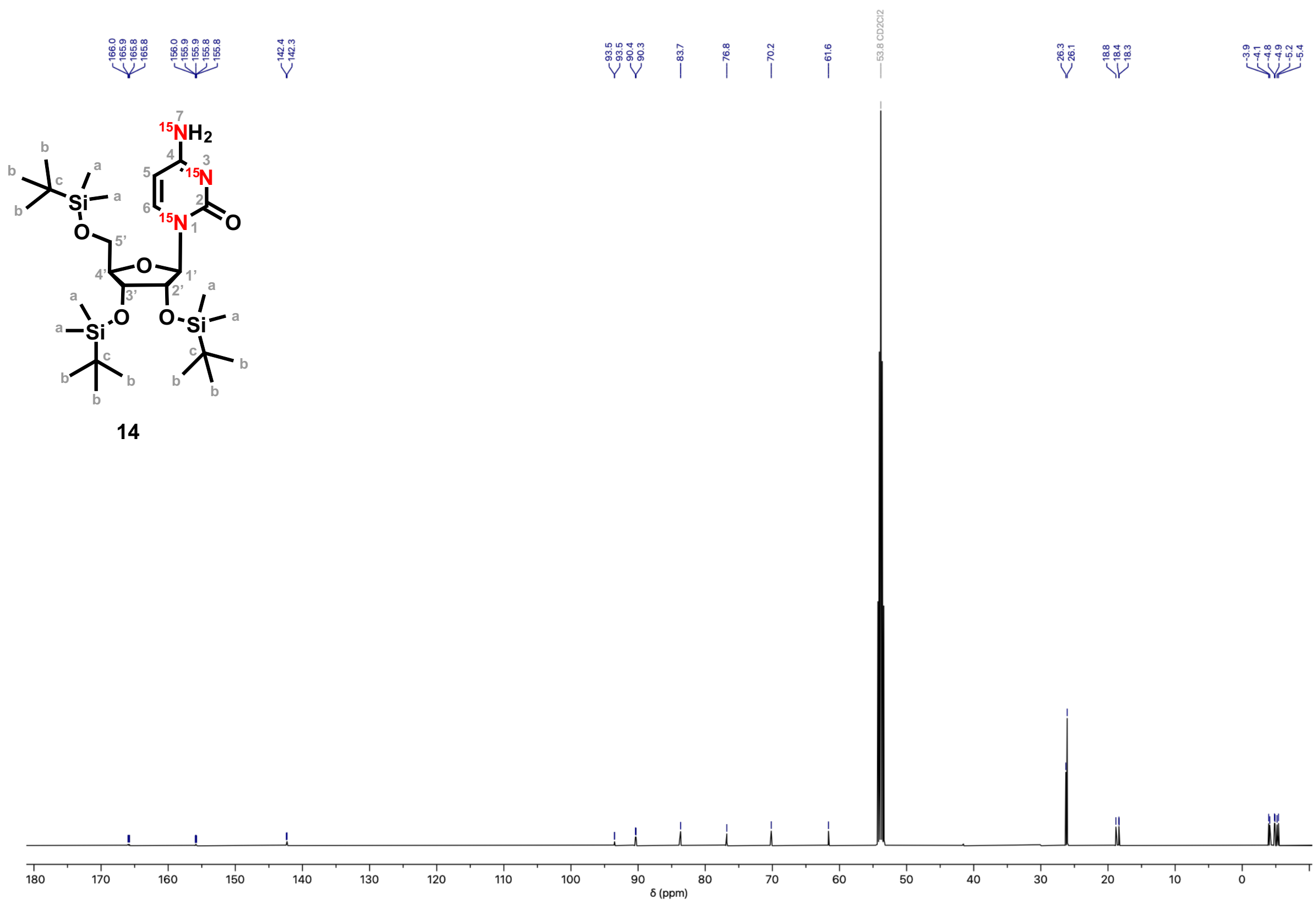

*O*2',3',5'-Tris(*tert*-butyldimethylsilyl)-5-iodo-(N^1,N^3,N^4-^{15}N)-cytidine (15)

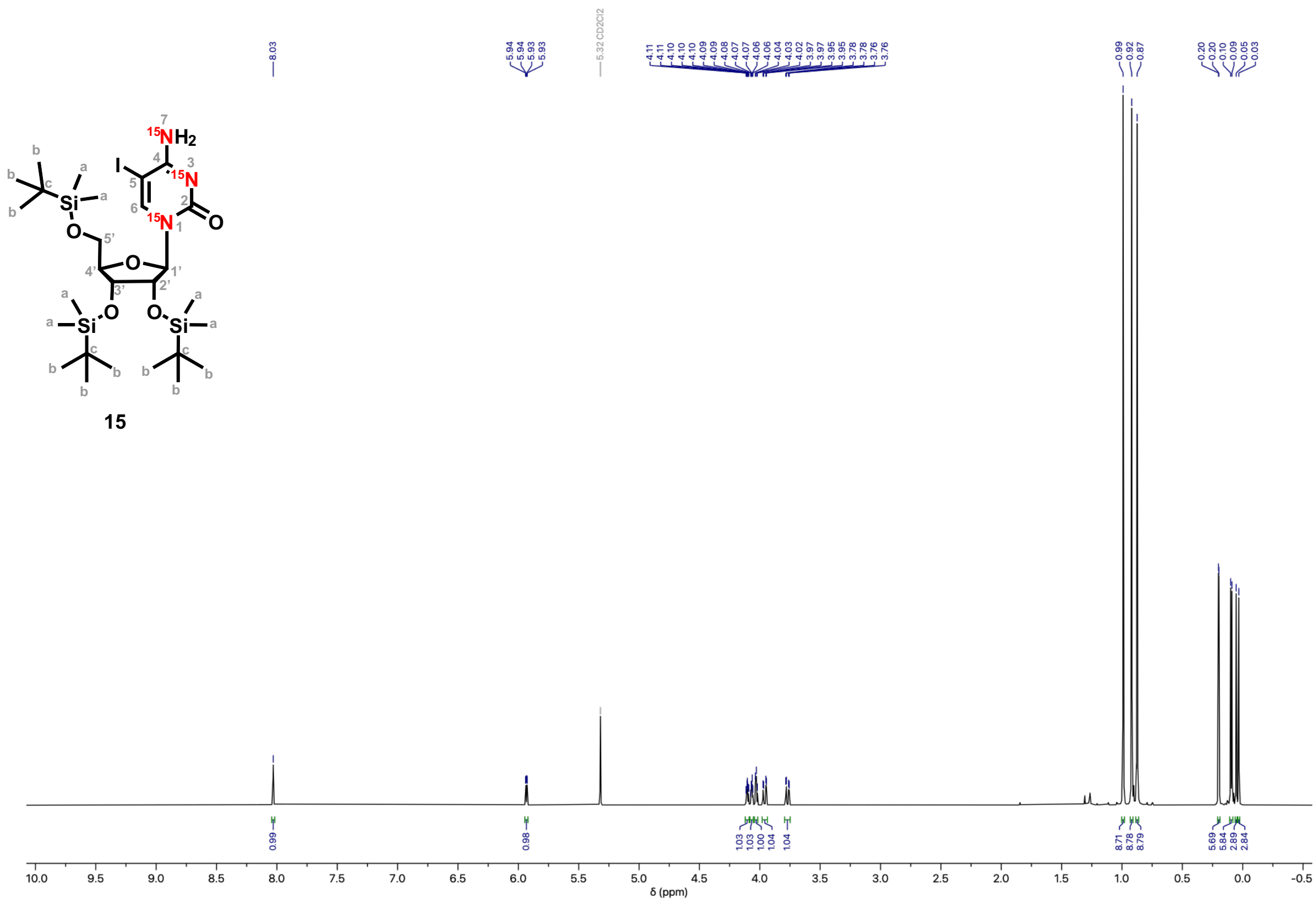

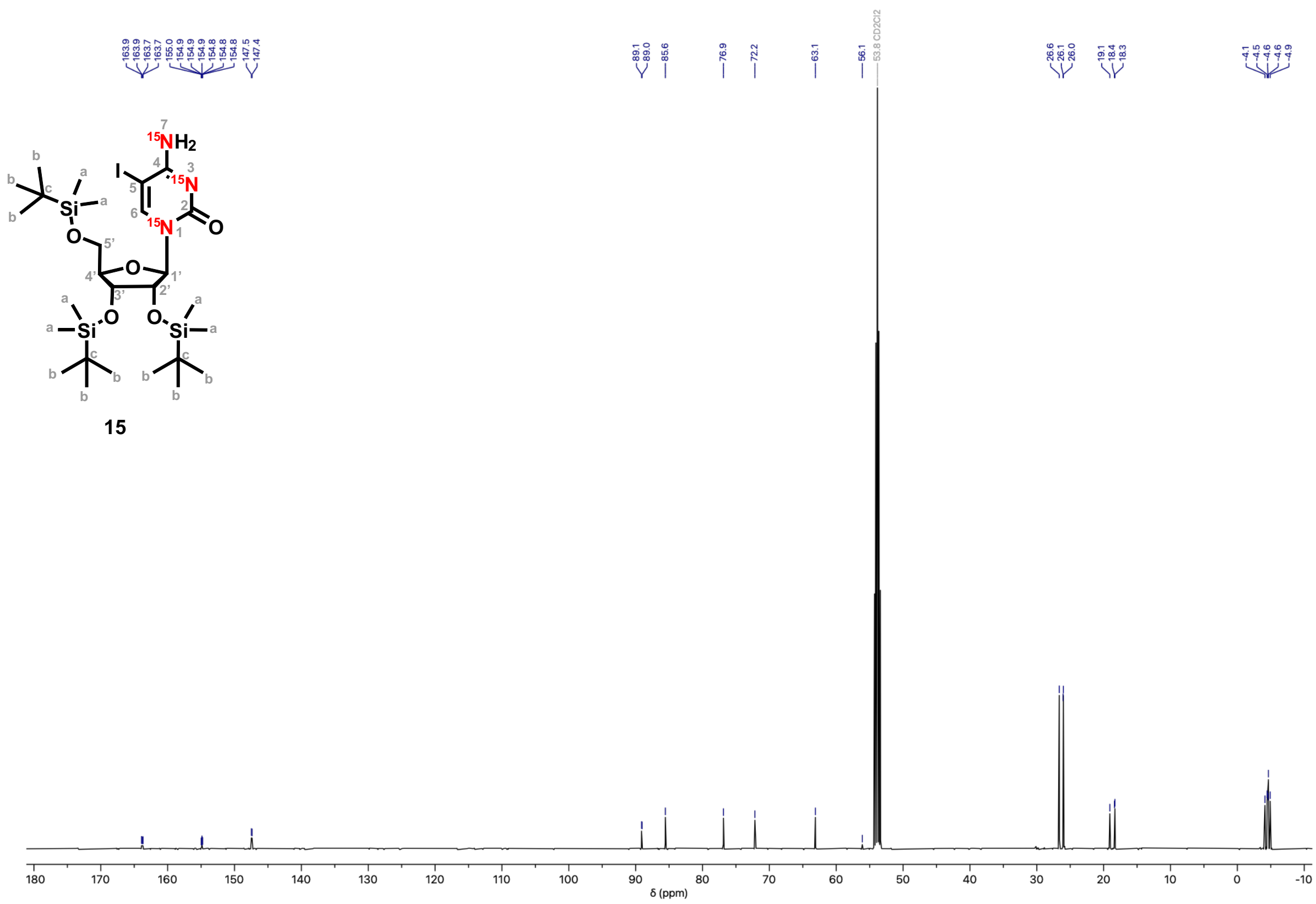

*O*2',3',5'-Tris(*tert*-butyldimethylsilyl)-5-(methoxycarbonyl)-(*N*1,*N*3,*N*4-^{15}N)-cytidine (16)

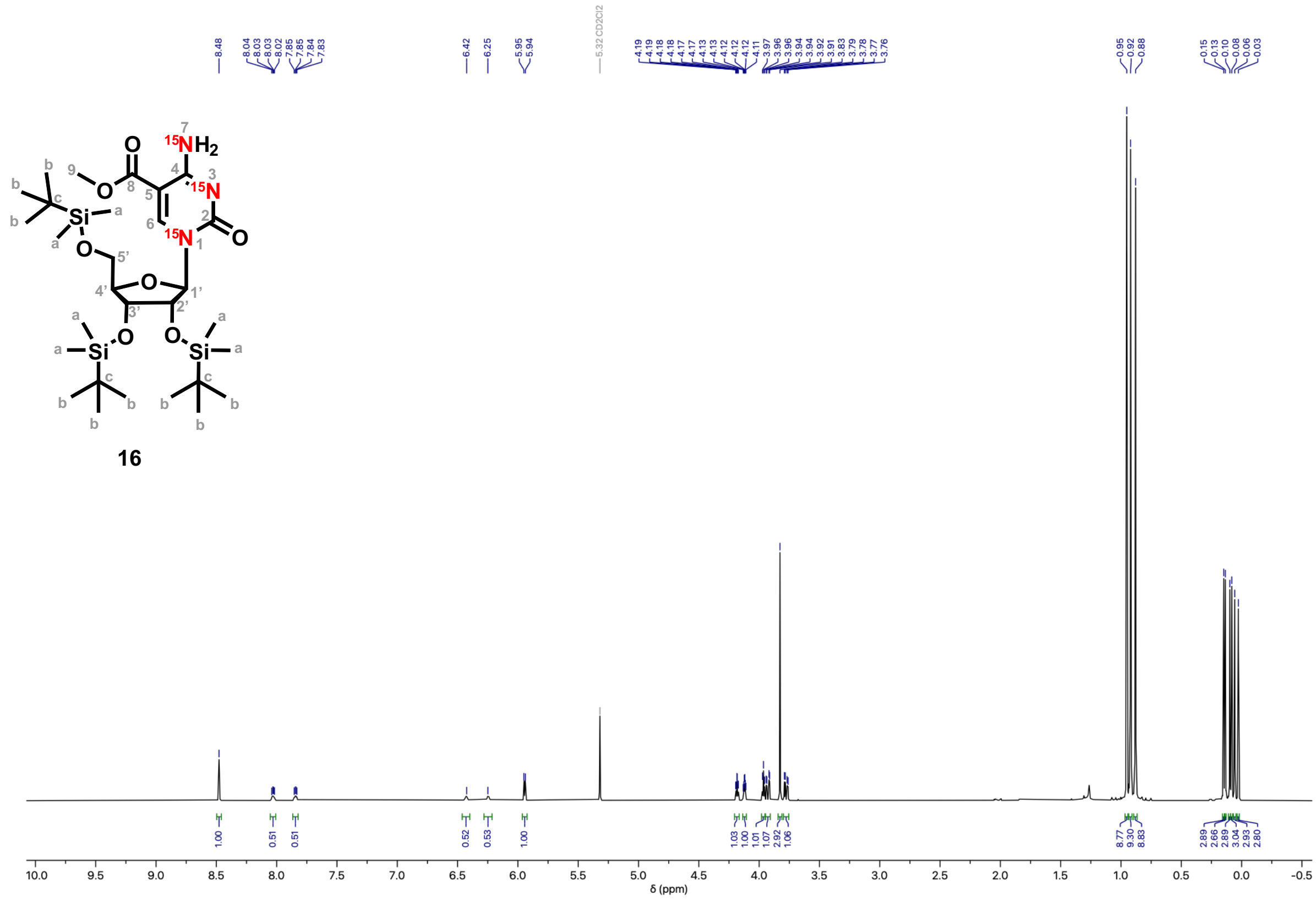

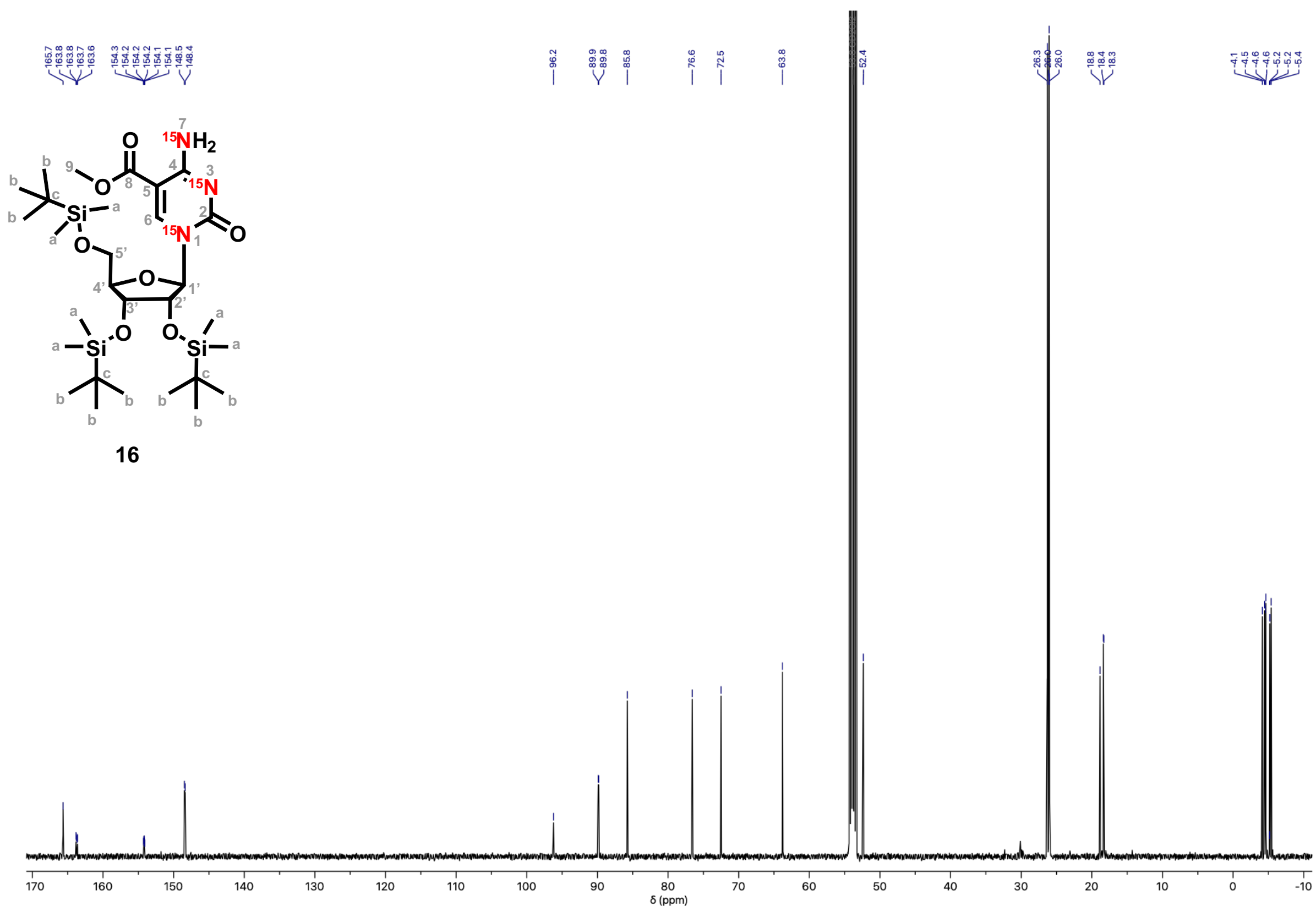

O2',3',5'-Tris(*tert*-butyldimethylsilyl)-5-formyl-(N^1,N^3,N^4-^{15}N)-cytidine (17)

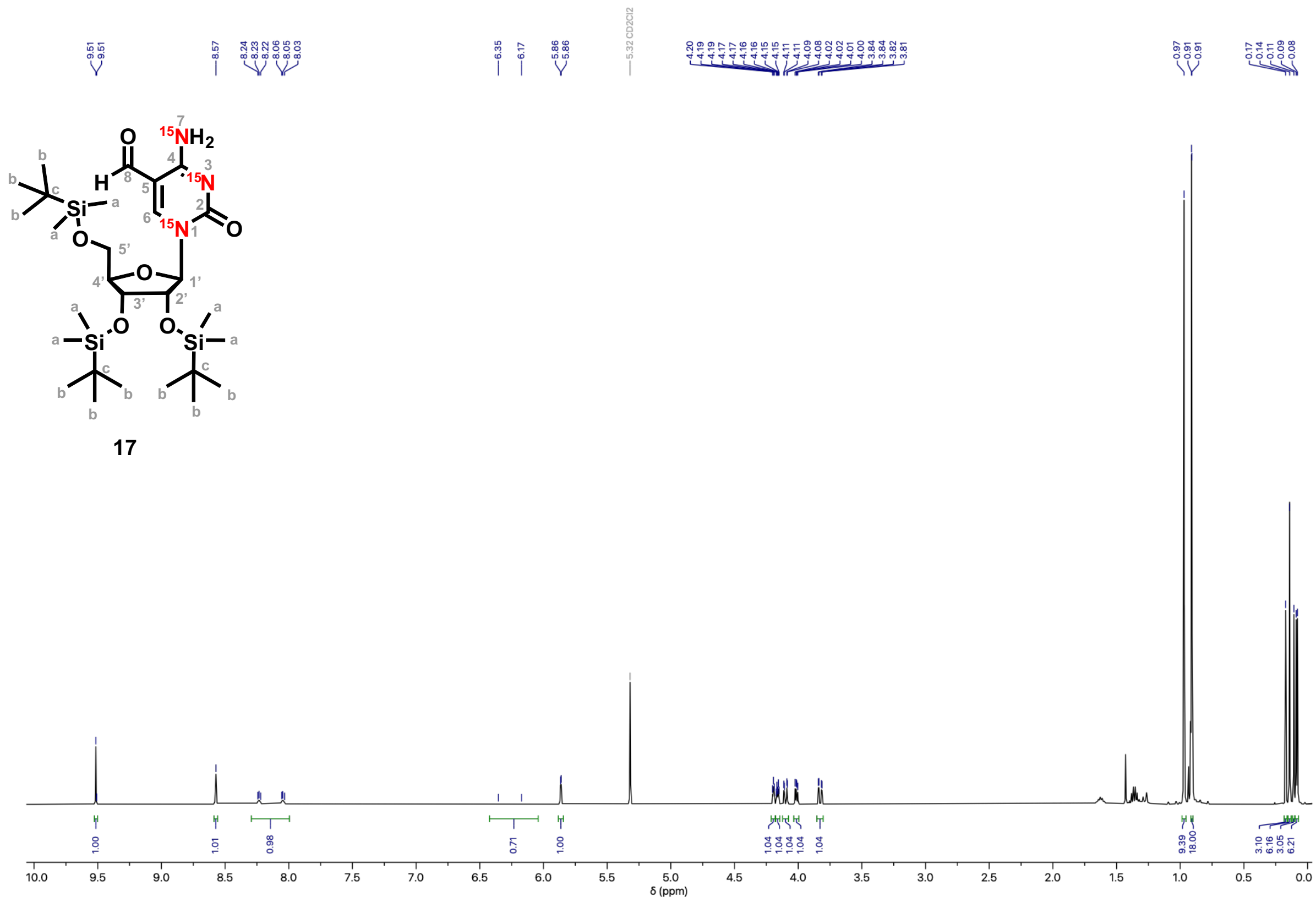

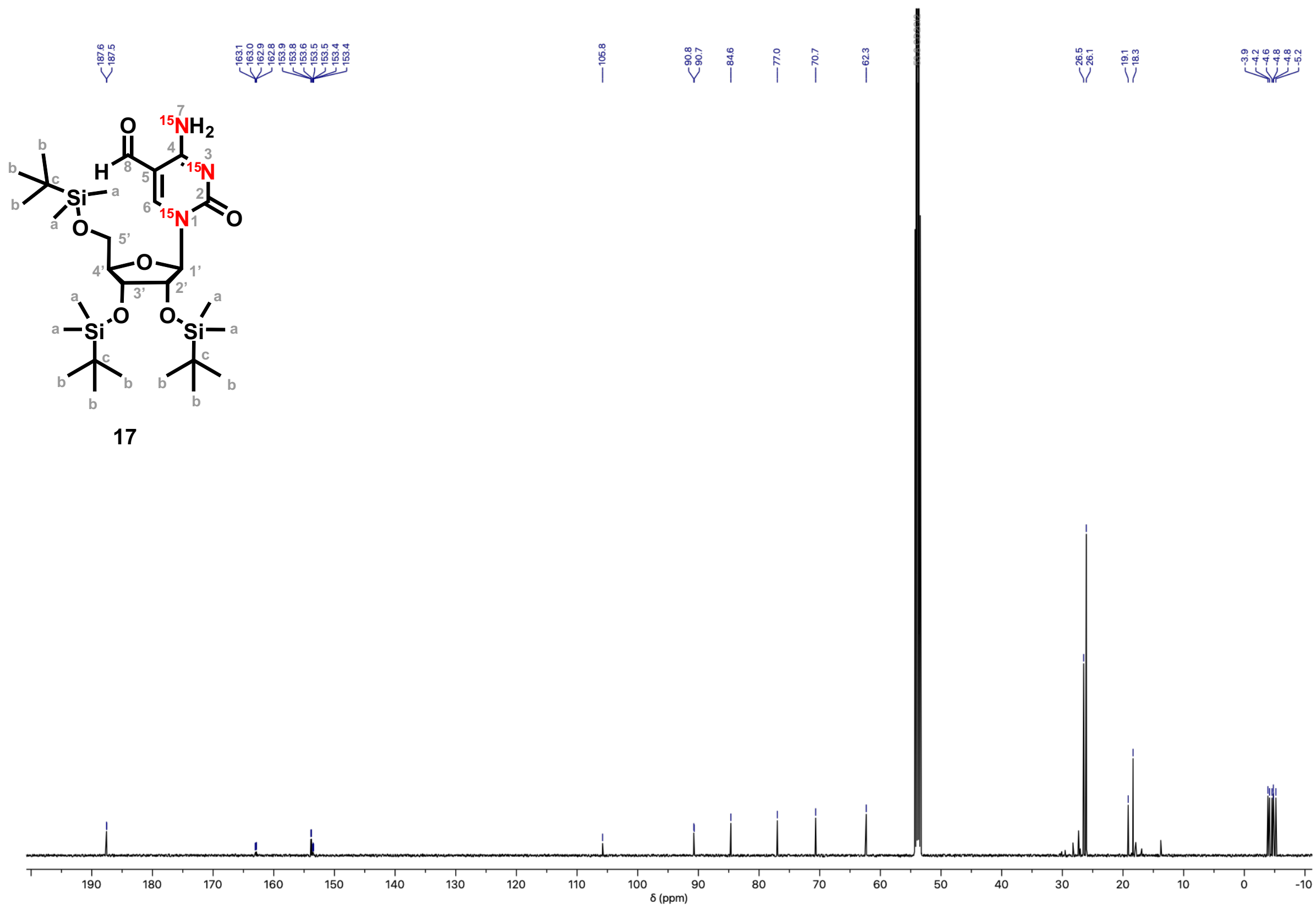

O2',3',5'-Tris(*tert*-butyldimethylsilyl)-5-hydroxymethyl-(N^1,N^3,N^4-^{15}N)-cytidine (18)

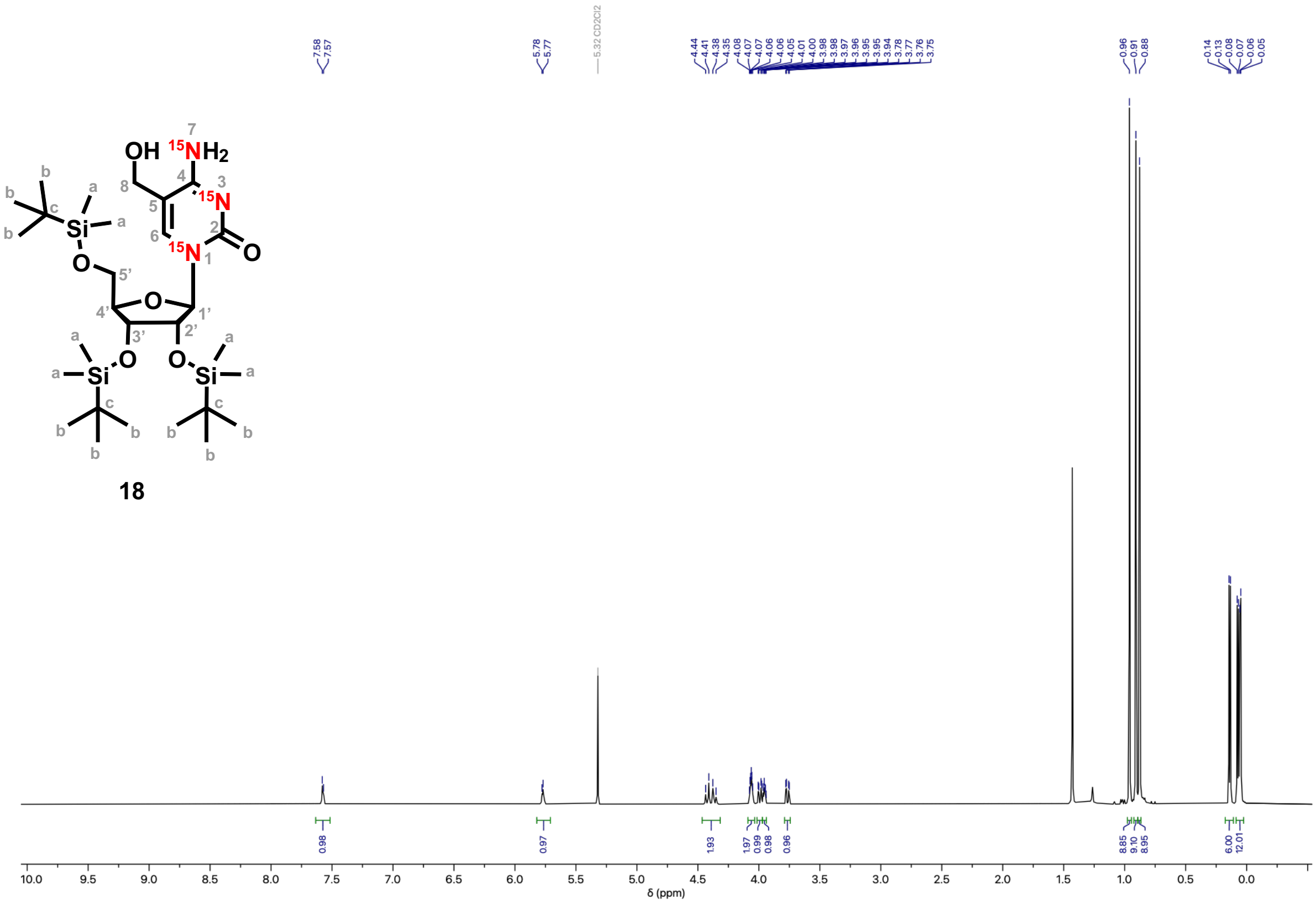

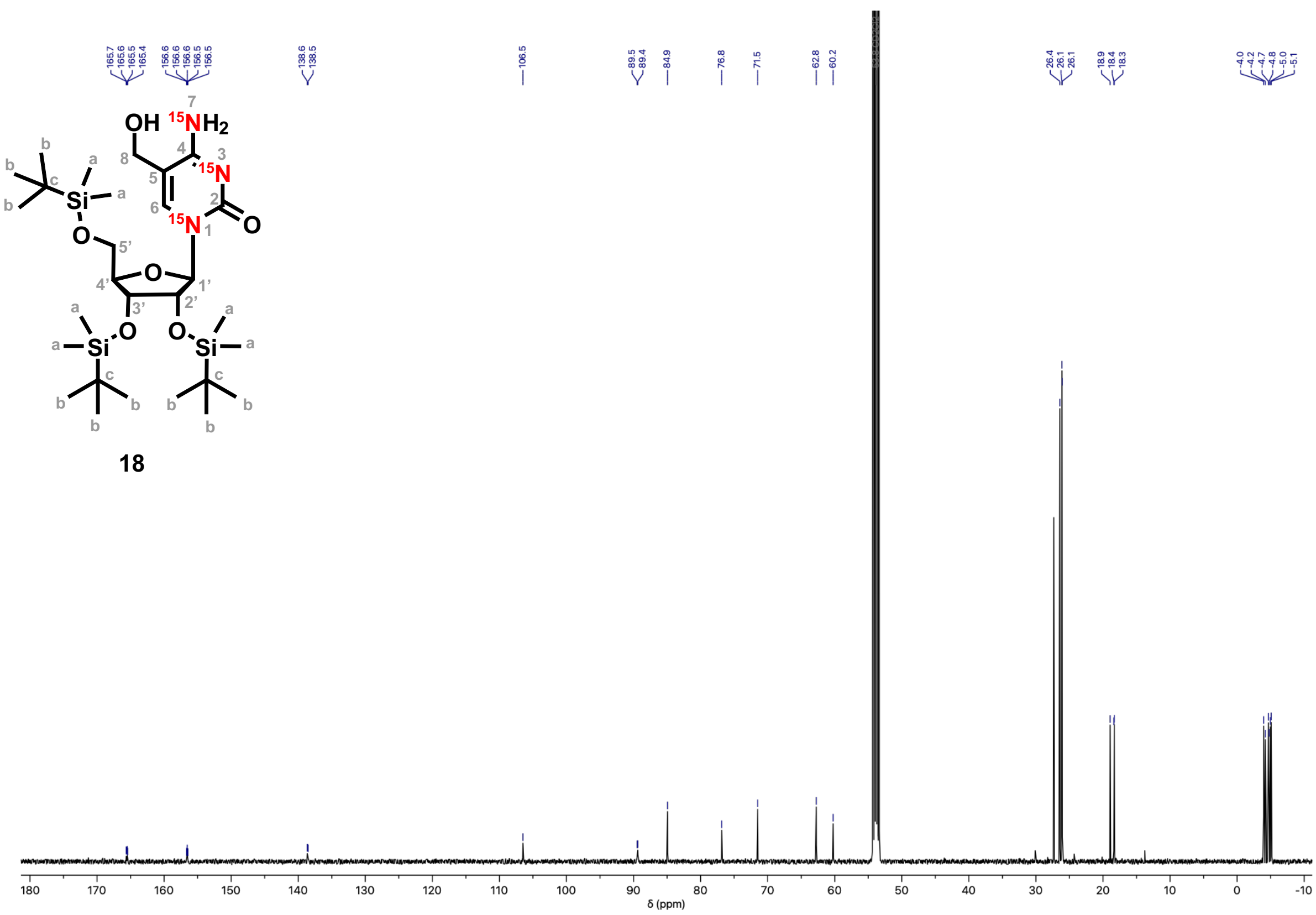

5-Hydroxymethyl-(N^1,N^3,N^4-^{15}N)-cytidine ([$^{15}N_3$]-hm5C)

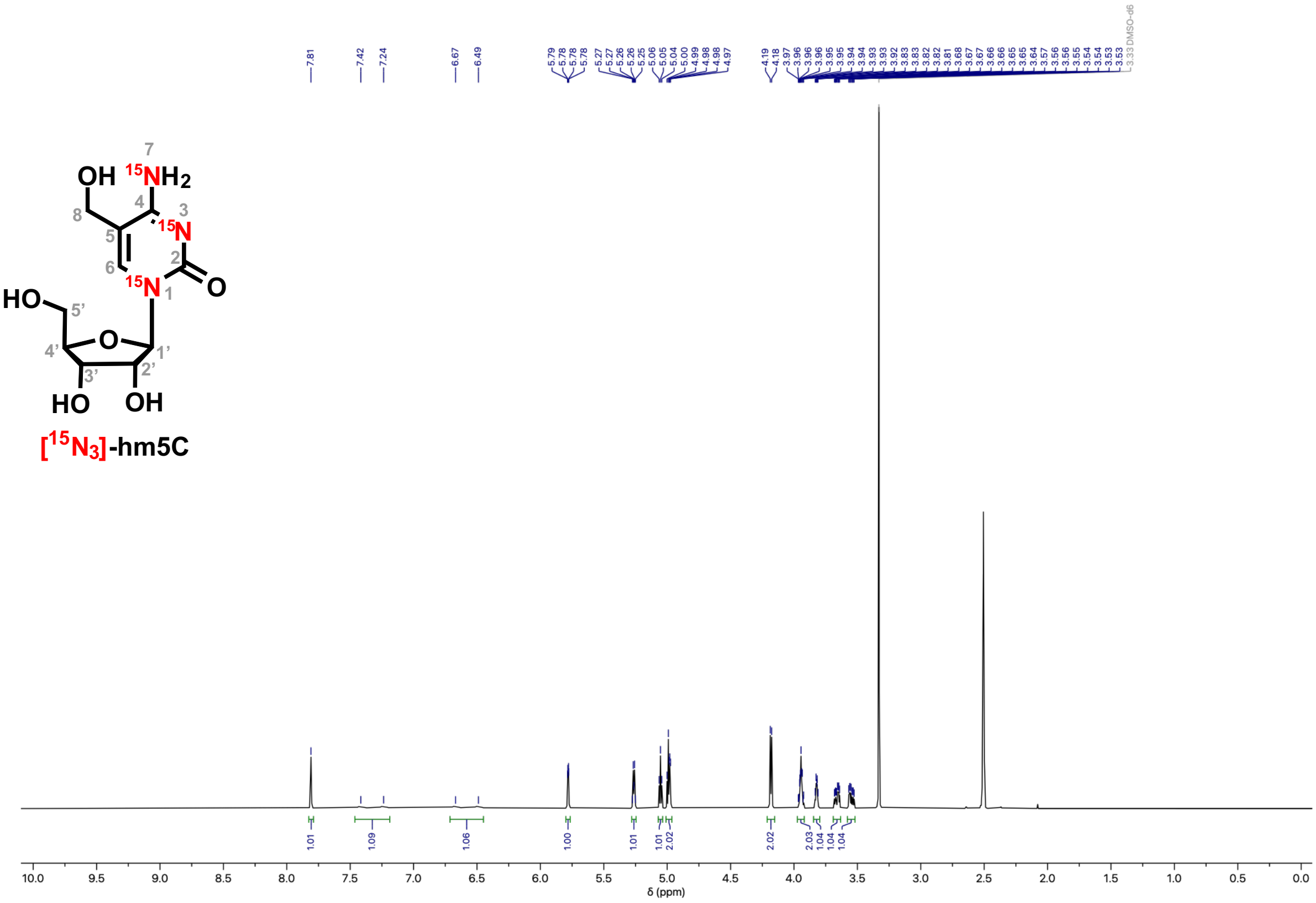

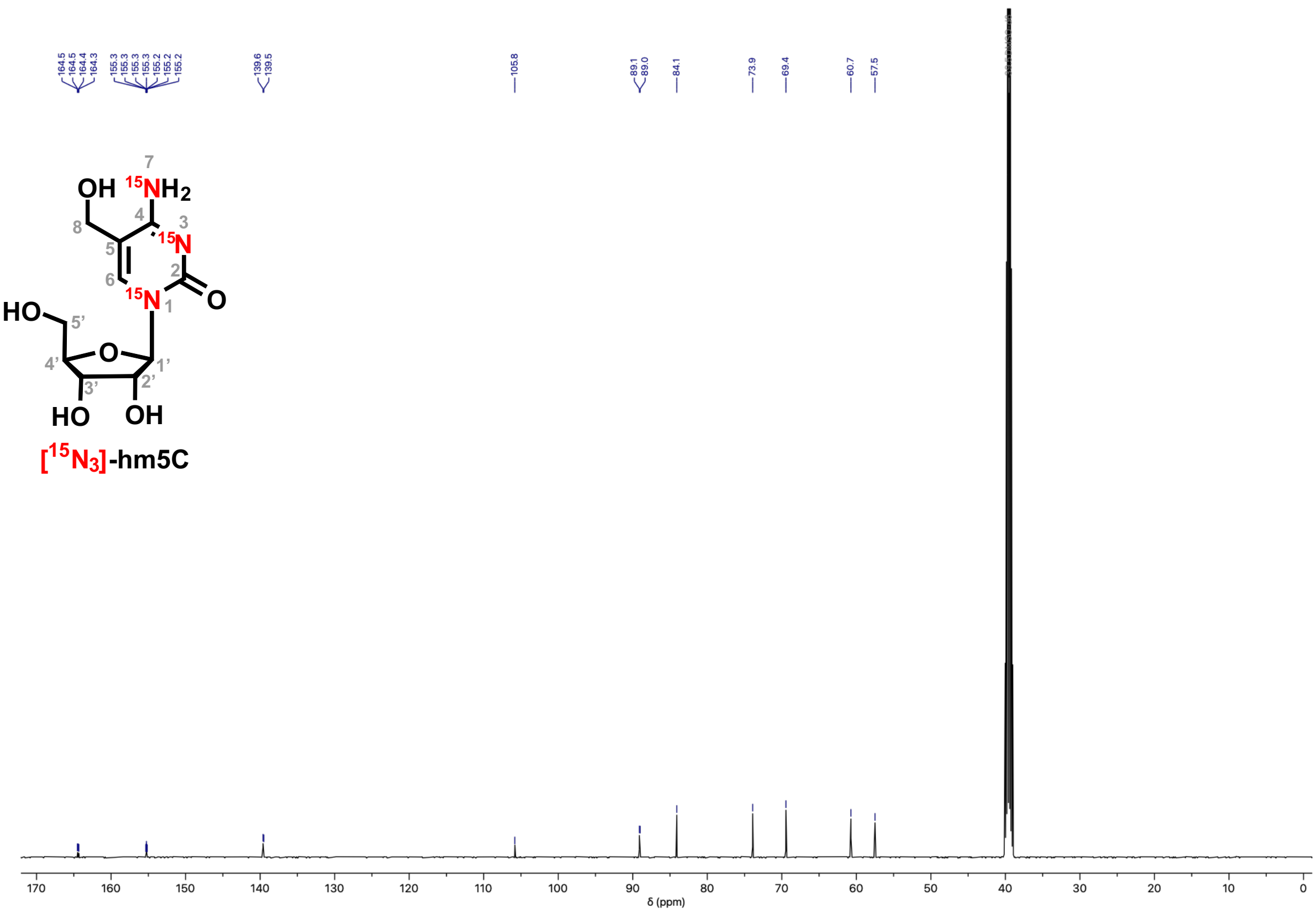

5-Formyl-(N^1,N^3,N^4-^{15}N)-cytidine ([$^{15}N_3$-f5C])

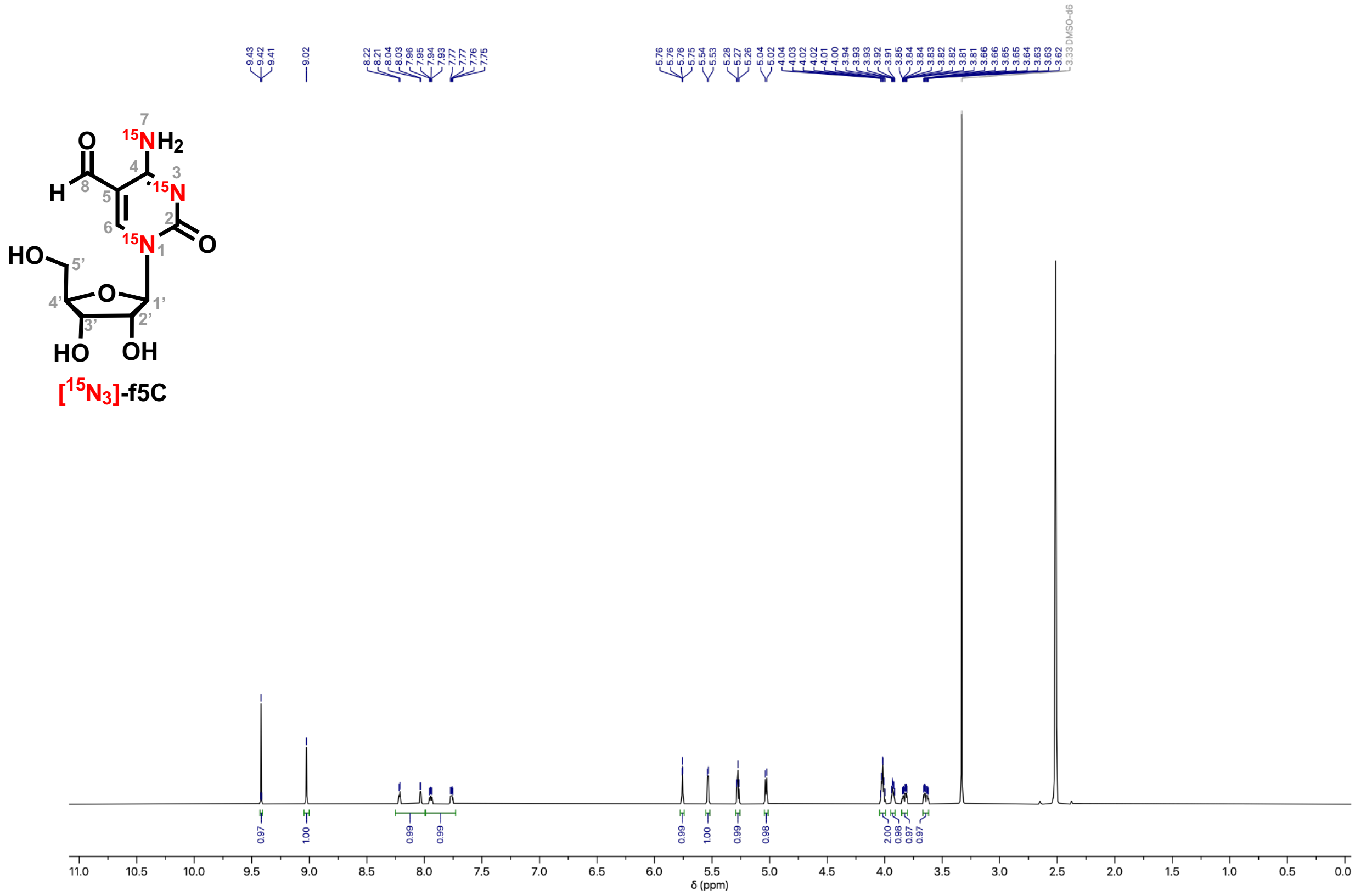

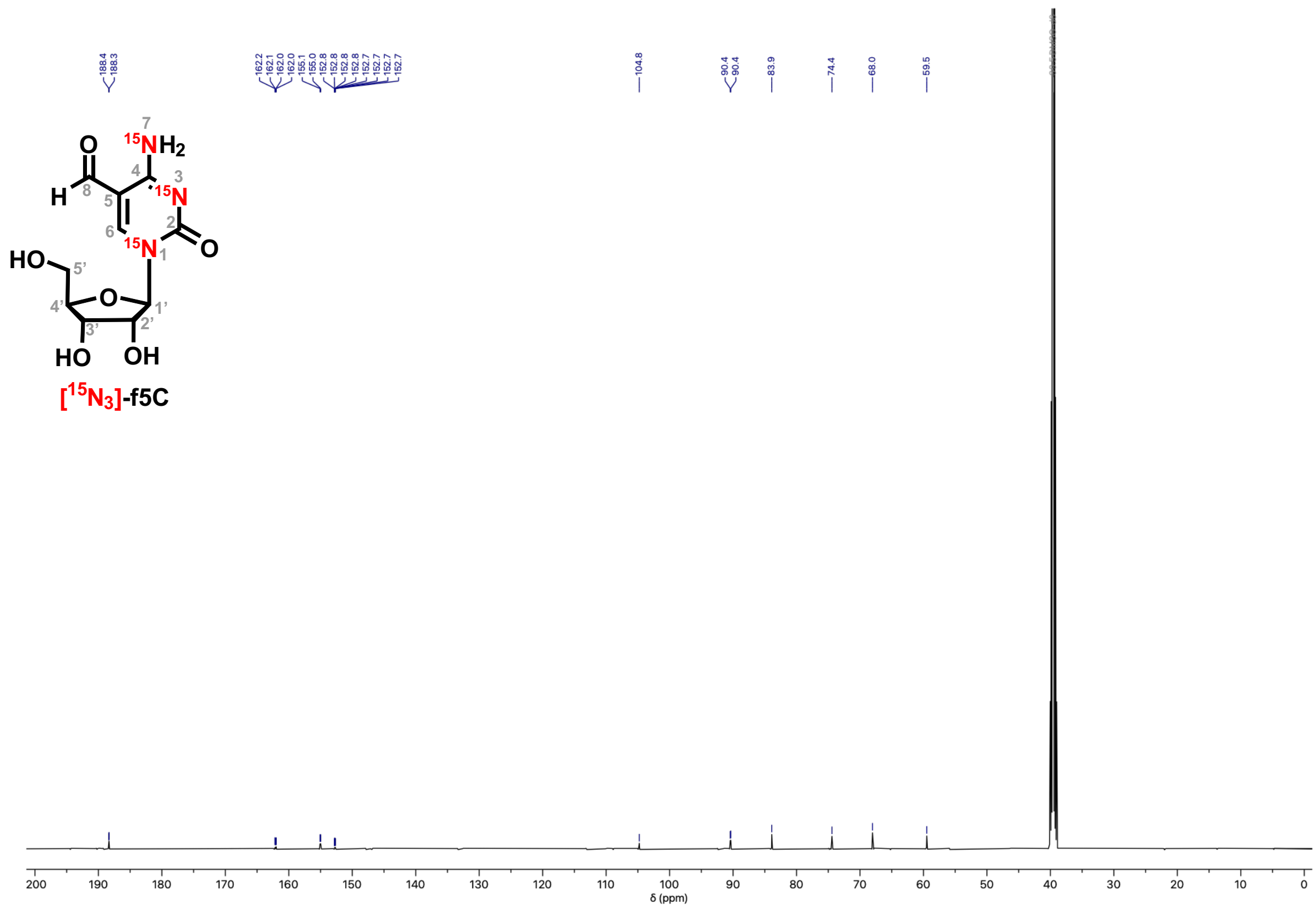

5-Carboxyl-(N^1,N^3,N^4-^{15}N)-cytidine ([$^{15}N_3$-ca5C])

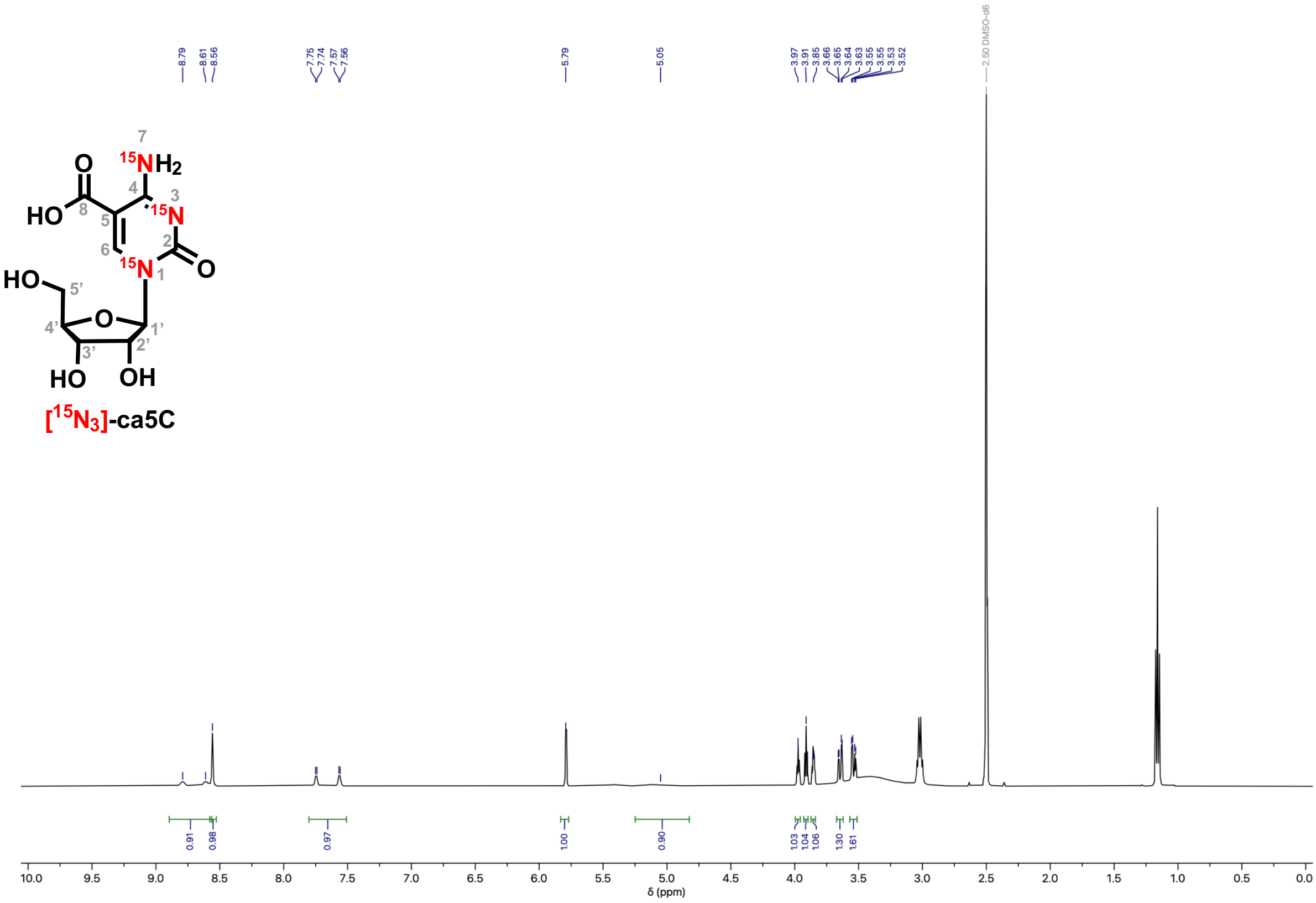

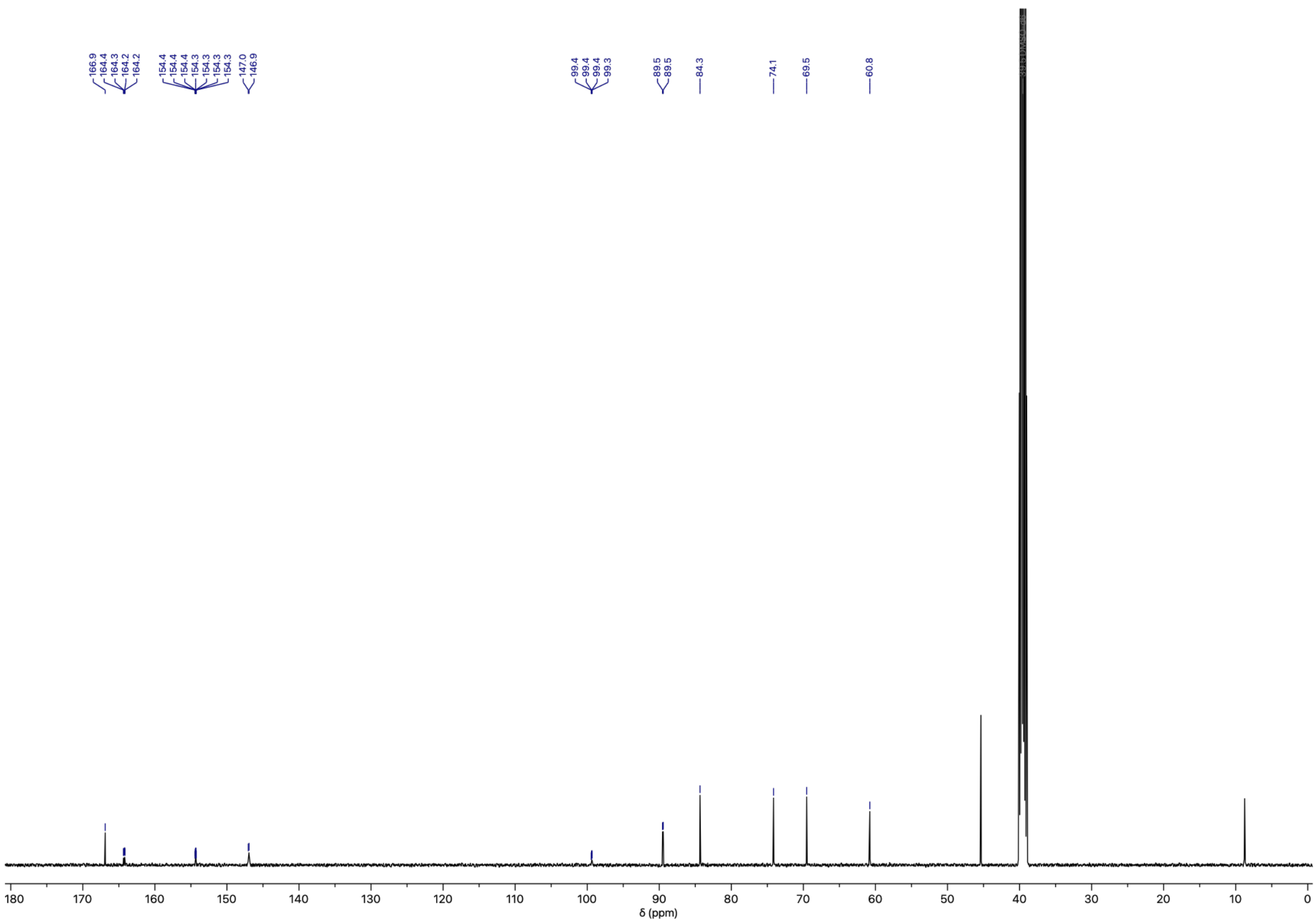

HR-ESI-MS

High-resolution electron spray mass spectrometry (HR-ESI-MS) was carried out by Dr. Werner Spahl in the Department of Chemistry at the Ludwig-Maximilians University. Mass spectra were recorded using a Thermo Finnigan LTQ FT, a Thermo Finnigan LTQ Orbitrap XL mass spectrometer or a Thermo Orbitrap Exploris 120. Depending on the sample, mass ranges of 40 u–2000 u were recorded. The resolution was set to 100,000 (for Orbitrap Exploris: 60,000) at m/z 400. The spray capillary voltage on the IonMax ESI head was 5 kV (for Orbitrap Exploris: 3 kV), the heater capillary temperature was 250 °C (for Orbitrap Exploris: 300 °C), the nitrogen sheath gas flow was 25 units, and the sweep gas flow was 5 units.

All measured mass spectrometry values are reported in the mass-to-charge ratio (m/z). The calculated exact mass of each compound is based on the most abundant isotope (except for heavy labelled compounds) of each containing nuclide (^{1}H, ^{12}C, ^{14}N, ^{16}O, ^{23}Na, ^{28}Si, ^{35}Cl, ^{31}P, ^{32}S and ^{127}I). The abbreviation "M" was used for the compound's molecular formula to assign detected peaks in the mass spectra.

*O*2',3',5'-Tris(*tert*-butyldimethylsilyl)-cytidine (1)

585, c27h55n3o5si3, geloest, Chloroform LTQ FT Ultra

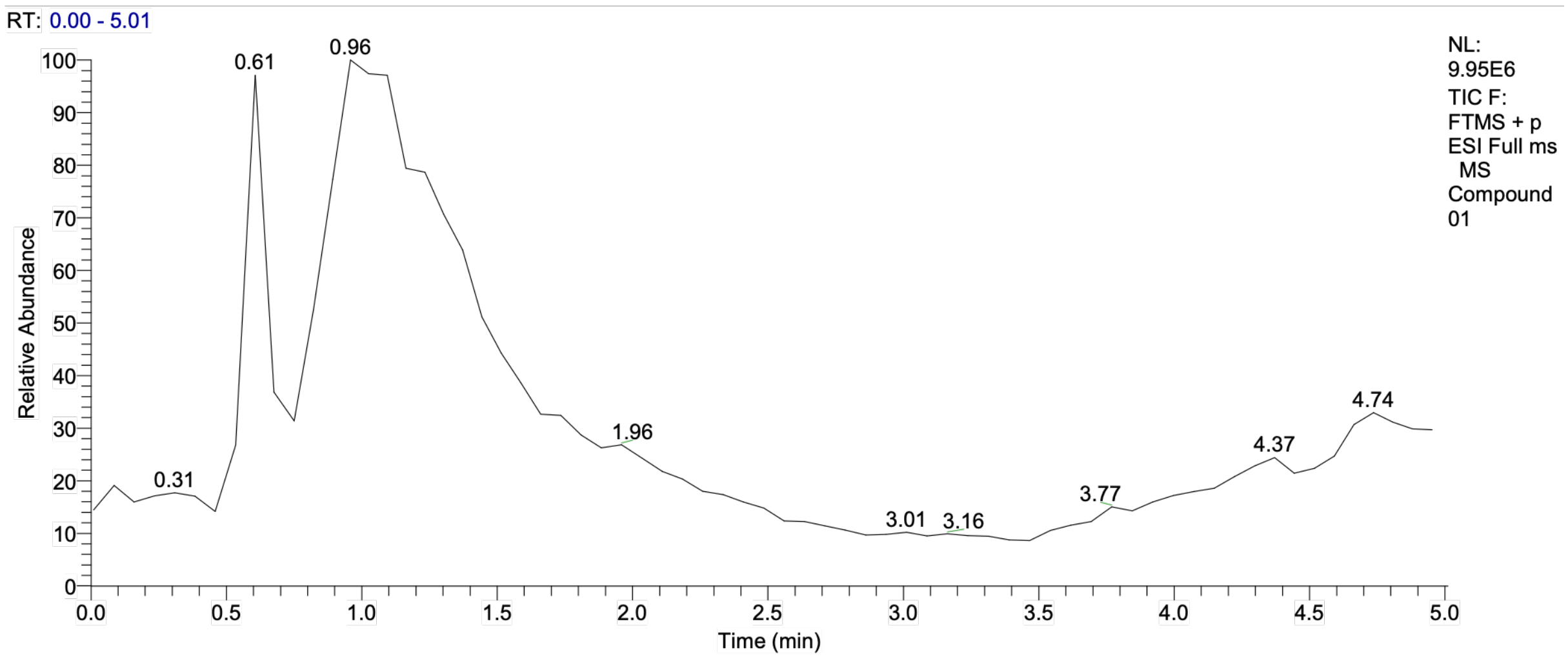

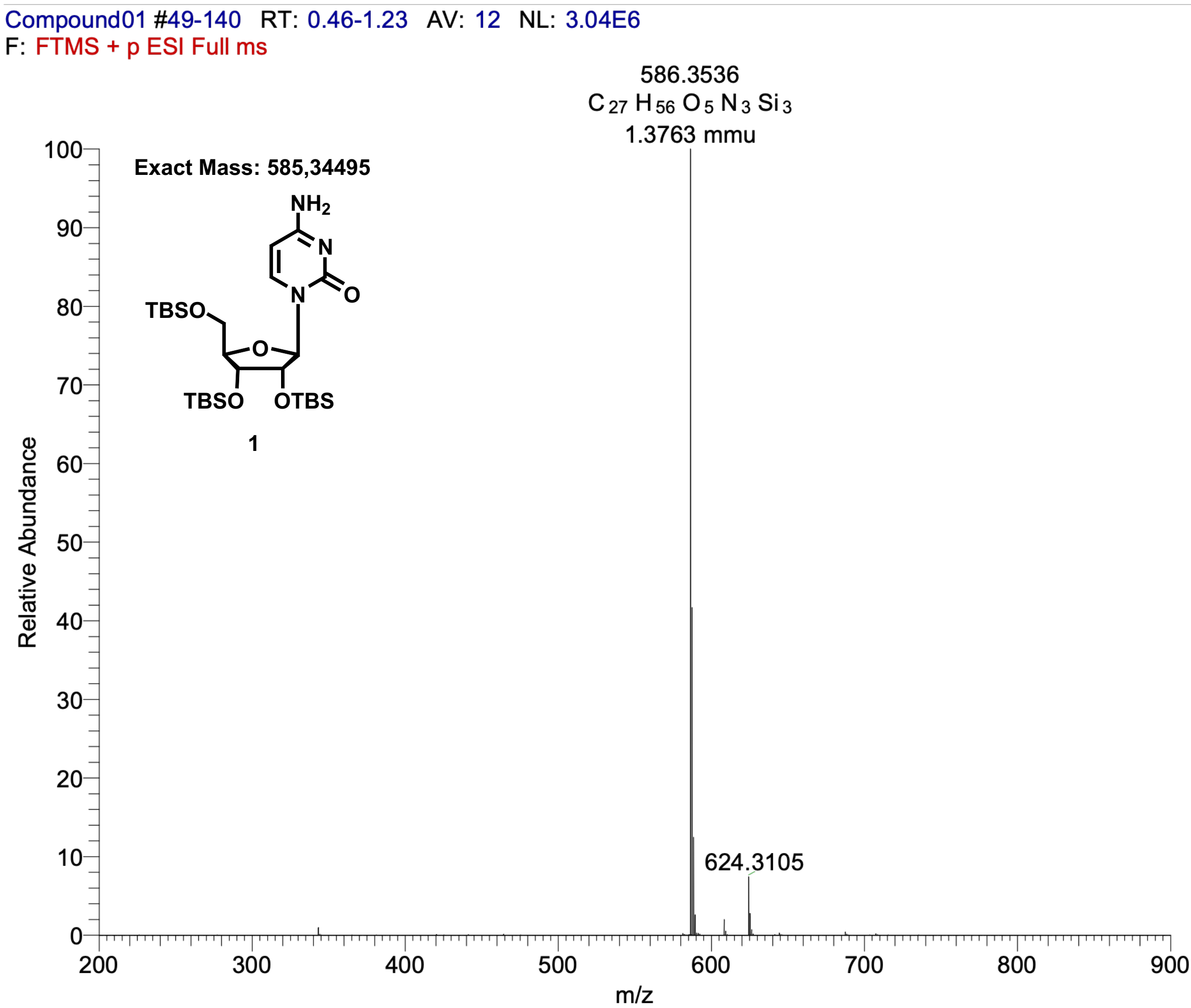

*O*2',3',5'-Tris(*tert*-butyldimethylsilyl)-5-iodo-cytidine (2)

711, c27h54in3o5si3, fest, Aceton LTQ FT Ultra

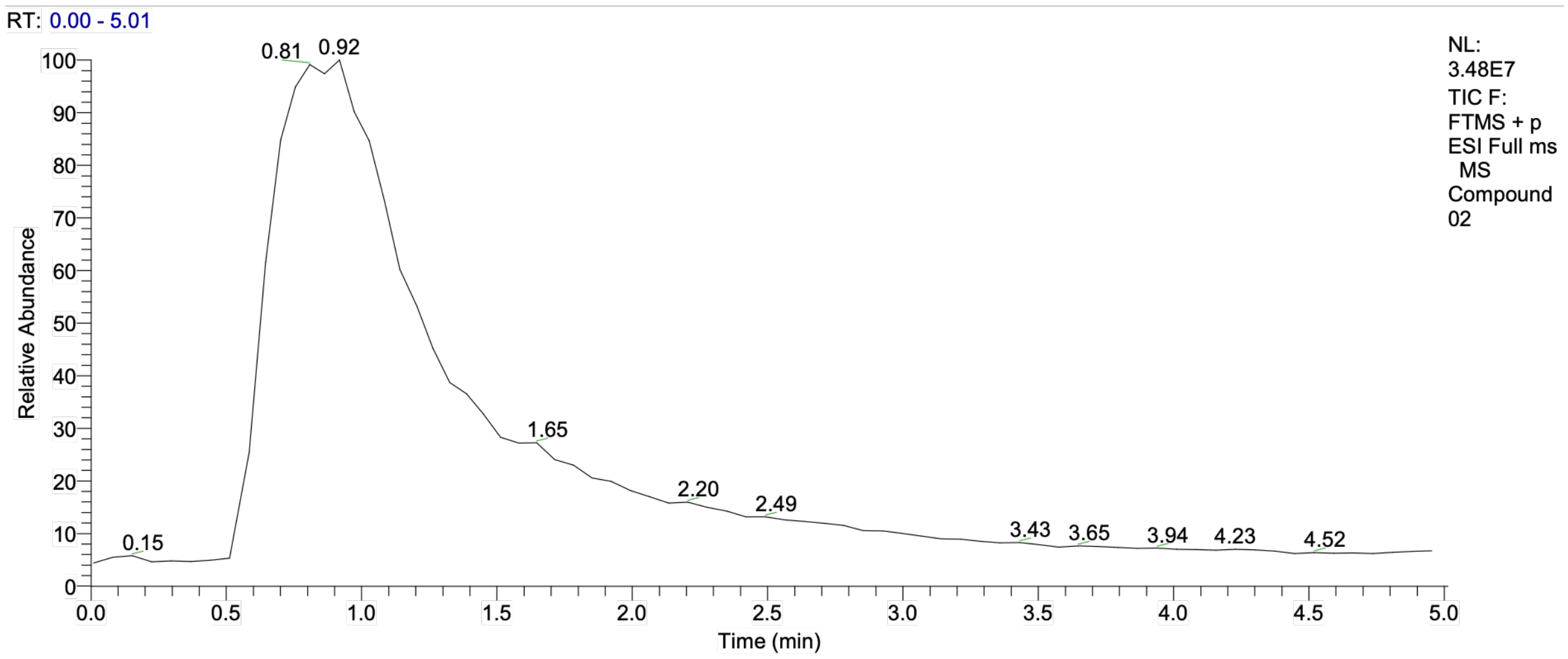

Compound02 #156-247 RT: 1.26-1.92 AV: 11 NL: 3.41E6
F: FTMS + p ESI Full ms

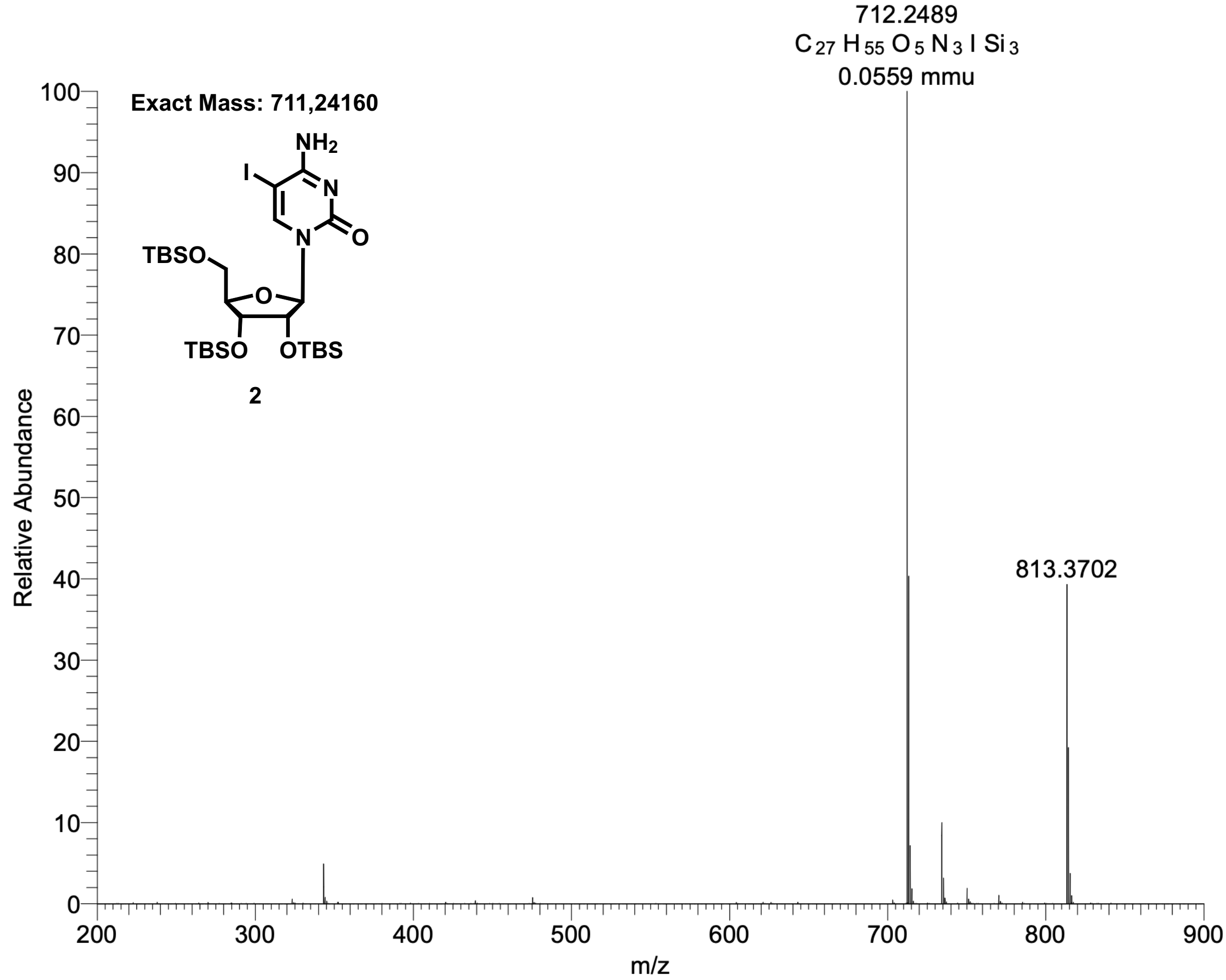

*O*2',3',5'-Tris(*tert*-butyldimethylsilyl)-5-(methoxycarbonyl)-cytidine (4)

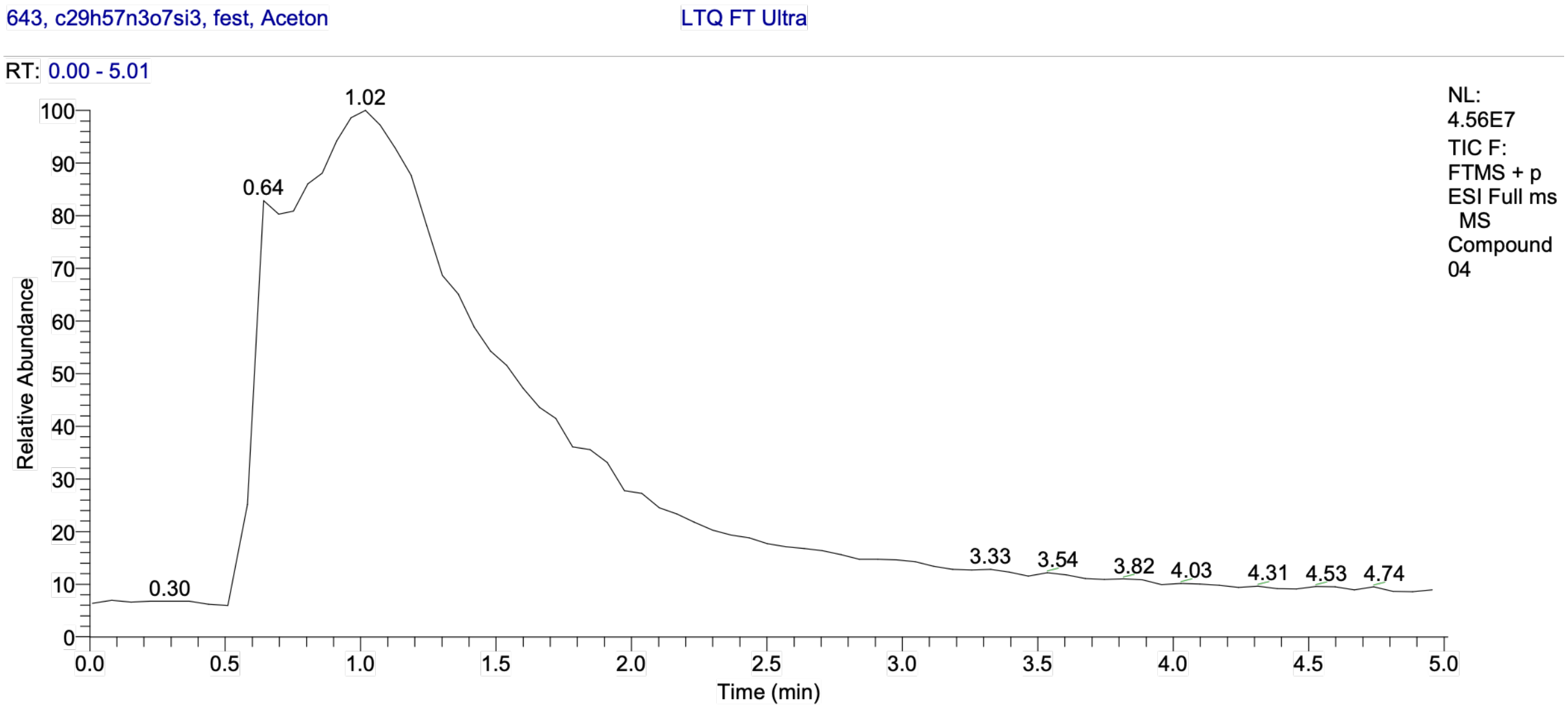

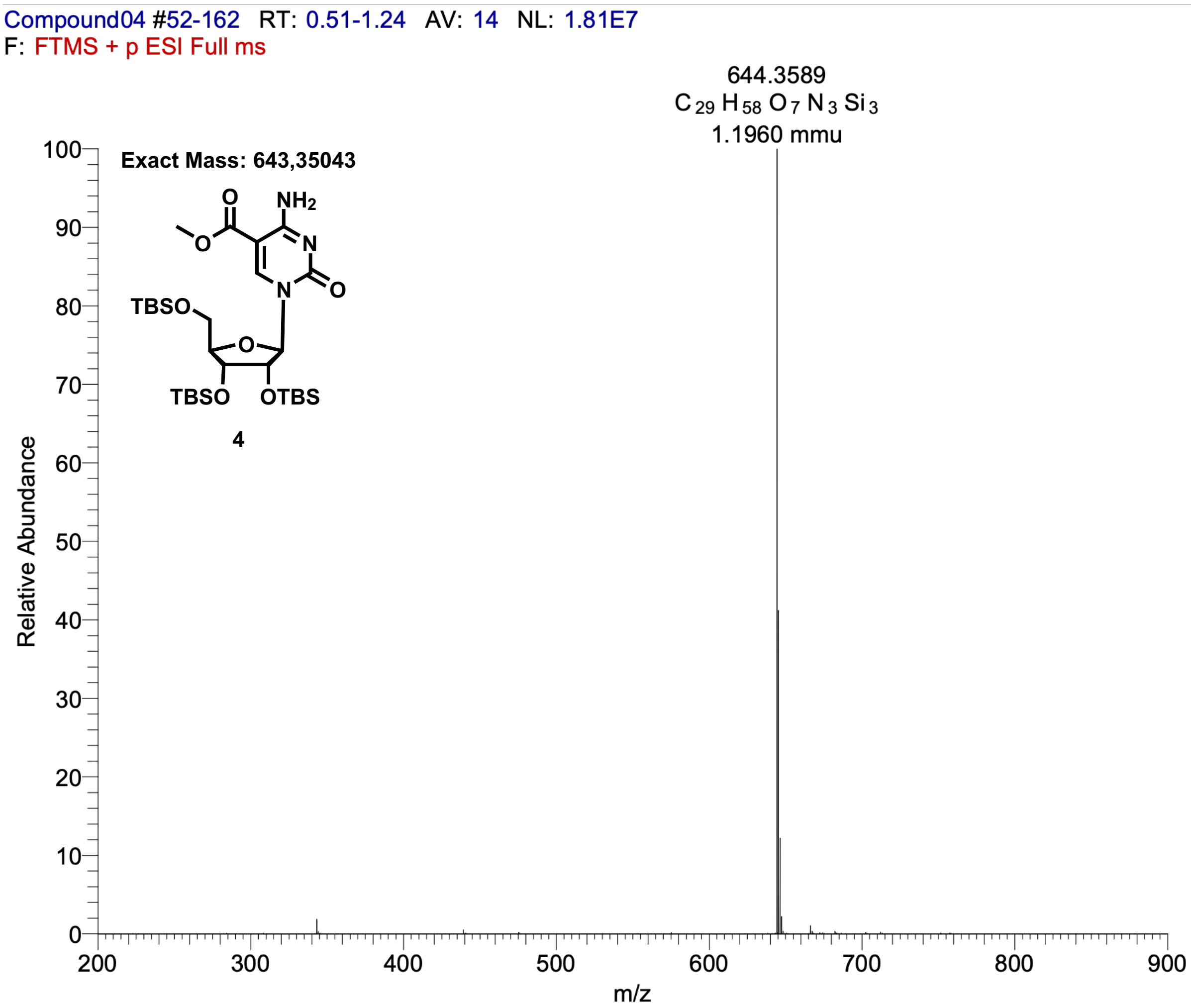

*O*2',3',5'-Tris(*tert*-butyldimethylsilyl)-5-methyl-cytidine (7)

600, c28h56n2o6si3, geloest, Methanol LTQ FT Ultra

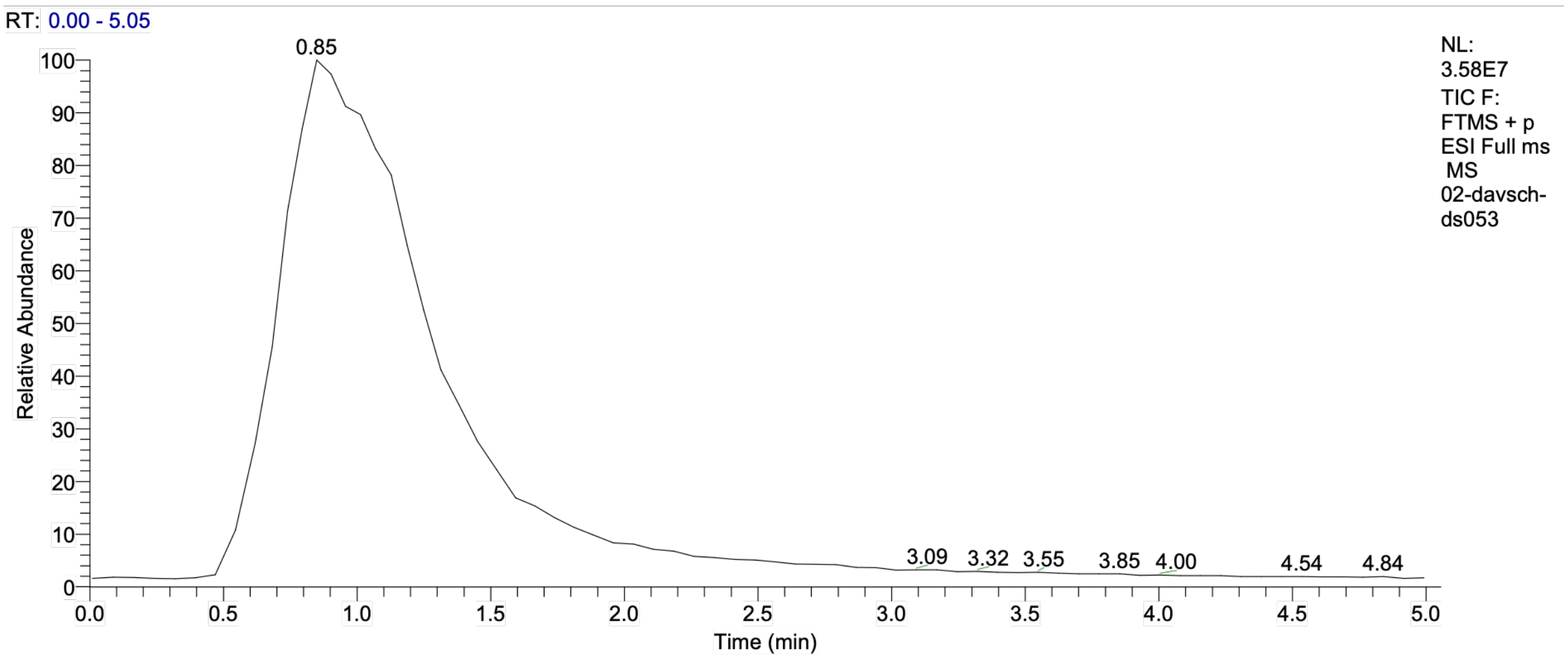

02-davsch-ds053 #144-244 RT: 1.19-2.03 AV: 13 NL: 1.03E6
F: FTMS + p ESI Full ms

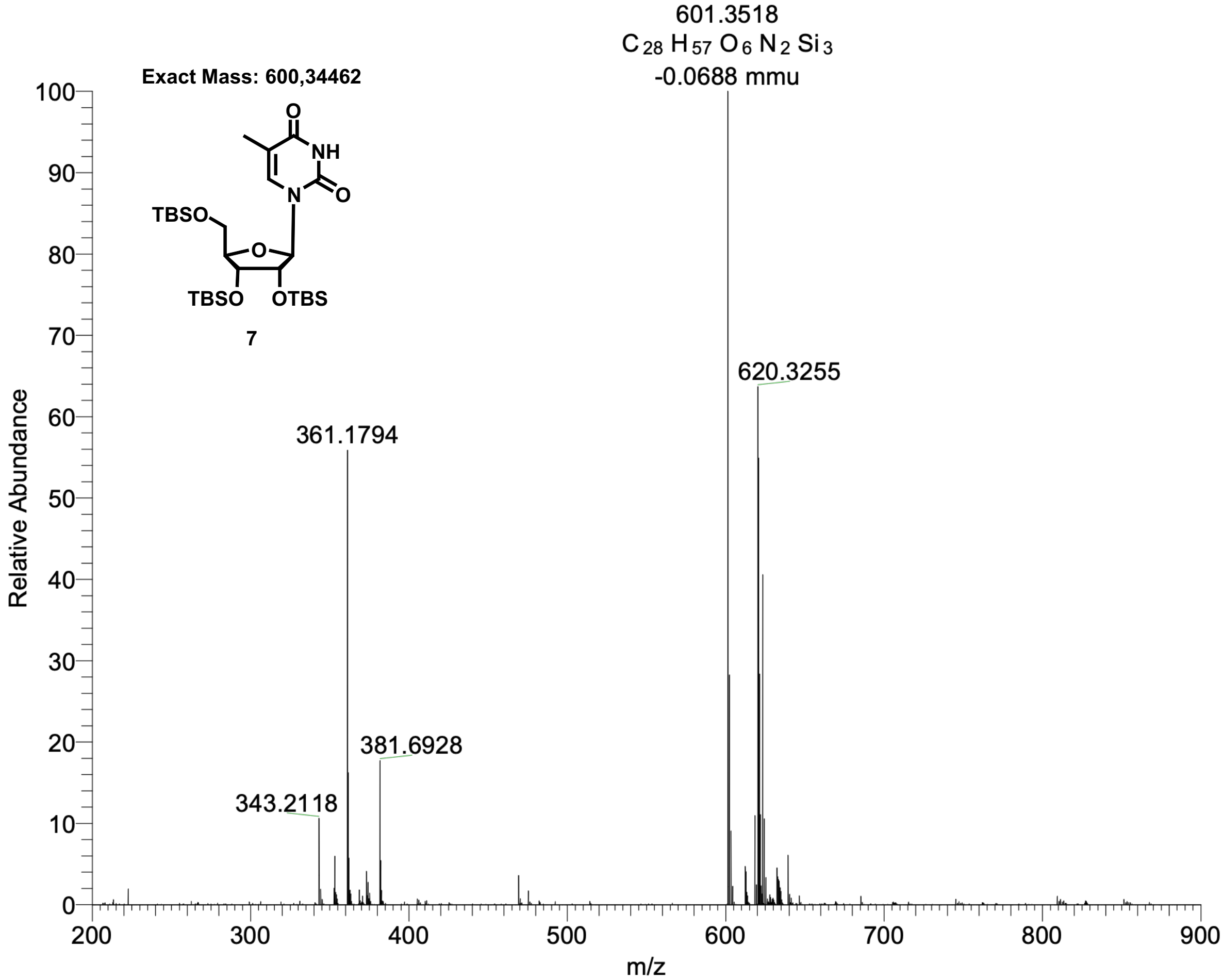

_O_2',3',5'-Tris(_tert_-butyldimethylsilyl)-_O_4-(2,4,6-Tri(iso-propyl)phenylsulfonyl)-5-methyl-cytidine (8)

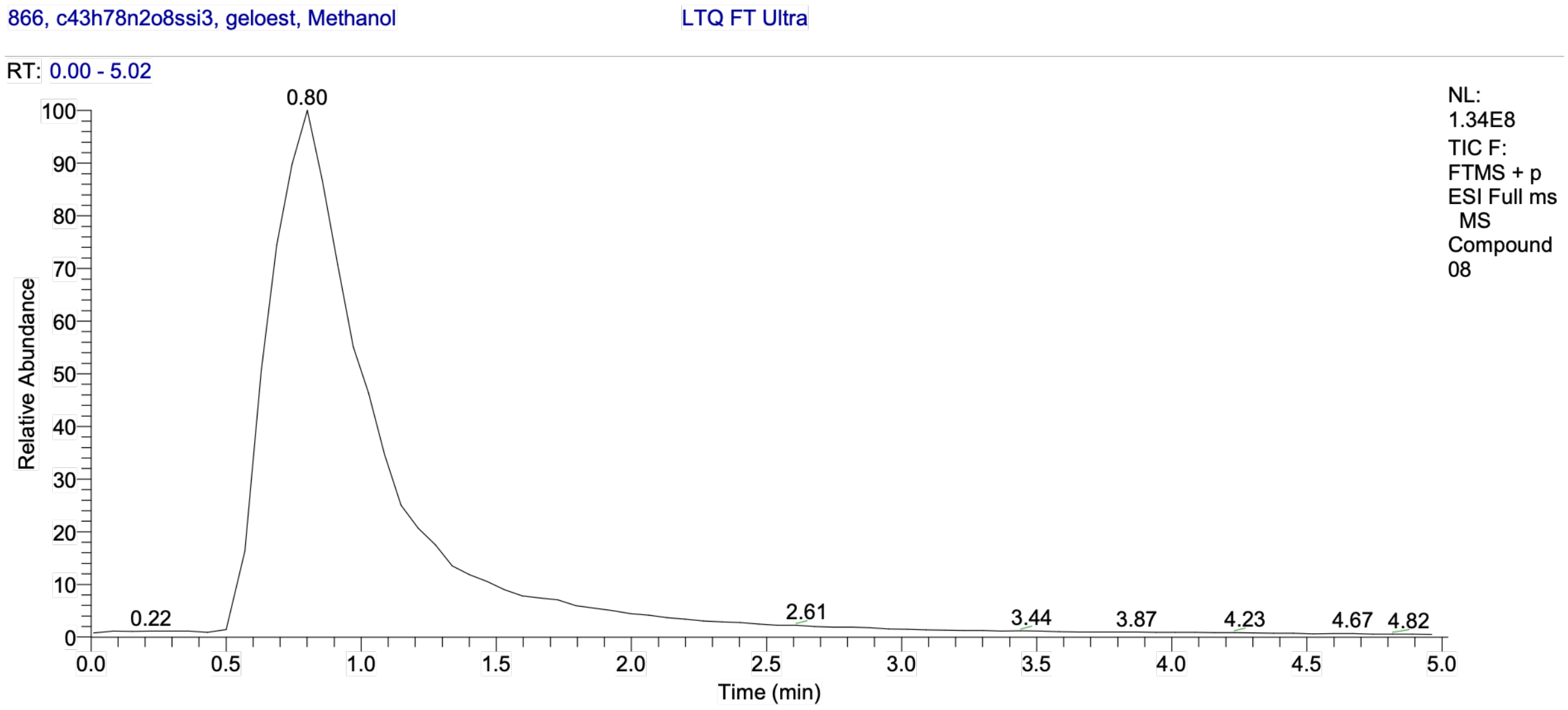

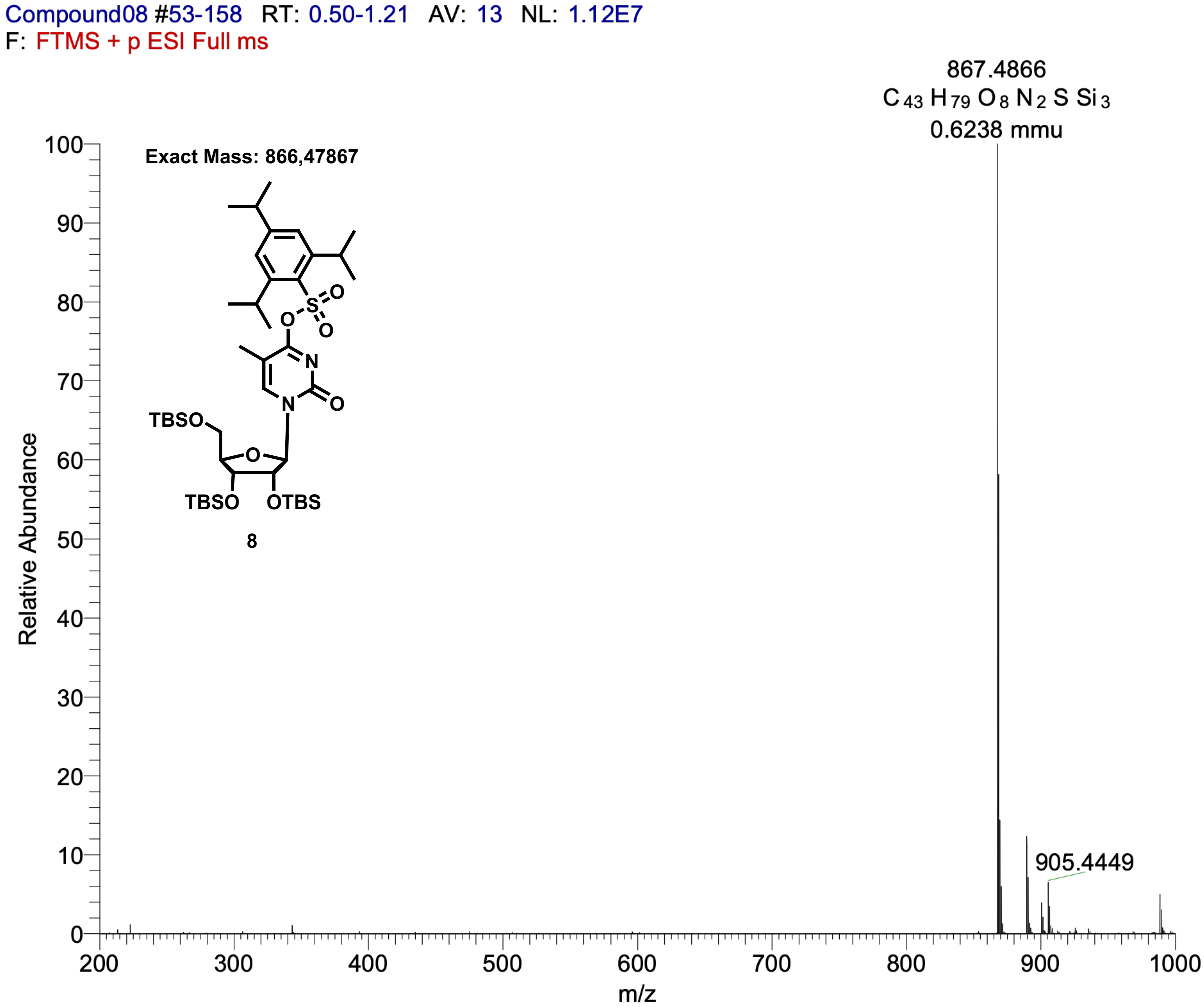

5-methyl-cytidine (m5C)

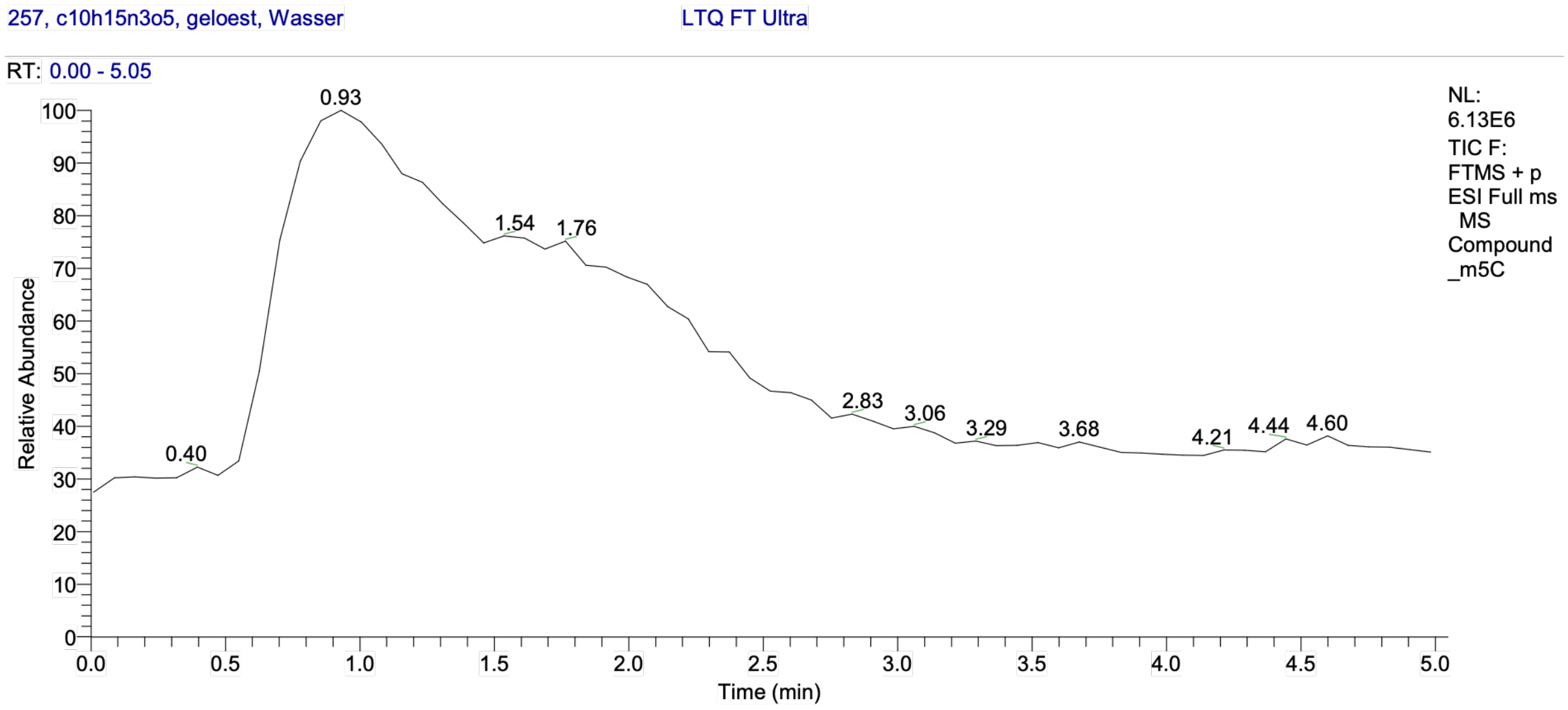

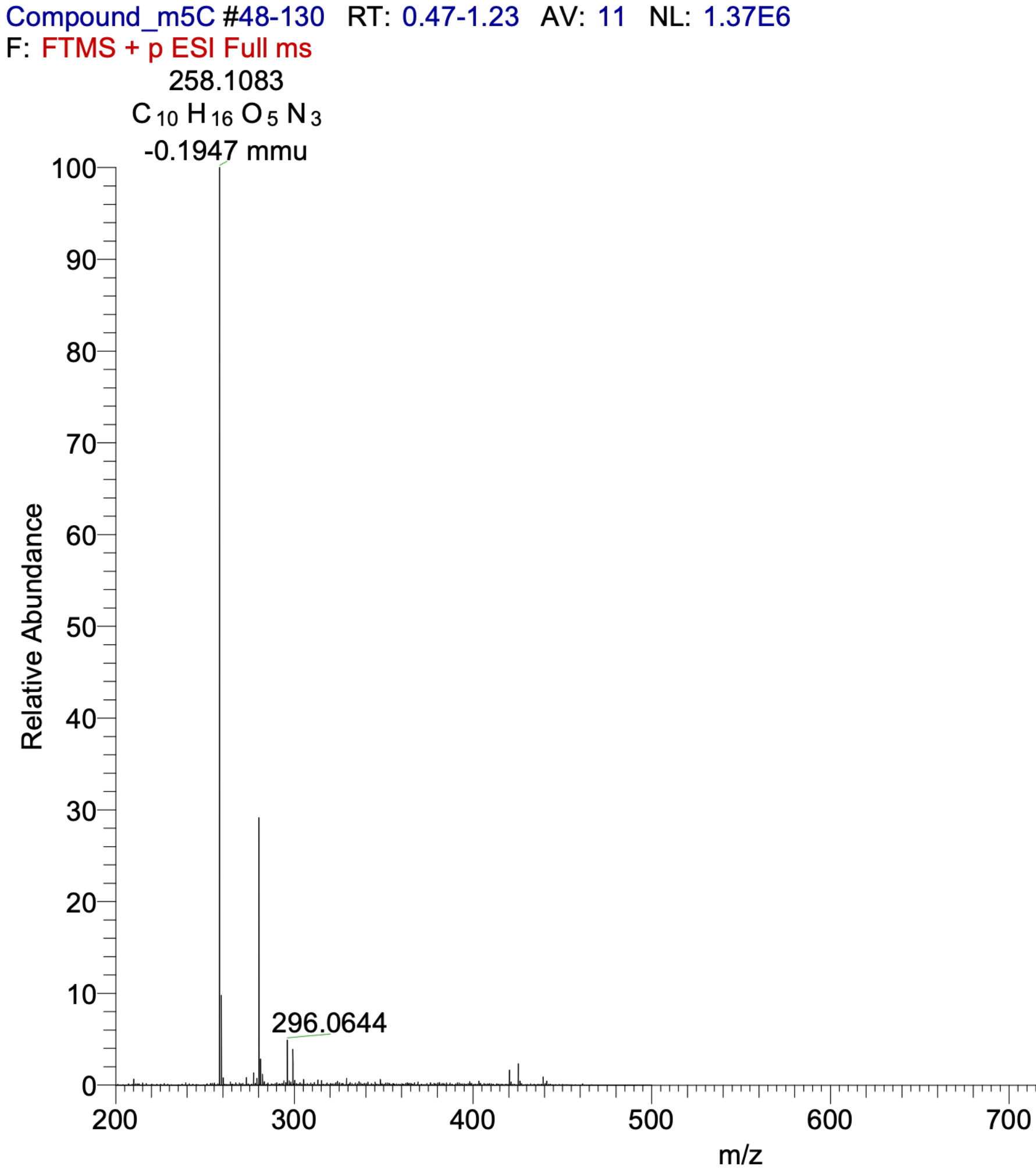

Exact Mass: 257.10117

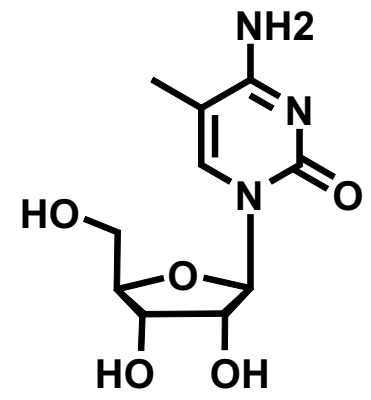

m5C

5-Hydroxymethyl-cytidine (hm5C)

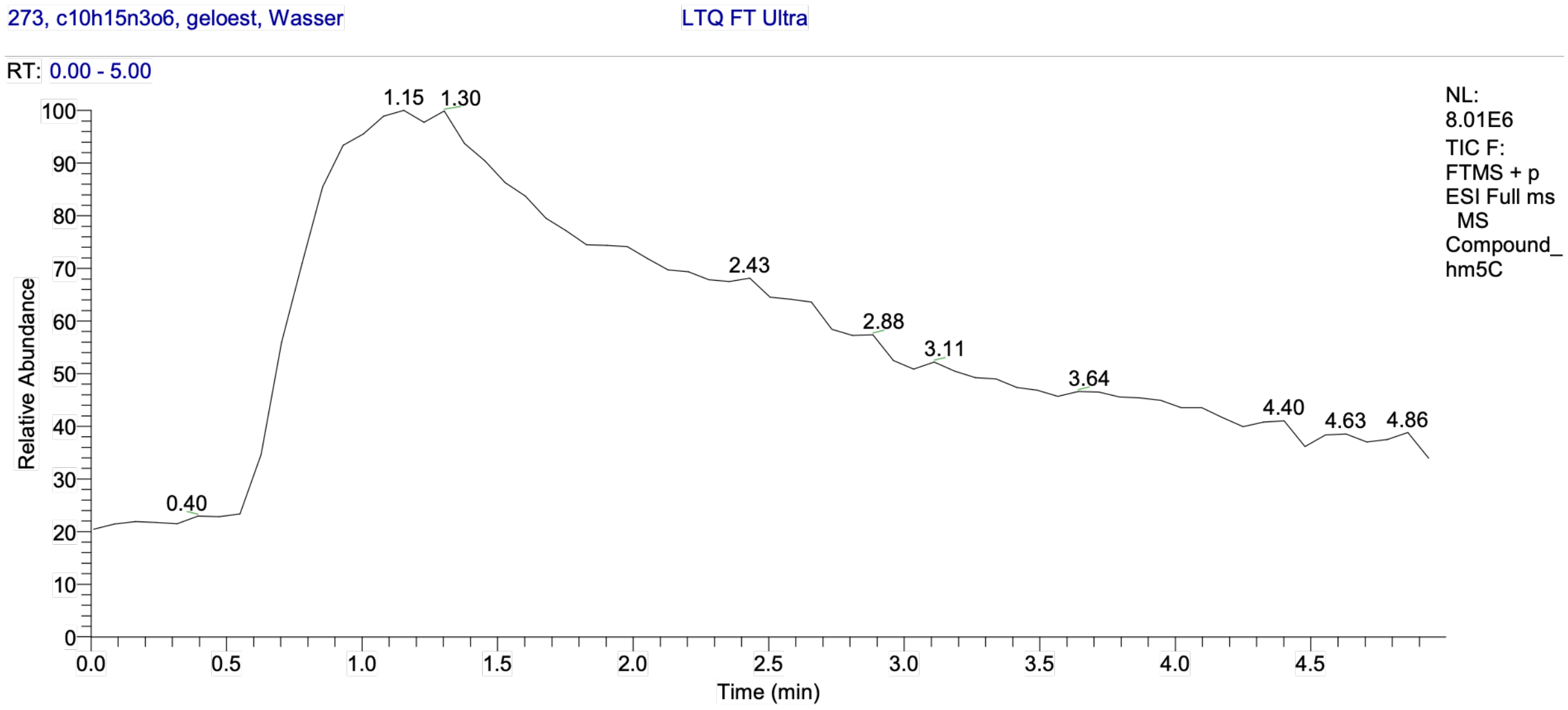

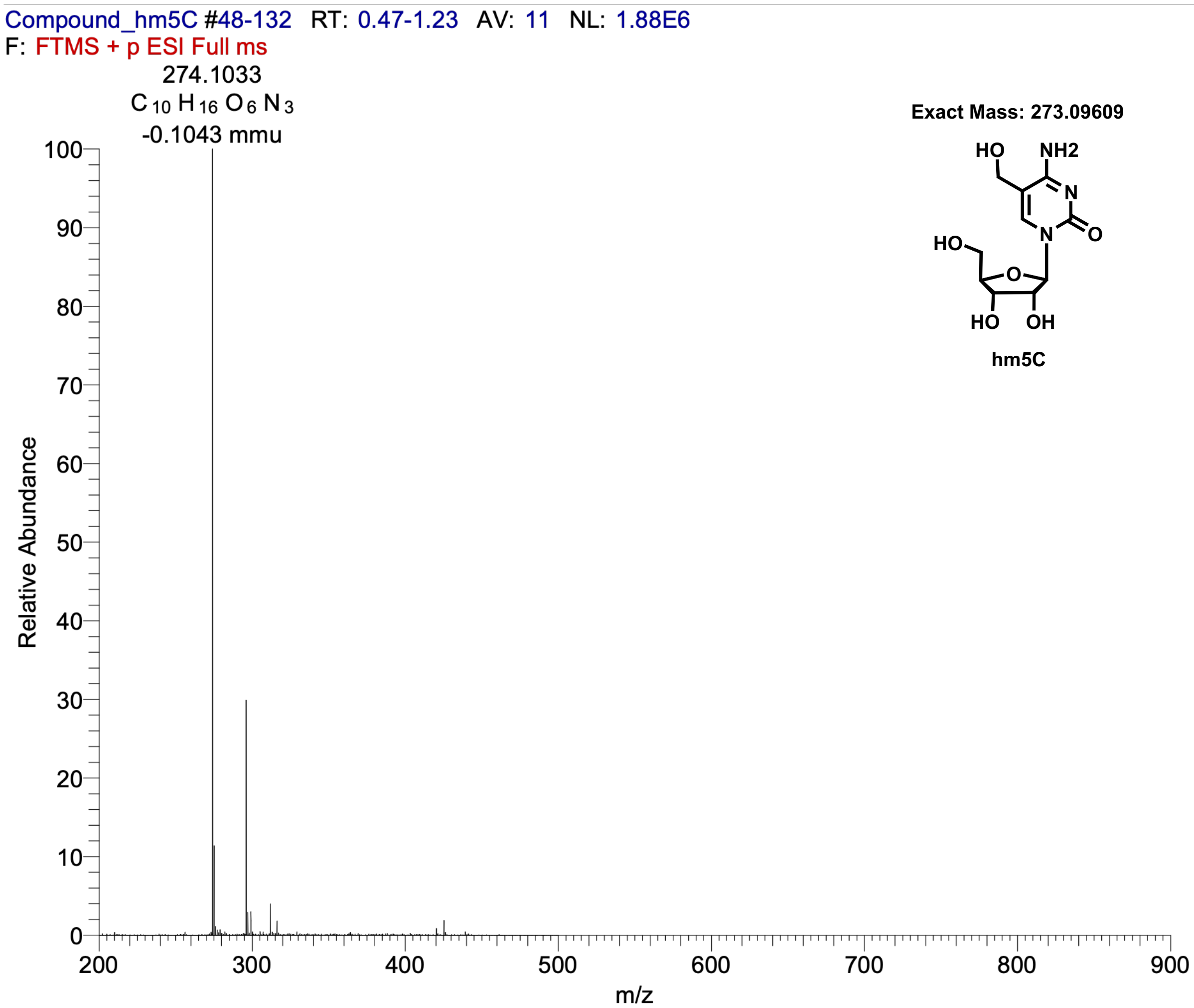

5-Formyl-cytidine (f5C)

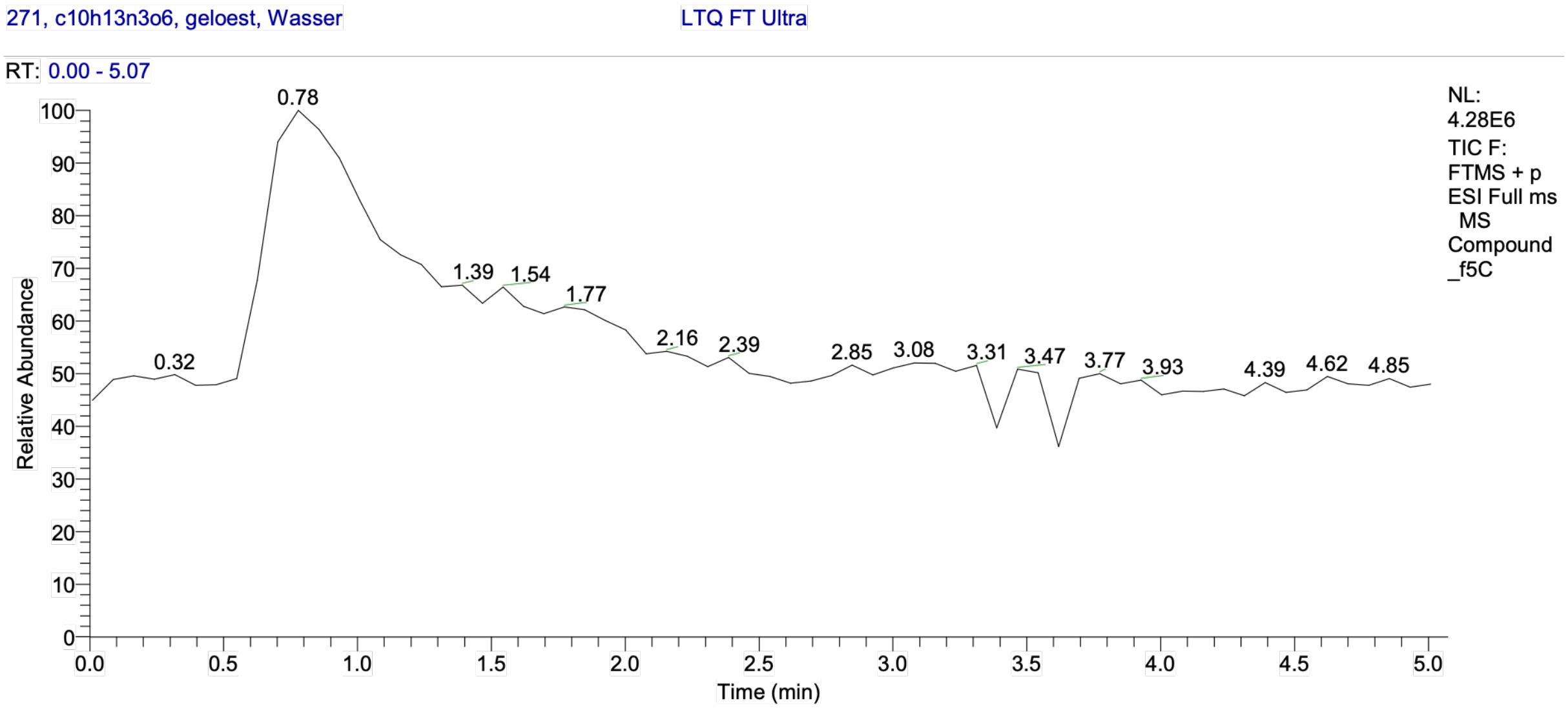

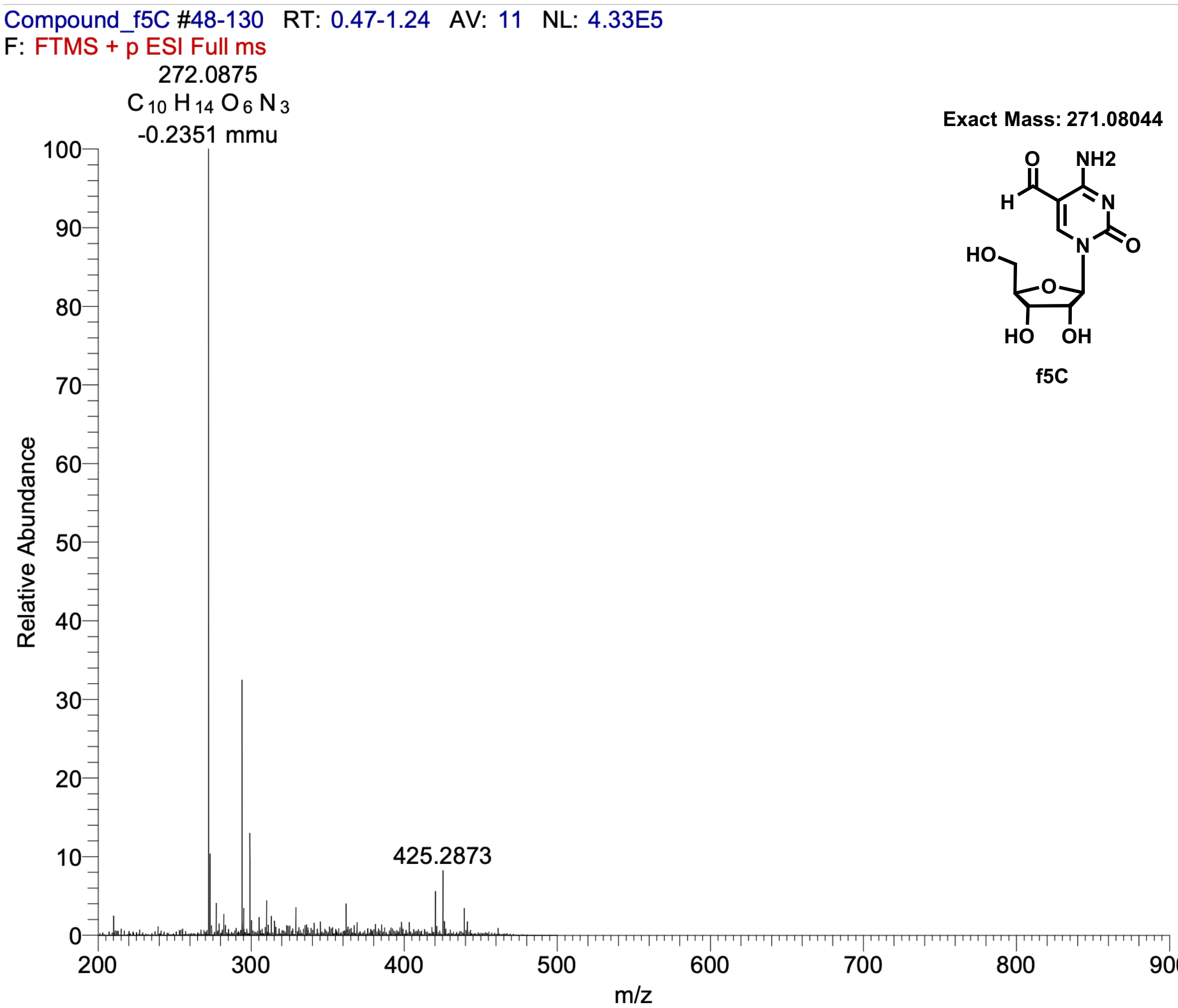

5-Carboxyl-cytidine (ca5C)

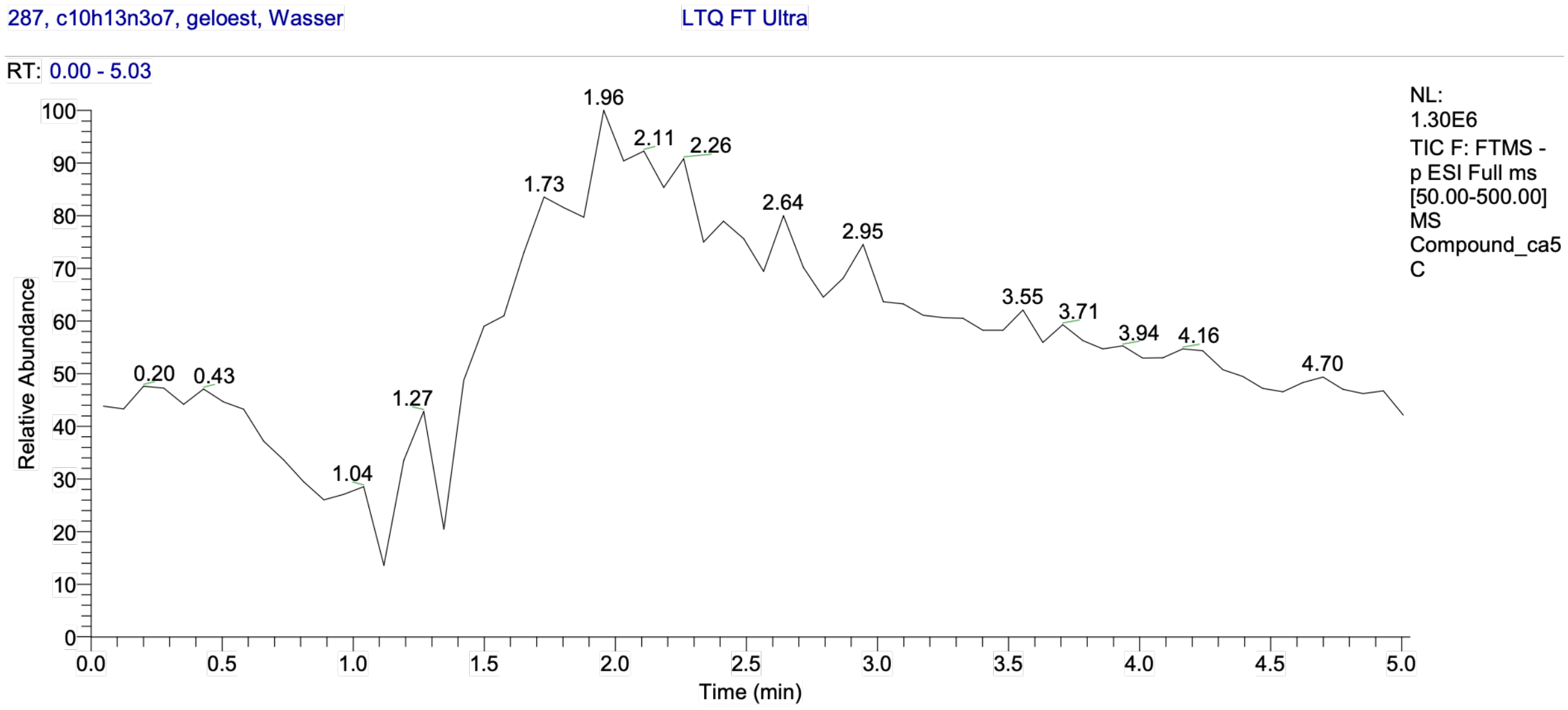

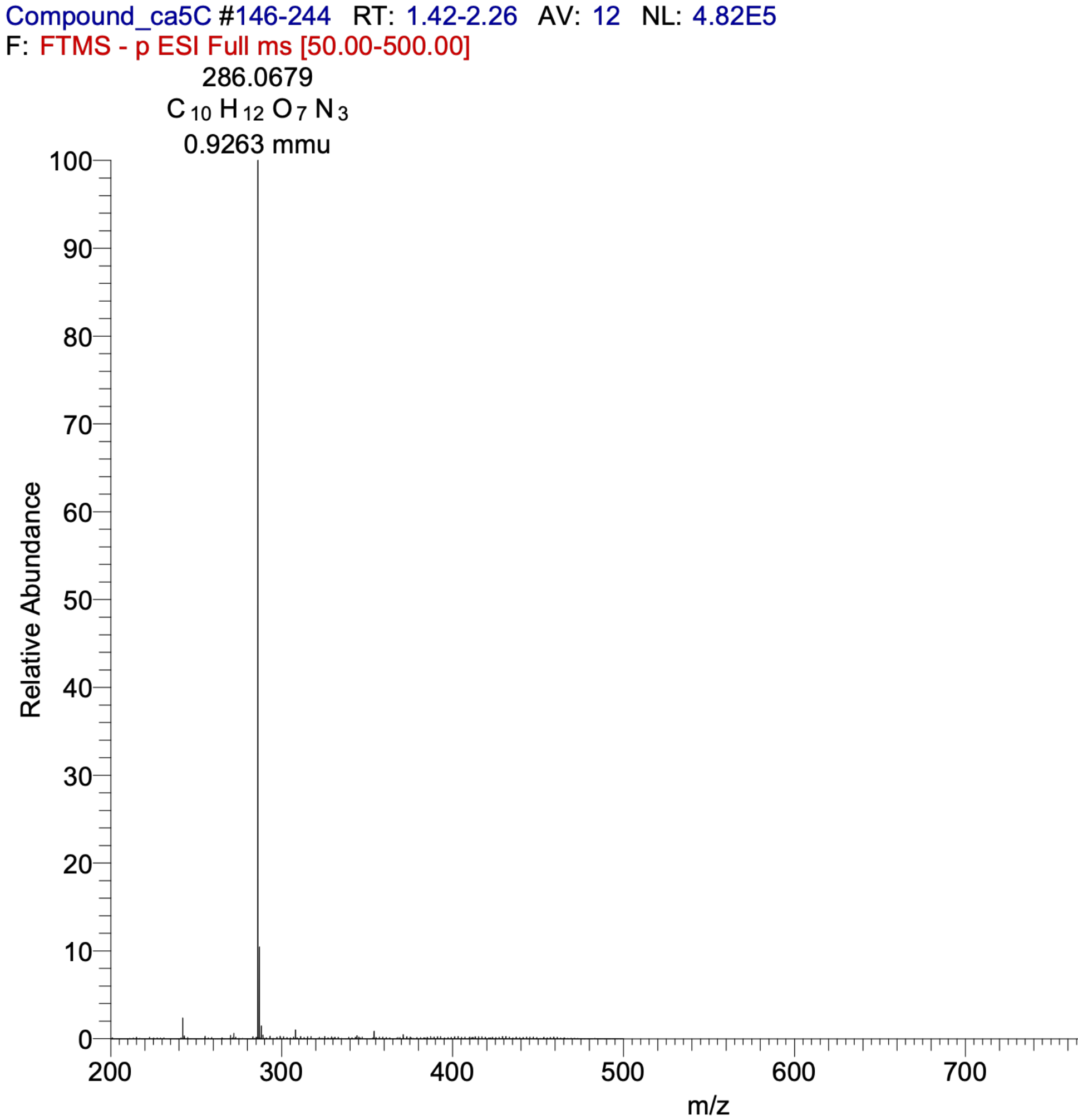

Exact Mass: 287.07535

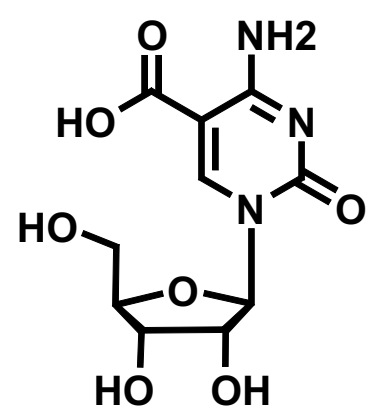

ca5C

Uracil-[$^{15}N_2$] (10)

114, c4h4(15n2)o2, fest, Wasser — LTQ Orbitrap XL

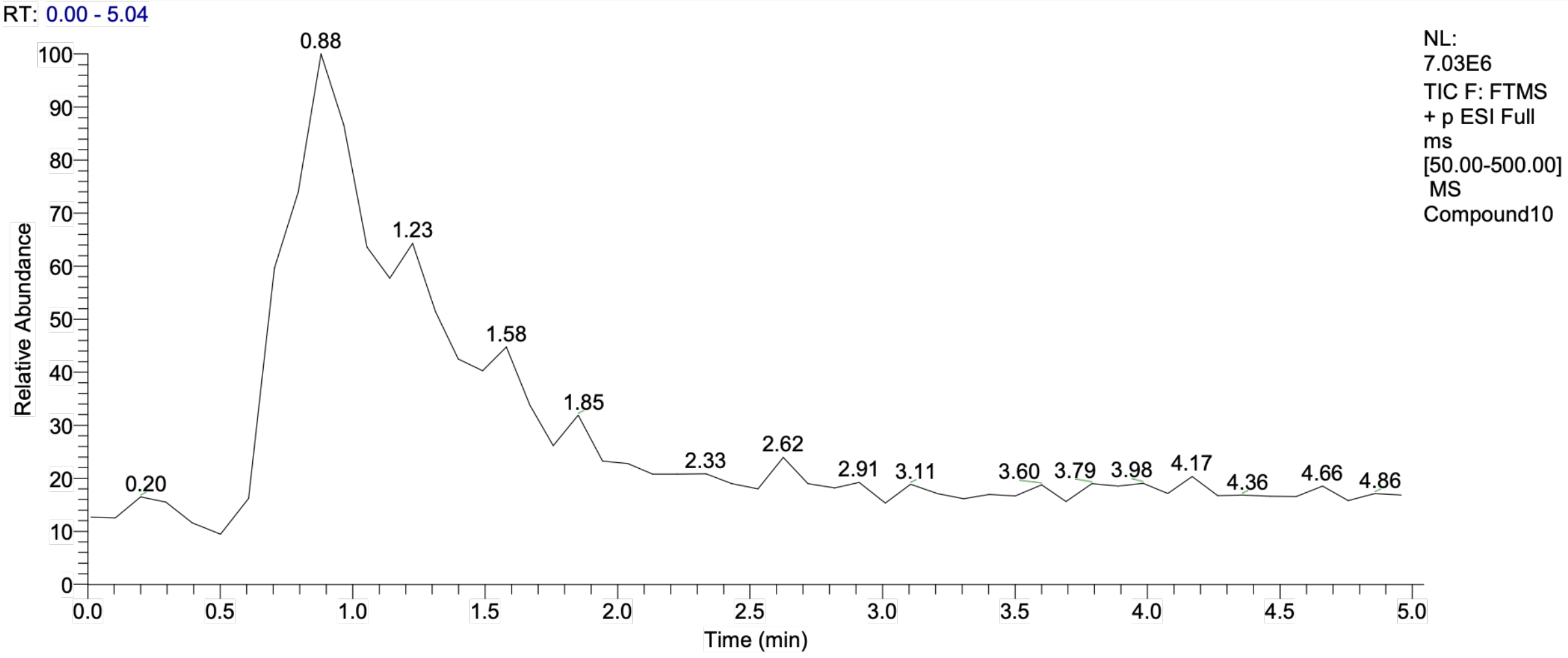

Compound10 #34-43 RT: 0.88-0.97 AV: 2 NL: 1.50E6
F: FTMS + p ESI Full ms [50.00-500.00]

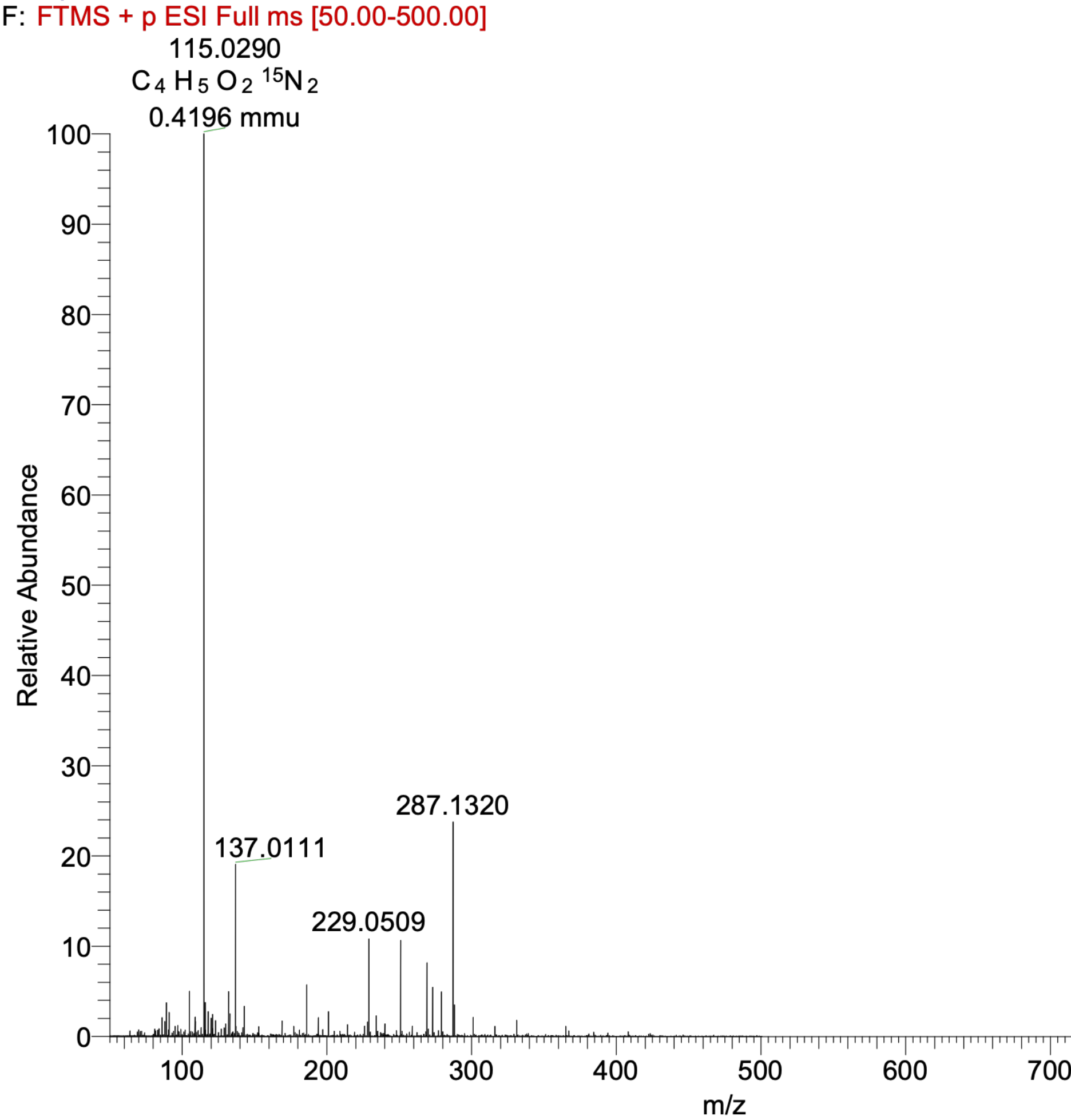

Exact Mass: 114,02135

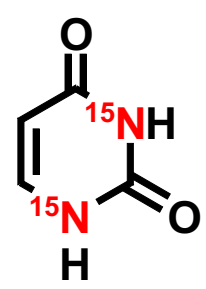

10

*O*2',3',5'-Tris(benzoyl)-(*N*[1],*N*[3]-^{15}N)-uridine (11)

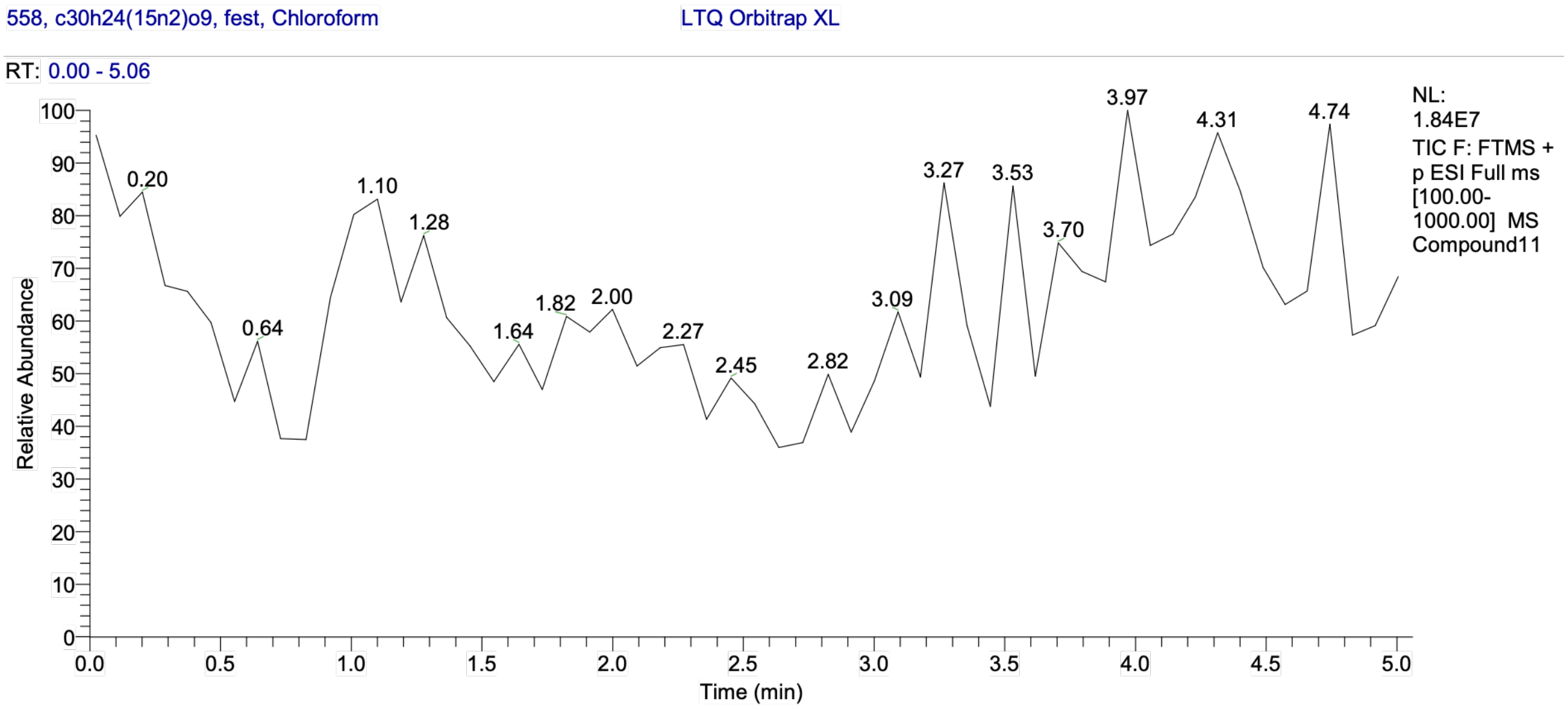

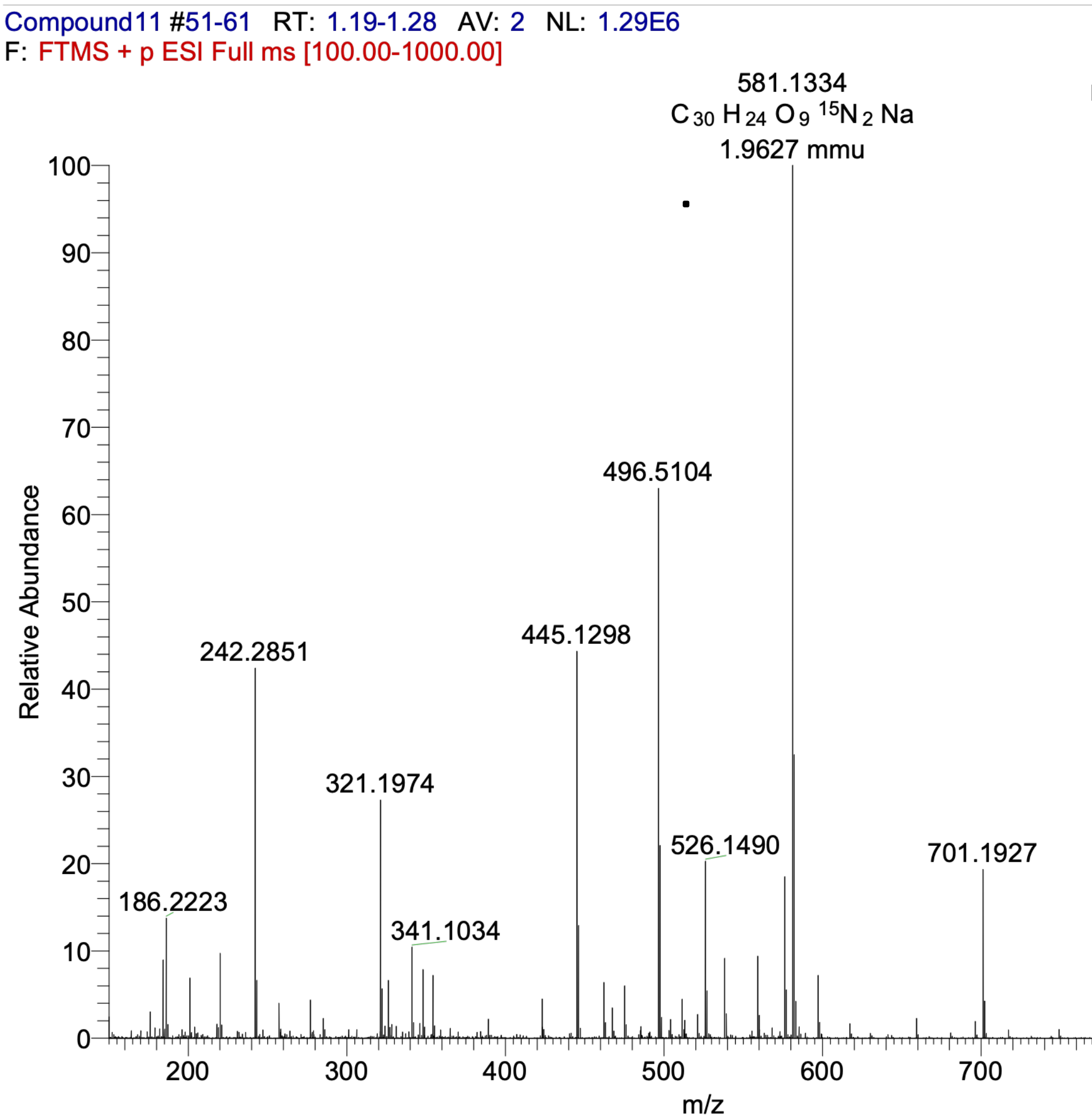

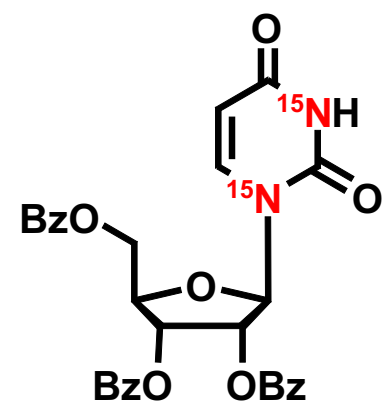

11

(N^1,N^3-^{15}N)-uridine (12)

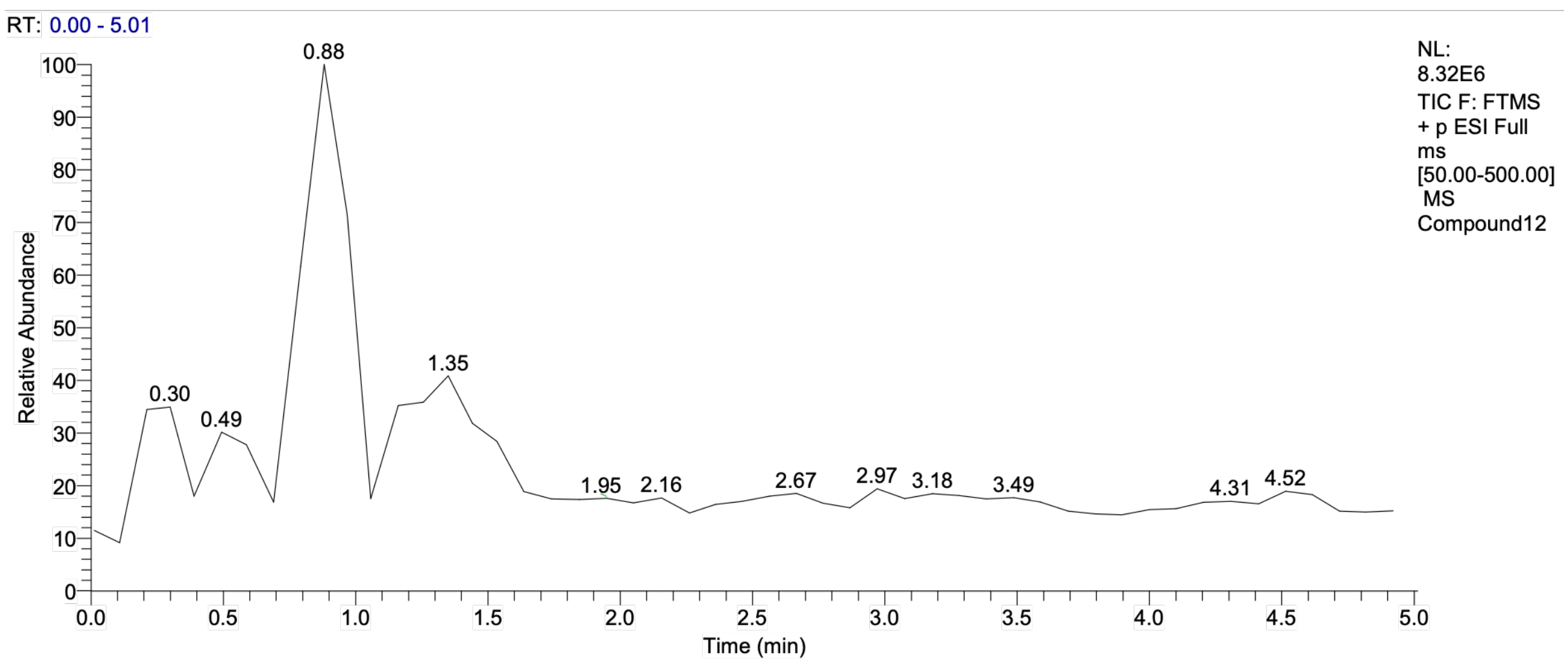

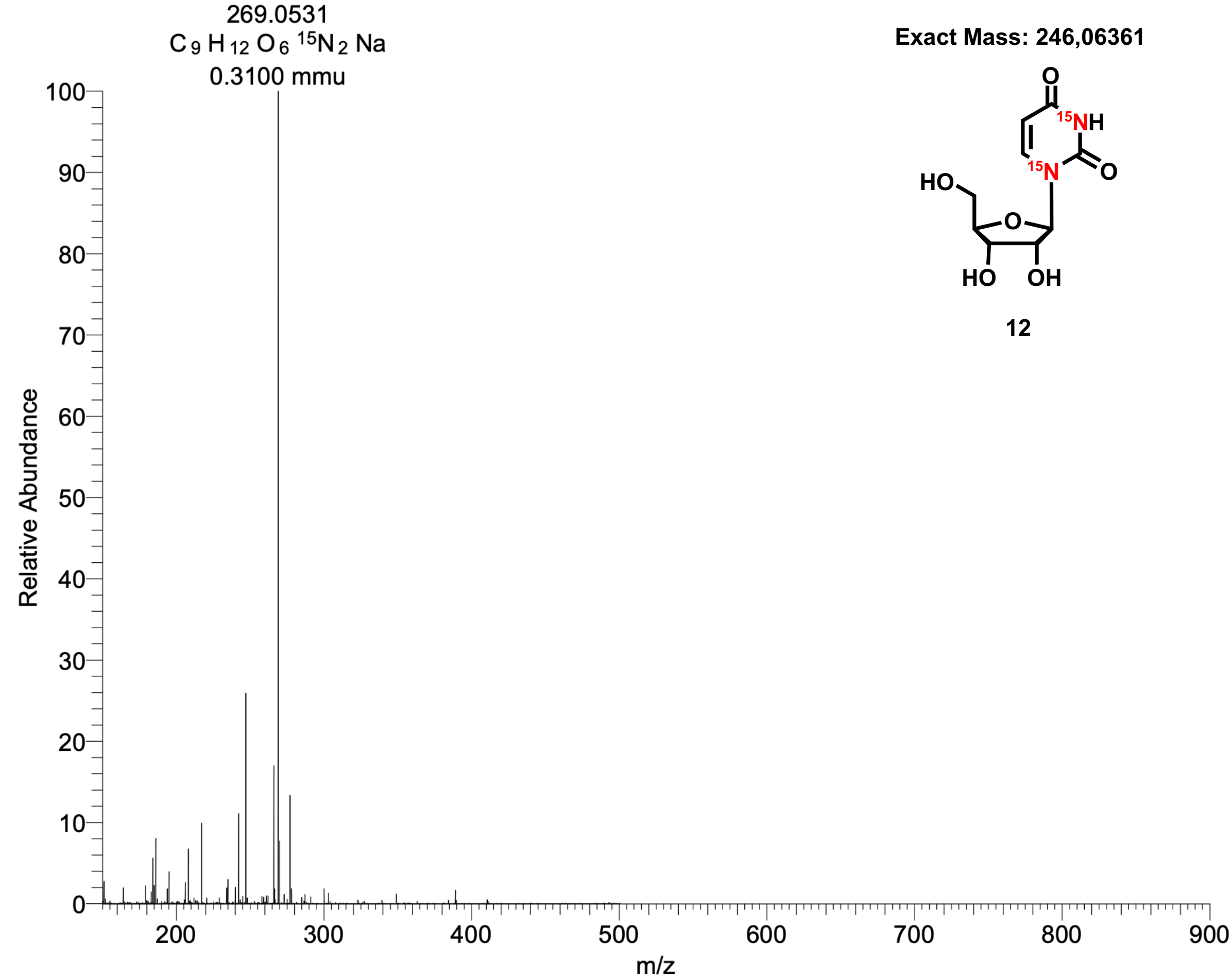

Exact Mass: 246,06361

12

*O*2',3',5'- Tris(*tert*-butyldimethylsilyl)-(N^1,N^3-^{15}N)-uridine (13)

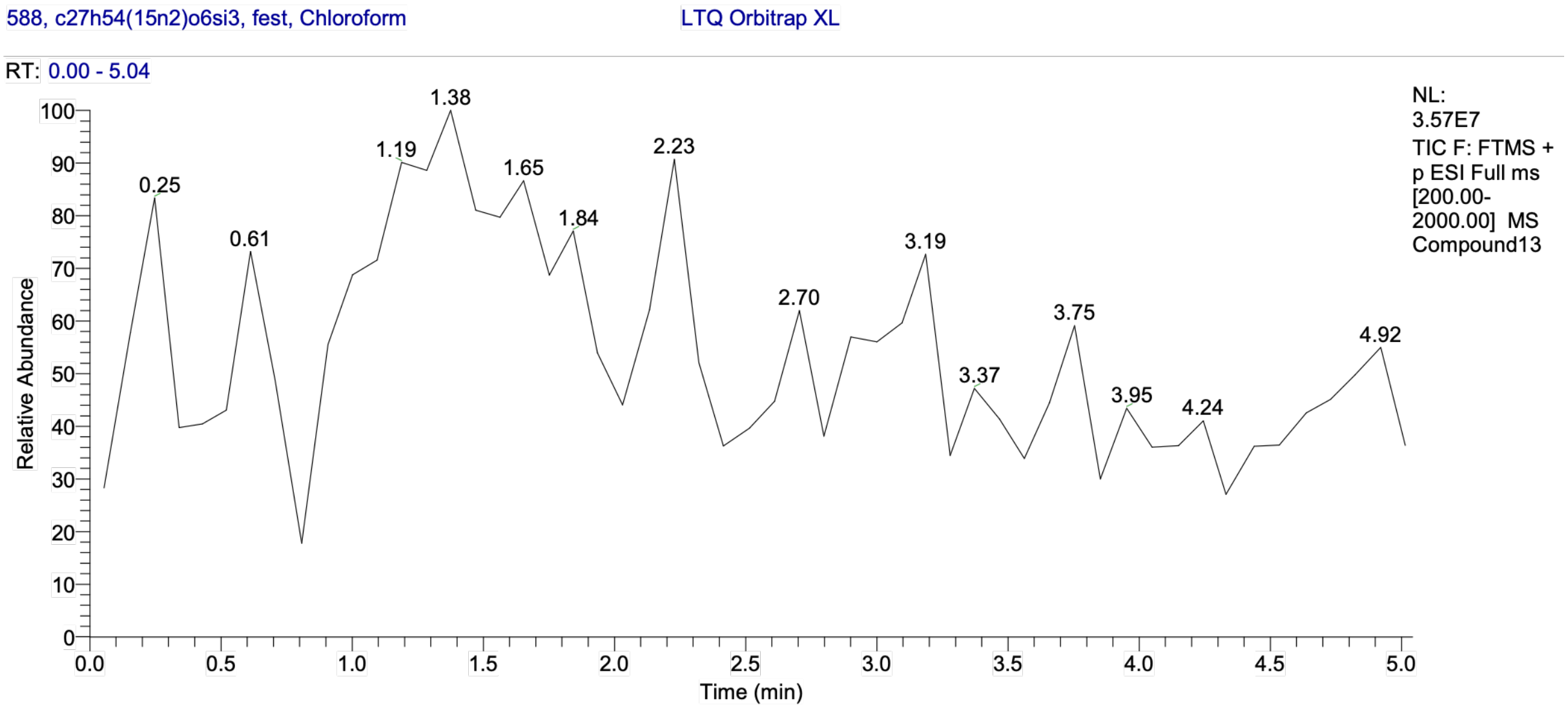

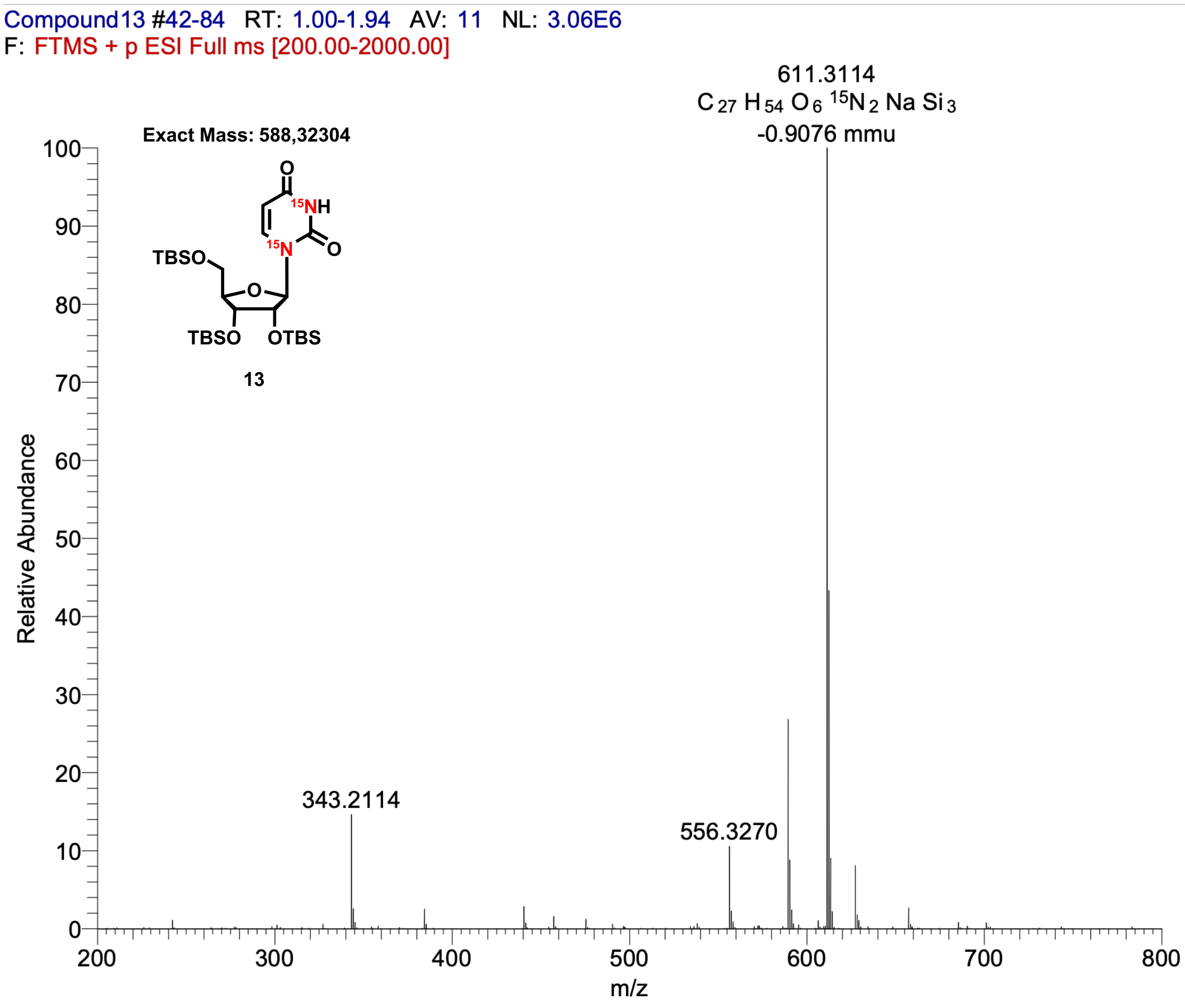

*O*2',3',5'- Tris(*tert*-butyldimethylsilyl)-(*N*[1],*N*[3],*N*[4]-^{15}N)-cytidine (14)

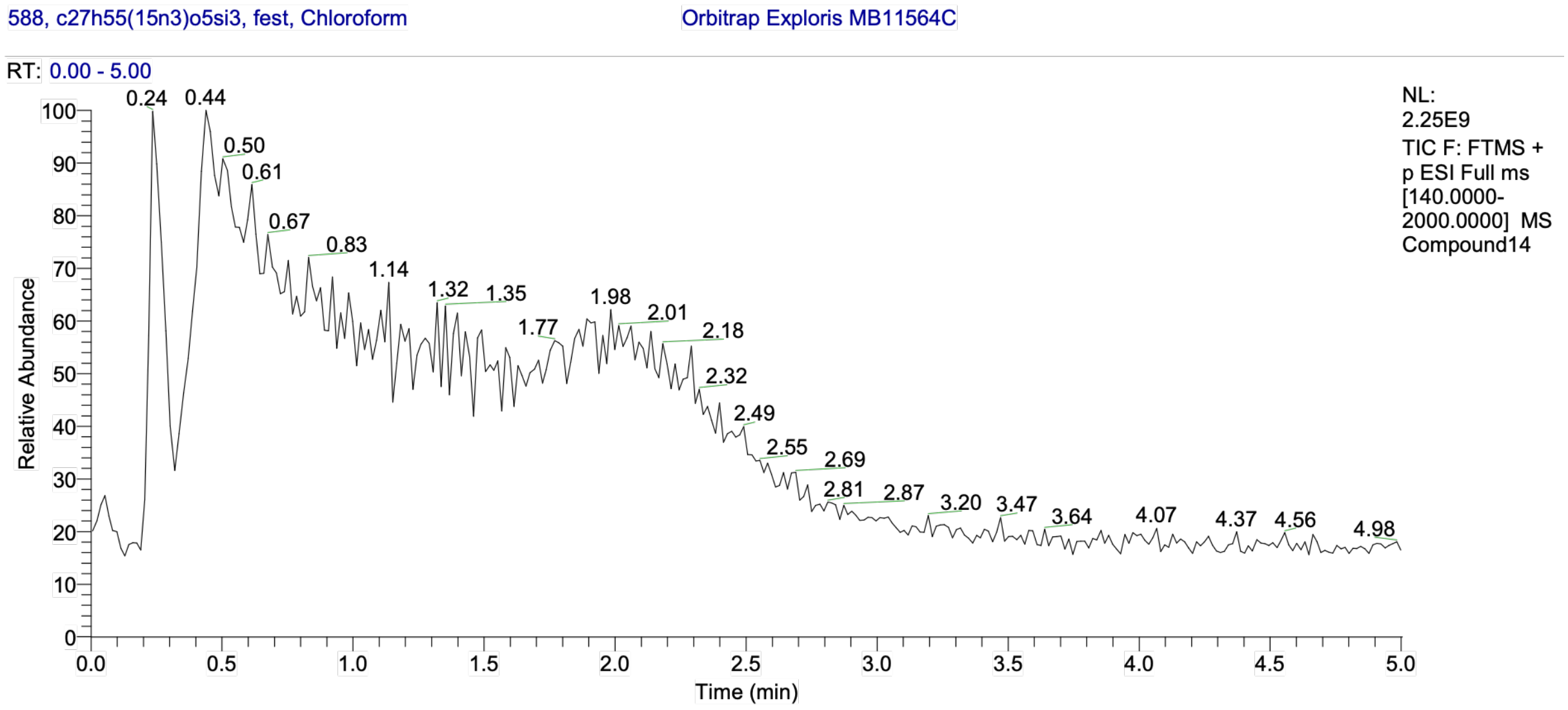

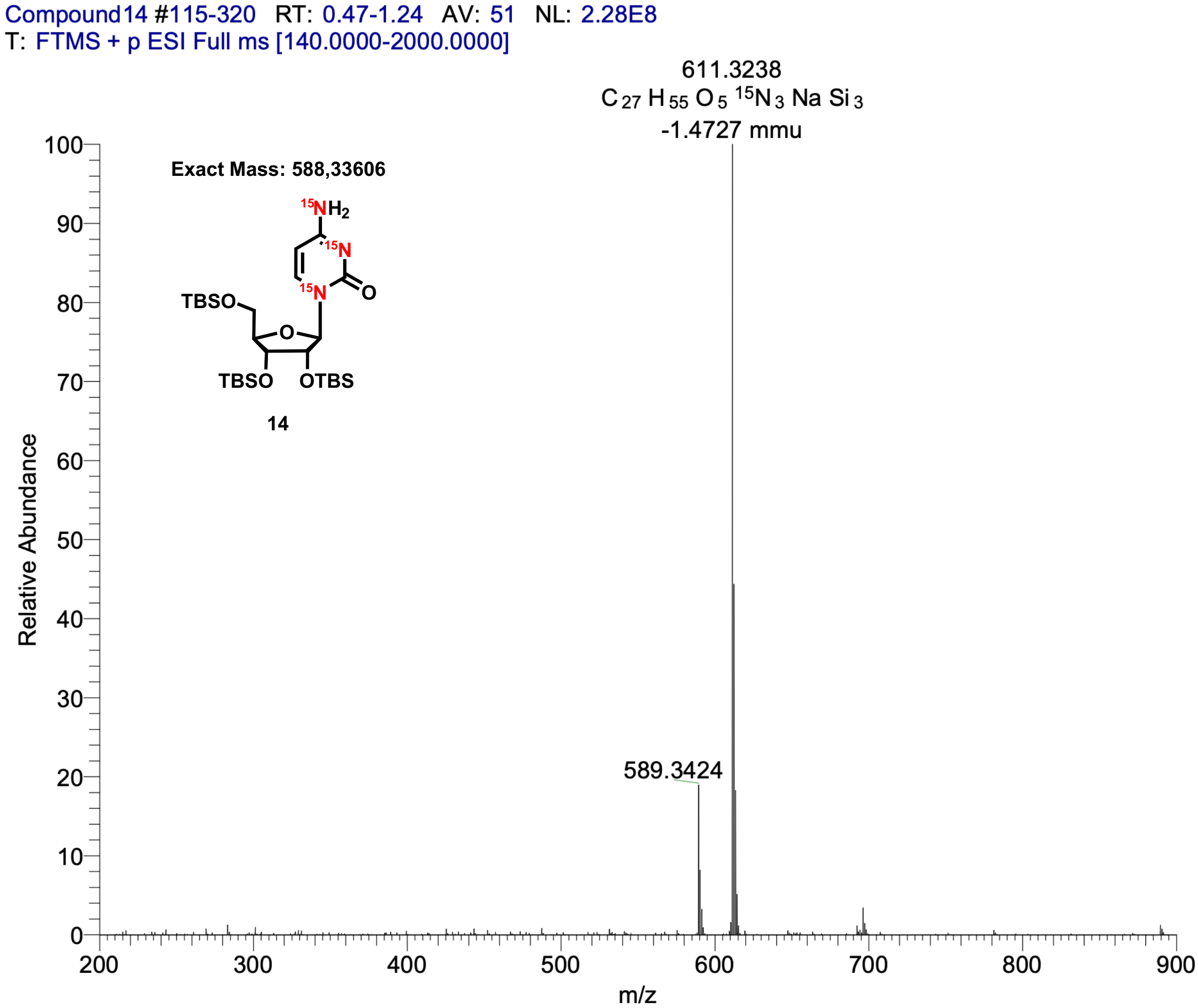

*O*2',3',5'-Tris(*tert*-butyldimethylsilyl)-5-iodo-(N^1,N^3,N^4-^{15}N)-cytidine (15)

714, c27h54i(15n3)o5si3, fest, Chloroform   Orbitrap Exploris MB11564C

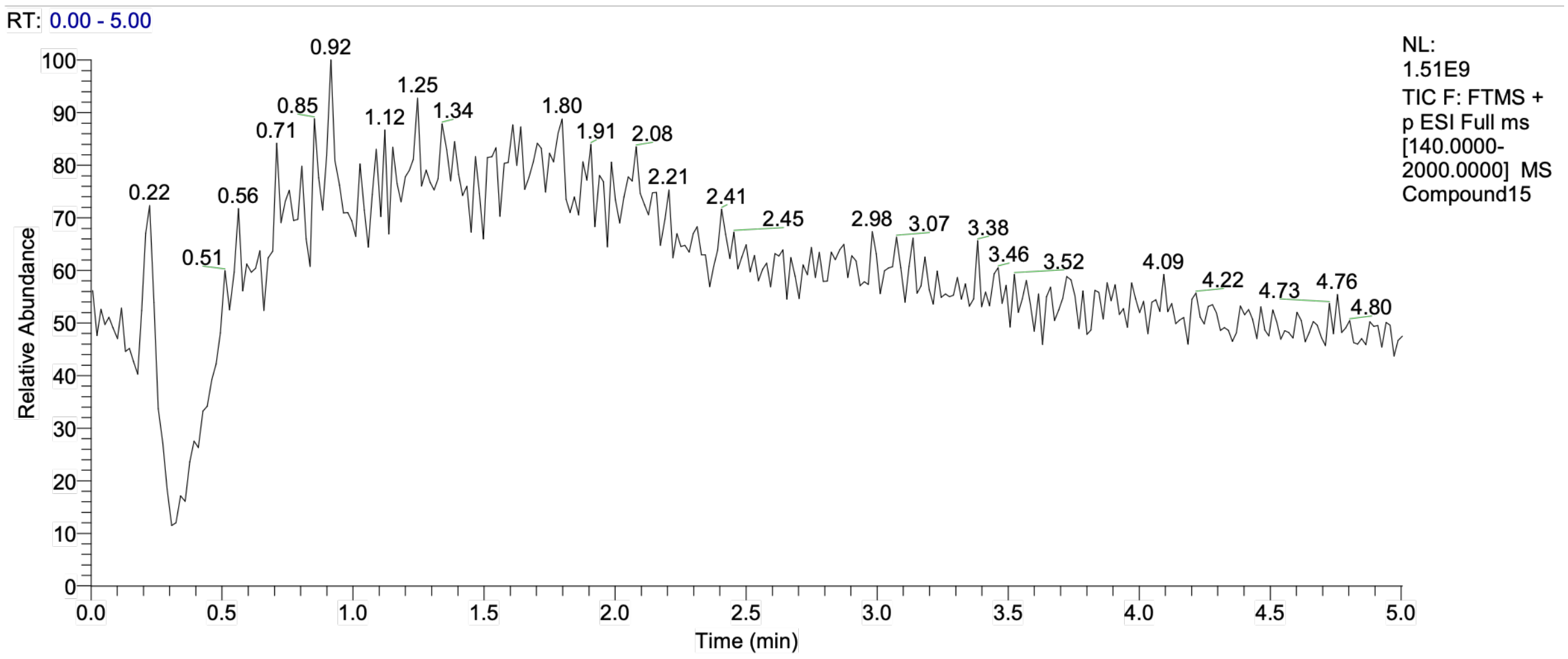

Compound15 #209-405 RT: 0.85-1.61 AV: 49 NL: 2.22E8
T: FTMS + p ESI Full ms [140.0000-2000.0000]

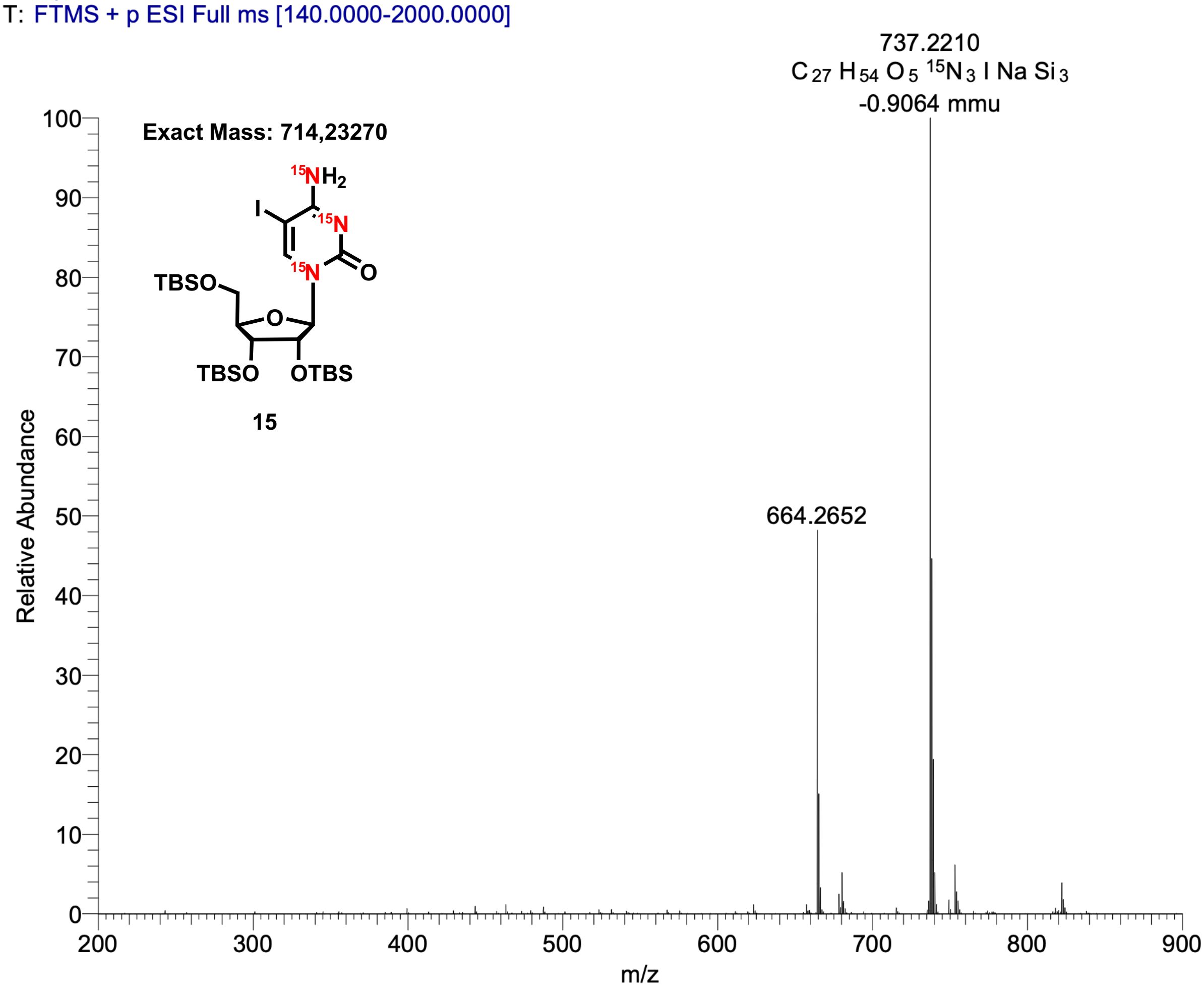

_O_2',3',5'-Tris(_tert_-butyldimethylsilyl)-5-(methoxycarbonyl)-(N^1,N^3,N^4-^{15}N)-cytidine (16)

646, c29h57(15n3)o7si3, fest, Chloroform

Orbitrap Exploris MB11564C

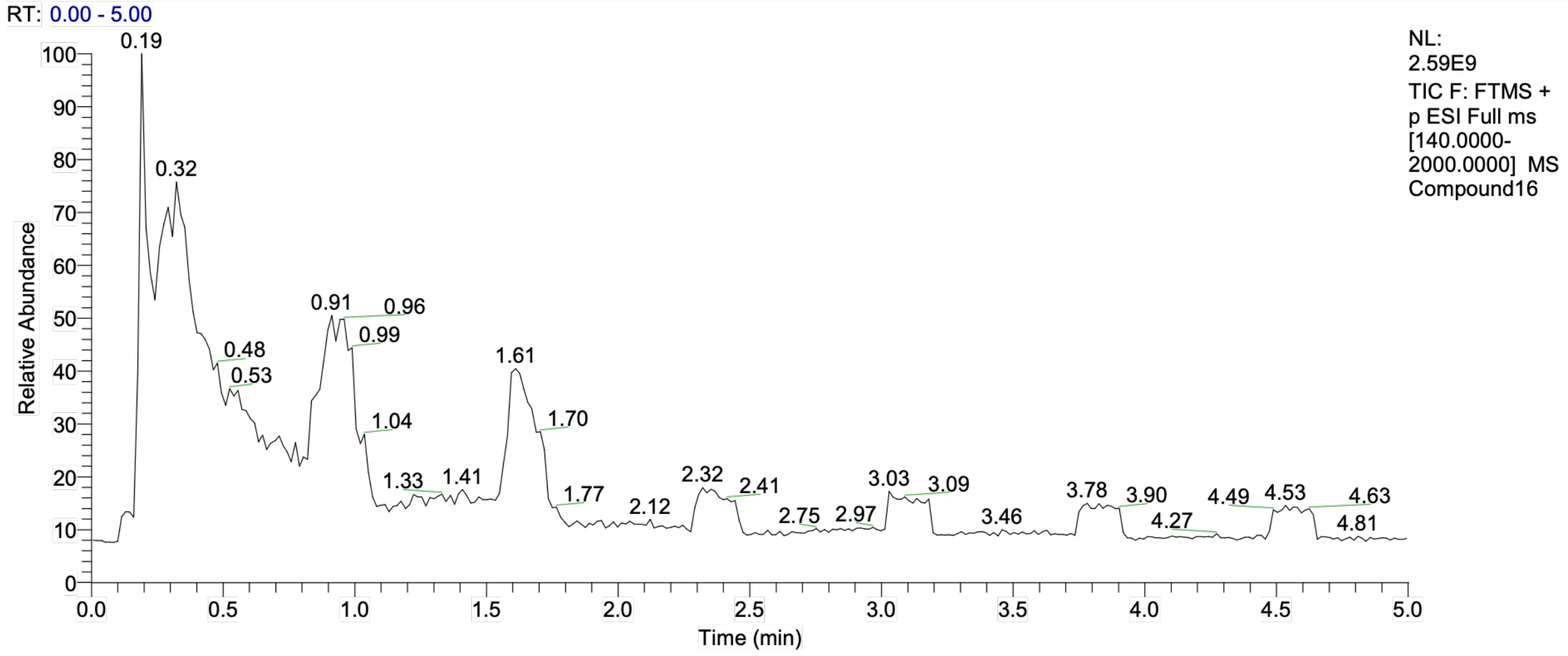

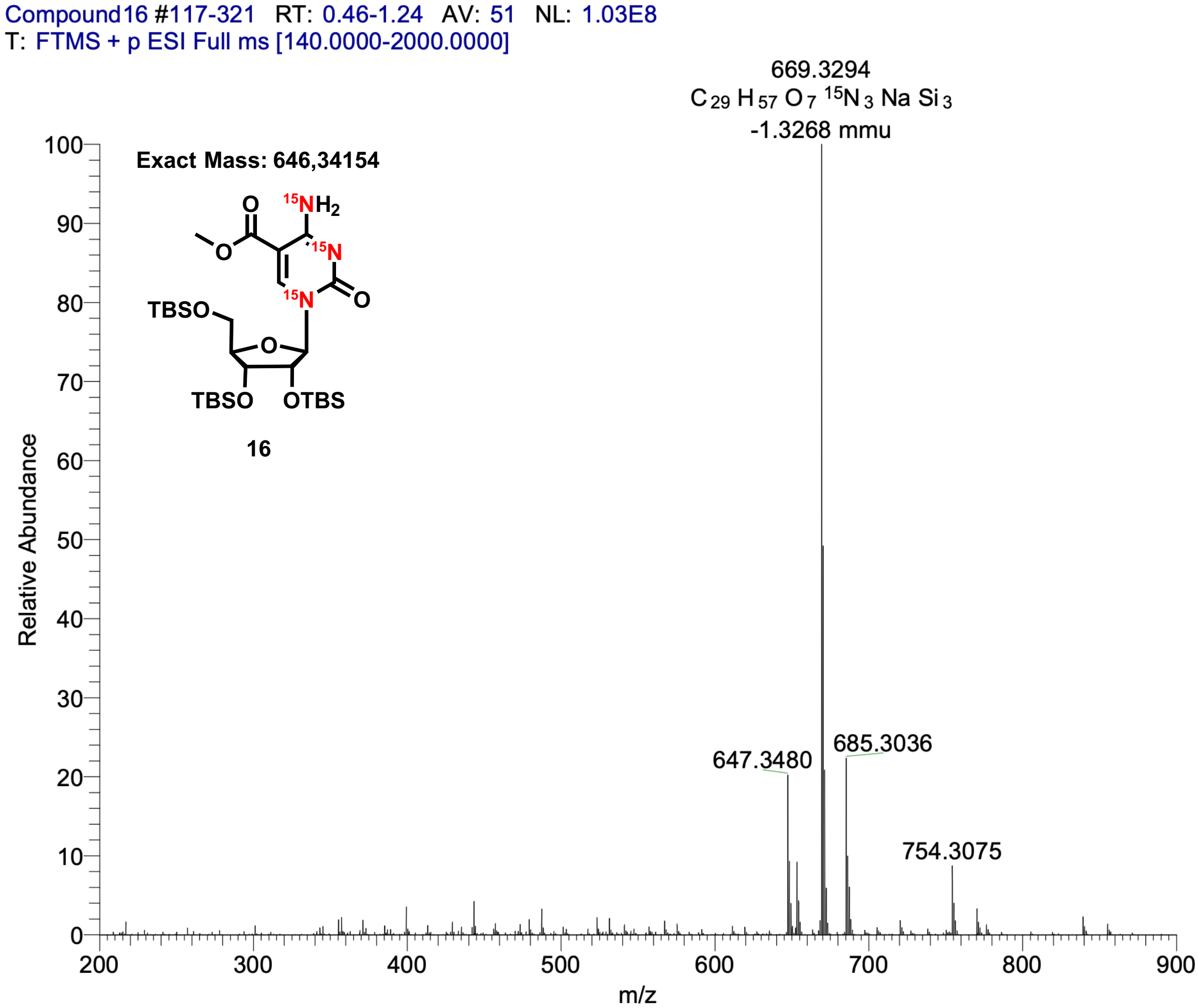

*O*2',3',5'-Tris(*tert*-butyldimethylsilyl)-5-formyl-(N^1,N^3,N^4-^{15}N)-cytidine (17)

616, c28h55(15n3)o6si3, fest, Chloroform   Orbitrap Exploris MB11564C

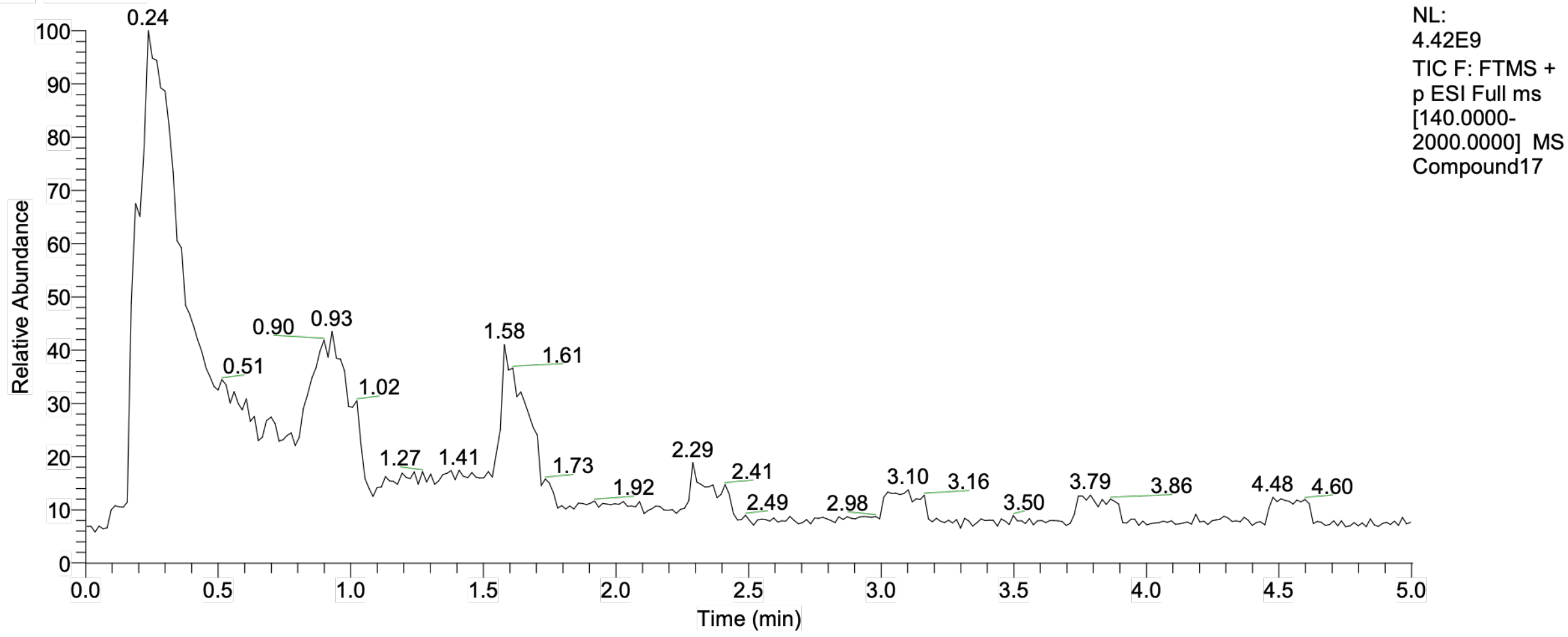

Compound17 #120-325 RT: 0.47-1.24 AV: 51 NL: 1.35E8
T: FTMS + p ESI Full ms [140.0000-2000.0000]

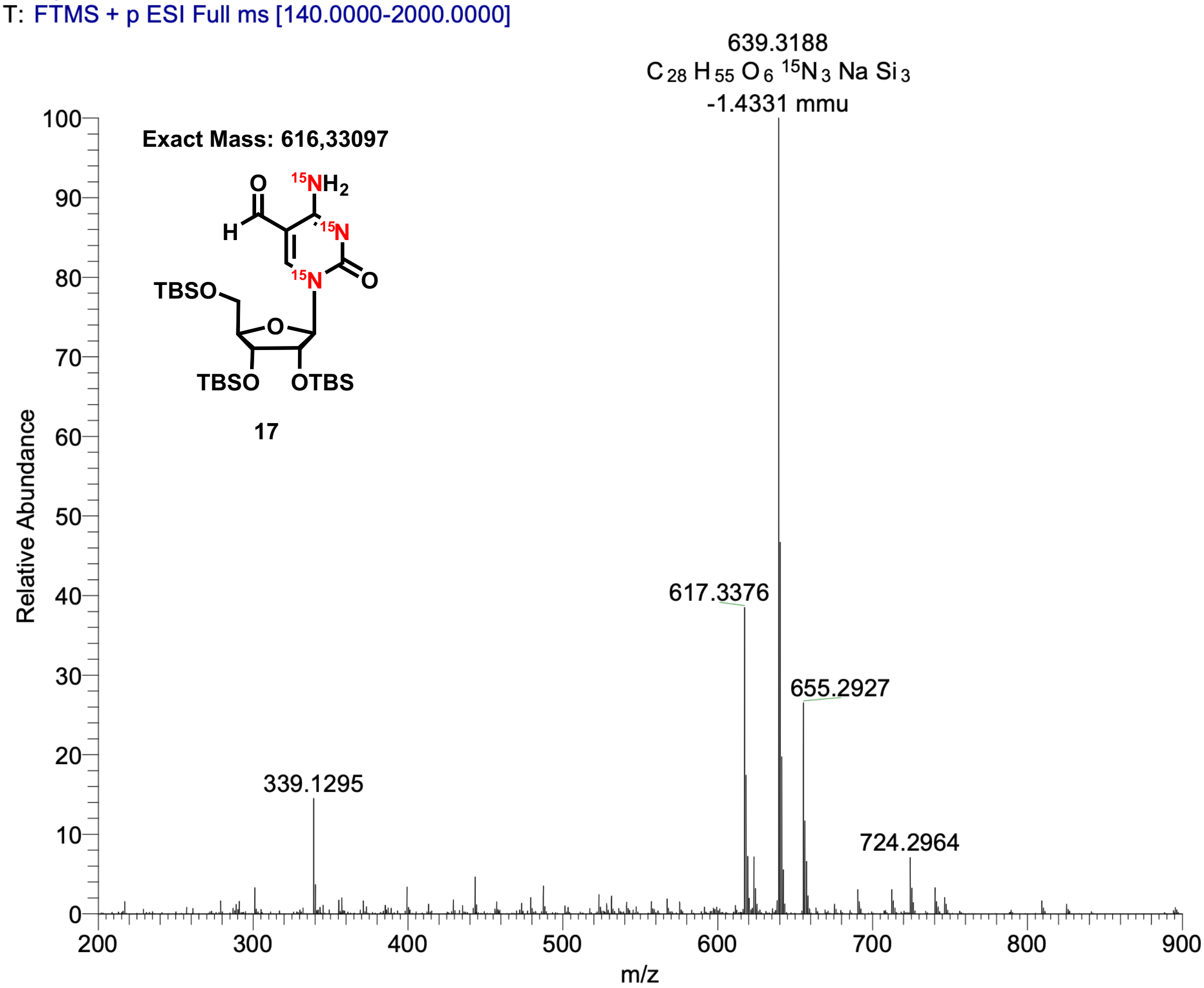

*O*2',3',5'-Tris(*tert*-butyldimethylsilyl)-5-hydroxymethyl-(N^1,N^3,N^4-^{15}N)-cytidine (18)

618, c28h57(15n3)o6si3, fest, Chloroform   Orbitrap Exploris MB11564C

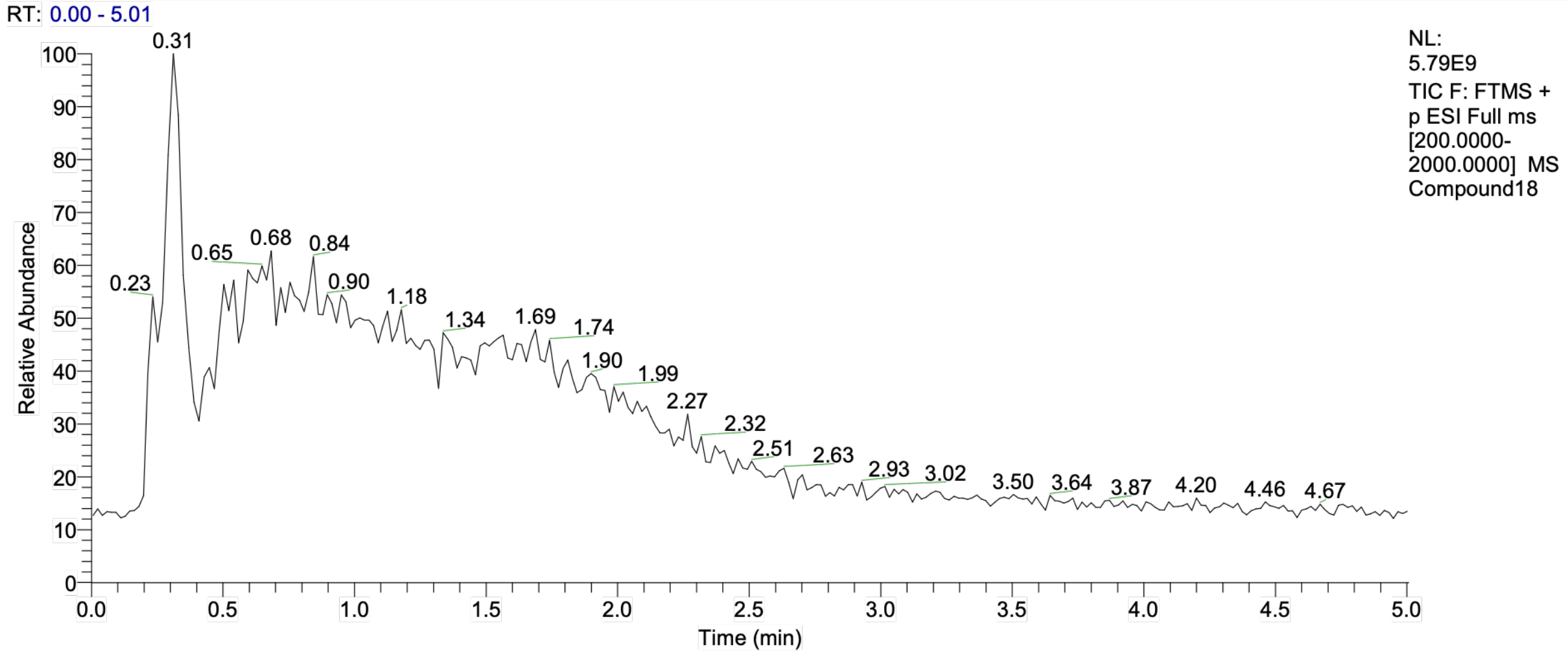

Compound18 #108-244 RT: 0.50-1.09 AV: 34 NL: 2.90E8
T: FTMS + p ESI Full ms [200.0000-2000.0000]

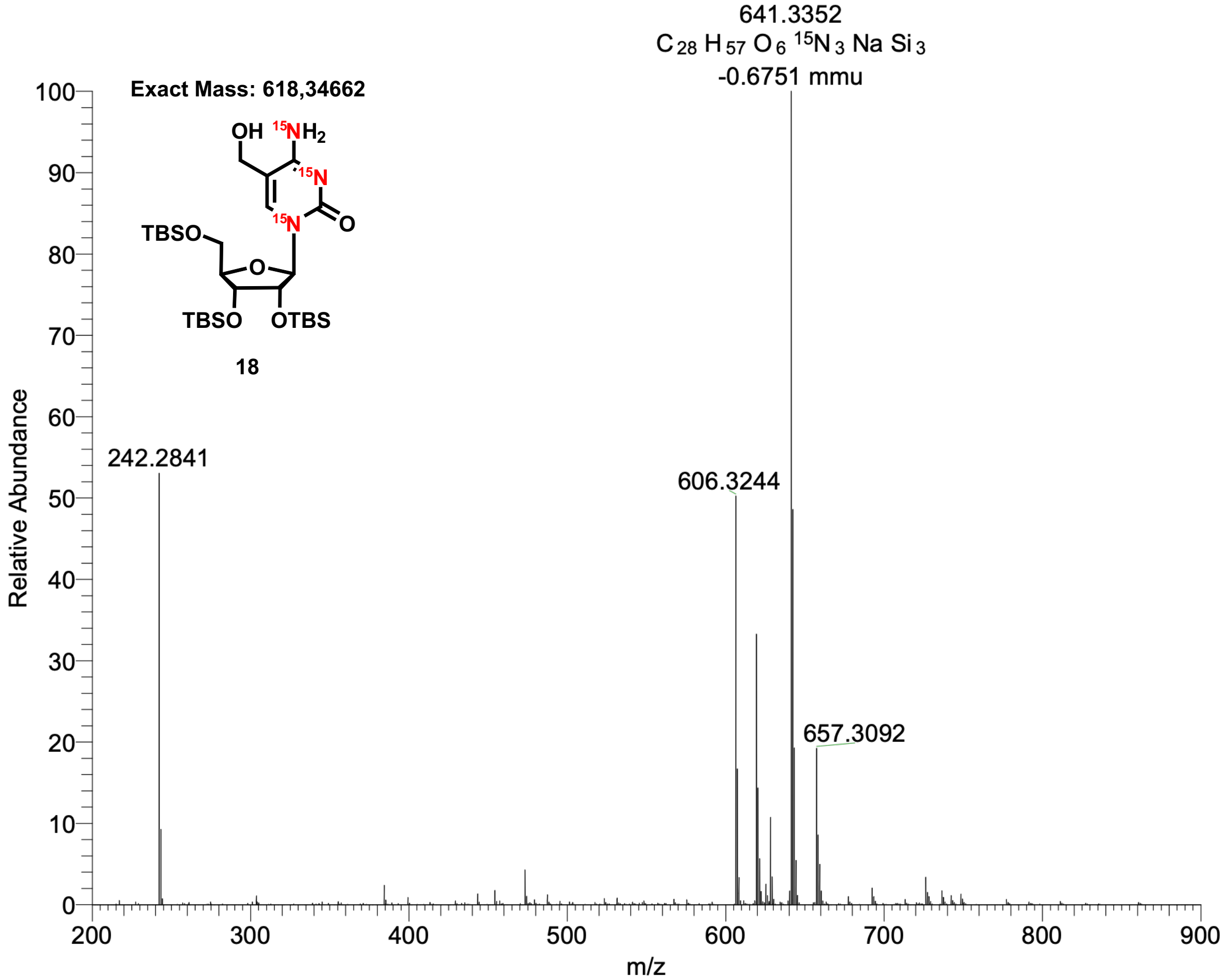

5-Hydroxymethyl-(N^1,N^3,N^4-^{15}N)-cytidine ([$^{15}N_3$]-hm5C)

274, c10h13(15n)3o6, geloest, Wasser Orbitrap Exploris MB11564C

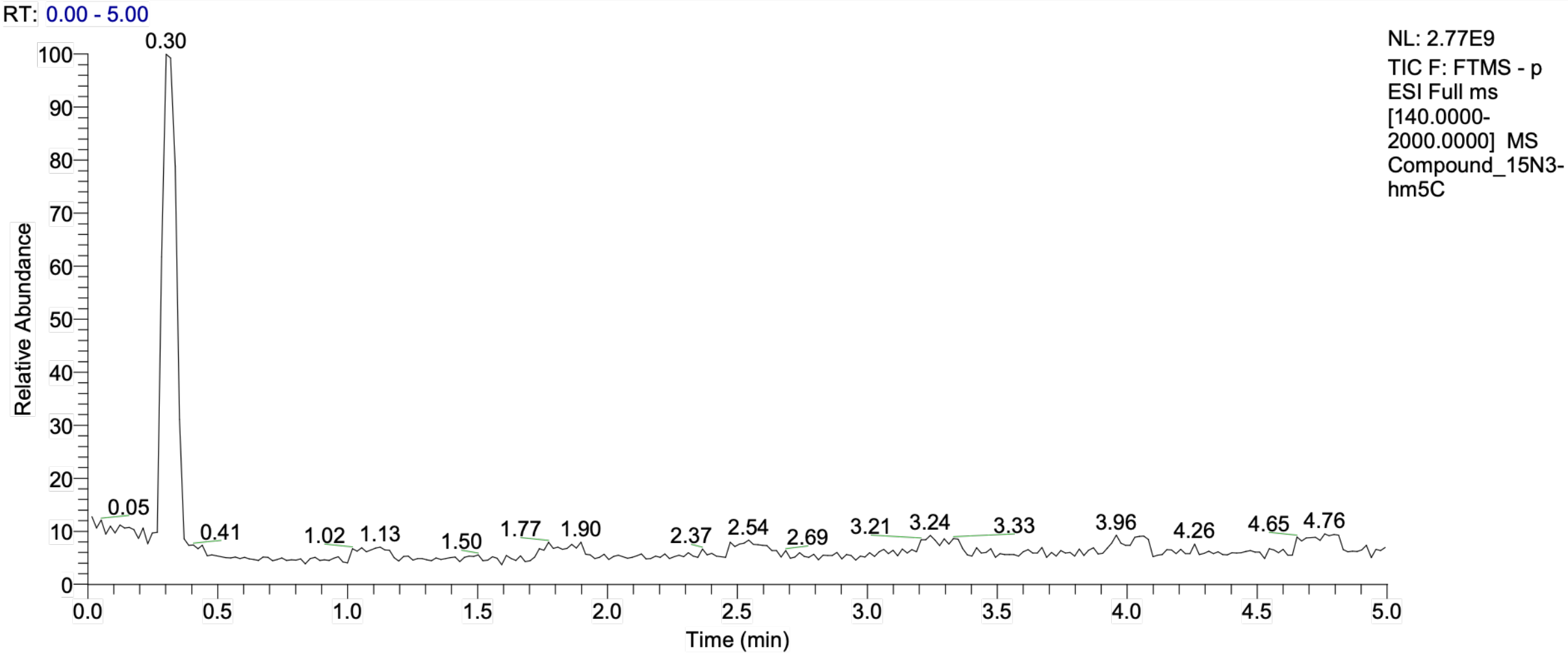

Compound_15N3-hm5C #67-80 RT: 0.30-0.35 AV: 4 NL: 1.63E8
T: FTMS - p ESI Full ms [140.0000-2000.0000]

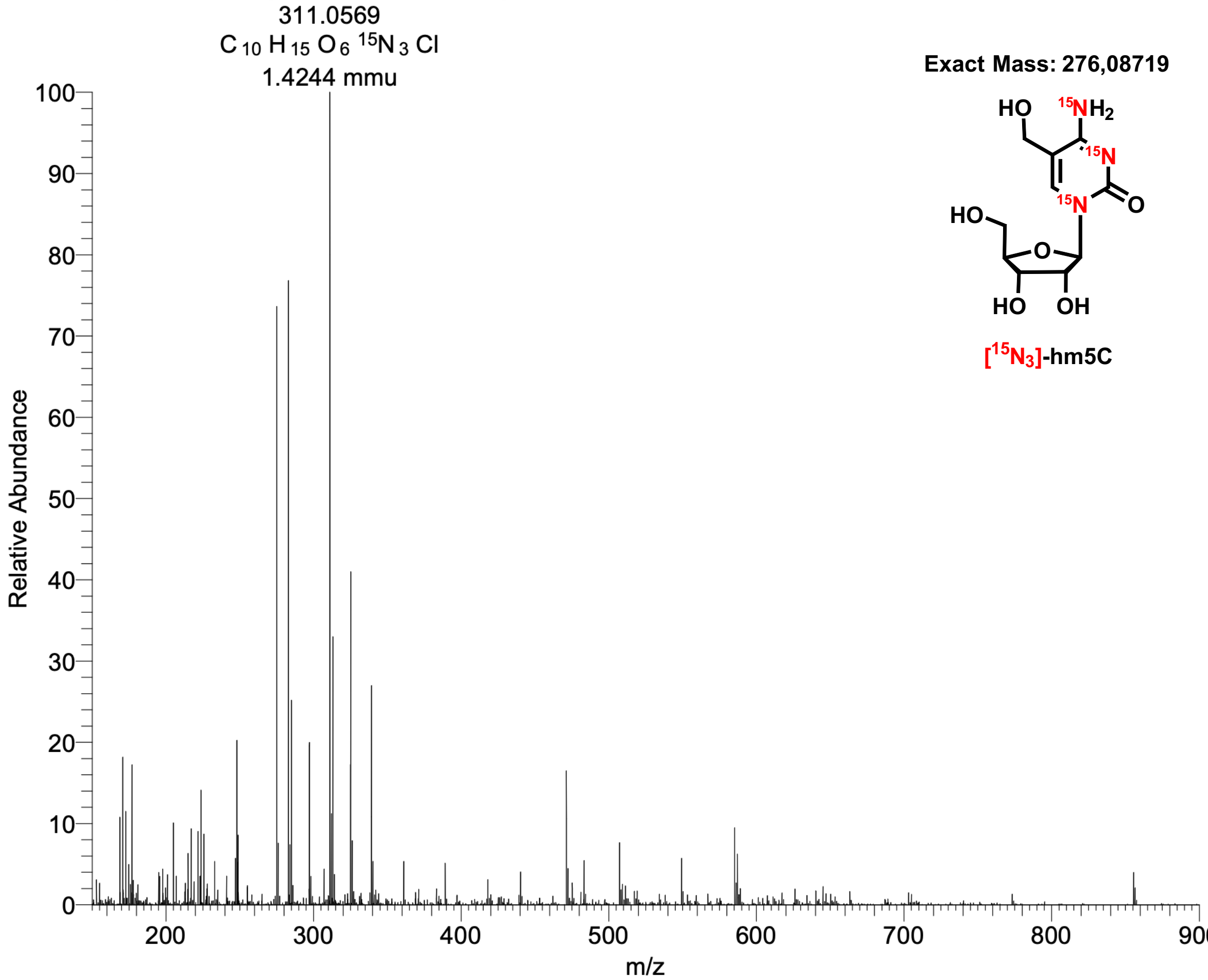

Exact Mass: 276,08719

[$^{15}N_3$]-hm5C

5-Formyl-(N^1,N^3,N^4-^{15}N)-cytidine ([$^{15}N_3$-f5C])

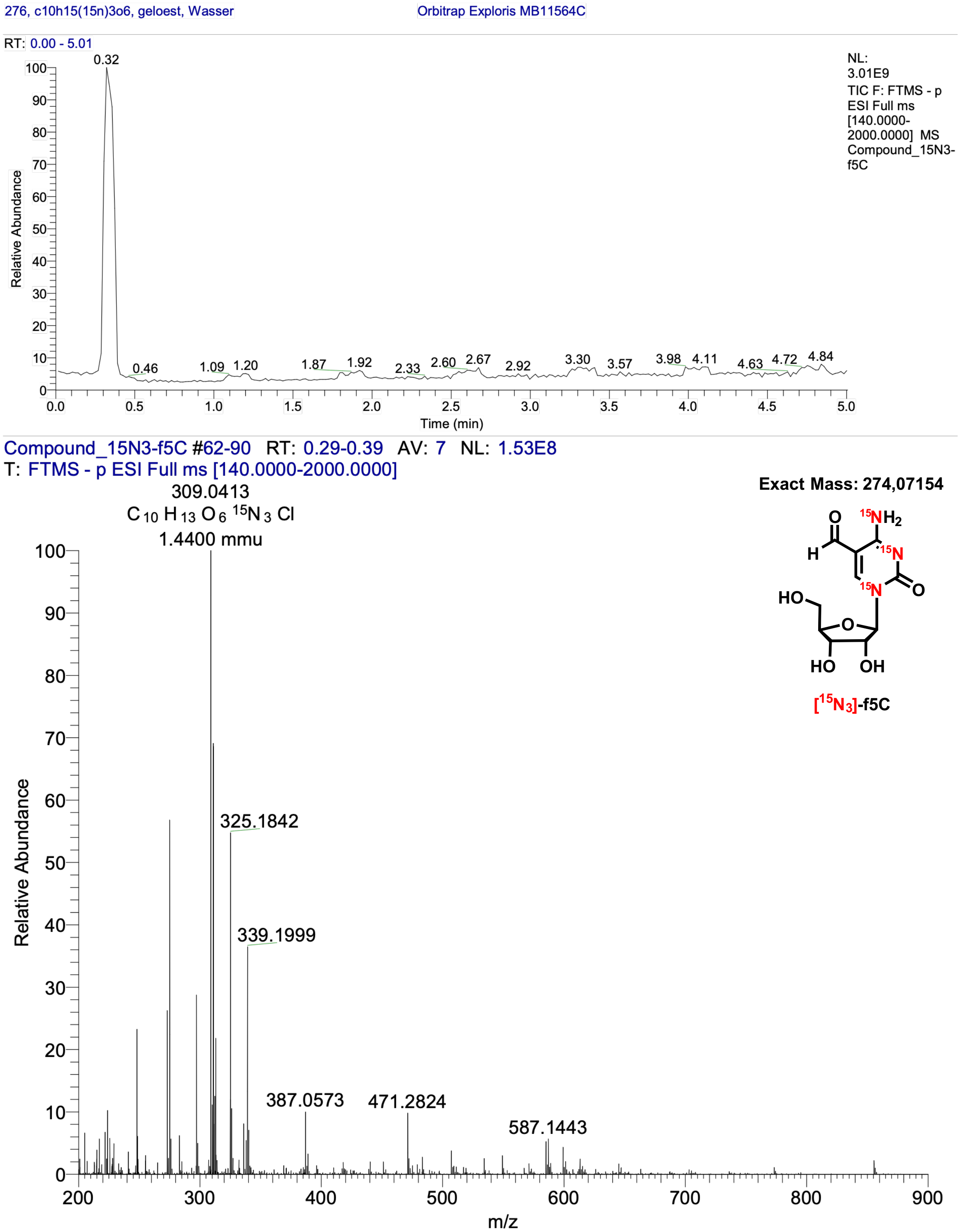

Exact Mass: 274,07154

[$^{15}N_3$]-f5C

5-Carboxyl-(N^1,N^3,N^4-^{15}N)-cytidine ([$^{15}N_3$-ca5C])

290, (15n3)c10h13o7, fest, Aceton	Orbitrap Exploris MB11564C

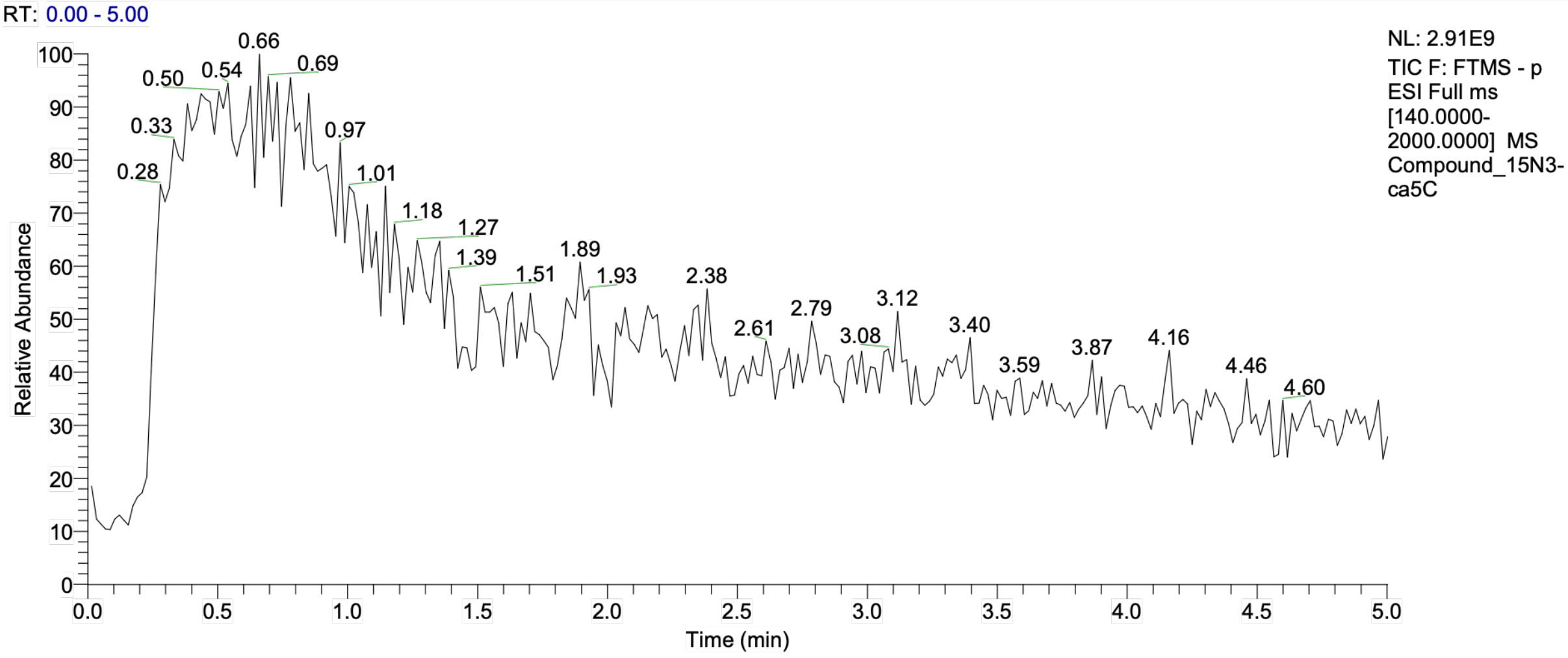

Compound_15N3-ca5C #62-240 RT: 0.28-1.04 AV: 45 NL: 7.15E8
T: FTMS - p ESI Full ms [140.0000-2000.0000]

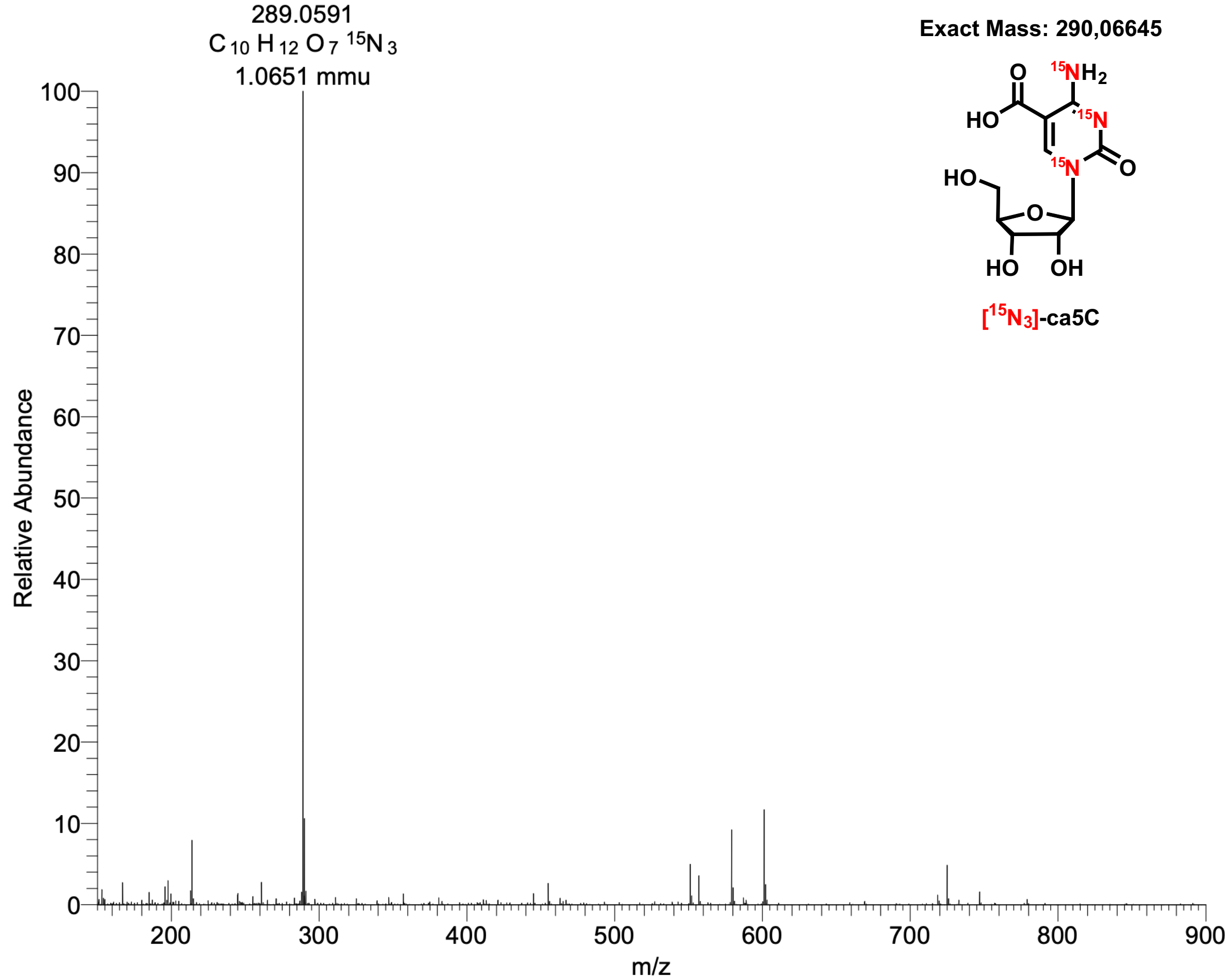

Supporting References

(1) Münzel, M.; Globisch, D.; Trindler, C.; Carell, T. Efficient Synthesis of 5-Hydroxymethylcytosine Containing DNA. Organic Letters 2010, 12 (24), 5671–5673. https://doi.org/10.1021/ol102408t.

(2) Hisamatsu, Y.; Hasada, K.; Amano, F.; Tsubota, Y.; Wasada-Tsutsui, Y.; Shirai, N.; Ikeda, S.; Odashima, K. Highly Selective Recognition of Adenine Nucleobases by Synthetic Hosts with a Linked Five-Six-Five-Membered Triheteroaromatic Structure and the Application to Potentiometric Sensing of the Adenine Nucleotide. Chemistry 2006, 12 (29), 7733–7741. https://doi.org/10.1002/chem.200600099.

(3) Michaelides, I. N.; Tago, N.; Viverge, B.; Carell, T. Synthesis of RNA Containing 5-Hydroxymethyl-, 5-Formyl-, and 5-Carboxycytidine. Chemistry 2017, 23 (63), 15894–15898. https://doi.org/10.1002/chem.201704216.

(4) Schröder, A. S.; Kotljarova, O.; Parsa, E.; Iwan, K.; Raddaoui, N.; Carell, T. Synthesis of (R)-Configured 2′-Fluorinated mC, hmC, fC, and caC Phosphoramidites and Oligonucleotides. Organic Letters 2016, 18 (17), 4368–4371. https://doi.org/10.1021/acs.orglett.6b02110.

(5) Schiesser, S.; Pfaffeneder, T.; Sadeghian, K.; Hackner, B.; Steigenberger, B.; Schroder, A. S.; Steinbacher, J.; Kashiwazaki, G.; Hofner, G.; Wanner, K. T.; Ochsenfeld, C.; Carell, T. Deamination, Oxidation, and C-C Bond Cleavage Reactivity of 5-Hydroxymethylcytosine, 5-Formylcytosine, and 5-Carboxycytosine. Journal of the American Chemical Society 2013, 135 (39), 14593–14599. https://doi.org/10.1021/ja403229y.

(6) Münzel, M.; Globisch, D.; Brückl, T.; Wagner, M.; Welzmiller, V.; Michalakis, S.; Müller, M.; Biel, M.; Carell, T. Quantification of the Sixth DNA Base Hydroxymethylcytosine in the Brain. Angewandte Chemie 2010, 49 (31), 5375–5377. https://doi.org/10.1002/anie.201002033.

(7) Patching, S. G.; Middleton, D. A.; Henderson, P. J. F.; Herbert, R. B. The Economical Synthesis of [2′-13C, 1,3-15N2]Uridine; Preliminary Conformational Studies by Solid State NMR. Organic & Biomolecular Chemistry 2003, 1 (12), 2057–2062. https://doi.org/10.1039/B301275A.

(8) Strebitzer, E.; Rangadurai, A.; Plangger, R.; Kremser, J.; Juen, M. A.; Tollinger, M.; Al-Hashimi, H. M.; Kreutz, C. 5-Oxyacetic Acid Modification Destabilizes Double Helical Stem Structures and Favors Anionic Watson–Crick like cmo5U-G Base Pairs. Chemistry 2018, 24 (71), 18903–18906. https://doi.org/10.1002/chem.201805077.

(9) Schön, A.; Kaminska, E.; Schelter, F.; Ponkkonen, E.; Korytiaková, E.; Schiffers, S.; Carell, T. Analysis of an Active Deformylation Mechanism of 5-Formyl-Deoxycytidine (fdC) in Stem Cells. Angewandte Chemie 2020, 59 (14), 5591–5594. https://doi.org/10.1002/anie.202000414.

(10) Jonasson, N. S. W. Functional models for iron(II)/α-keto acid dependent enzymes. PhDThesis, Ludwig-Maximilians-Universität München, 2021. https://edoc.ub.uni-muenchen.de/28981/ (accessed 2025-10-06).

(11) Lindlar né Jonasson, N. S. W.; Menke, A.; Senft, L.; Squarcina, A.; Schmidl, D.; Fisher, K.; Demeshko, S.; Kruse, J. C.; Josephy, T.; Mayer, P.; Gutenthaler-Tietze, J.; Comba, P.; Meyer, F.; Ivanović-Burmazović, I.; Daumann, L. J. Two Plus Four Equals Three–Iron(II)/Iron(IV) Comproportionation as an Additional Pathway for Iron(IV)-Oxido Reactions. Inorganic Chemistry 2025, 64 (8), 3719–3734. https://doi.org/10.1021/acs.inorgchem.4c04518.
